# Rearrangement of *o*-(pivaloylaminomethyl)benzaldehydes: an experimental and computational study

**DOI:** 10.3762/bjoc.16.136

**Published:** 2020-07-13

**Authors:** Csilla Hargitai, Györgyi Koványi-Lax, Tamás Nagy, Péter Ábrányi-Balogh, András Dancsó, Gábor Tóth, Judit Halász, Angéla Pandur, Gyula Simig, Balázs Volk

**Affiliations:** 1 Directorate of Drug Substance Development, Egis Pharmaceuticals Plc., P.O. Box 100, H-1475 Budapest, Hungary; 2 Medicinal Chemistry Research Group, Research Centre for Natural Sciences, P.O. Box 286, H-1519 Budapest, Hungary; 3 Department of Inorganic and Analytical Chemistry, Budapest University of Technology and Economics, Szent Gellért tér 4, H-1111 Budapest, Hungary

**Keywords:** DFT calculations, NMR, reaction mechanism, rearrangement, ring-chain tautomerism

## Abstract

Treatment of alkoxy-substituted *o*-(pivaloylaminomethyl)benzaldehydes under acidic conditions resulted in the formation of the regioisomeric aldehydes and/or dimer-like products. Detailed NMR studies and single-crystal X-ray measurements supported the structure elucidation of the compounds. DFT calculations were also carried out to clarify the reaction mechanism, and to explain the observed product distributions and structural variances in the dimer-like products. Studies on the transformation of unsubstituted *o*-(pivaloylaminomethyl)benzaldehyde under similar conditions were presented as well.

## Introduction

**Scheme 1:**
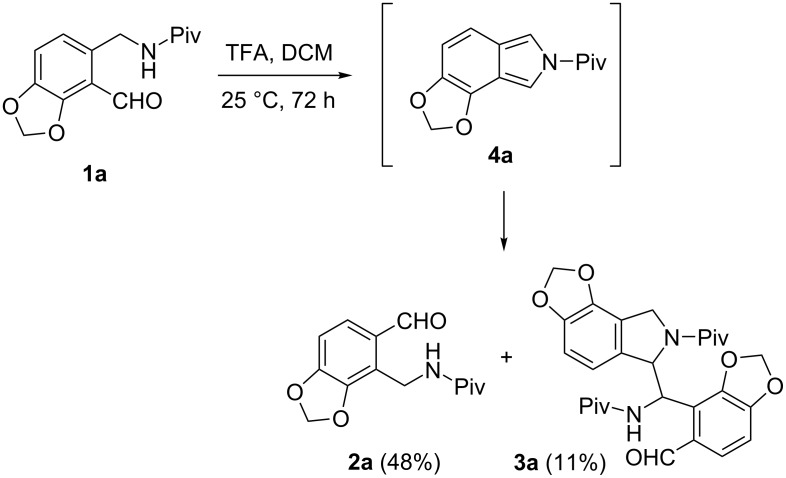
Rearrangement of methylenedioxy-substituted aminoaldehyde **1a** to regioisomer **2a** and formation of the dimer-like product **3a**.

In a preliminary publication [1] we disclosed that methylenedioxy-substituted *o*-(pivaloylaminomethyl)benzaldehyde (**1a**), when kept in dichloromethane (DCM) in the presence of a catalytic amount (0.13 equiv) of trifluoroacetic acid (TFA) for 72 h at room temperature, gave isomeric aldehyde **2a** (48%) and the dimer-like racemic product **3a** (11%). Both transformations were rationalized by the intermediacy of the isoindole **4a** (Scheme 1).

The formation of aldehyde **2a** can be explained by a protonation of the ring tautomer **1a**, followed by an acid-catalyzed water elimination to afford intermediate isoindole **4a** [2–6]. Then, the addition of water to the sterically less-hindered site of the latter compound followed by ring opening resulted in the rearranged aldehyde **2a** [1].

**Scheme 2:**
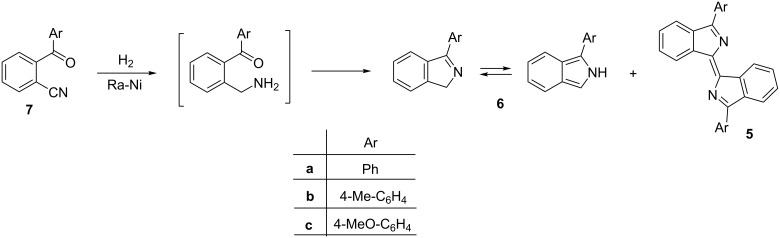
Synthesis of 1-arylisoindoles **6** and formation of dimers **5**.

As regards the formation of dimer-like product **3a**, the tendency of pyrrole [7–8], indole [8], and isoindole [3,9–10] to dimerize and polymerize was observed long ago. A repeatedly mentioned example was the formation of type **5** dimers under various conditions. They were first isolated during the synthesis of 1-arylisoindoles **6** by hydrogenation of *o*-cyanobenzophenones **7** in the presence of Raney nickel (Ra-Ni), sometimes as the main product, depending on the activity of the catalyst [10] (Scheme 2).

The oxidative dimerization of 1-phenylisoindole (**6a**) to compound **5a** took place under various conditions: when refluxing in benzene in the presence of air [5,11], during an attempted nitrosation (with sodium nitrite in acetic acid), and in Mannich reactions [12]. For the formation of dimers **5**, various mechanisms were envisaged. Nevertheless, the formation of any dimer-like product structurally similar to **3a** has not been observed so far.

In this report we describe our efforts to extend the reaction described in Scheme 1 to further *o*-(pivaloylaminomethyl)benzaldehydes and to support the results by density functional theory (DFT) calculations, single-crystal X-ray measurements and comprehensive NMR studies.

## Results and Discussion

### Acid-catalyzed transformations of compounds **1a–d**

**Scheme 3:**
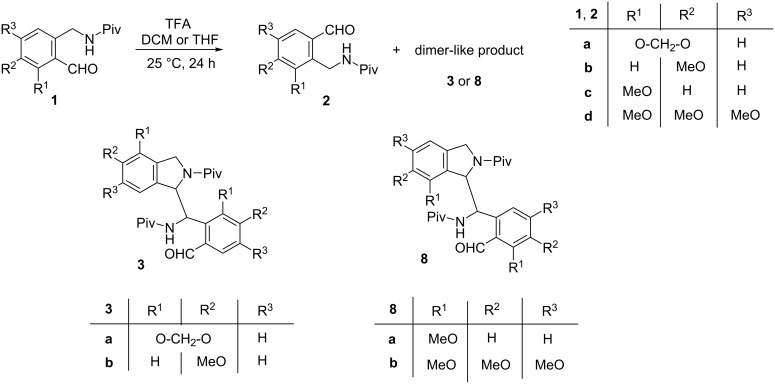
Rearrangement of aminoaldehydes **1** to regioisomers **2** and formation of dimer-like products **3** and **8**.

First we kept compounds **1a** and **1b** [2] in DCM in the presence of a catalytic amount (0.1 equiv) of TFA for 24 h at room temperature (Scheme 3) and the results are summarized in Table 1. In the case of aldehyde **1a**, the yields obtained under these conditions were similar to those of the reaction mentioned above (Scheme 1). The treatment of derivative **1b**, which possesses a methoxy group in the position *para* to the pivaloylaminomethyl moiety, resulted in the formation of significantly more of the dimer-like compound **3b** as the major product and only a small amount of the aldehyde **2b**. When the reactions were carried out under the same conditions in tetrahydrofuran (THF) instead of DCM, substantially less dimer-like product **3a** or **3b** was formed in favor of the rearranged aldehyde **2a** or **2b**.

**Table 1:** Yields of rearranged aldehydes **2** and dimer-like products **3** and **8** in two different solvents.

starting material	yield of products in DCM				yield of products in THF			
	
	**2**	yield (%)		yield (%)	**2**	yield (%)		yield (%)

**1a**	**a**	51	**3a**	13	**a**	59	**3a**	4
**1b**	**b**	6	**3b**	43	**b**	35	**3b**	19
**1c**	**c**	traces	**8a**	8	**c**	traces	**8a**	7
**1d**	**d**	–	**8b**	10	**d**	–	**8b**	14

**Figure 1:**
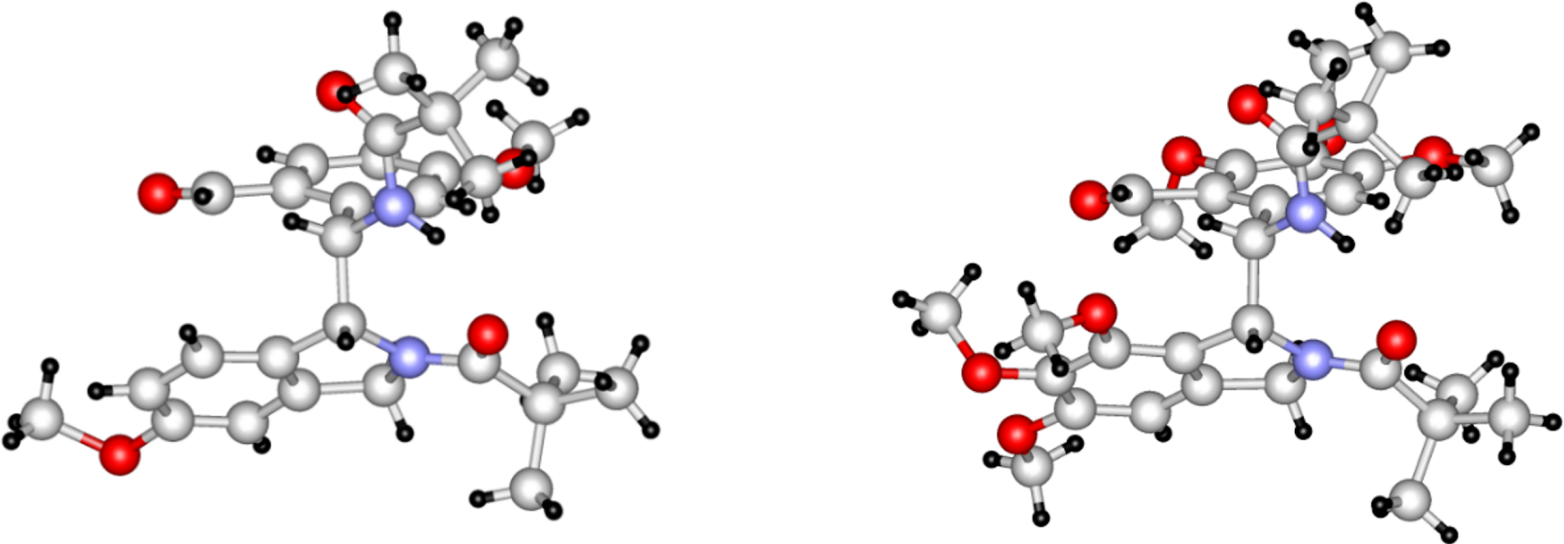
X-ray structures of compounds **3b** (left) and **8b** (right).

When starting from aldehyde **1c** [2], containing the methoxy function in the *ortho* position of the formyl moiety and *meta* to the pivaloylaminomethyl group, a practically full conversion of **1c** was achieved in both solvents. However, only traces of an isomeric aldehyde (most likely **2c**, based on analogy with **2a**,**b**) could be detected by LC–MS besides several dimer-like products. The major one (**8a**, Scheme 3) could be isolated in a pure form (Table 1). In the case of the trimethoxy derivative **1d** (prepared similarly to **1a**–**c**, see Experimental), ca. 90% conversion was achieved in both solvents and the formation of the isomeric aldehyde **2d** was not observed at all. As the only product, the dimer-like compound **8b** could be isolated and characterized. The structure determination of products **3b** and **8b** was also supported by single-crystal X-ray measurements. These compounds were obtained as racemates and Figure 1 shows those enantiomers in which the chiral center of the isoindoline moiety possesses an *R* configuration. All attempts to grow appropriate single crystals from derivative **8a** were unsuccessful.

It has to be emphasized that theoretically, the formation of four dimer-like regioisomers could be expected from aldehydes **1a**–**d**, each of them as the mixture of two diastereomeric racemates. Although HPLC–MS measurements indeed indicated the presence of isomeric dimers in the crude product mixtures, only the major isomers **3a**,**b**, and **8a**,**b** could be isolated.

The main structural difference between the dimer-like products **3a**,**b** and **8a**,**b** concerns the position of the linkage of the *ortho*-formylated *N*-pivaloylaminobenzyl moiety to the isoindoline building block. In the former products **3a**,**b** it is attached to the sterically less hindered, while in the latter compounds **8a**,**b** to the more hindered site of the isoindoline part.

### Proposed mechanism for the formation of rearranged aldehydes and dimer-like products

**Scheme 4:**
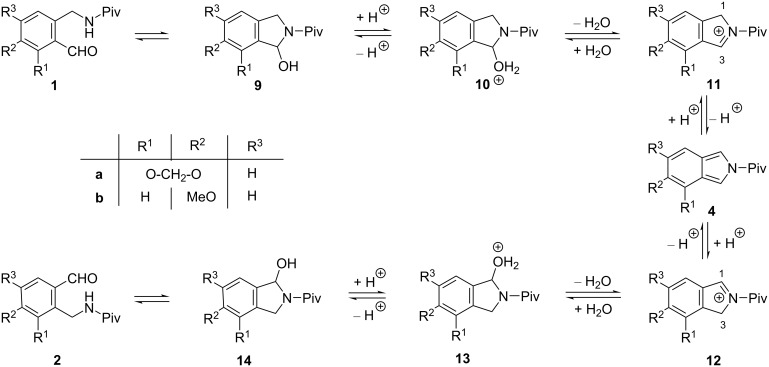
Proposed mechanism of the isomerization of aldehydes **1** via isoindoles **4**.

The formation of the rearranged aldehydes **2a**,**b** can be explained by the protonation of ring tautomers **9** to **10** (Scheme 4), followed by a water elimination, and subsequent deprotonation of cations **11** to afford isoindoles **4** [3–5]. The protonation of the latter [6], followed by the attack of water at position 1 of cations **12**, and subsequent deprotonation of intermediates **13**, results in hydroxyisoindolines **11**, which can finally tautomerize to aldehydes **2a**,**b**. In the previous report, we have demonstrated the existence of an equilibrium in the course of the transformation of **1a** to **2a** by trapping isoindole intermediate **4a** with *N*-phenylmaleimide as a dienophile in the acid-catalyzed reaction of both aldehydes **1a** and **2a** [1]. Moreover, as a reverse reaction, the formation of aldehyde **1a** was observed in the transformation of aldehyde **2a** under similar conditions [1]. However, this equilibrium is obviously influenced by the formation of the dimer-like products.

**Scheme 5:**
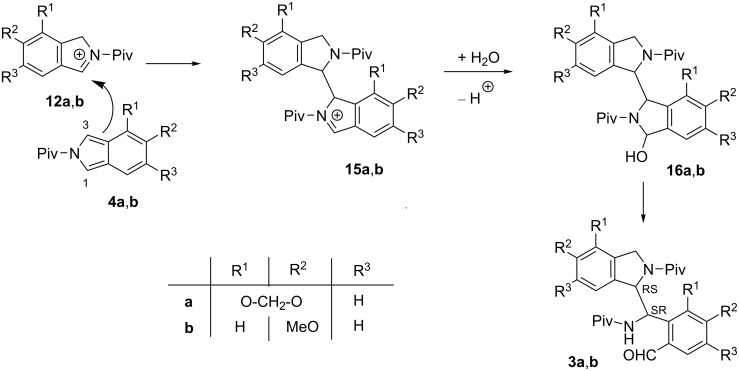
Proposed mechanism of the formation of dimer-like products **3a**,**b**.

The formation of the dimer-like products **3a** and **3b** can be rationalized by the electrophilic attack of cations **12a**,**b** at position 3 of isoindoles **4a**,**b** to give cations **15a**,**b** (Scheme 5). The reaction of the latter with water, followed by deprotonation leads to dimer-like compounds **16a**,**b**, being the ring tautomers of the isolated products **3a** and **3b**.

**Scheme 6:**
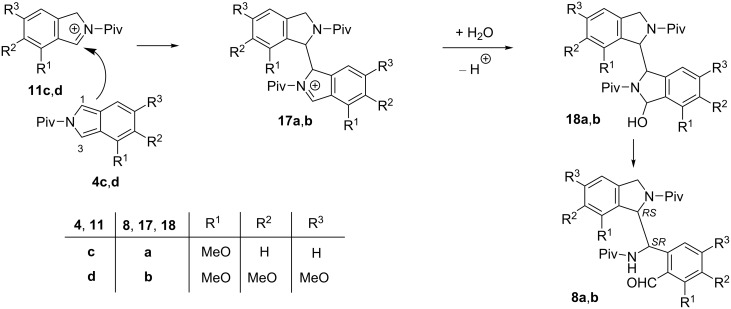
Proposed mechanism for the formation of dimer-like products **8a**,**b**.

On the contrary, the formation of dimer-like products **8a** and **8b** can be explained by the electrophilic attack of cations **11c**,**d** at position 1 (instead of position 3) of isoindoles **4c**,**d** to afford cations **17a**,**b** (Scheme 6), which transform to the dimer-like products **5a**,**b** via their ring tautomers **18a**,**b**.

**Scheme 7:**
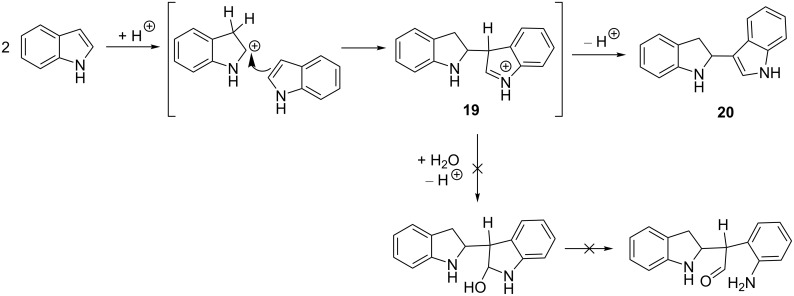
Dimerization of indole under acidic conditions.

The mechanism proposed for the formation of the intermediates **15a**,**b** and **17a**,**b** is supported by the fact that electrophilic substitution reactions of isoindole are well known in the literature [3,13–18] and these are analogous to that suggested for the dimerization of indole under acidic conditions (Scheme 7) [8]. Electrophilic attack of indole protonated at position 3 toward the position 3 of another indole molecule, followed by deprotonation affords dimer-like derivative **20**. The fundamental difference of this transformation from that we observed in the case of isoindoles is that the attack of water at cation **19** and subsequent ring opening did not happen here.

### DFT calculations

**Figure 2:**
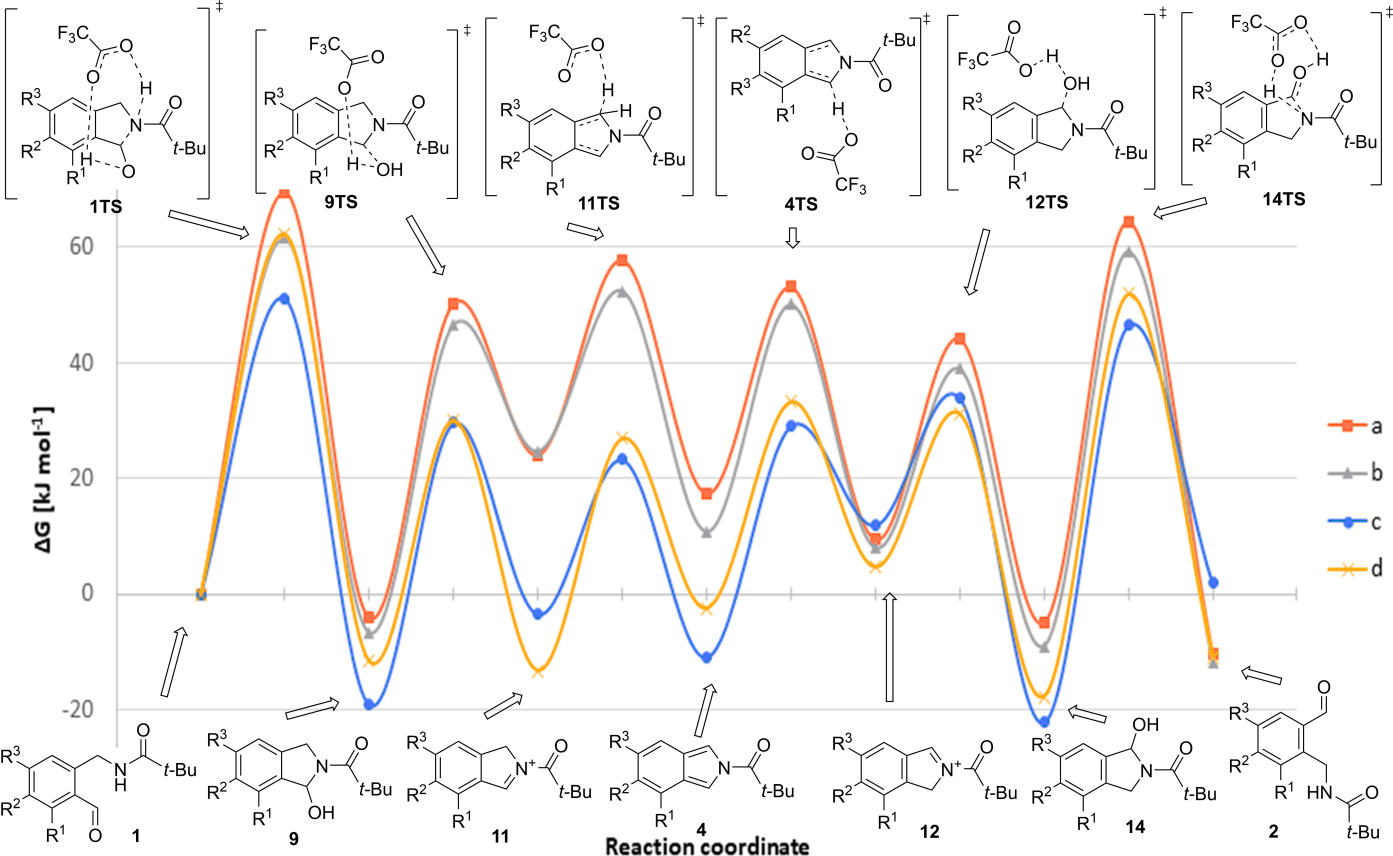
Gibbs free energy diagram for the **1**→**2** rearrangement.

The proposed mechanism of the **1**→**2** rearrangement (Scheme 4) was investigated in detail by DFT level quantum chemical computations using the Gaussian 09 program package [19] (Figure 2). Optimization and frequency calculation have been accomplished using the B3LYP/6-31+G (d,p) method [20–22], followed by single point energy calculation with the B3LYP/6-311++G (3df,3pd) method [23–24], considering the solvent effect of DCM with the IEFPCM implicit solvent model [25] in both cases. When inspecting the Gibbs-free energy diagram of the transformation of aldehydes **1a**–**c** (Figure 2, Table 2 and Table 3), it is noteworthy that the main trends until the formation of isoindole intermediates **4a**–**c** are very similar. However, there is a significant difference in the **4→12** transformations. While in the case of aldehydes **1a** and **1b** this step is exergonic, for aldehyde **1c** it is highly endergonic. This finding explains why, contrary to the formation of aldehydes **2a** and **2b**, product **2c** was practically not formed in the product mixture. Furthermore, it also explains the preference between the two regioisomeric cations **11** and **12** in the dimer-formation step. In the case of isoindoles **4a**,**b**, cations **12a**,**b** are more stable than cations **11a**,**b**, and the routes **11**→**4**→**12** leading to **12a**,**b** are exergonic. The simultaneous presence of isoindole **4a** and cation **12a**, or **4b** and **12b**, respectively, gives rise to the formation of dimer-like products **3a** or **3b** (Scheme 5). On the contrary, for isoindole **4c** the step to **12c** is endergonic (with Δ*G* = 23.5 kJ·mol^−1^) and regioisomer **11c** is energetically more advantageous, explaining the formation of the dimer of different type (**8a**, Scheme 6).

**Table 2:** Relative Gibbs free energy values corresponding to the transformations.

starting material	Δ*G* (kJ·mol^−1^)					
	
	**1**→**9**	**9**→**11**	**11**→**4**	**4**→**12**	**12**→**14**	**14**→**2**

**1a**	−4.2	28.2	−6.7	−9.7	−14.4	−9.4
**1b**	−6.8	31.5	−13.9	−2.8	−17.8	−2.9
**1c**	−19.1	15.6	−7.4	23.5	−34.1	24.1
**1d**	−11.7	−1.6	10.8	7.2	−22.4	6.9

**Table 3:** Relative Gibbs free energy values corresponding to the transition states.

starting material	Δ*G*^#^ (kJ·mol^−1^)					
	
	1TS	9TS	11TS	4TS	12TS	14TS

**1a**	69.5	54.4	33.7	25.6	34.7	65.2
**1b**	61.5	53.4	27.7	39.3	30.1	68.2
**1c**	51.2	48.7	26.7	40.0	22.0	68.7
**1d**	62.3	41.7	40.2	35.7	26.5	69.6

A closer look on the transformation of trimethoxy derivative **1d** reveals that dehydration is almost isoergonic (Δ*G***_9d_**_→_**_11d_** = −1.6 kJ·mol^−1^), but the formation of the isoindole intermediate (**11d**→**4d**) is endergonic (unlike in the case of the three other derivatives), with a larger activation free energy (Δ*G*^#^**_11TS_** = 40.2 kJ·mol^−1^) than those of congeners **a**–**c**. Moreover, there are two further endergonic steps in this sequence (**4d**→**12d**, **14d**→**2d**), which data altogether explain why product **2d** was not detected at all and why cation **11d** is preferred in the dimer formation, leading to product **8b**.

According to the calculations it turned out that the acid catalyst (TFA in this case), as expected, had a crucial role in the protonation and deprotonation as well as in the dehydration and hydration elementary steps of the reaction sequence. Without catalyst, the **9a**→**11a**→**4a** transformations required an energy investment of 150.7 and 163.3 kJ·mol^−1^, instead of 54.4 kJ·mol^−1^ and 33.7 kJ·mol^−1^, calculated with TFA (Table 3).

In conclusion, the DFT calculations are consistent with the observation that the formation of the rearranged aldehydes **2a**,**b** is more favorable than that of **2c**,**d**, and explain the difference observed between the regiochemistry of dimers **3a**,**b** and **8a**,**b**.

### Acid-catalyzed transformation of unsubstituted *ortho*-(pivaloylaminomethyl)benzaldehyde (**1e**)

In order to reduce the number of possible dimer-like products, we carried out the reaction with unsubstituted *o*-(pivaloylaminomethyl)benzaldehyde (**1e**). Since the positional change of the two functional groups cannot be observed in this case, we could focus on the formation of the dimer-like products.

**Scheme 8:**
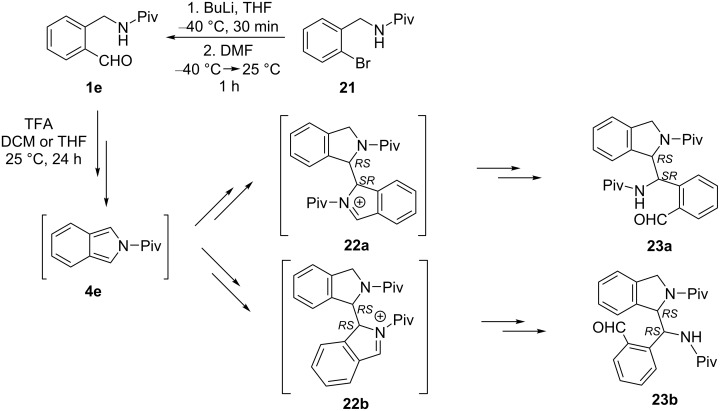
Reaction of *o*-(pivaloylaminomethyl)benzaldehyde (**1e**) to give dimer-like products **23a** and **23b**.

**Figure 3:**
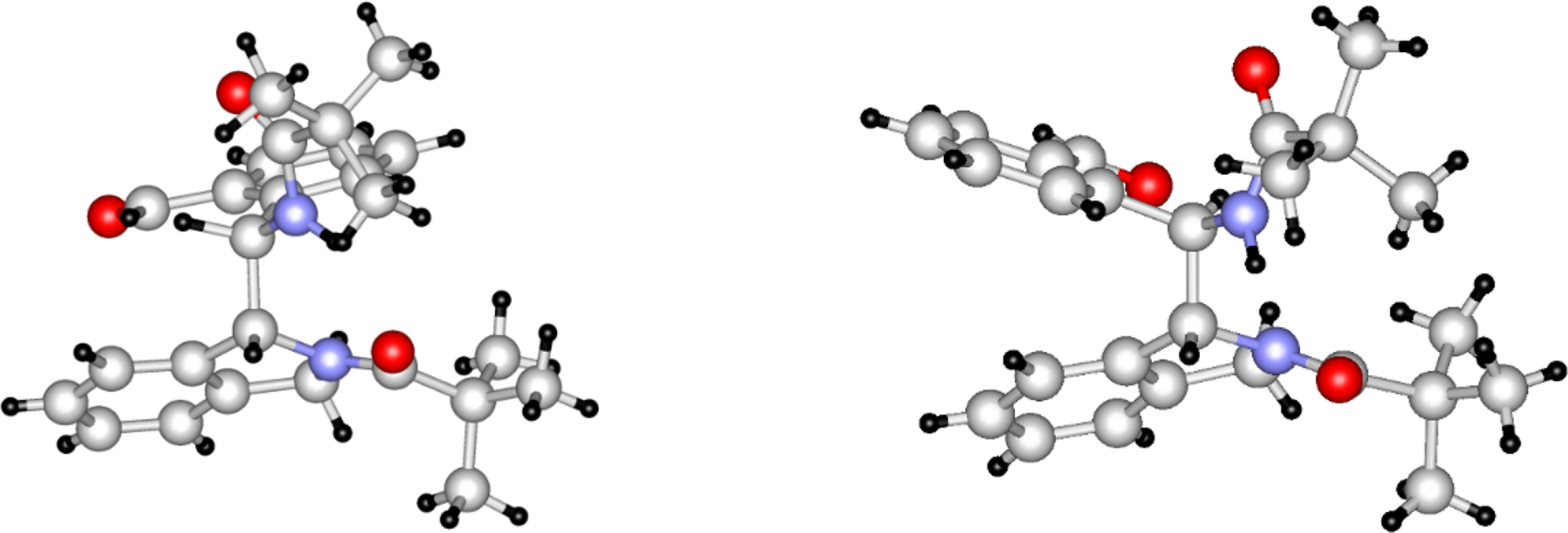
X-ray structures of compounds **23a** (left) and **23b** (right).

Treatment of unsubstituted derivative **1e** (prepared from bromo derivative **21** [26] by formylation via lithiation) in DCM in the presence of a catalytic amount (0.1 equiv) of TFA for 24 h at room temperature led to dimer-like product **23a** in 58% yield, obviously via the intermediate cation **22a** (Scheme 8). The structure of **23a** was supported by NMR data and also by single-crystal X-ray measurement (Figure 3). Nevertheless, HPLC–MS analysis of the crude product mixture showed, in addition to **23a** (77%), the presence of an isomeric dimer-like product (**23b**, 10%) and unreacted starting material (8%). Due to the similar chromatographic behavior of **23b** and **23a** on normal phase, they were isolated from the crude product by reversed-phase preparative HPLC, then identified by detailed NMR experiments, and single-crystal X-ray measurement (Figure 3) as diastereomer racemates. When the reaction of **1e** was carried out under the same conditions in THF instead of DCM, HPLC–MS analysis of the crude product mixture showed the presence of dimer-like product **23a** (25%), the recovered starting material **1e** (67%), and traces (ca. 1%) of the isomeric dimer-like product (**23b**). After work-up, 18% of compound **23a** was obtained in addition to 51% of **1e**.

As shown in the structures, the chiral center of the isoindoline moiety in the enantiomer of compound **23a** shown in Figure 3 is *R*, while the other chiral center exhibits an *S* configuration, so its racemic form is hereinafter referred to as *RS*–*SR*. By the same logic, another designation for racemic **23b** is *RR*–*SS*. The formation of intermediates **22a** and **22b** (Scheme 8), as the rate-determining step leading to major product **23a** (*RS*–*SR*) and minor product **23b** (*RR*–*SS*), respectively, was also investigated by DFT level quantum chemical calculations with the same parameters as in the case of the **1**→**2** rearrangement. The racemic *RS* and *RR* forms were taken into consideration, and the computations showed that the formation of the *RS* compound required an energy investment of Δ*G*^#^**_4e_**_→_**_22a_** = 89.1 kJ·mol^−1^, while the reaction leading to the minor (*RR*) isomer had a larger activation barrier (Δ*G*^#^**_4e_**_→_**_22b_** = 101.9 kJ·mol^−1^). Moreover, the formation of the latter intermediate was significantly more endergonic (Δ*G***_4e_**_→_**_22b_** = 69.2 kJ·mol^−1^) than Δ*G***_4e_**_→_**_22a_** (37.9 kJ·mol^−1^).

The structure elucidation of compounds **3a** [1], **3b**, **8a**,**b**, and **23a**,**b** was based on the molecular formula obtained from HRMS, comprehensive ^1^H and ^13^C NMR study and on the single-crystal X-ray structure of compounds **3a** [1], **3b**, **8b**, **23a**, and **23b** (for spectra and detailed interpretation, see Supporting Information File 1). According to the single-crystal X-ray experiments of compounds **3b**, **8b**, and **23a**, the solid-phase structures are stabilized by a hydrogen bond between the amide NH and the carbonyl oxygen atom of the other amide group. The H–C–C–H dihedral angles between the two asymmetric centers are not far from 90° as shown by the X-ray structures of these compounds (Figure 1 and Figure 3), and also reflected by their low ^3^*J*(H,H) coupling constants (ca. 1 Hz) detected in CDCl_3_ solution. In the ^1^H NMR spectra of compounds **3b**, **8a**,**b**, and **23a**, the δΝΗ chemical shifts appeared in a narrow range between 9.39–9.06 ppm, indicating the presence of strong hydrogen bonds also in solution. These data suggest that the structures of the predominating conformers should be similar in the solid and liquid phases. Furthermore, we have managed to detect NOE steric proximities between the aldehyde H atom and the *H*-C(7) (in compounds **3b**, **23a**) or C*H*_3_O-C(7) hydrogens (in compounds **8a**,**b**) of the isoindoline moiety. Supposing an *R* configuration at the chiral center of the isoindoline unit, this kind of steric arrangement is only possible, if the other asymmetric center exists in an *S* configuration.

As regards the minor dimer-like product **23b**, the high-resolution mass spectrum proposed exactly the same molecular formula as for **23a**. The ^1^H and ^13^C spectra taken in CDCl_3_ solution also exhibited several similar chemical shifts to those obtained for **23a**, but some interesting differences were also detected. The δNH = 8.50 ppm chemical shift in **23b** indicates a weaker hydrogen bond, compared to the value of 9.39 ppm obtained for **23a** [27]. The detected ^3^*J*(H,H) coupling constant for the hydrogens attached to the two asymmetric centers is 10 Hz in this case, indicating a dihedral angle of ca. 180° in **23b**. Moreover, an unexpected extremely upfield aromatic δH(7) value (5.71 ppm) was measured for the isoindoline moiety of this compound, i.e., a diamagnetic shift of ca. 1.8 ppm occurred in this position. In order to explain this phenomenon, we should consider the anisotropic effect of the aromatic rings. Such steric arrangement is only possible in case of a *RR*–*SS* diastereomer (**23b**), with only one of the aromatic hydrogen atoms of the isoindoline moiety [H(7)] located in close proximity above the middle of the other aromatic ring. It can be concluded after all that the NMR data also made it possible to assign the *RS*–*SR* diastereomeric structure for compounds **3b**, **8a**,**b**, and **23a**, and the *RR*–*SS* structure for congener **23b**.

**Figure 4:**
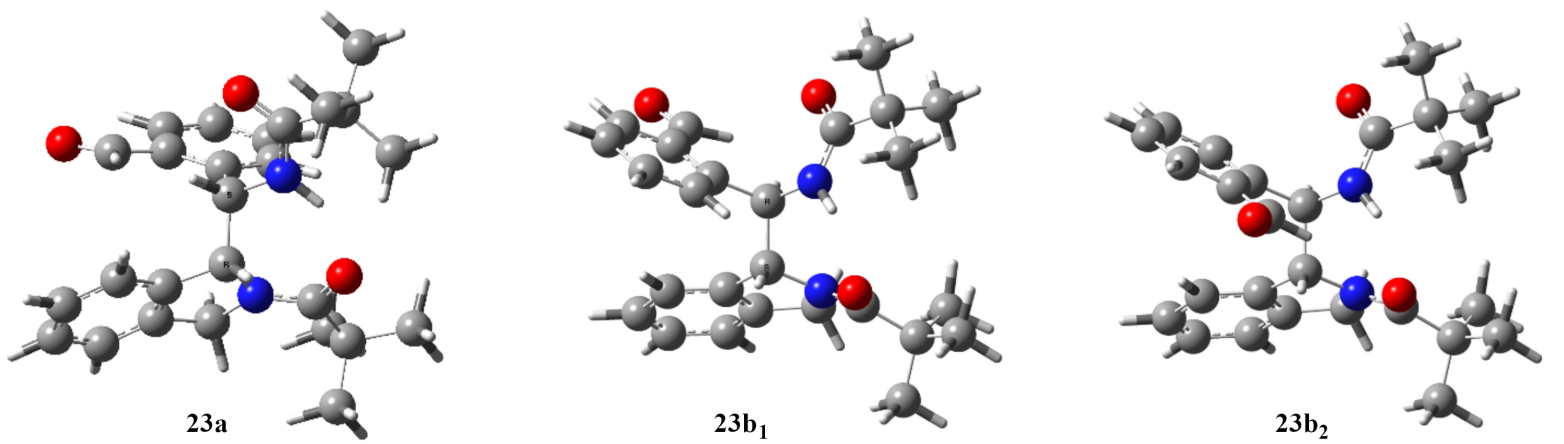
Structures of the minimal energy conformer of stereoisomer **23a** and those of two minimal energy conformers of **23b** (**23b_1_** and **23b_2_**) in DCM solution, based on DFT calculations.

In order to get a better insight into the stereochemistry of compounds **23a** and **23b**, we calculated their refined stereostructure by means of DFT geometry optimization in a DCM solution. As a result, one minimal energy conformer was identified for **23a** and two for **23b** (Figure 4). In the two preferred conformers of **23b** (**23b_1_** and **23b_2_**), the plane of the *ortho*-formyl-substituted phenyl rings is twisted with 180° compared to each other. The Gibbs free energy difference between the two conformers is 4.1 kJ·mol^−1^ suggesting the possibility of a rotation of the *ortho*-formylphenyl group. Notably, conformer **23b_1_** is the more stable one, corresponding to the structure determined by solid-phase X-ray measurements (Figure 3, right). It should be mentioned that a few signals in the NMR spectra of **23b**, e.g., δ*H*C−N (5.71 dd), δH*C*= (131.1), and δ*C*= (142.5) showed a considerable line-broadening (coalescence), revealing a partially hindered rotation of the formylphenyl moiety, i.e., the occurrence of a conformational equilibrium has to be taken into account. Even the δH*C*−N ^13^C NMR signal remained under the noise level, indicating that the interconversion of these conformers is slow in the NMR time-scale.

Although the NMR spectra have been measured in CDCl_3_, we are convinced that the conformations observed in CDCl_3_ are in good accordance with the ones computed considering the implicit solvent effect of DCM. Based on the structural similarity, the close dielectric constant (ε = 4.7 for CHCl_3_ and 8.9 for DCM) [28], the similar polarity index (*P* = 2.7 for CHCl_3_ and 3.1 for DCM) [29–31], and the very close dipole moment (1.15 for CHCl_3_, 1.14 for DCM) [32], the conformation of the compounds should be almost the same in the NMR solvent and in the DCM reaction medium. This is further supported by the recent results of Allen et al., who have shown that CHCl_3_ and DCM molecules exhibited the same interactions also in crystal packing [33].

## Conclusion

When alkoxy-substituted *o*-(pivaloylaminomethyl)benzaldehydes **1a**,**b** were kept in solution (DCM or THF) in the presence of a catalytic amount of TFA at room temperature, the formation of regioisomeric aldehydes **2a**,**b** and dimer-like products **3a**,**b** was observed. After a similar treatment of related benzaldehydes **1c**,**d**, only the dimer-like products **8a**,**b** were isolated. The transformations occur via an isoindole intermediate **4**. In order to reduce the number of expected products, the similar reaction of unsubstituted *o*-(pivaloylaminomethyl)benzaldehyde (**1e**) was also studied*.* DFT calculations supported the mechanism proposed for the transformations and explained the structural differences of the dimer-like products, as well as the observed product distributions.

## Experimental

Compounds **1a**,**b**,**c** [2], **2a**, **3a** [1] and **21** [26] are known in the literature, while compounds **1d**,**e**, **2b**, **3b**, **8a**,**b** and **23a**,**b** are new. The synthetic procedures and characterizations are given either below (**2a** and **3a** with new synthetic procedures, and **8a**, **23a**) or in Supporting Information File 1 (**1d**, **1e**, **2b**, **3b**, **8b**, **23b**). All melting points were determined on a Büchi B-540 (Flawil, Switzerland) capillary melting point apparatus and are uncorrected. IR spectra were obtained on a Bruker ALPHA FT-IR spectrometer (Billerica, MA, USA) in KBr pellets. ^1^H NMR, ^13^C NMR, DeptQ, Dept-135, edHSQC, selective HSQC, HMBC, selective HMBC, NOESY, ^1^H,^1^H-COSY, one-dimensional selective NOE, and selective TOCSY spectra were recorded at 295 K on a Bruker Avance III HD 600 (Billerica, MA, USA; 600 and 150 MHz for ^1^H and ^13^C NMR spectra, respectively) spectrometer equipped with a Prodigy cryo-probehead. The pulse programs were taken from the Bruker software library (TopSpin 3.5) and full ^1^H and ^13^C assignments were achieved with widely accepted strategies [34–35]. ^1^H NMR assignments were accomplished using general knowledge of chemical shift dispersion with the aid of the ^1^H-^1^H coupling pattern (^1^H NMR spectra). CDCl_3_ was used as the solvent and tetramethylsilane (TMS) as the internal standard. Chemical shifts (δ) and coupling constants (*J*) are given in ppm and Hz, respectively. The lists of the NMR signals can be found in Supporting Information File 1. To facilitate the understanding of the ^1^H and ^13^C signal assignments, the structures of the compounds are also depicted on the spectra (Supporting Information File 1 pages S3–S43). High-resolution mass spectra were recorded on a Bruker O-TOF MAXIS Impact mass spectrometer (Billerica, MA, USA) coupled with a Dionex Ultimate 3000 RS HPLC (Sunnyvale, CA, USA) system with a diode array detector. HRMS spectra of the diastereomeric **23a** and **23b** compounds are shown on page S44 of Supporting Information File 1. Single-crystal X-ray diffraction (SC-XRD) measurements were carried out on a Rigaku R-Axis Spider diffractometer (Tokyo, Japan) with imaging plate area detector using graphite monochromatic Cu Kα radiation. SC-XRD structures were deposited at the Cambridge Crystallographic Data Centre under the following numbers: CCDC 1953421 (**3a**), 1953424 (**3b**), 1953422 (**8b**), 1953423 (**23a**) and 1958809 (**23b**). All reagents were purchased from commercial sources and used without further purification. The reactions were followed by analytical thin-layer chromatography on silica gel 60 F_254_ (Darmstadt, Germany) and on a Shimadzu LC-20 HPLC equipment coupled with an LCMS-2020 mass spectrometer (Kyoto, Japan). Purifications by flash chromatography were performed applying a Teledyne Isco Combiflash^®^ Rf system (Thousand Oaks, CA, USA) with Redisep^®^ Rf silica flash columns using a hexane–EtOAc solvent system. The preparative HPLC separation was carried out using a Merck LaPrep Sigma LP1200 pump and a Merck LaPrep P314 UV detector, under the conditions described below for compound **23b**.

The DFT level computations at the B3LYP/6-31+G (d,p) level of theory were performed considering the solvent effect of DCM using the IEFPCM solvent model with the Gaussian 09 program package. The geometries of the molecules were optimized in all cases, and frequency calculations were also performed to assure that the structures are in a local minimum or in a saddle point. This was followed by single point measurements at the B3LYP/6-311++G (3df,3pd) level of theory that resulted in the energy values presented in Table S1 (Supporting Information File 1, page S45) and used for the figures of the paper. The conformations of the reported structures have been determined by conformational analysis. The solution-phase Gibbs free energies were obtained by frequency calculations as well. The *G* values obtained were given under standard conditions, the corrected total energies of the molecules were taken into account. Entropic and thermal corrections were evaluated for isolated molecules using standard rigid rotor harmonic oscillator approximations. That is, the Gibbs free energy was taken as the “sum of electronic and thermal free energies” printed in a Gaussian 09 vibrational frequency calculation. Standard state correction was taken into account. The transition states were optimized with the QST3 or the TS (berny) method. Transition states were identified by having one imaginary frequency in the Hessian matrix, and IRC calculations were performed in order to prove that the transition states connect two corresponding minima.

**General procedure I for the synthesis of compounds 2a,b, 3a,b, 8a,b**, **and 23a.** TFA (0.1 equiv) was added to a solution of **1a**–**e** in DCM (20 mL). After stirring for 24 h at room temperature, an aqueous sodium carbonate solution (5%, 7 mL) and DCM (4 mL) were added. The aqueous layer was extracted with DCM (2 × 4 mL). The combined organic layer was dried over MgSO_4_. The solvent was evaporated and the residue purified by flash chromatography (5**–**30% EtOAc in hexane). The corresponding fractions were collected, evaporated, and recrystallized from EtOAc/hexane to afford the title compounds as white solids.

**General procedure II for the synthesis of compounds 2a,b, 3a,b, 8a,b and 23a.** TFA (0.1 equiv) was added to a solution of **1a**–**e** in THF (20 mL). After stirring for 24 h at room temperature, an aqueous sodium carbonate solution (5%, 7 mL) and DCM (4 mL) were added. The aqueous layer was extracted with DCM (2 × 4 mL). The combined organic layer was dried over MgSO_4_. The solvent was evaporated and the residue purified by flash chromatography (5–30% EtOAc in hexane). The corresponding fractions were collected, evaporated, and recrystallized from EtOAc/hexane to afford the title compounds as white solids.

***N*-[(5-Formyl-2*H*-1,3-benzodioxol-4-yl)methyl]-2,2-dimethylpropanamide (2a)** [1]. Method A: This compound was prepared according to general procedure I using **1a** [2] (1.02 g, 3.88 mmol) and TFA (30 µL, 44 mg, 0.39 mmol). The title compound (517 mg, 51%) was isolated as white solid. Mp 125**–**126 °C (EtOAc/hexane); IR (KBr): νNH 3344, νCH 3082, νHC=O 1682, νC=O (amide) 1641, νC=C (Ar) 1596, 1494, ν_as_C−O−C 1268, *ν_s_*C−O−C 1064 cm^−1^; HRMS (*m*/*z*): [M + H]^+^ calcd for C_14_H_18_NO_4_^+^ 264.1230; found: 264.1229; anal. calcd for C_14_H_17_NO_4_ (263.29): N, 5.32; H, 6.51; C, 63.87; found: N, 5.38; H, 6.44; C, 63.77%; NMR data are identical with those published earlier [1].

Method B: This compound was prepared according to general procedure II using **1a** [2] (1.00 g, 3.80 mmol) and TFA (29 µL, 43 mg, 0.38 mmol). The title compound (592 mg, 59%) was isolated as white solid. Analytical data are identical with those described in Method A.

***N*-[(*S,R*)-[(6*R,S*)-7-(2,2-Dimethylpropanoyl)-2*H*,6*H*,7*H*,8*H*-[1,3]dioxolo[4,5-*e*]isoindol-6-yl](5-formyl-2*H*-1,3-benzodioxol-4-yl)methyl]-2,2-dimethylpropanamide (3a)** [1], Method A: This compound was prepared according to general procedure I using **1a** [2] (1.02 g, 3.88 mmol) and TFA (30 µL, 44 mg, 0.39 mmol). The title compound (135 mg, 13%) was isolated as a white solid. Mp 248–250 °C (EtOAc/hexane); IR (KBr): νNH 3336, νCH 3088, νHC=O 1680, νC=O (amide) 1659, νC=C (Ar) 1614, 1474, ν_as_C−O−C 1257, *ν_s_*C−O−C 1052 cm^−1^; HRMS (*m*/*z*): [M + H]^+^ calcd for C_28_H_33_N_2_O_7_^+^ 509.2283; found: 509.2284; anal. calcd for C_28_H_32_N_2_O_7_ (508.57): N, 5.51; H, 6.34; C, 66.13%; found: N, 5.52; H, 6.13; C, 65.90%. NMR data are identical with those published earlier [1].

Method B: This compound was prepared according to general procedure II using **1a** [2] (1.00 g, 3.80 mmol) and TFA (29 µL, 43 mg, 0.38 mmol). The title compound (42 mg, 4%) was isolated as a white solid. Analytical data are identical with those described in Method A.

***N*-[(*S,R*)-[(1*R,S*)-2**-**(2,2-Dimethylpropanoyl)-7-methoxy-2,3-dihydro-1*H*-isoindol-1-yl](2-formyl-3-methoxyphenyl)methyl}-2,2-dimethylpropanamide (8a).** Method A: This compound was prepared according to general procedure I using **1c** [2] (1.01 g, 4.06 mmol) and TFA (31 µL, 46 mg, 0.41 mmol). The title compound (78 mg, 8%) was isolated as white solid. Mp 169−171 °C (EtOAc/hexane); IR (KBr): νNH 3291, νCH 3027, νHC=O 1695, νC=O (amide) 1663, νC=C (Ar) 1612, 1527, ν_as_C−O−C 1276, *ν_s_*C−O−C 1074 cm^−1^; HRMS (*m*/*z*): [M+H]^+^ calcd for C_28_H_37_N_2_O_5_^+^ 481.2697; found: 481.2681; anal. calcd for C_28_H_36_N_2_O_5_ (480.61): N, 5.83; H, 7.55; C, 69.98%; found: N, 5.87; H, 7.36; C, 69.83%. ^1^H and ^13^C spectra are shown on page S19 of Supporting Information File 1. Hydrogen–hydrogen connectivities were elucidated utilizing the ^1^H,^1^H-COSY experiment (page S20, Supporting Information File 1). The detected low ^3^*J*(H,H) coupling constant (ca. 1 Hz) revealed also in this case a dihedral angle of nearly 90° for the H–C–C–H moiety. One-dimensional sel-NOE experiments on signals 9.78, 6.88, 6.30, 5.91, 4.59, and 3.42 elucidated the characteristic steric proximities (pages S21 and S22 in Supporting Information File 1) and proved the *SR*–*RS*-type structure. The ^13^C signal assignment was supported with HSQC and HMBC spectra (pages S23 and S24 in Supporting Information File 1).

Method B: This compound was prepared according to general procedure II using **1c** [2] (1.00 g, 4.02 mmol) and TFA (31 µL, 46 mg, 0.40 mmol). The title compound (71 mg, 7%) was isolated as white solid. Analytical data are identical with those described in Method A.

***N*-[(*S,R*)-[(1*R,S*)-2-(2,2-Dimethylpropanoyl)-2,3-dihydro-1*H*-isoindol-1-yl](2-formylphenyl)methyl]-2,2-dimethylpropanamide (23a).** Method A: This compound was prepared according to general procedure I using **1e** (1.01 g, 4.61 mmol) and trifluoroacetic acid (35 µL, 53 mg, 0.46 mmol). The title compound (584 mg, 58%) was isolated as white solid. Mp 156−158 °C (EtOAc/hexane); IR (KBr): νNH 3314, νCH 2965, νHC=O 1693, νC=O (amide) 1657, νC=C (Ar) 1613, 1481 cm^−1^; HRMS [M + H]^+^ calcd for C_26_H_33_N_2_O_3_^+^ 421.2491; found: 421.2484; anal. calcd for C_26_H_32_N_2_O_3_ (420.55): N, 6.66; H, 7.67; C, 74.26%; found: N, 6.70; H, 7.42; C, 73.98%. The ^1^H spectrum is shown on page S32 of Supporting Information File 1. The two-dimensional NOESY experiment (page S33 in Supporting Information File 1) revealed the characteristic steric proximities and proved the *SR*–*RS*-type structure. The ^13^C signal assignment (page S34 in Supporting Information File 1) was supported by edHSQC and HMBC measurements, together with their band selective versions (pages S35–S37 in Supporting Information File 1).

Method B: This compound was prepared according to general procedure II using **1e** (1.03 g, 4.70 mmol) and TFA (36 µL, 54 mg, 0.47 mmol). The title compound (182 mg, 18%) was isolated as white solid. Analytical data are identical with those described in Method A.

## Supporting Information

Detailed NMR studies (^1^H NMR, ^13^C NMR, DeptQ, Dept-135, edHSQC, selective HSQC, HMBC, selective HMBC, NOESY, ^1^H,^1^H-COSY, one-dimensional selective NOE, selective TOCSY spectra) of new compounds; HRMS spectra of compounds **23a** and **23b**; coordinates and energy values of the computed structures; cif files and structure report files of compounds **3a**, **3b**, **8b**, **23a**, **23b**.

File 1 Detailed NMR studies.

File 2 Crystallographic information files for compounds **3a**, **3b**, **8b**, **23a**, and **23b**.
