# Supplementary material for: Rearrangement of o-(pivaloylaminomethyl)benzaldehydes: an experimental and computational study
Source: Beilstein J Org Chem. 2020 Jul 13;16:1636–48. doi: 10.3762/bjoc.16.136 (PMC7372232; doi:10.3762/bjoc.16.136)
Supplement: File 1 — Detailed NMR studies. [file Beilstein_J_Org_Chem-16-1636-s001.pdf]

BEILSTEIN JOURNAL OF ORGANIC CHEMISTRY

Supporting Information

for

Rearrangement of *o*-(pivaloylaminomethyl)benzaldehydes: an experimental and computational study

Csilla Hargitai, Györgyi Koványi-Lax, Tamás Nagy, Péter Ábrányi-Balogh, András Dancsó, Gábor Tóth, Judit Halász, Angéla Pandur, Gyula Simig and Balázs Volk

Beilstein J. Org. Chem. **2020,** *16,* 1636–1648. doi:10.3762/bjoc.16.136

Detailed NMR studies

License and Terms: This is a supporting information file under the terms of the Creative Commons Attribution License (http://creativecommons.org/licenses/by/4.0). Please note that the reuse, redistribution and reproduction in particular requires that the authors and source are credited. The license is subject to the *Beilstein Journal of Organic Chemistry* terms and conditions: (https://www.beilstein-journals.org/bjoc)

Table of contents

Compound **1d**, ^{1}H and DeptQ (^{13}C) NMR S3

Compound **1d**, edHSQC S4

Compound **1d**, HMBC S5

Compound **1e**, ^{1}H and ^{13}C NMR S6

Compound **1e**, edHSQC and HMBC S7

Compound **2b**, ^{1}H NMR with sel-NOE S8

Compound **2b**, ^{13}C and Dept-135 NMR S9

Compound **2b**, HSQC and HMBC S10

Compound **3b**, ^{1}H NMR S11

Compound **3b**, Steric proximities detected by sel-NOE S12

Compound **3b**, Steric proximities detected by sel-NOE S13

Compound **3b**, $^{1}H,^{1}H$-COSY S14

Compound **3b**, ^{13}C + Dept-135 .... S15

Compound **3b**, HSQC + selective HSQC S16

Compound **3b**, HMBC S17

Compound **3b**, HMBC section S18

Compound **8a**, ^{1}H and ^{13}C NMR S19

Compound **8a**, $^{1}H,^{1}H$-COSY S20

Compound **8a**, Steric proximities detected by sel-NOE S21

Compound **8a**, Steric proximities detected by sel-NOE S22

Compound **8a**, HSQC S23

Compound **8a**, HMBC S24

Compound **8b**, ^{1}H and $^{1}H,^{1}H$-COSY S25

Compound **8b**, Steric proximities detected by sel-NOE S26

Compound **8b**, Steric proximities detected by sel-NOE S27

Compound **8b**, Steric proximities detected by NOESY S28

Compound **8b**, ^{13}C NMR S29

Compound **8b**, HSQC S30

Compound **8b**, HMBC S31

Compound **23a**, ^{1}H NMR S32

Compound **23a**, Steric proximities detected by NOESY S33
Compound **23a**, ^{13}C NMR S34
Compound **23a**, edHSQC S35
Compound **23a**, HMBC S36
Compound **23a**, selective HSQC and selective HMBC S37
Compound **23b**, ^{1}H and DeptQ NMR S38
Compound **23b**, Identification of the three ^{1}H spin-systems by sel. TOCSY S39
Compound **23b**, Steric proximities detected by NOESY S40
Compound **23b**, Steric proximities detected by sel-NOE S41
Compound **23b**, edHSQC S42
Compound **23b**, HMBC S43
Compounds **23a** and **23b**, HRMS S44
DFT calculations S45
  Energy values obtained for the computation of the **1**→**2** transformation and the related dimerization S45
  Negative frequencies of the computed transition states S46
  Coordinates of the computed structures S46
Synthesis and further characterization of compounds **1d**, **1e**, **2b**, **3b**, **8b** and **23b** S67

Compound **1d**, ^{1}H and DeptQ (^{13}C) NMR spectra (600/150 MHz)

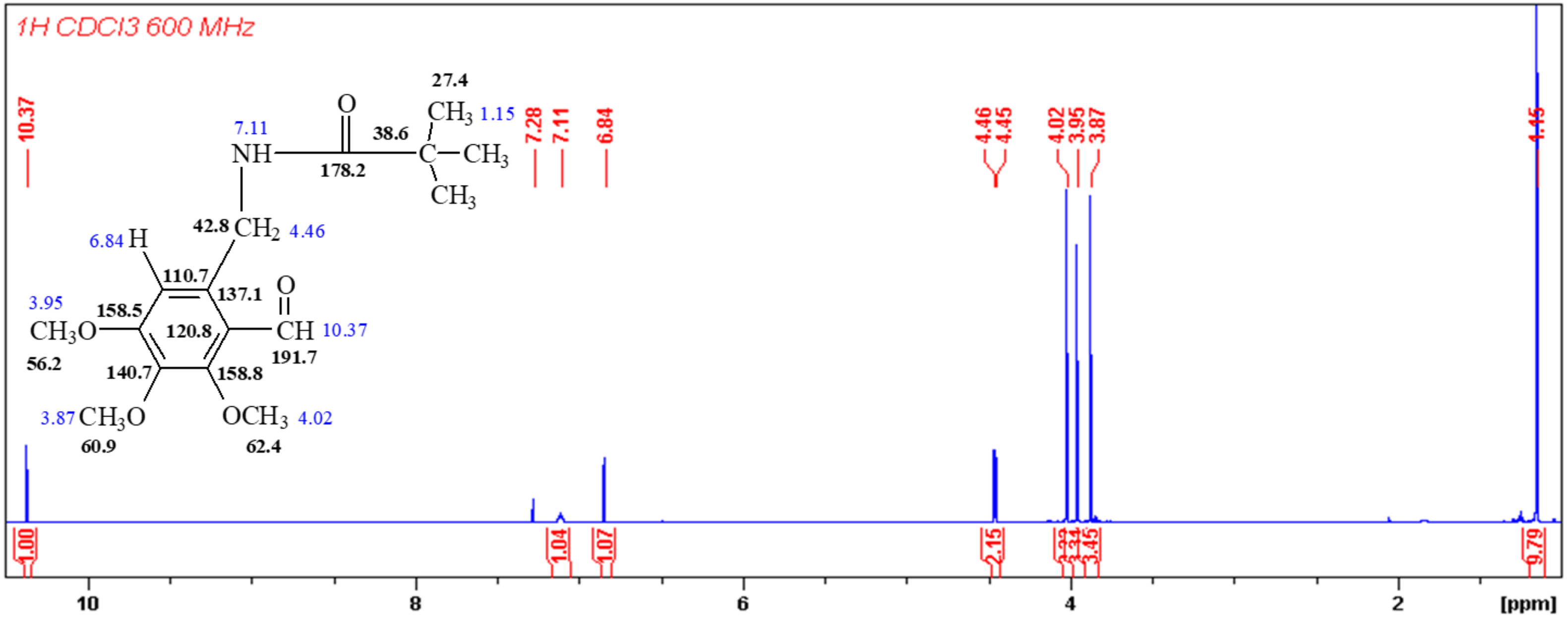

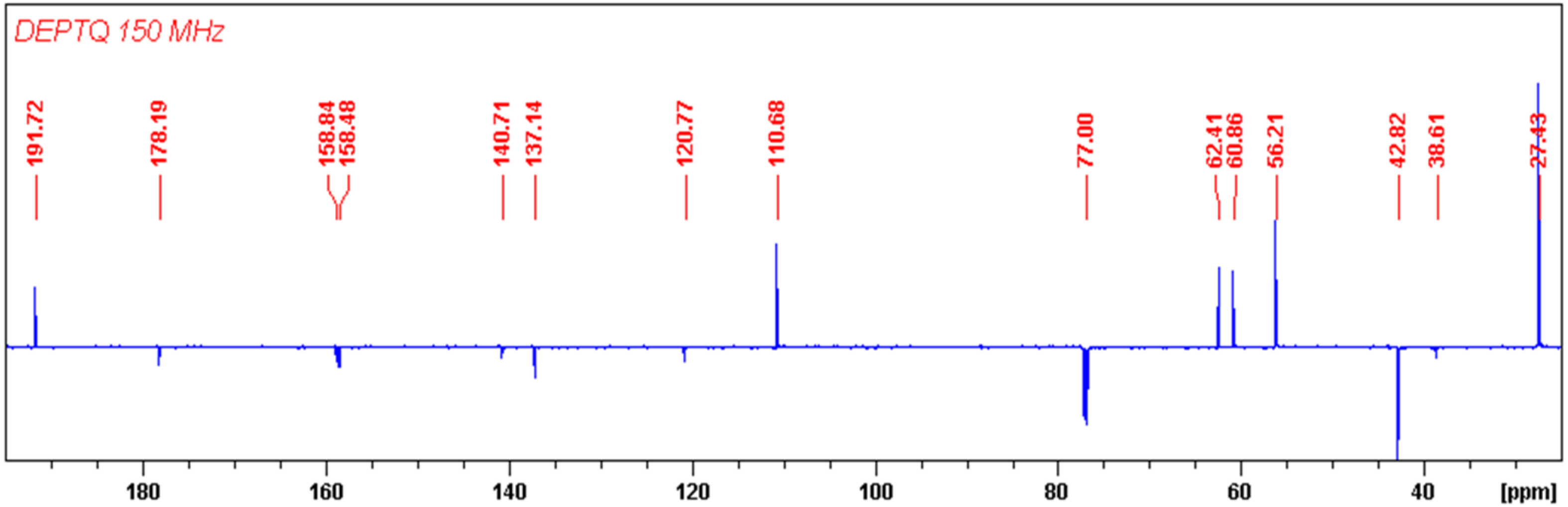

Compound **1d**, edHSQC

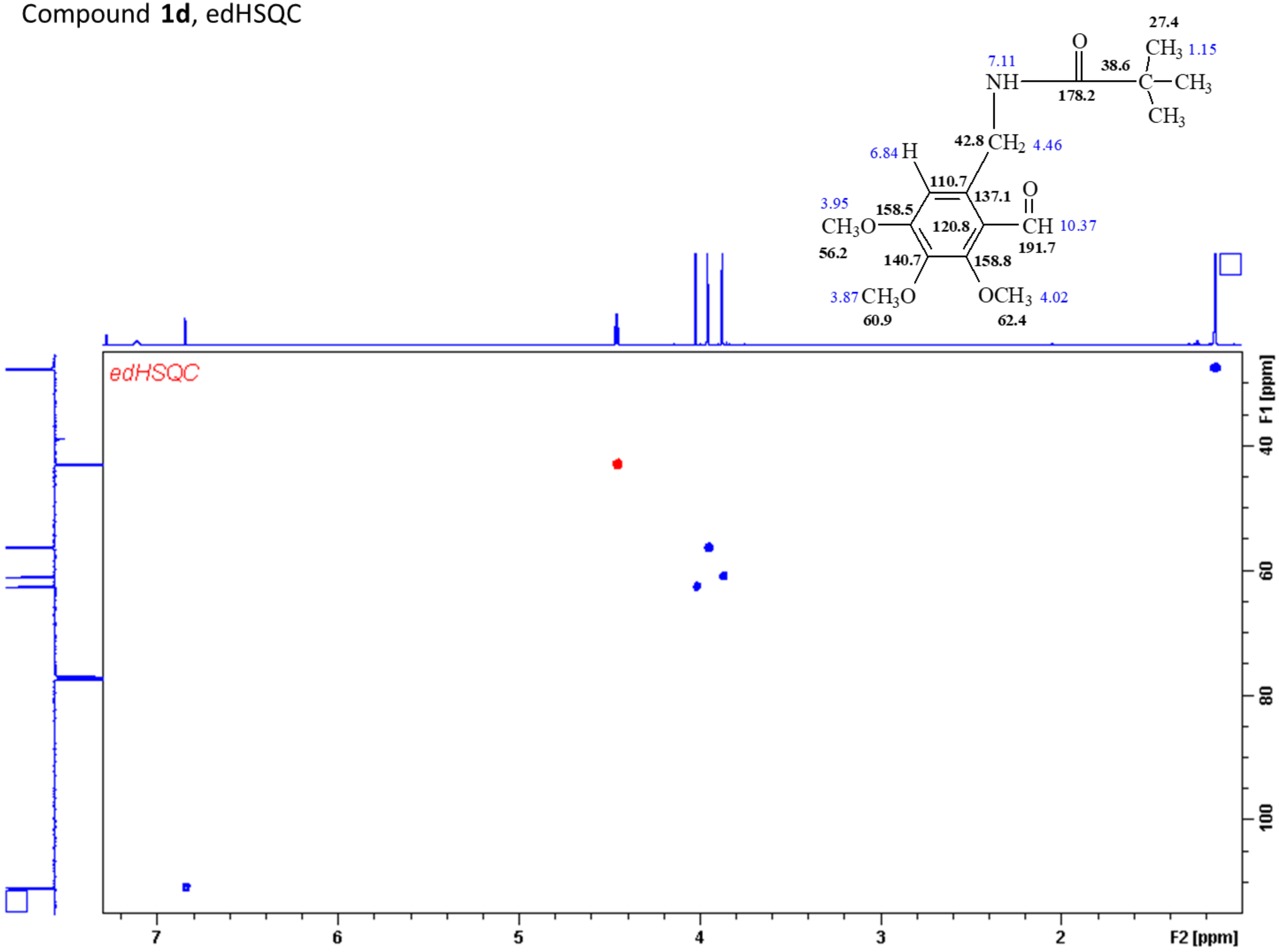

Compound **1d**, HMBC

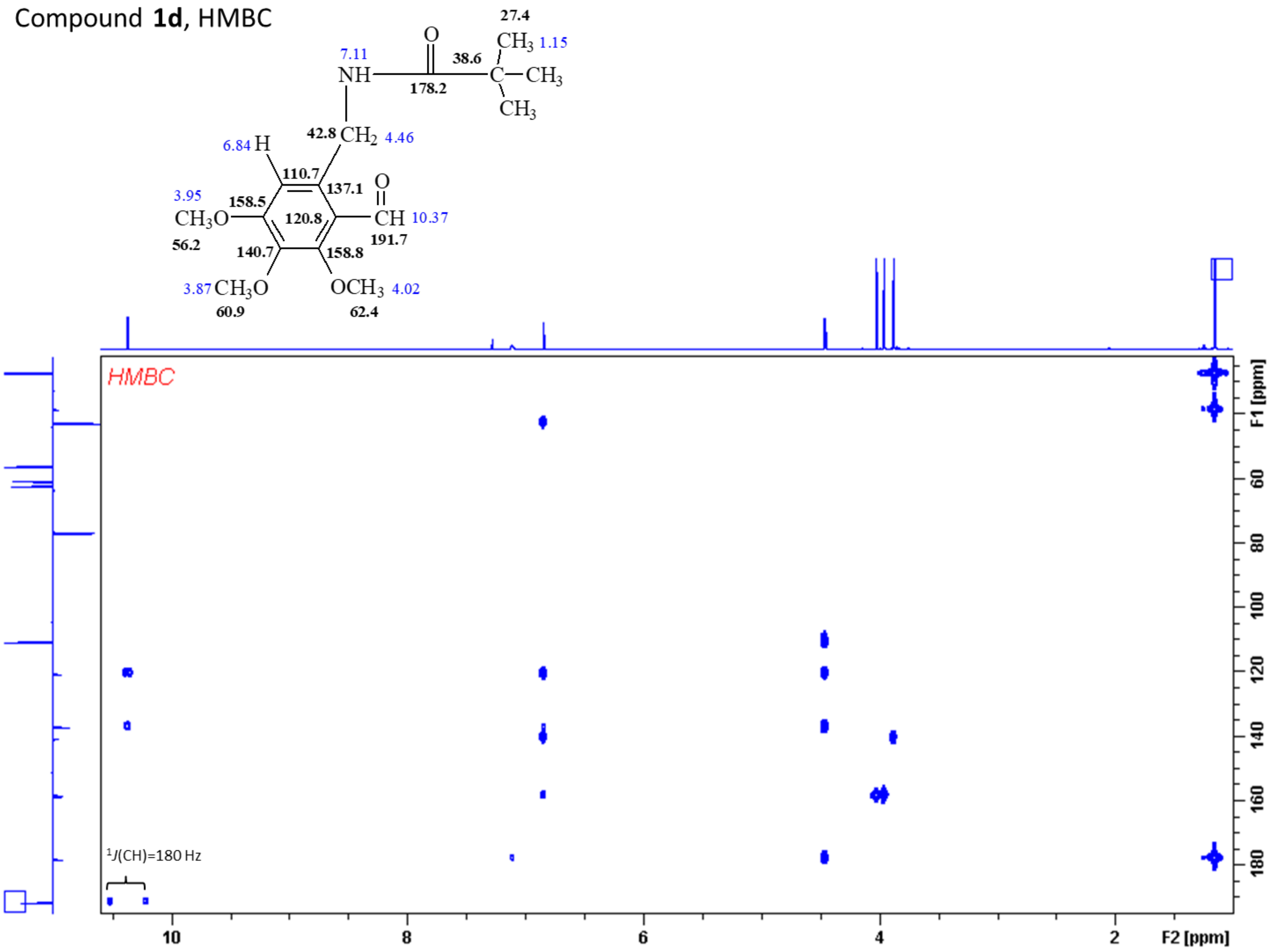

Compound **1e**, ^{1}H and ^{13}C NMR spectra (600/150 MHz)

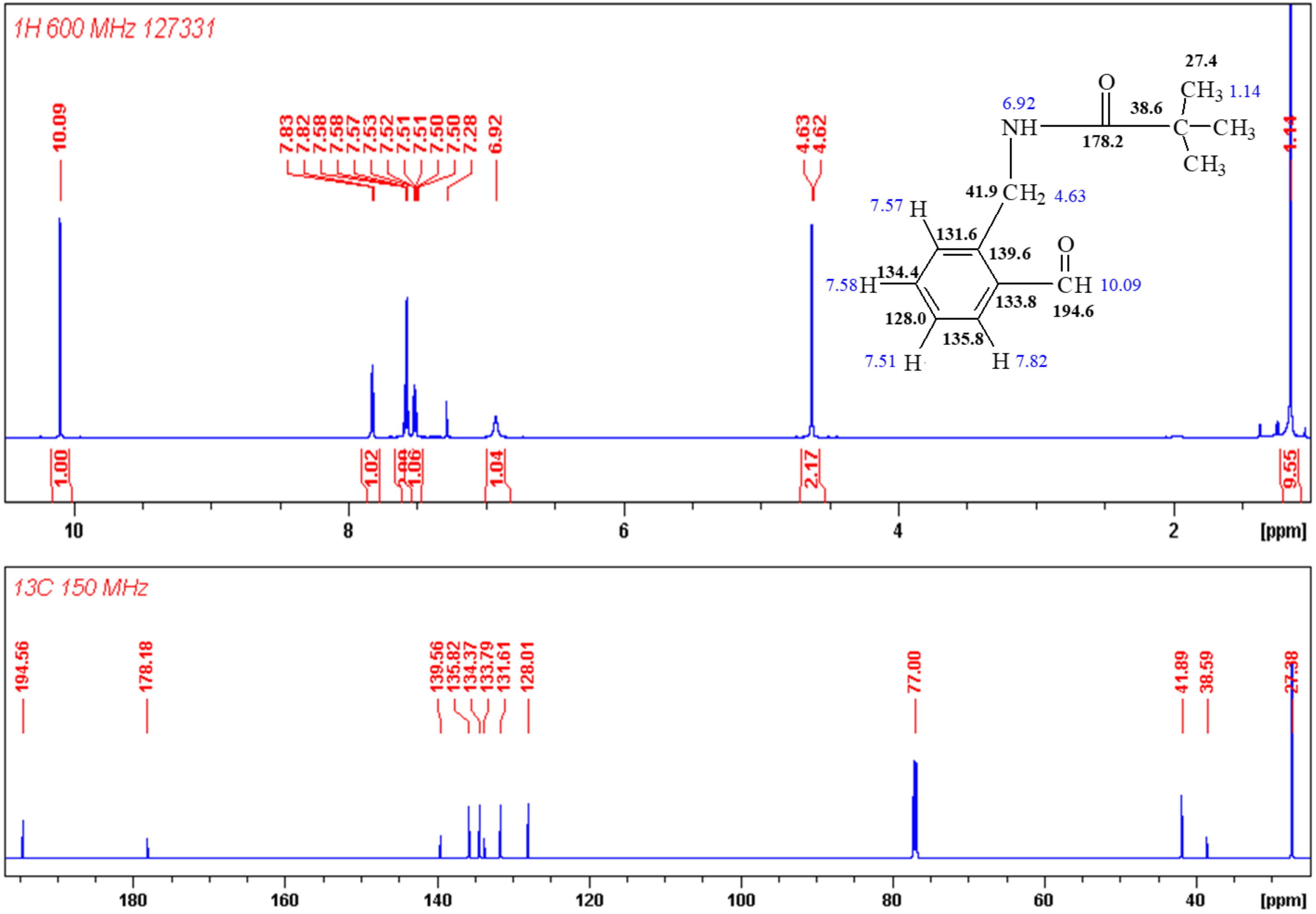

Compound **1e**, edHSQC and HMBC

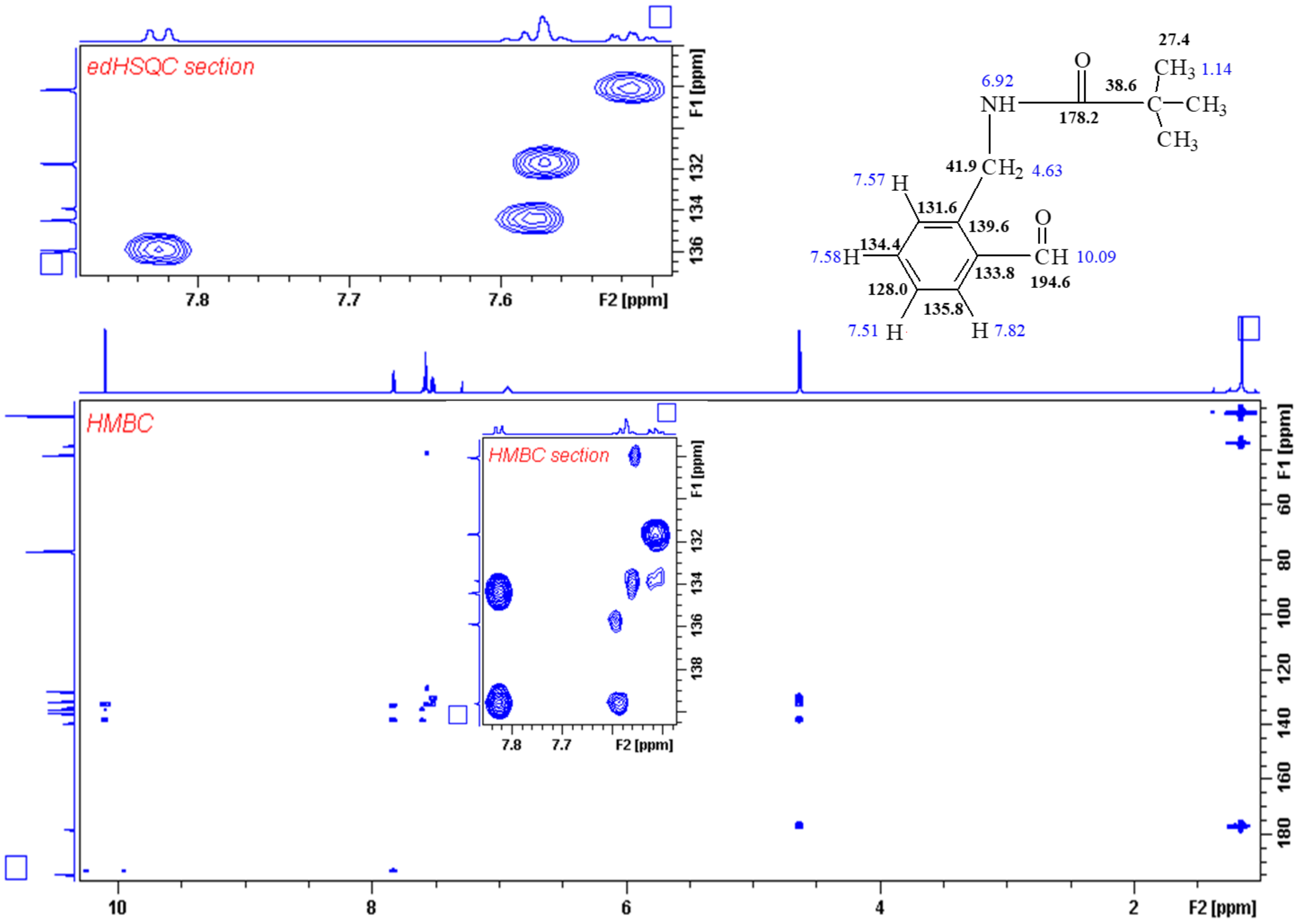

Compound **2b**, ^{1}H NMR with sel-NOE on signals at 4.59 and 9.92 ppm

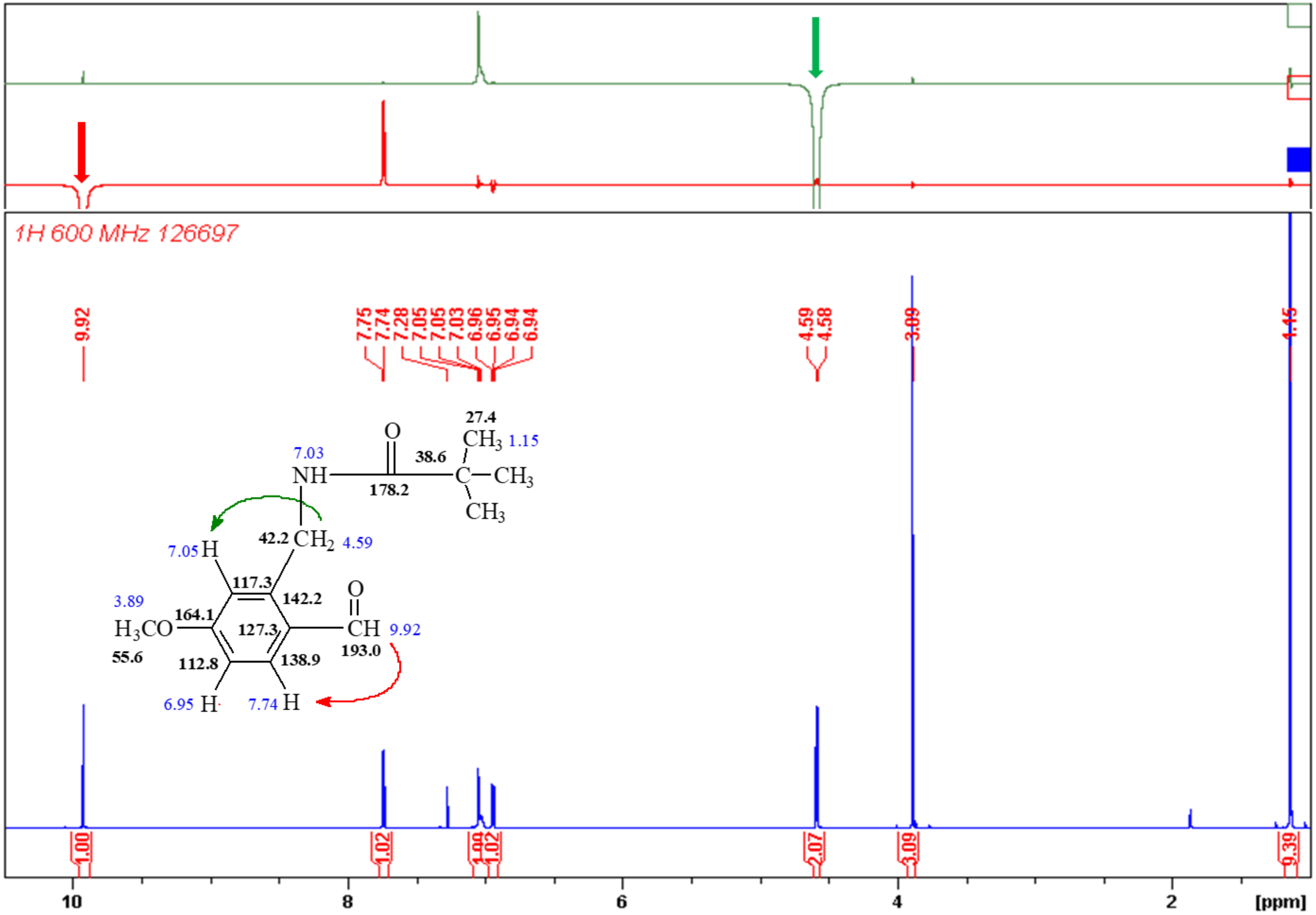

Compound **2b**, ^{13}C and Dept-135 NMR spectra (150 MHz)

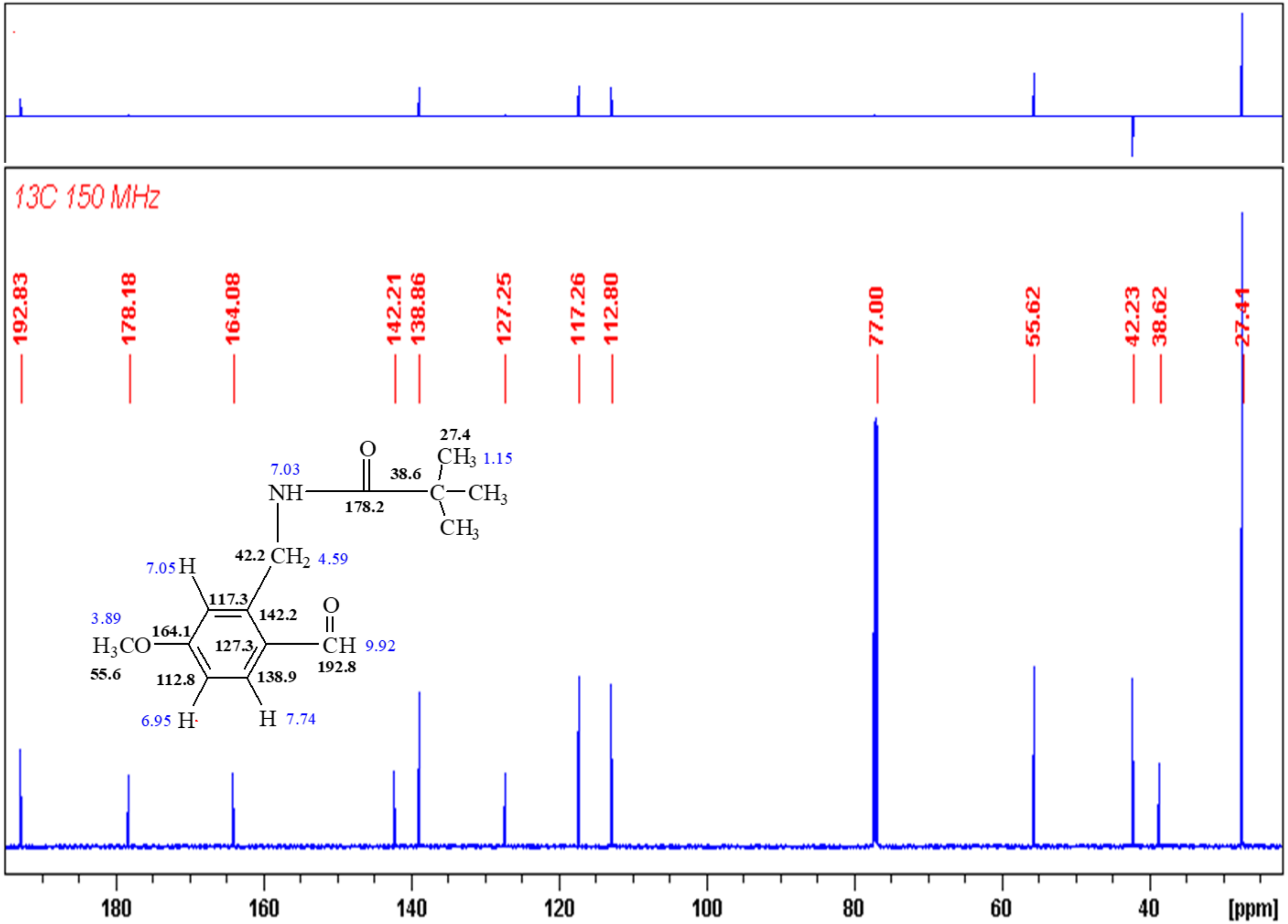

Compound **2b**, HSQC and HMBC

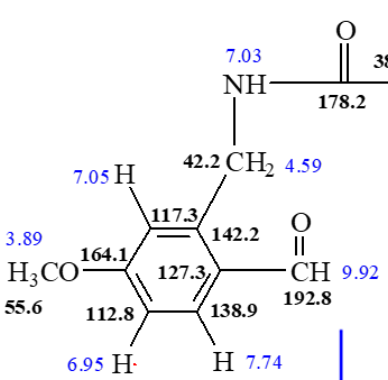

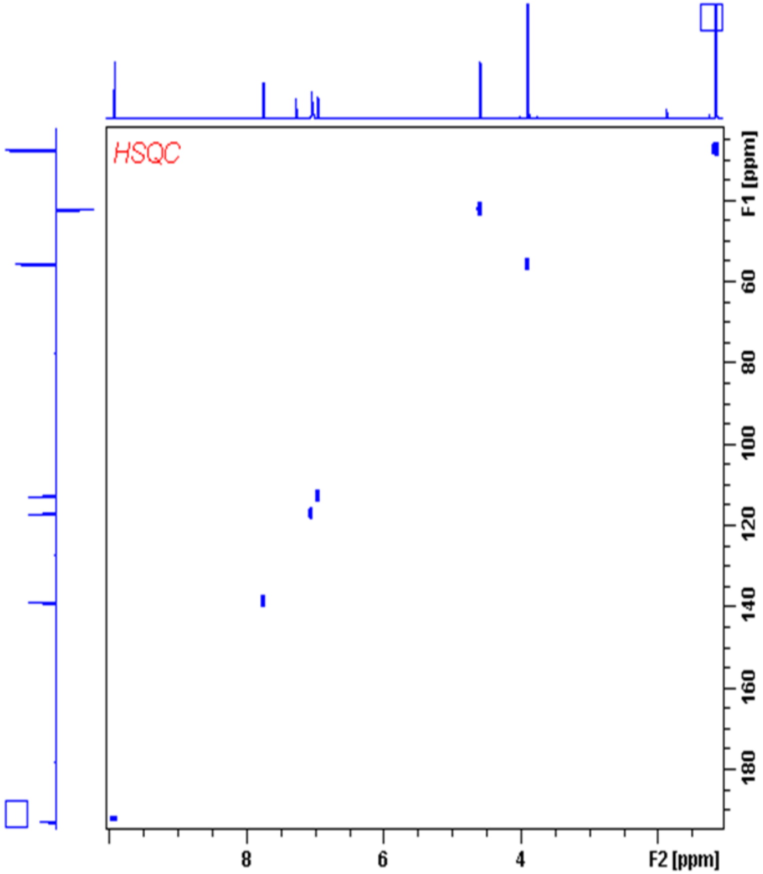

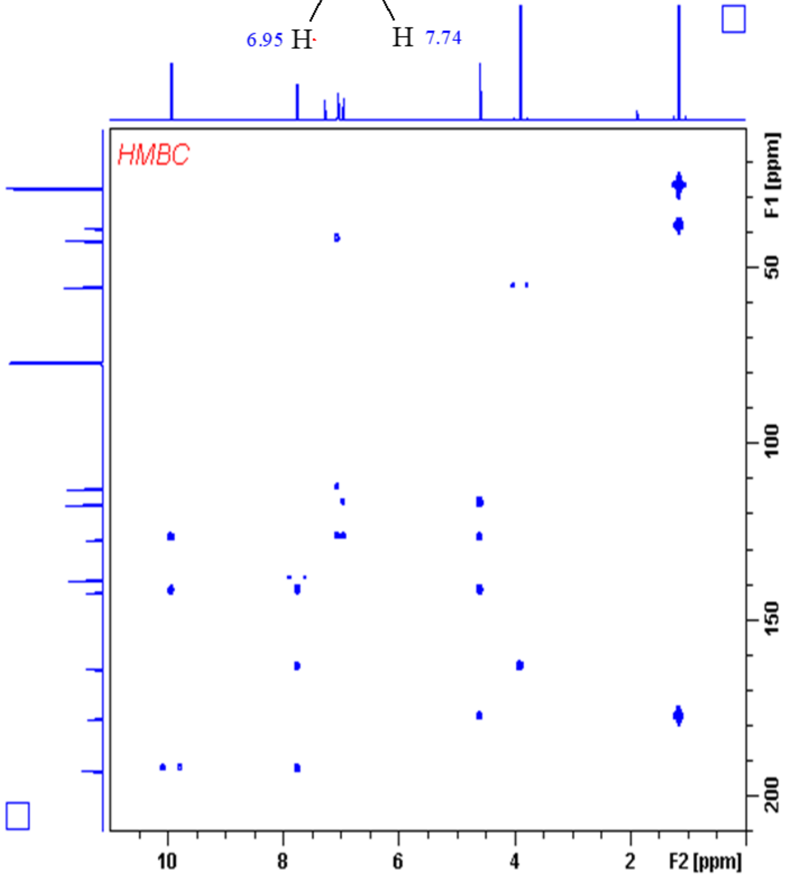

Compound **3b**, ^{1}H NMR 600 MHz $CDCl_3$

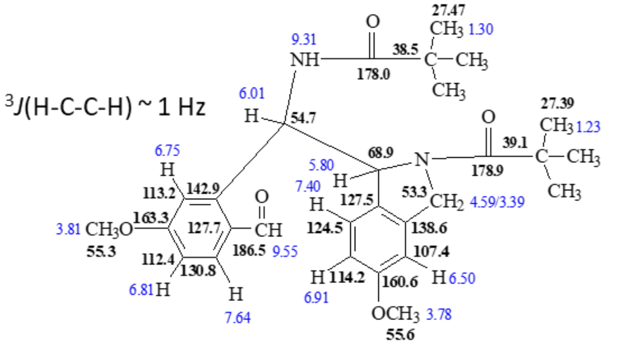

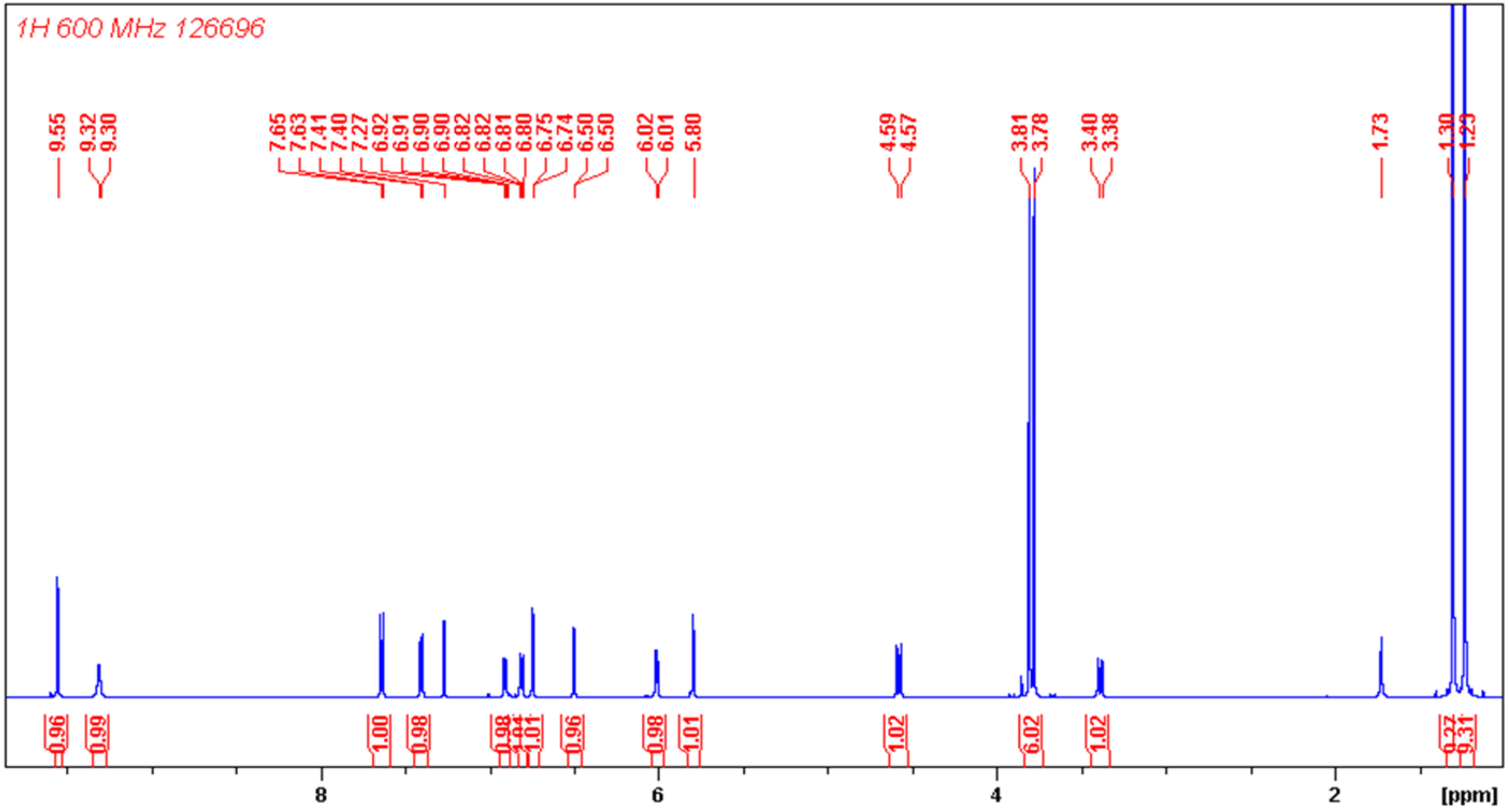

Compound **3b**, Steric proximities detected by selNOE on signals 9.55, 6.75 and 6.01

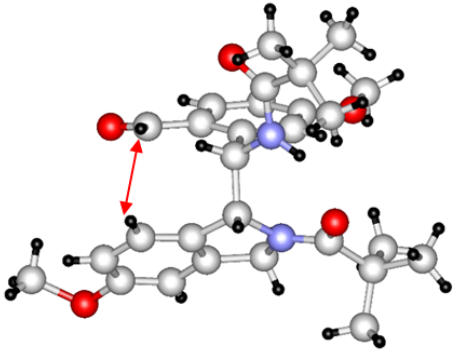

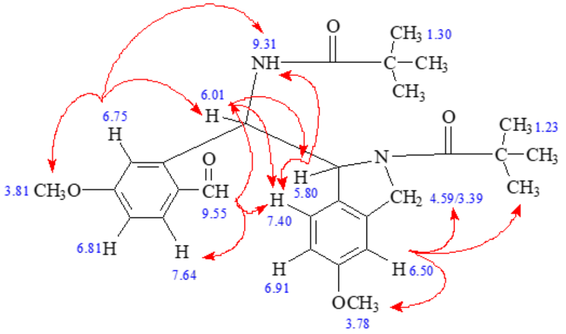

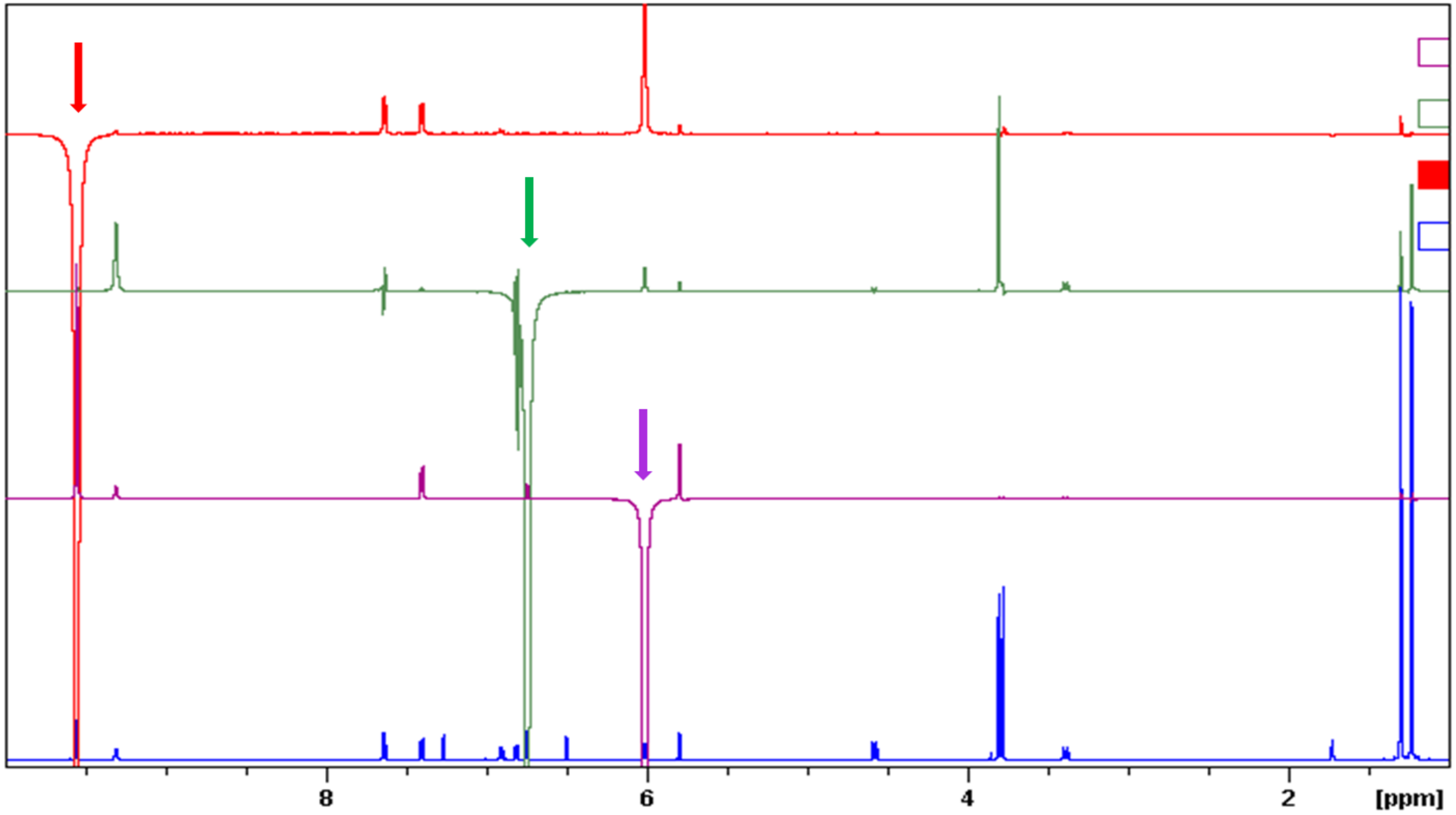

Compound **3b**, Steric proximities detected by selNOE on signals 6.50 and 5.80

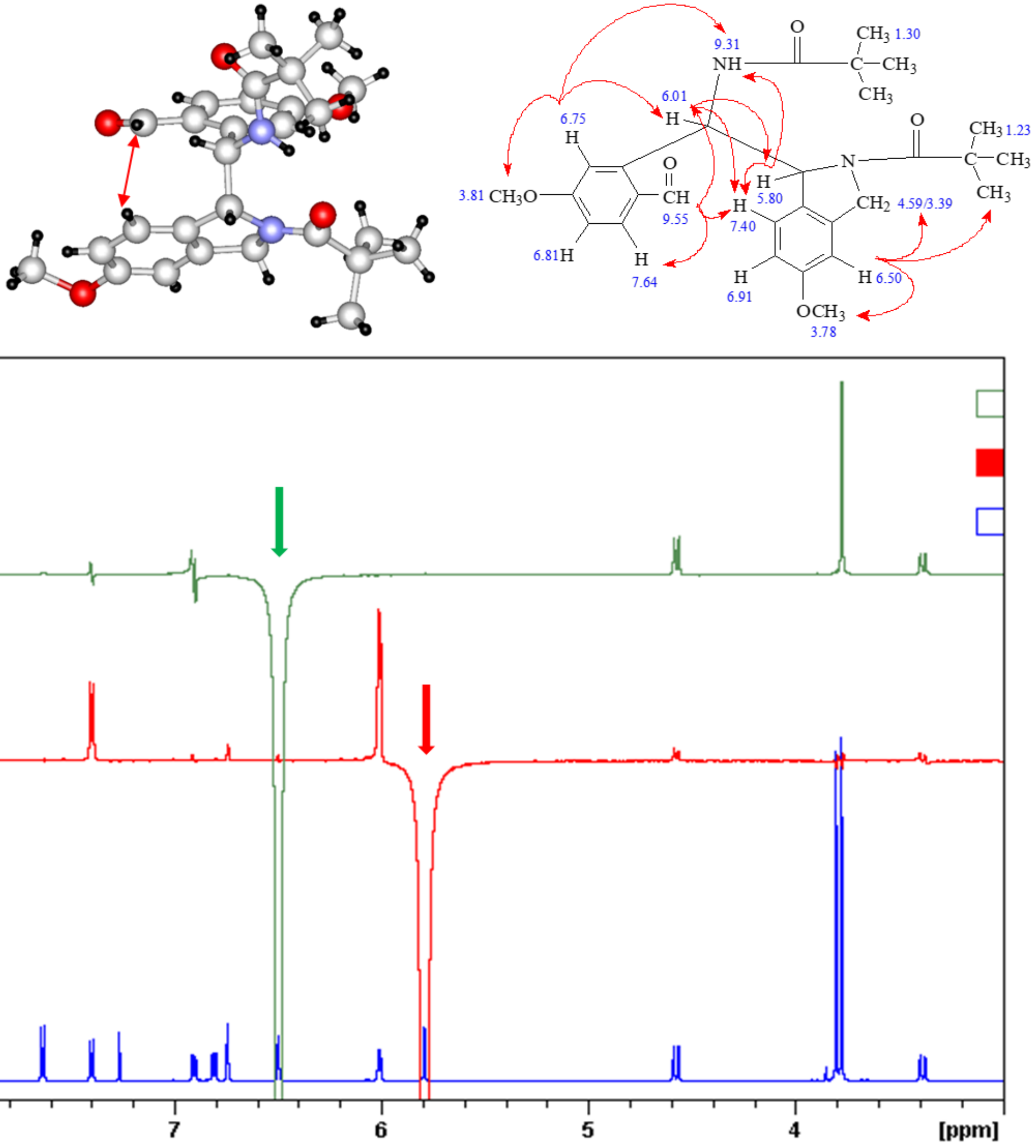

Compound **3b**, $^1H,^1H$-COSY

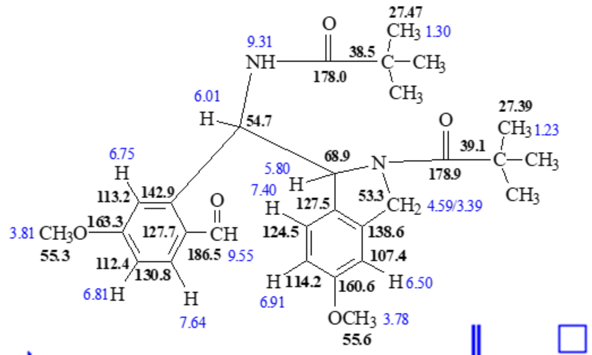

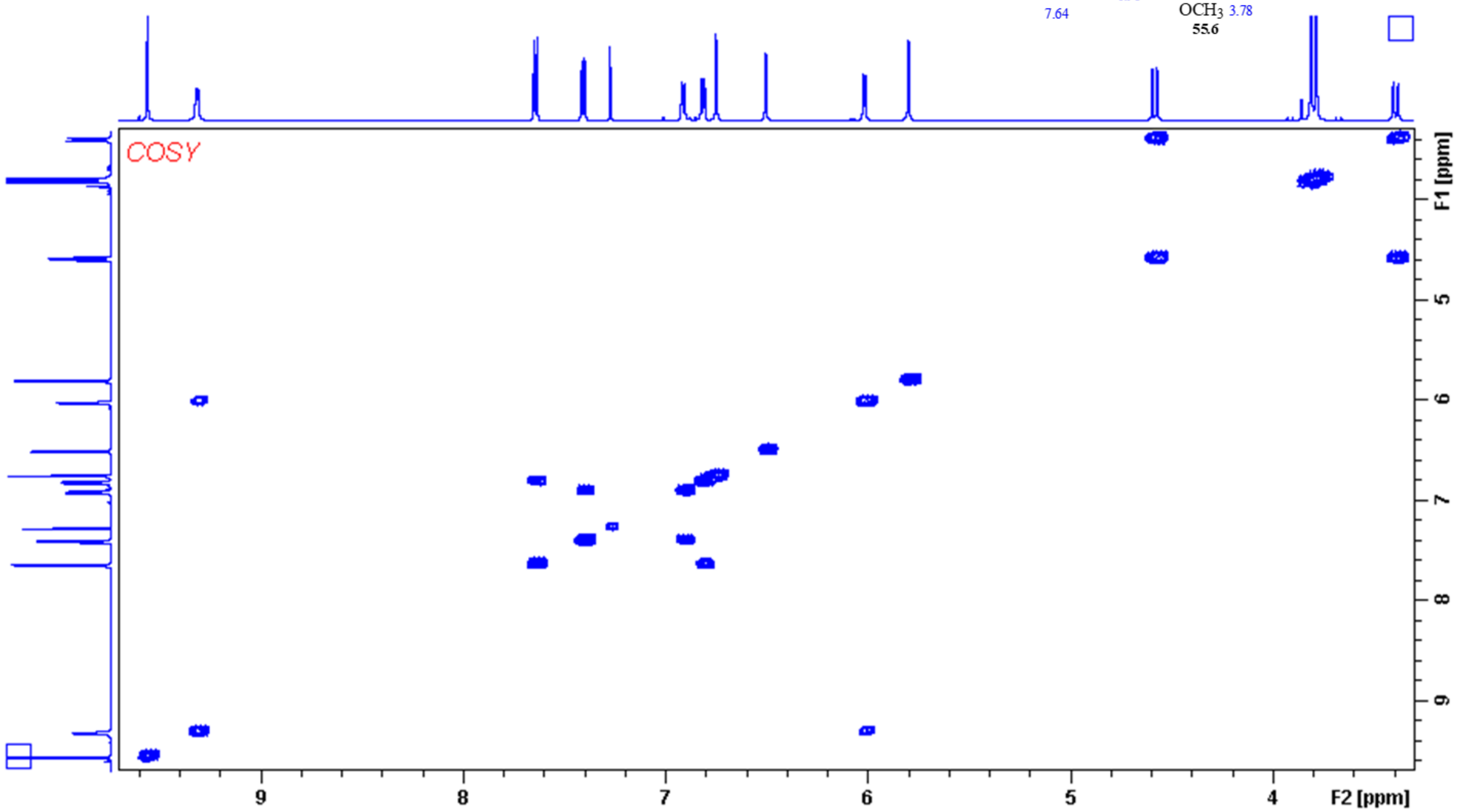

Compound **3b**, ^{13}C + DEPT-135

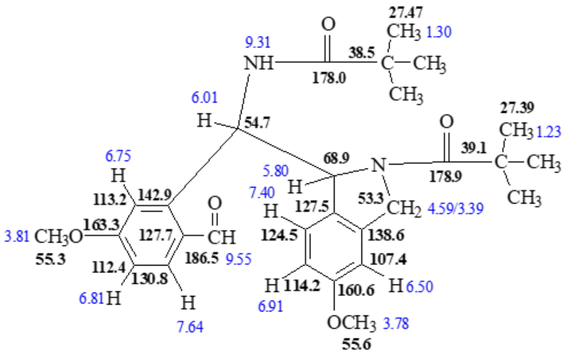

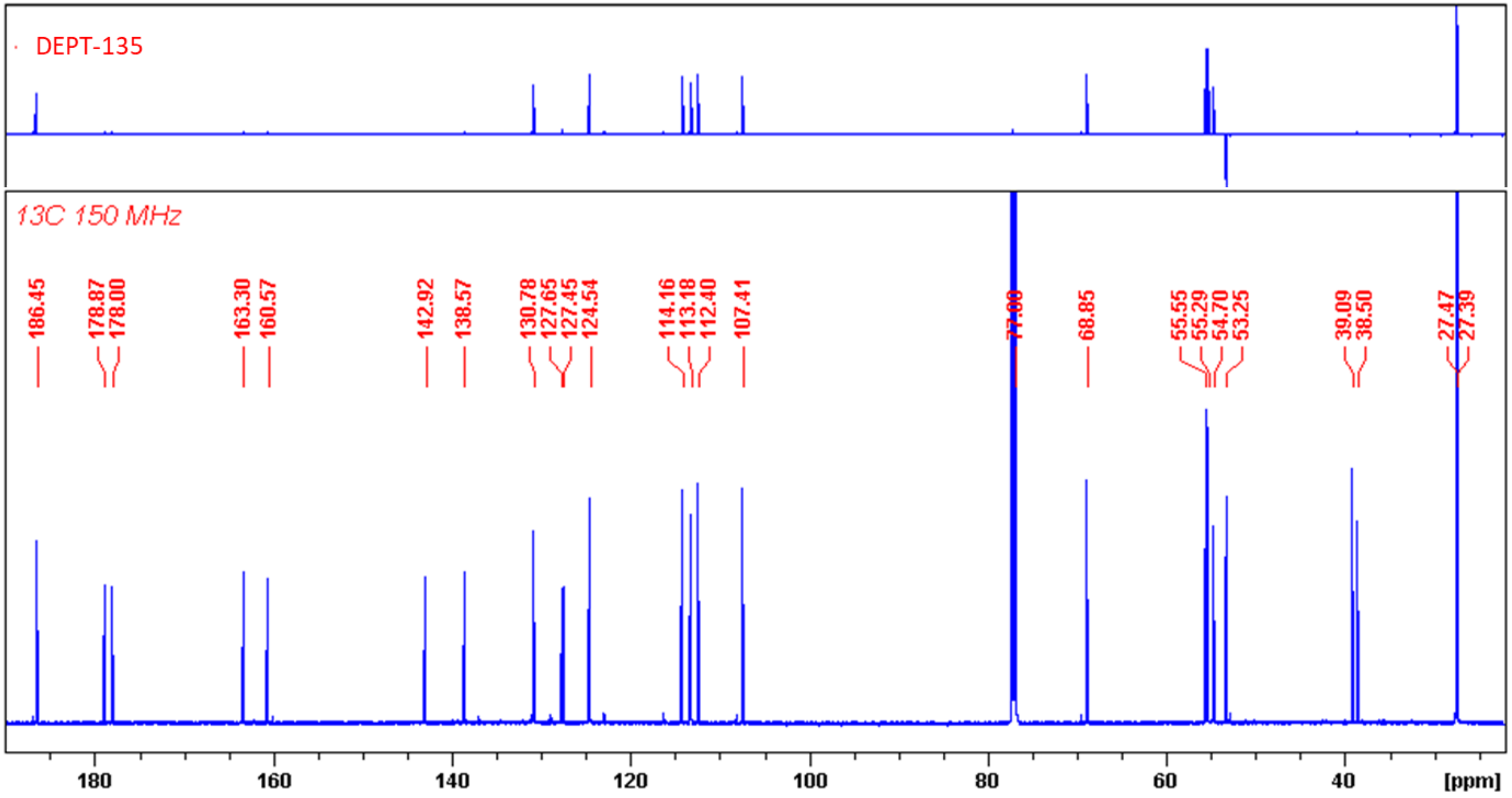

Compound **3b**, HSQC + selective HSQC

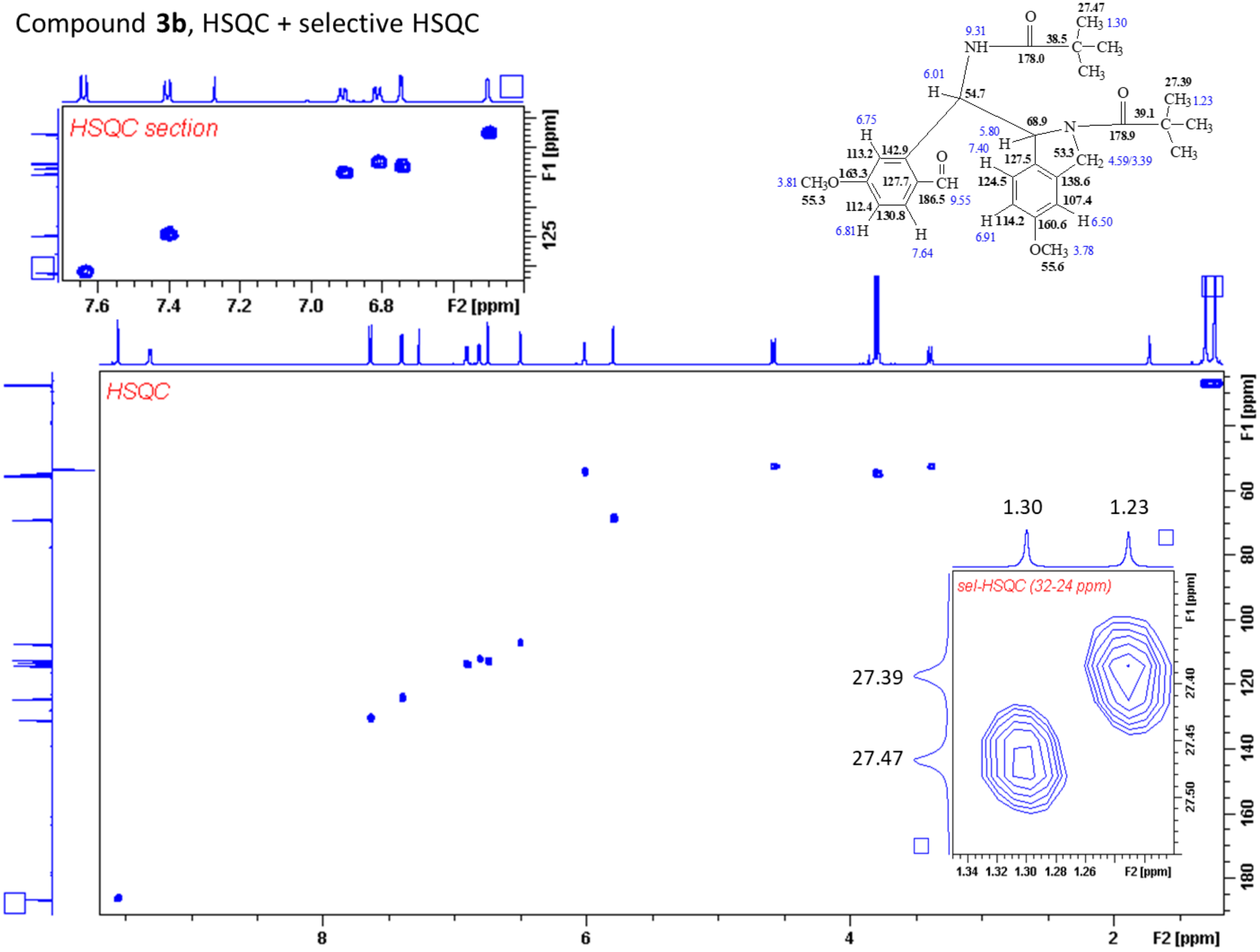

Compound **3b**, HMBC

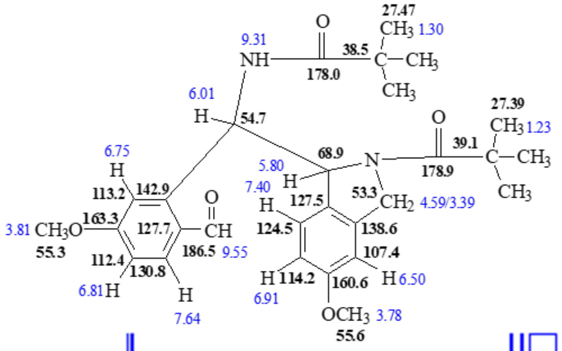

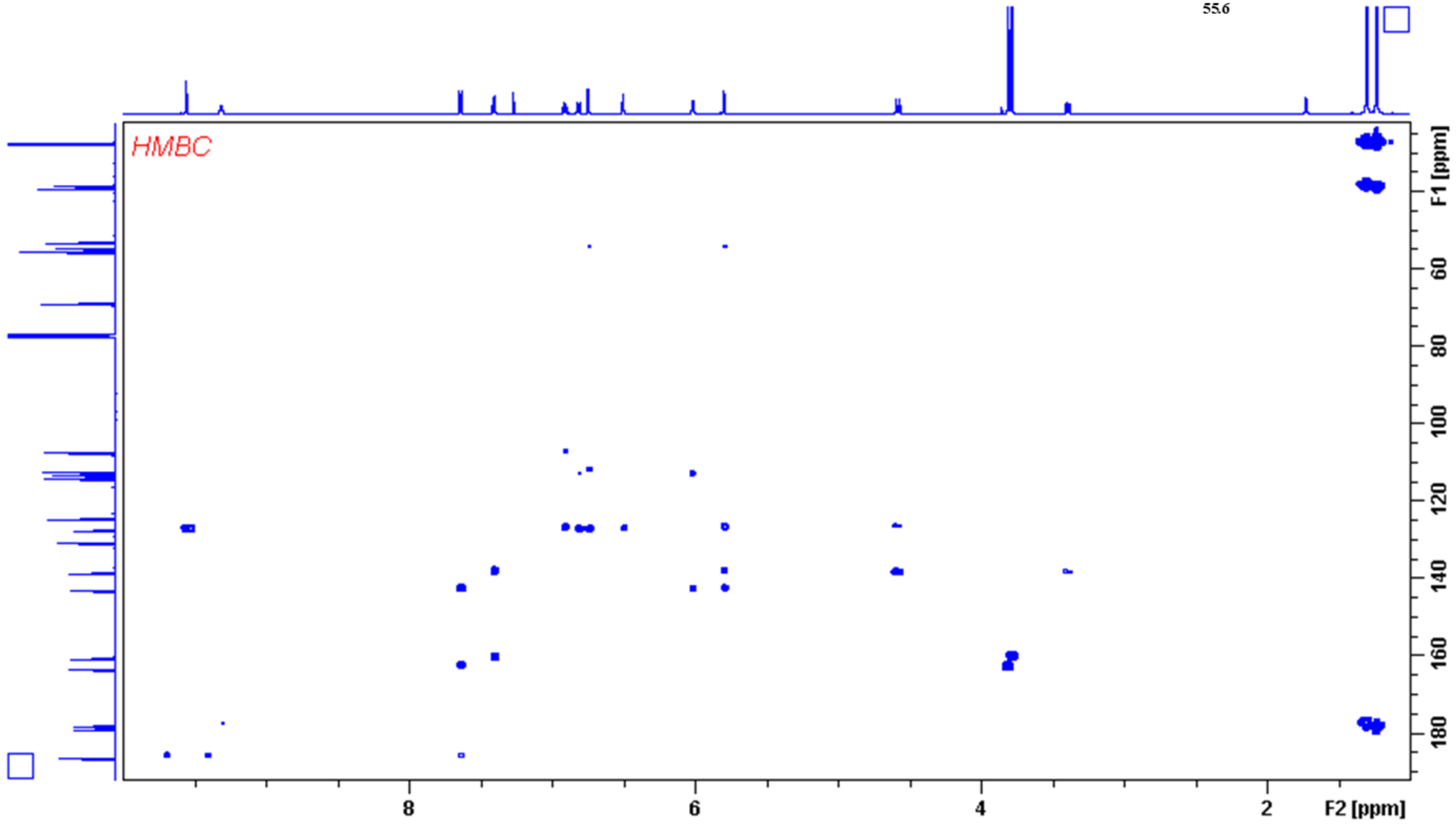

Compound **3b**, HMBC section

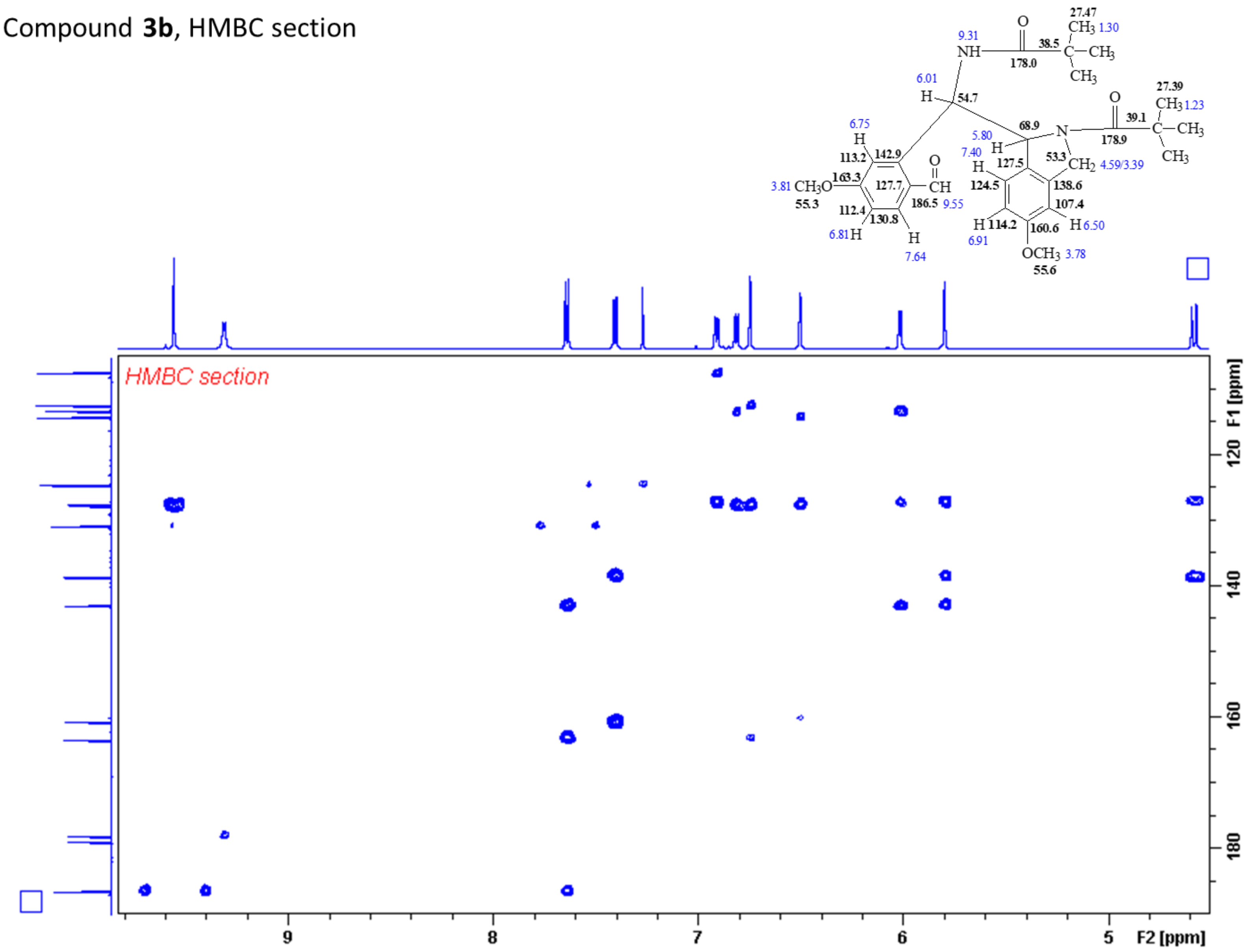

Compound **8a**, ^1H and ^{13}C NMR (600/150 MHz in $CDCl_3$)

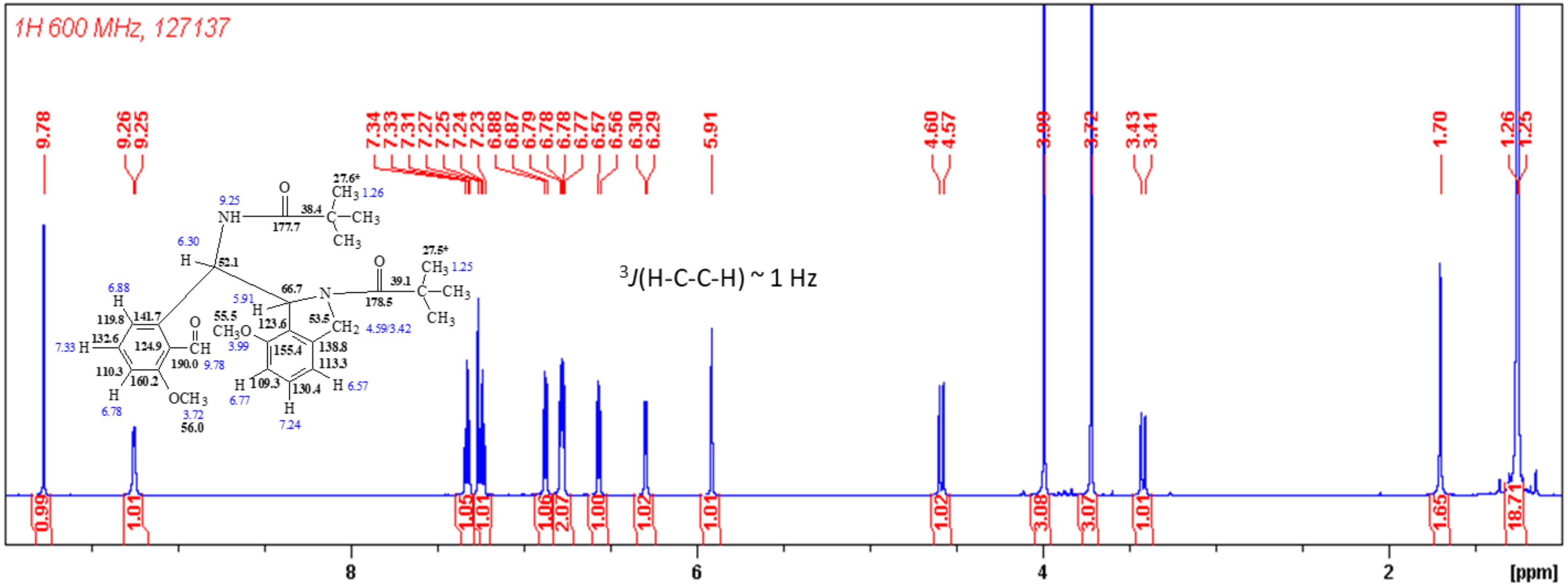

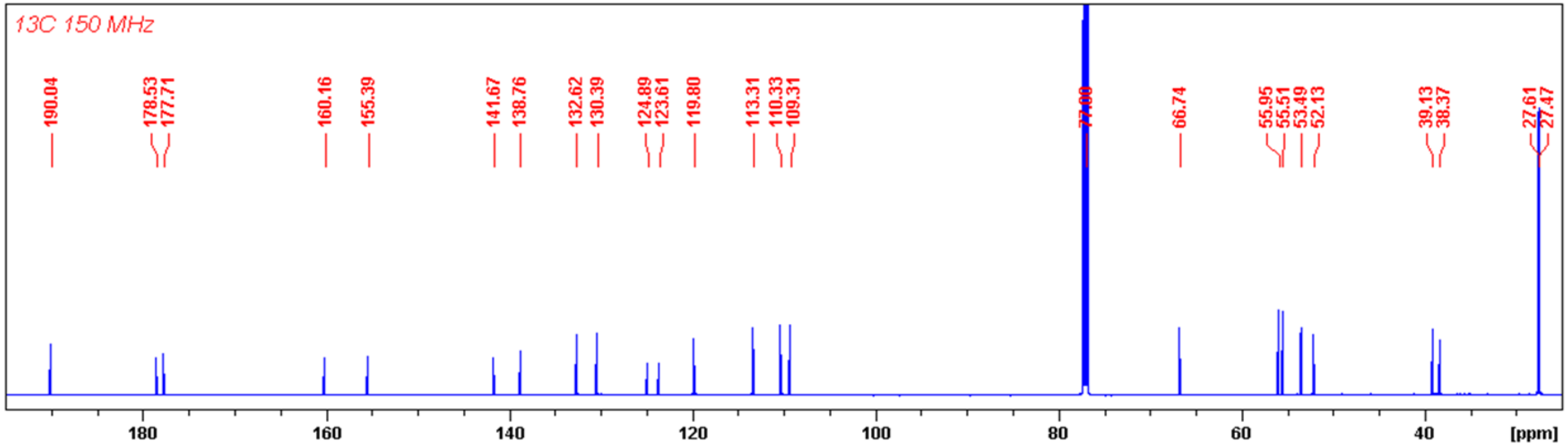

Compound **8a**, $^{1}H,^{1}H$-COSY

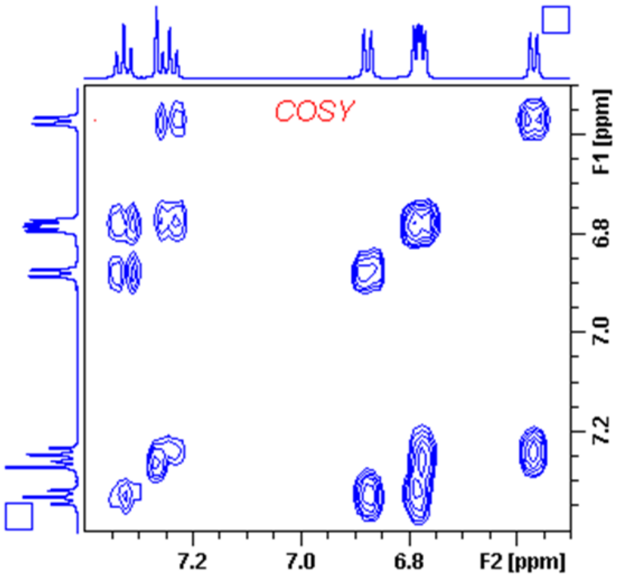

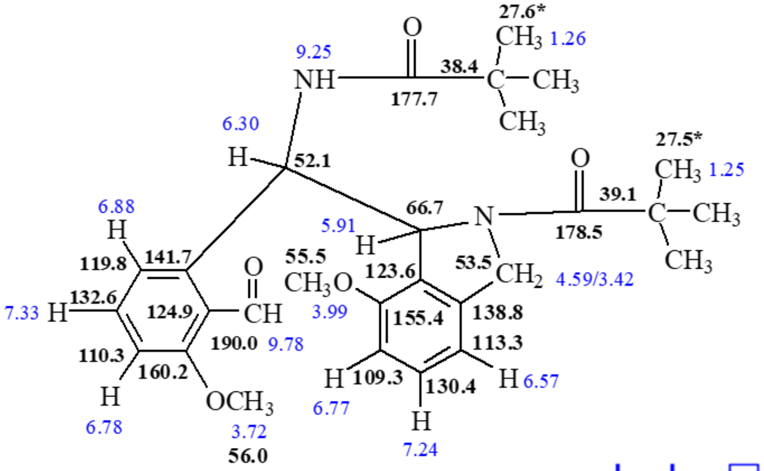

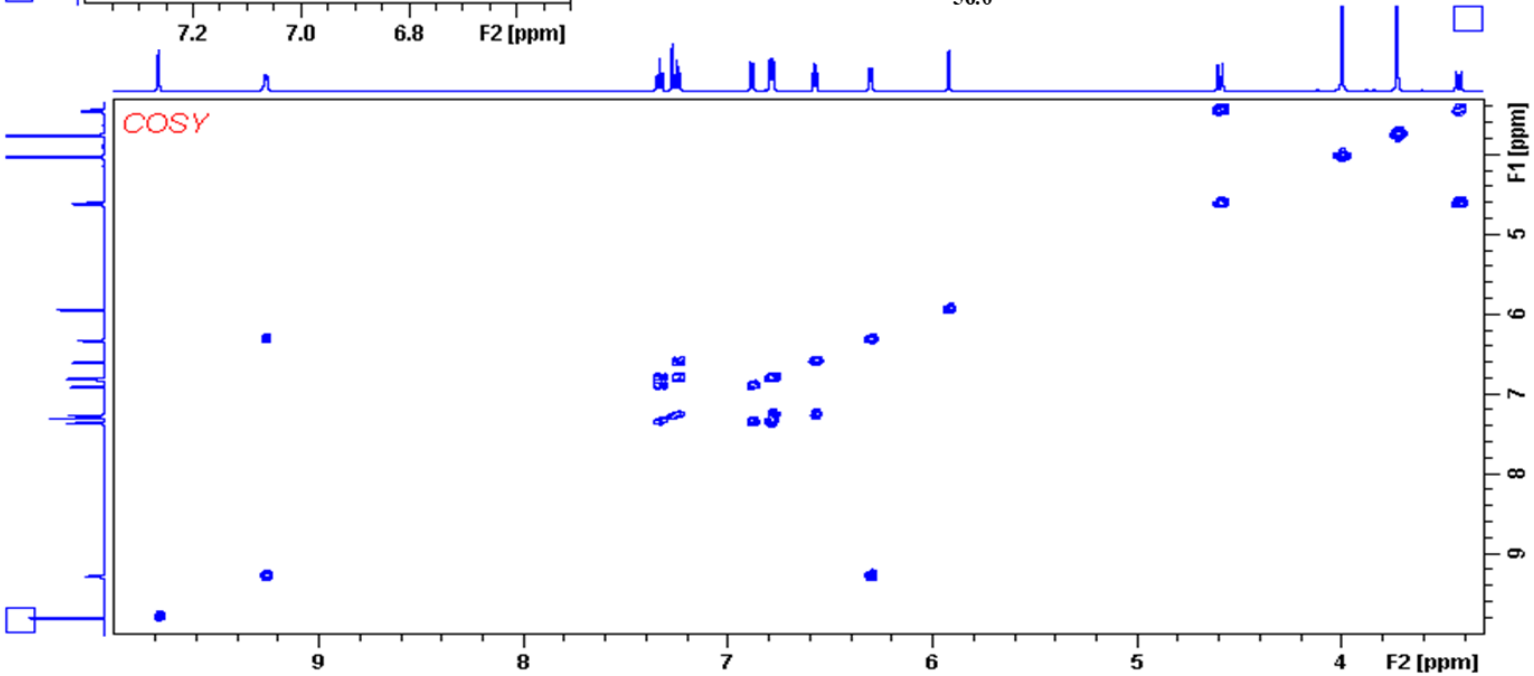

Compound 8a, Steric proximities detected by selNOE on signals 9.78, 6.88 and 6.30

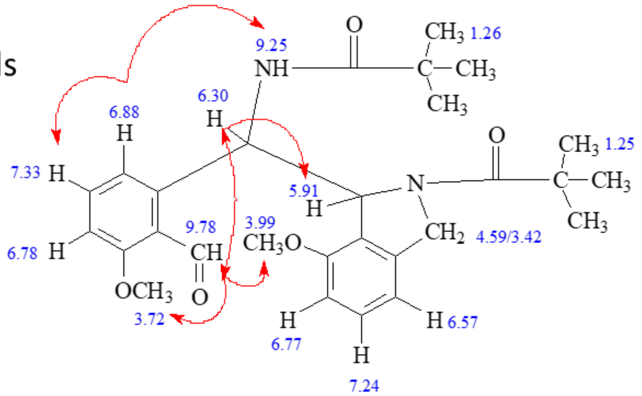

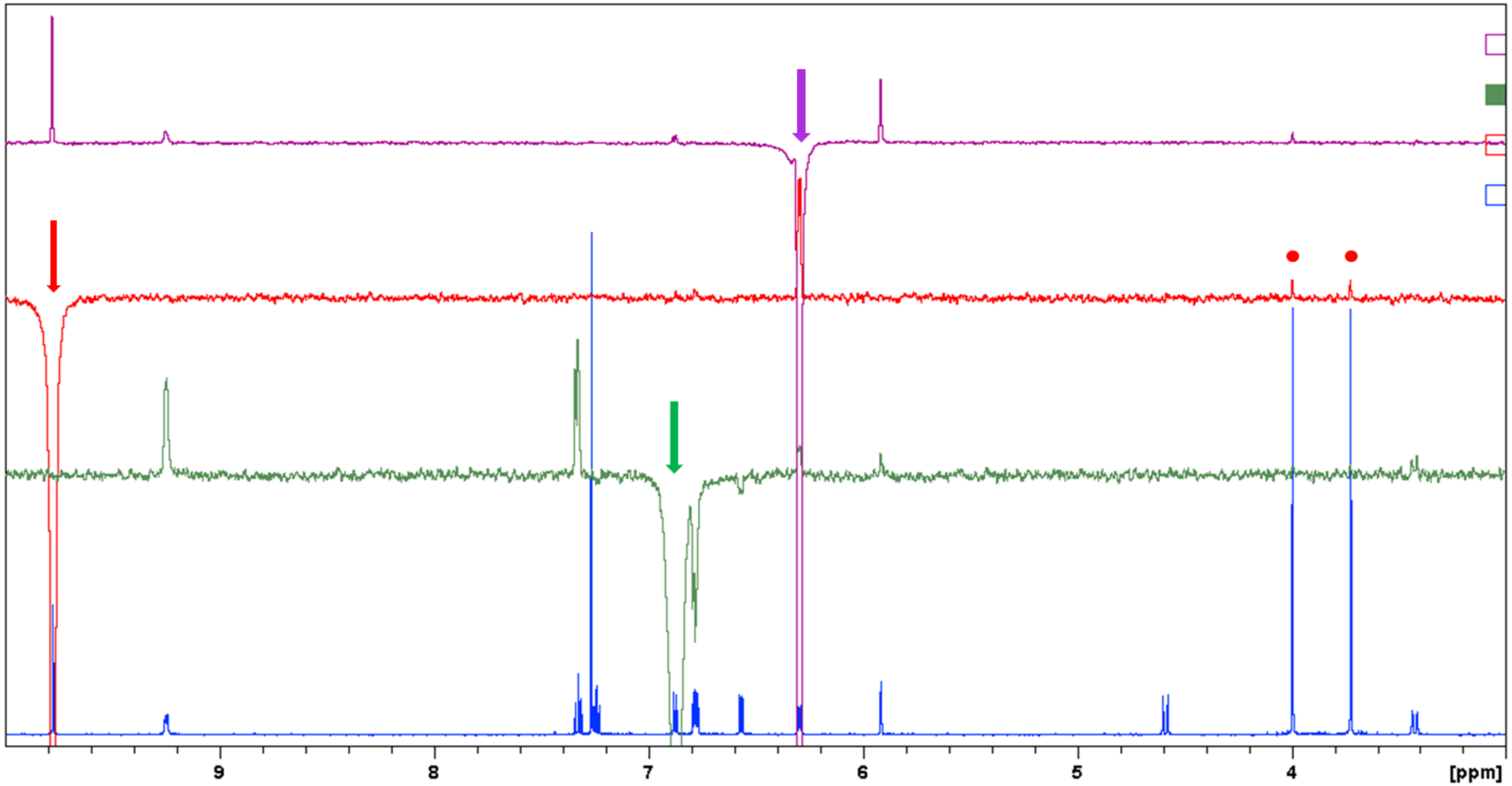

Compound **8a**, Steric proximities detected by selNOE on signals 5.91, 4.59 and 3.42

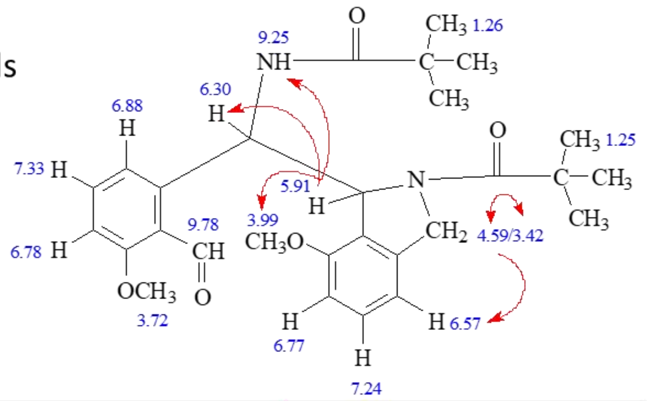

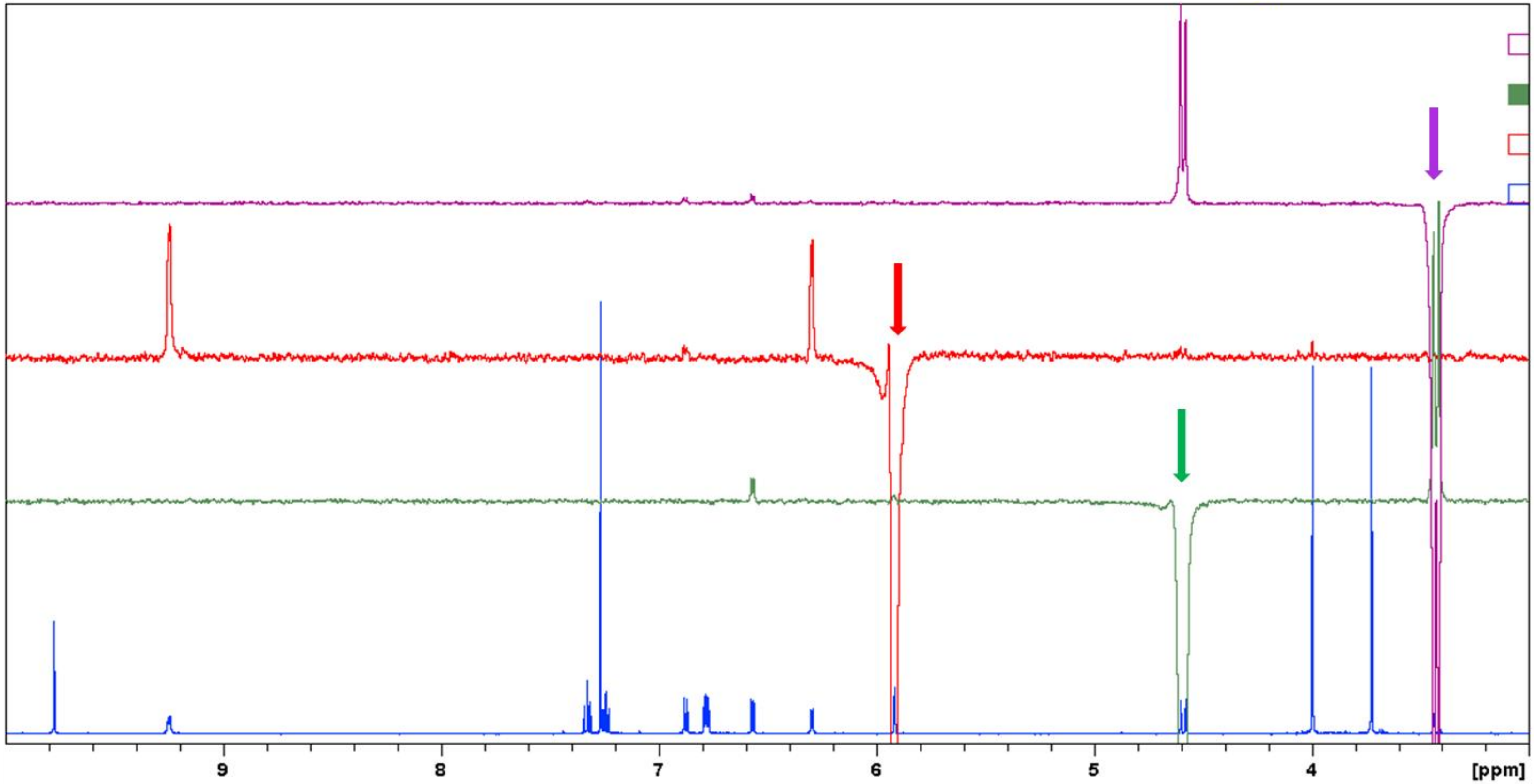

Compound **8a**, HSQC

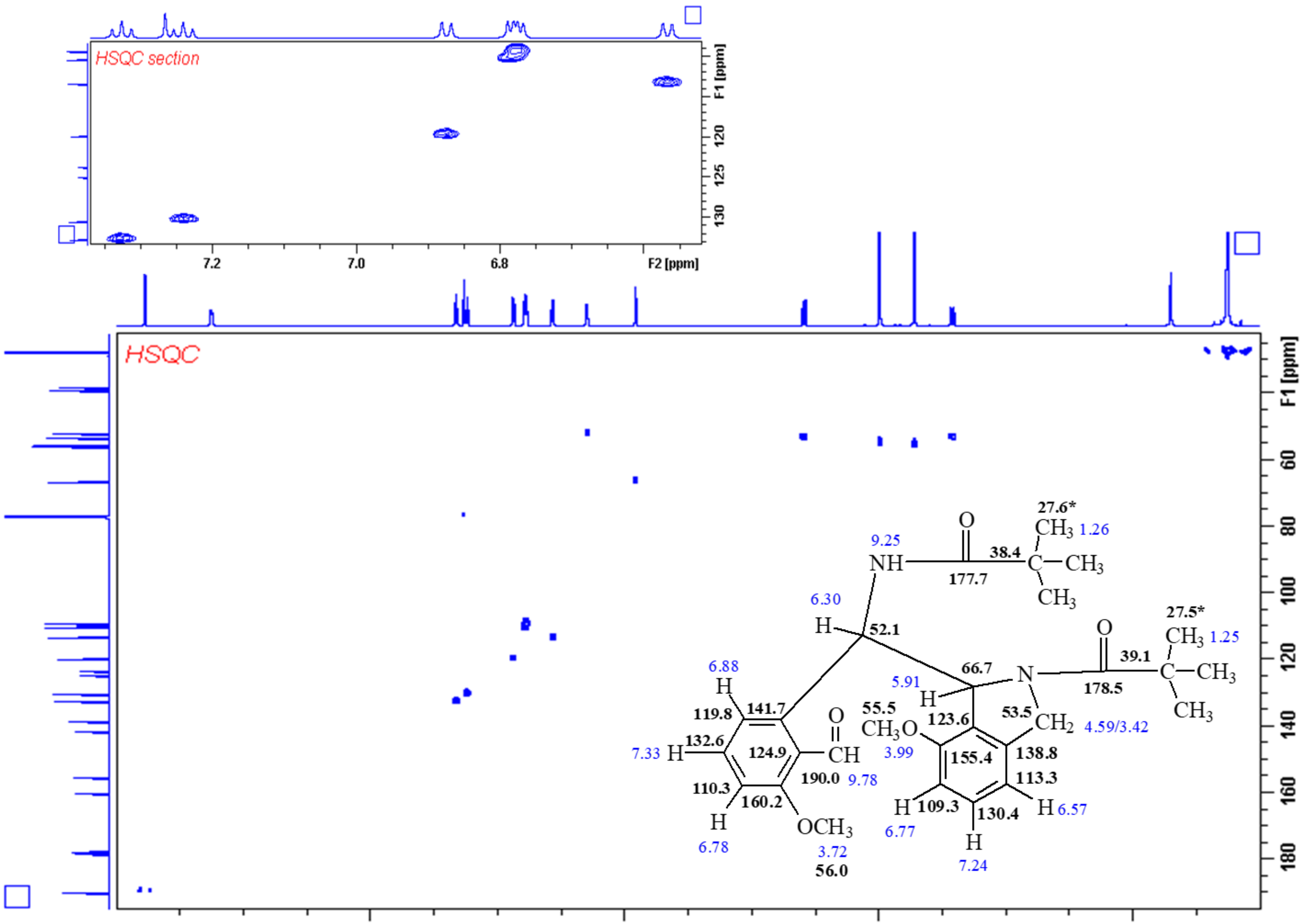

Compound 8a, HMBC

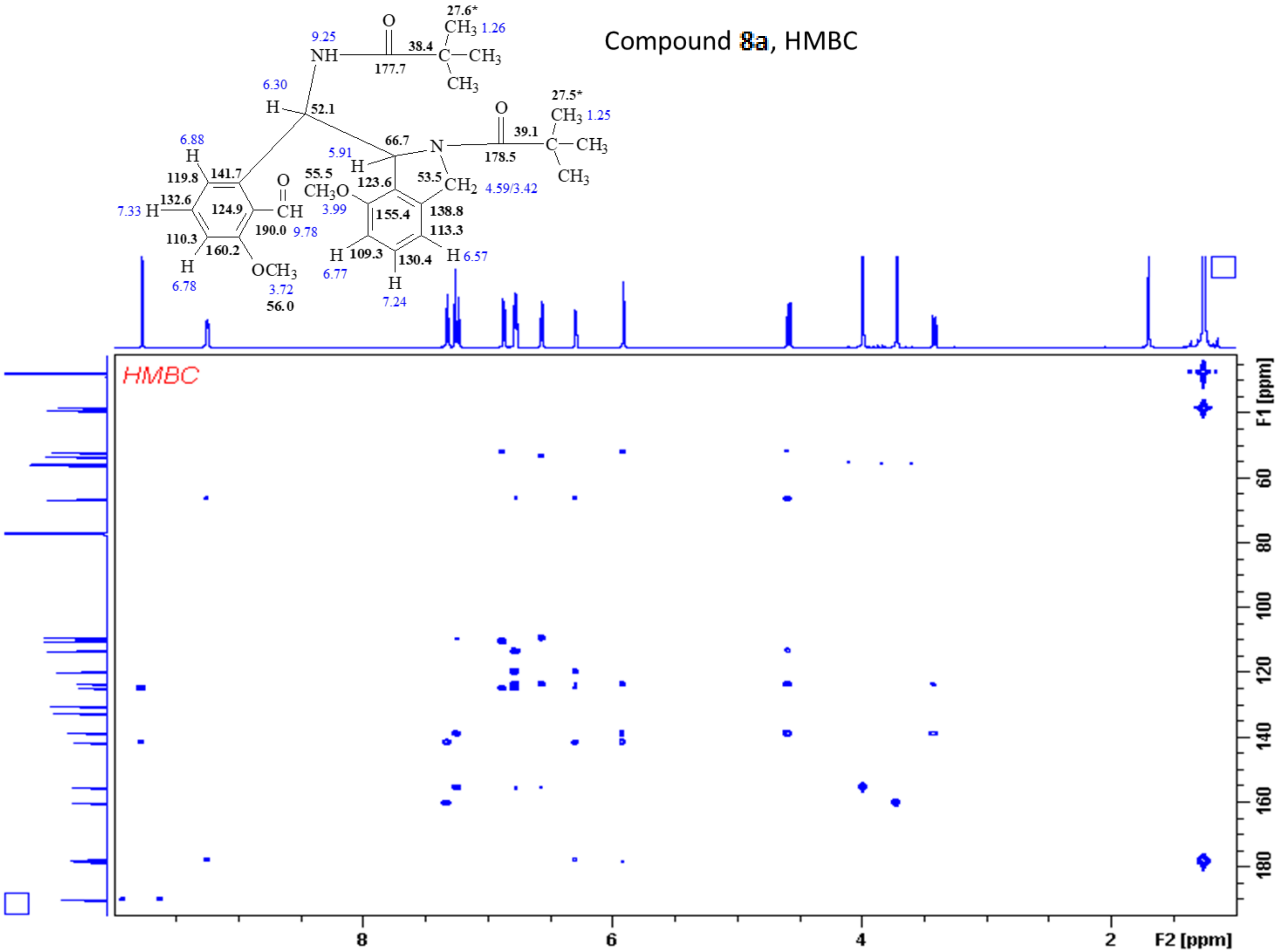

Compound **8b**, ^{1}H and ^{1}H,^{1}H-COSY 600 MHz

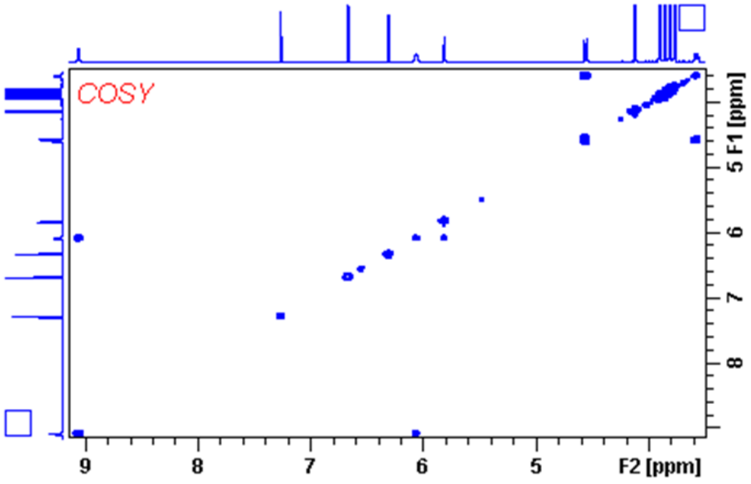

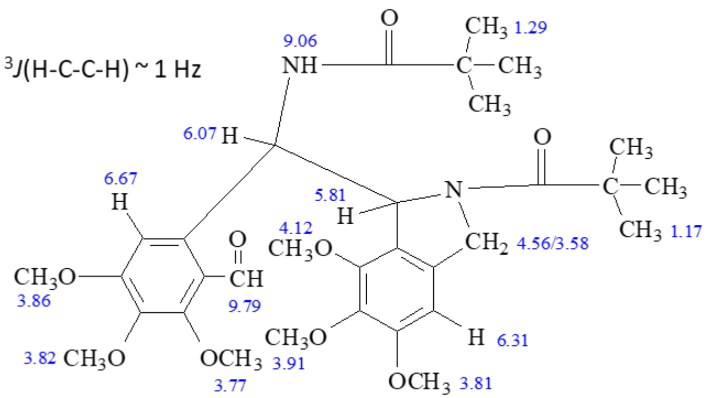

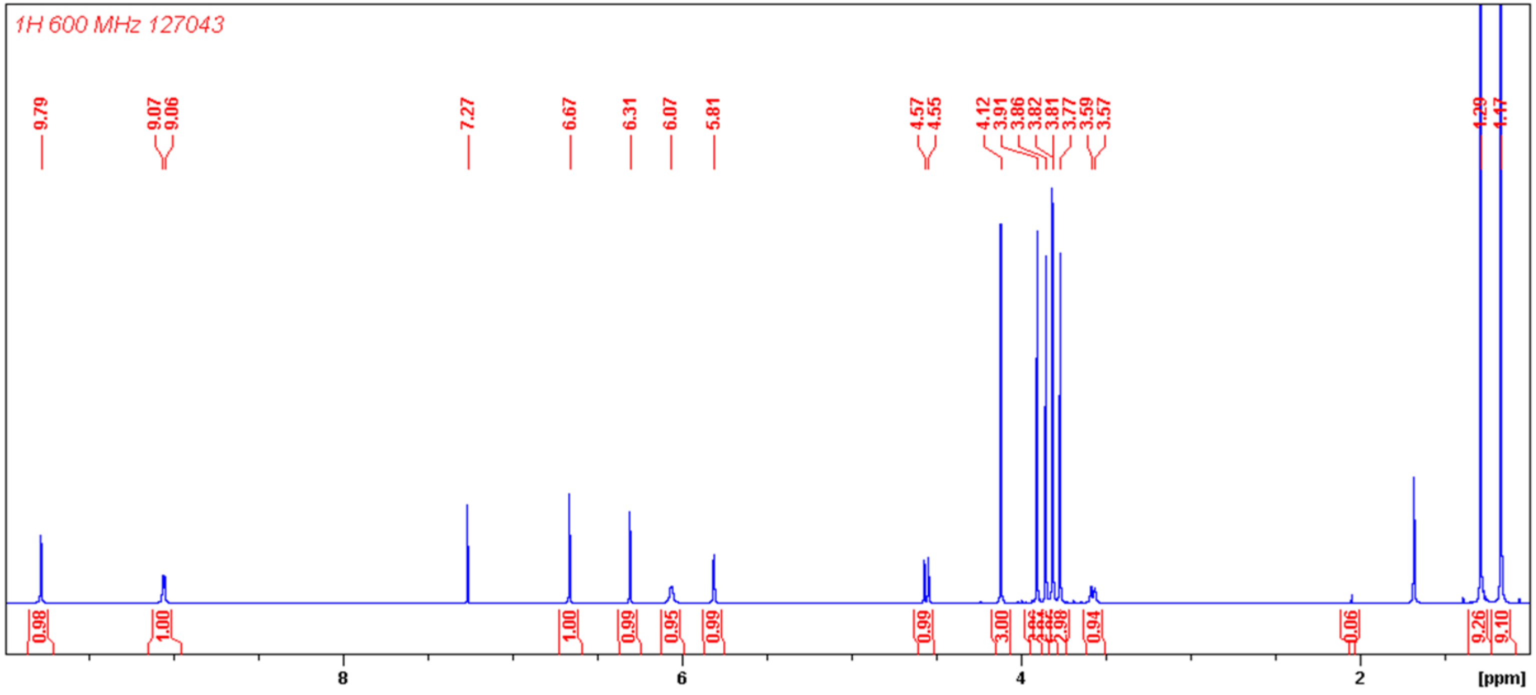

Compound **8b**, Steric proximities detected by selNOE on signals 9.79, 9.06 and 6.67

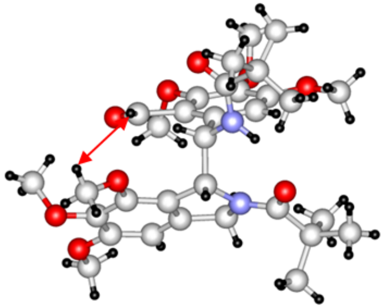

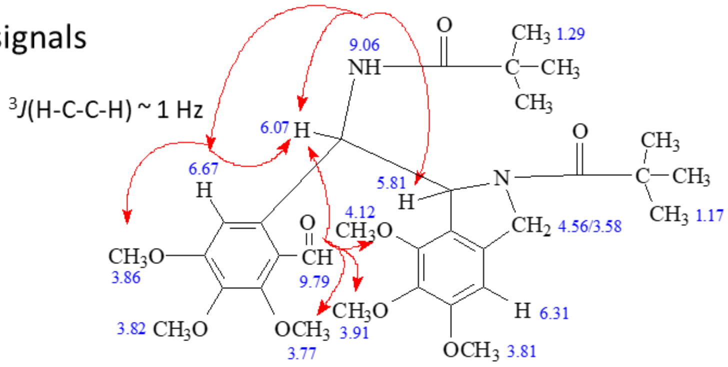

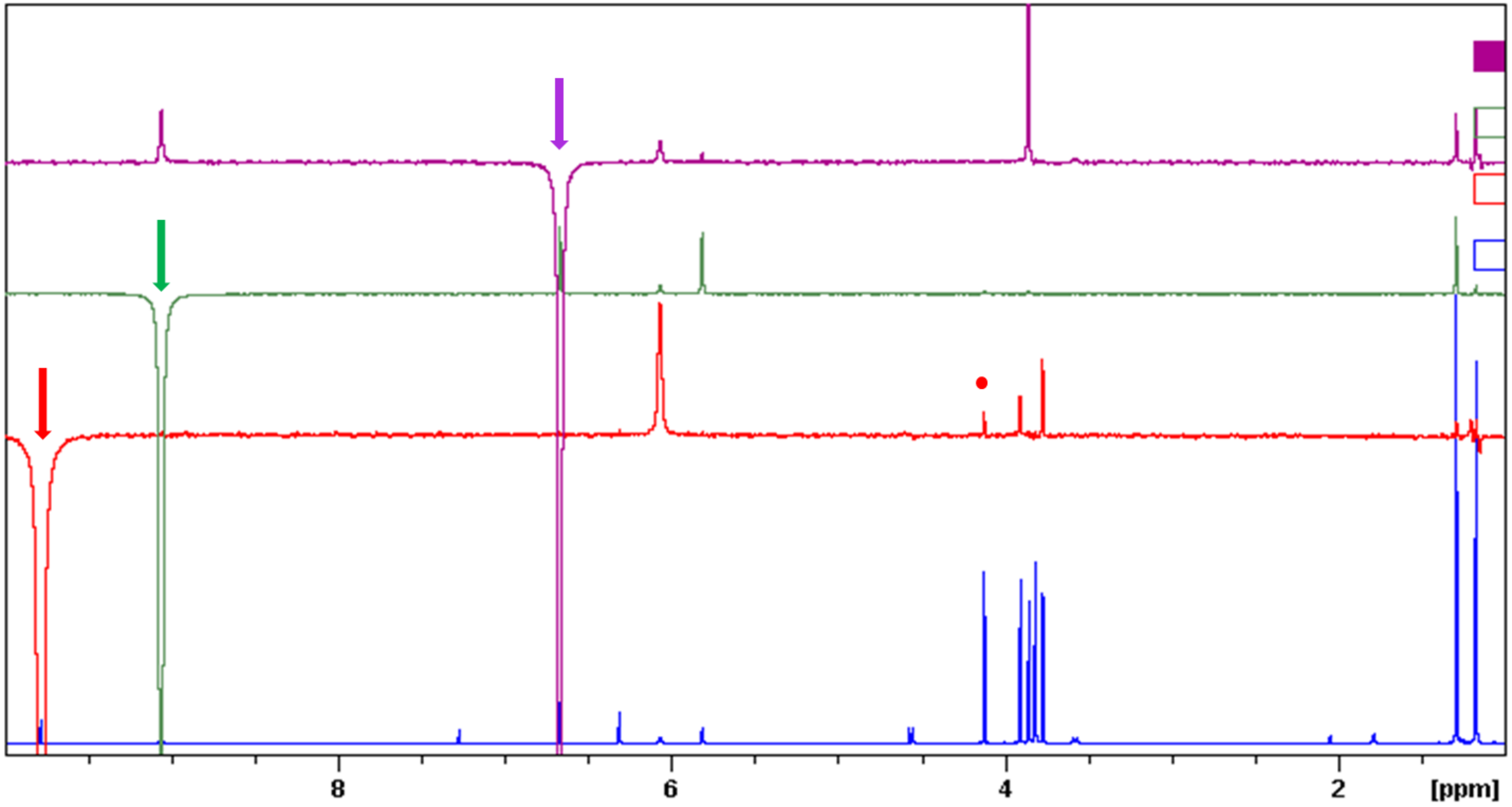

Compound **8b**, Steric proximities detected by selNOE on signals 6.07, 5.81 and 4.56

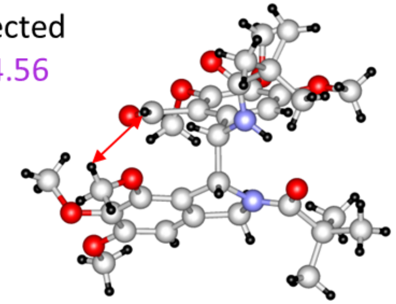

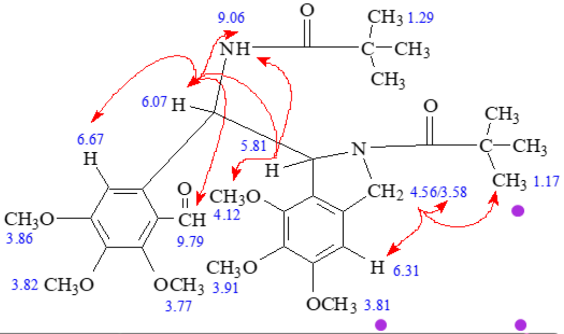

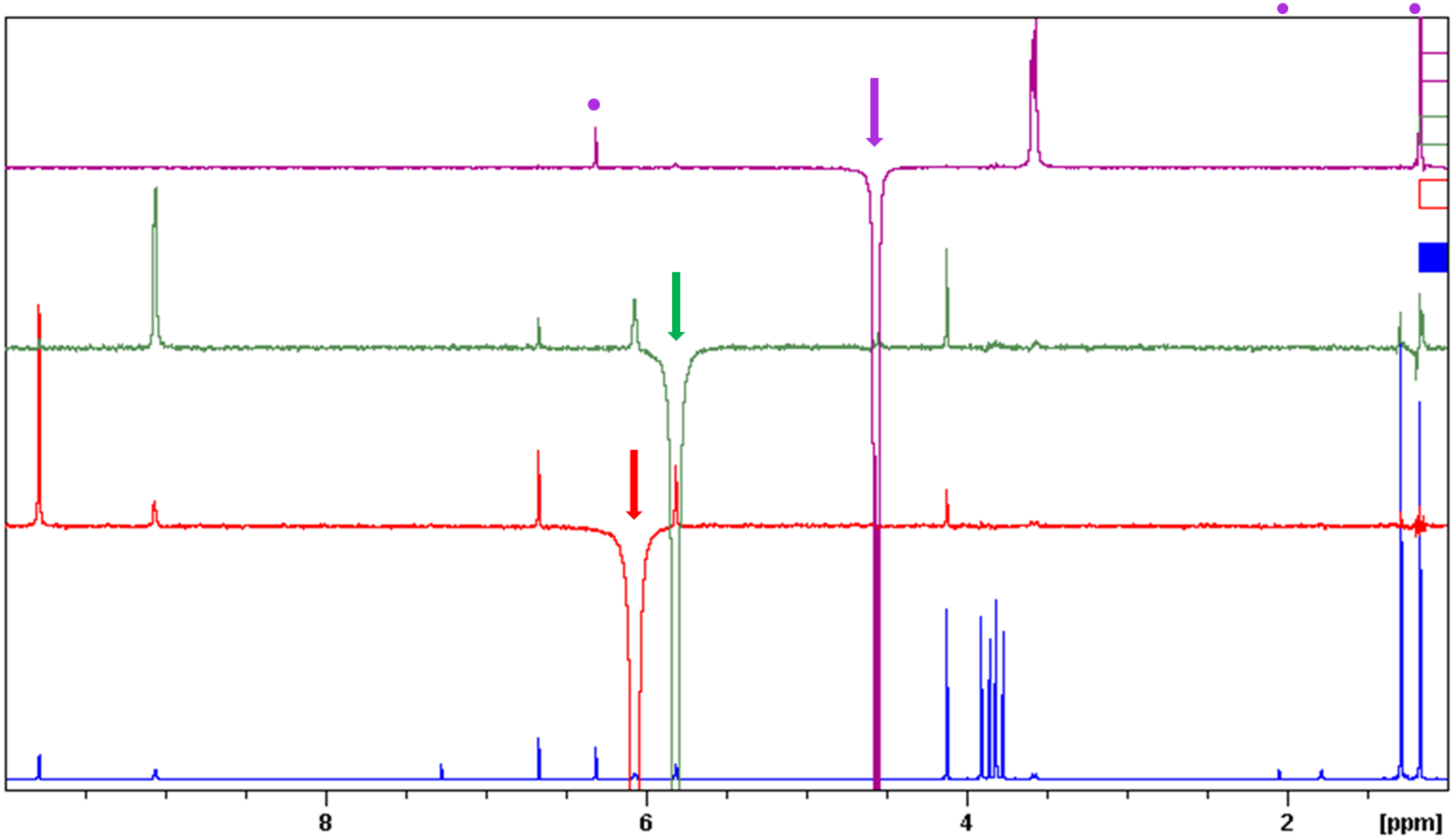

Compound **8b**, Steric proximities detected by NOESY

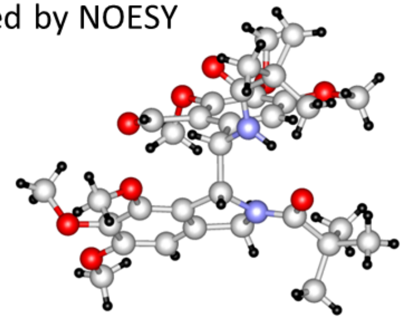

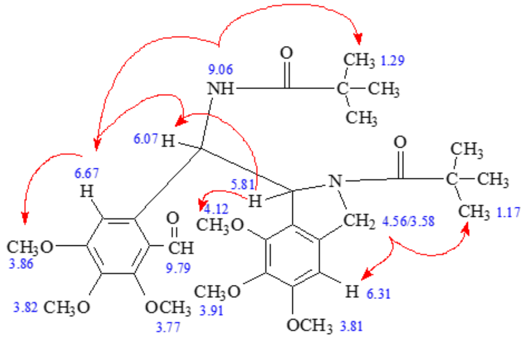

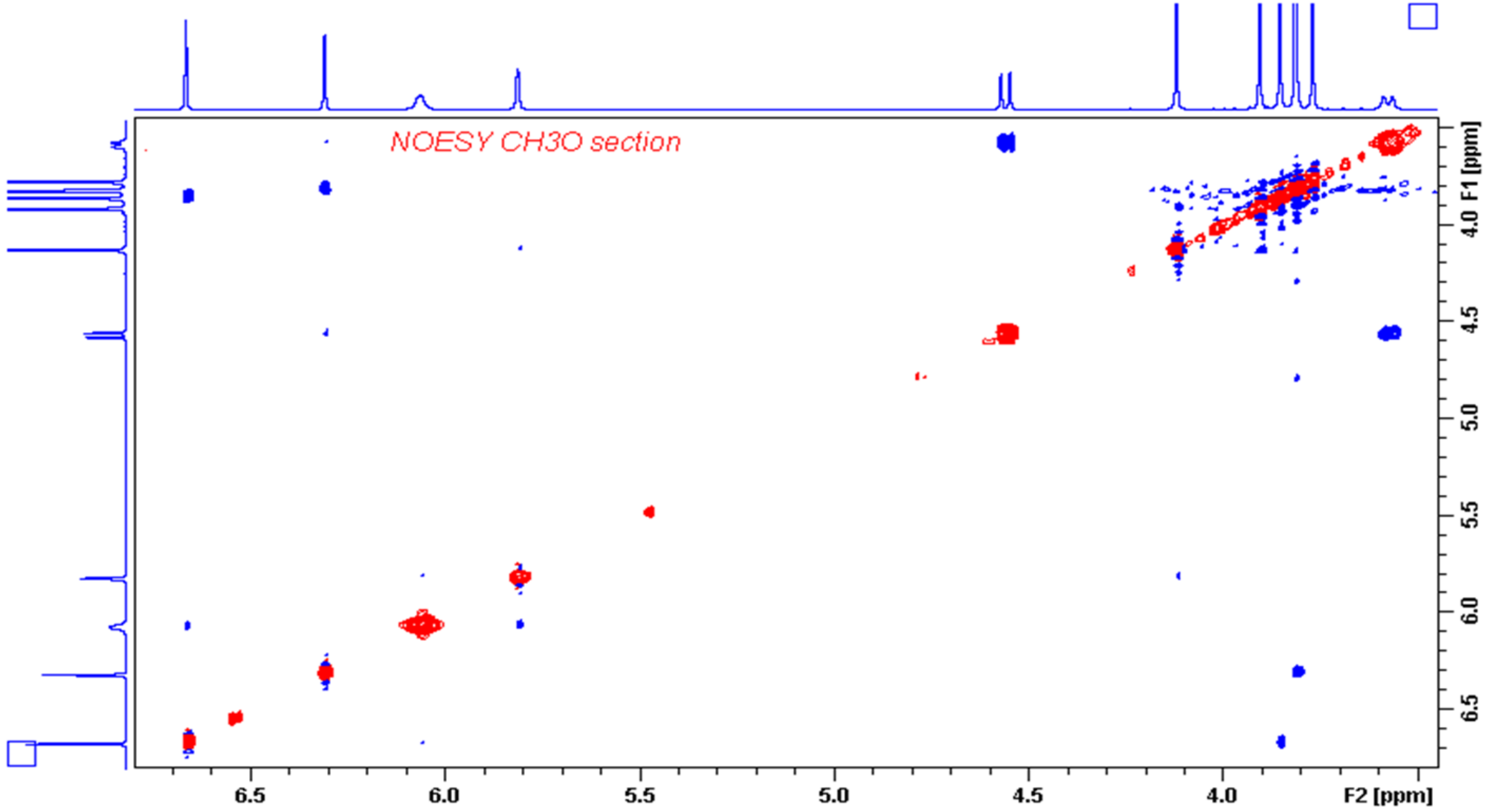

Compound **8b** , ^{13}C 150 MHz

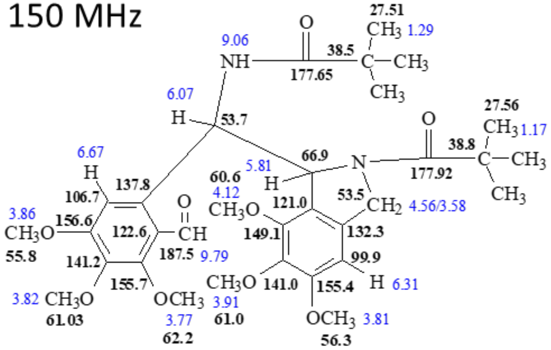

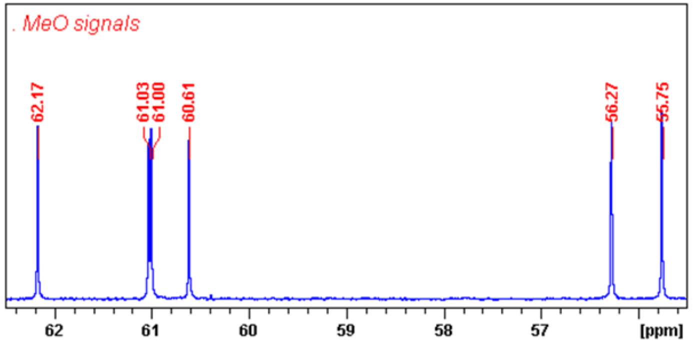

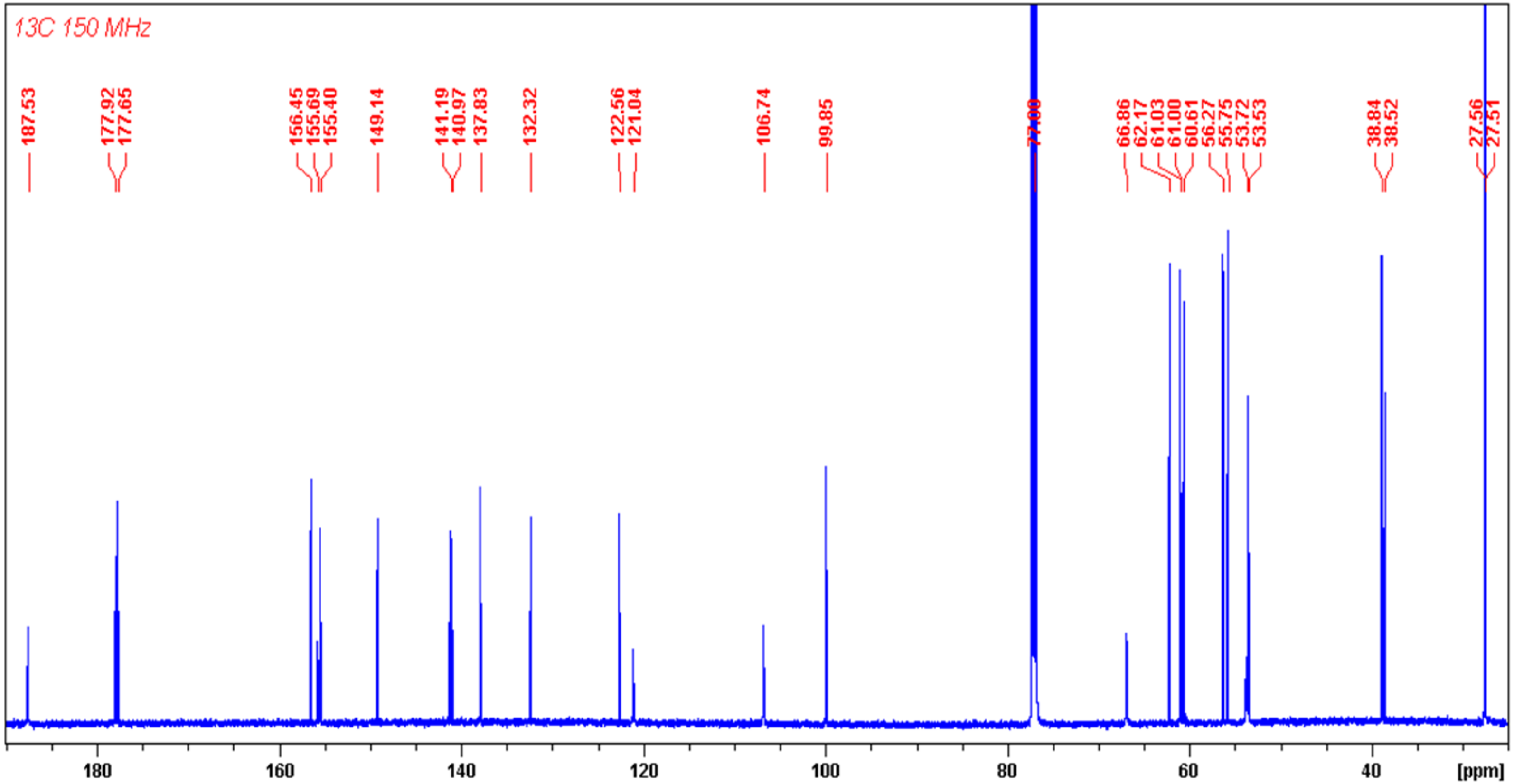

Compound **8b** , HSQC

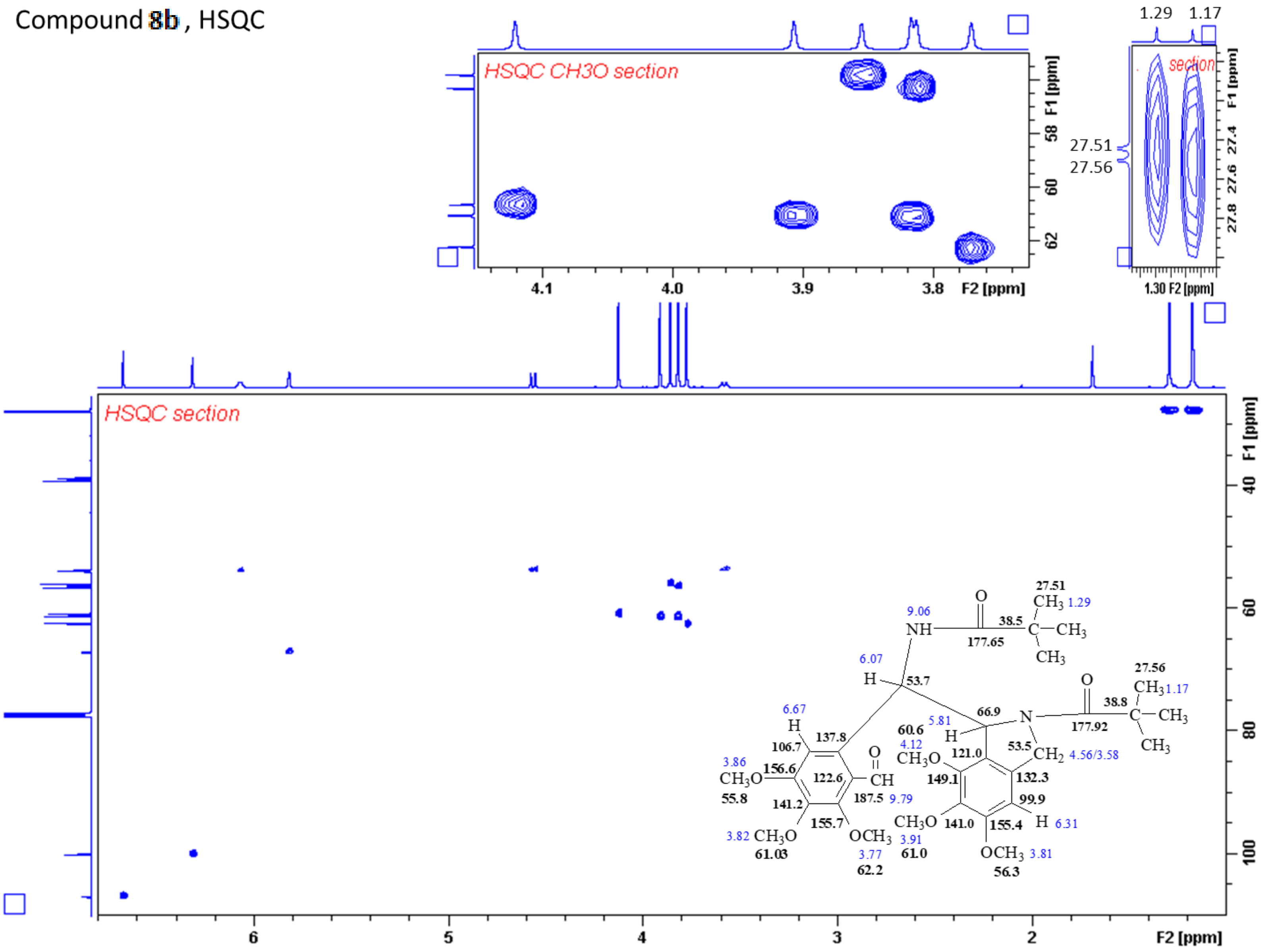

Compound **8b**, HMBC

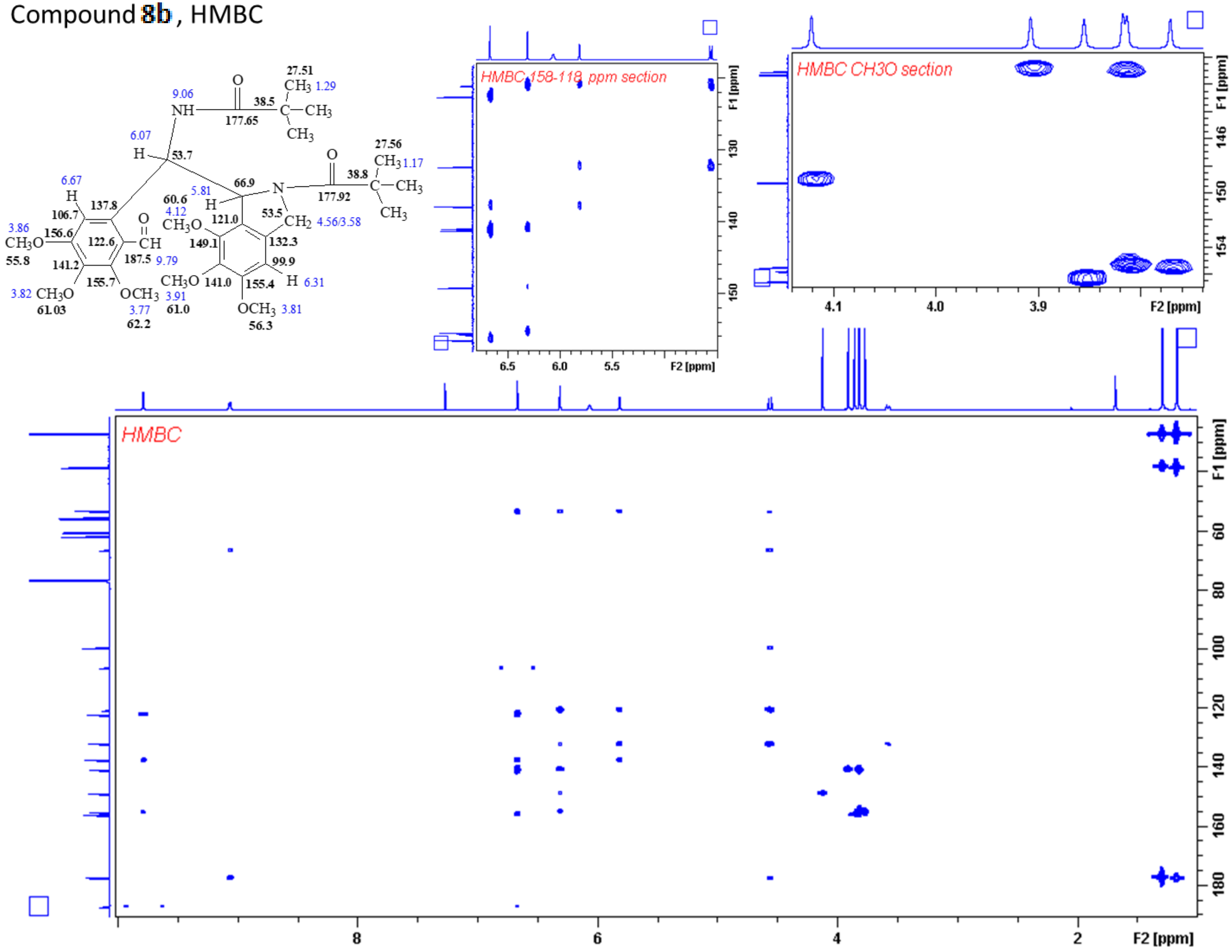

Compound **23a**, ^{1}H 600 MHz in $CDCl_3$

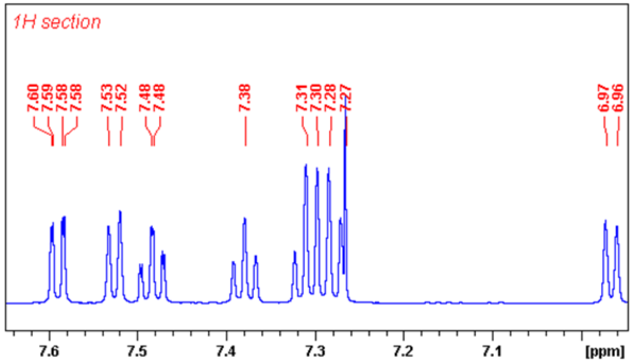

^{3}J(H-C-C-H) ~ 1 Hz

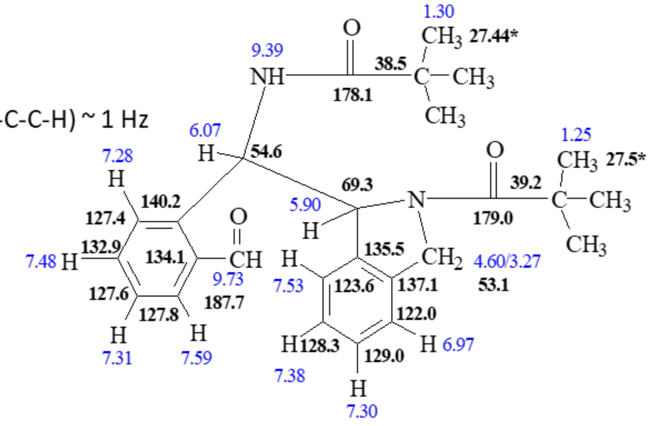

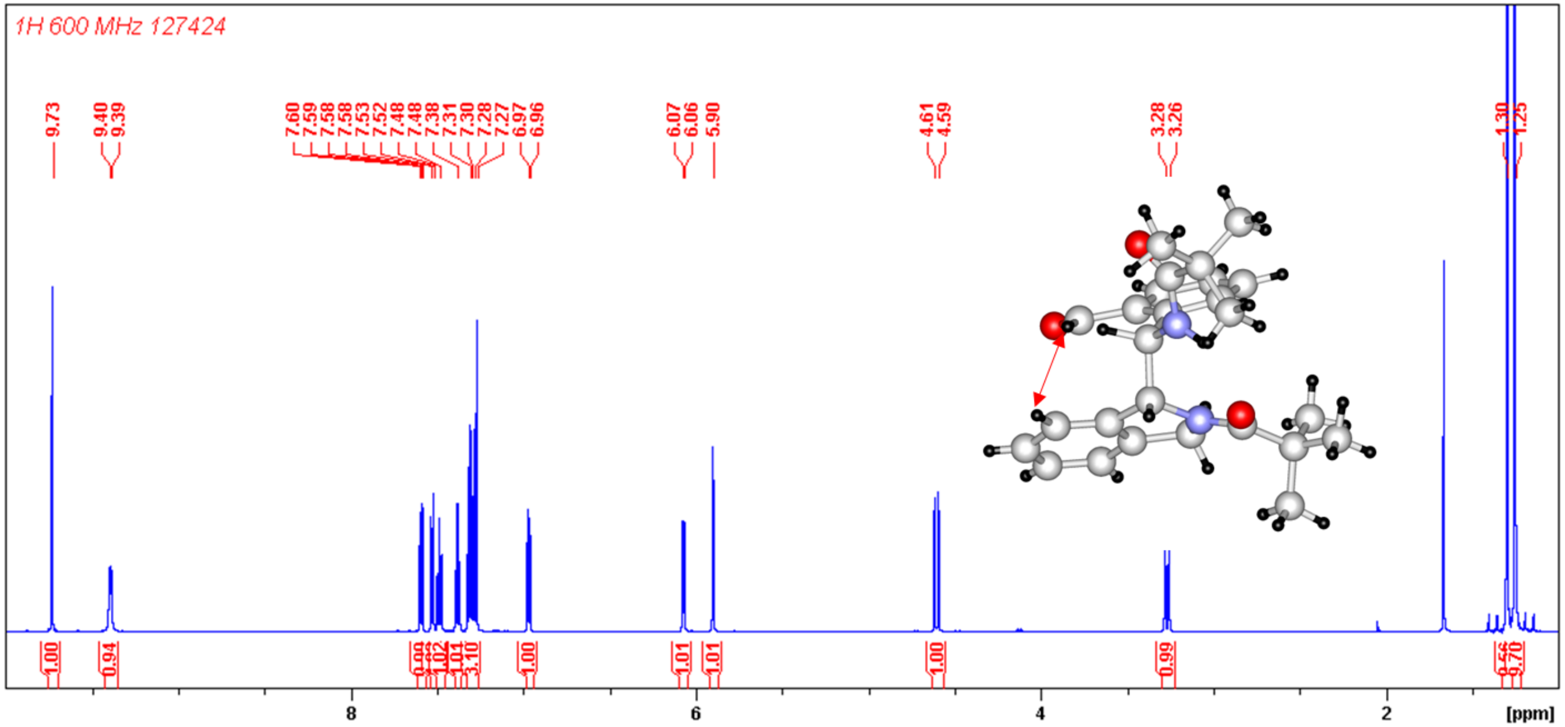

Compound **23a**, Steric proximities detected by NOESY

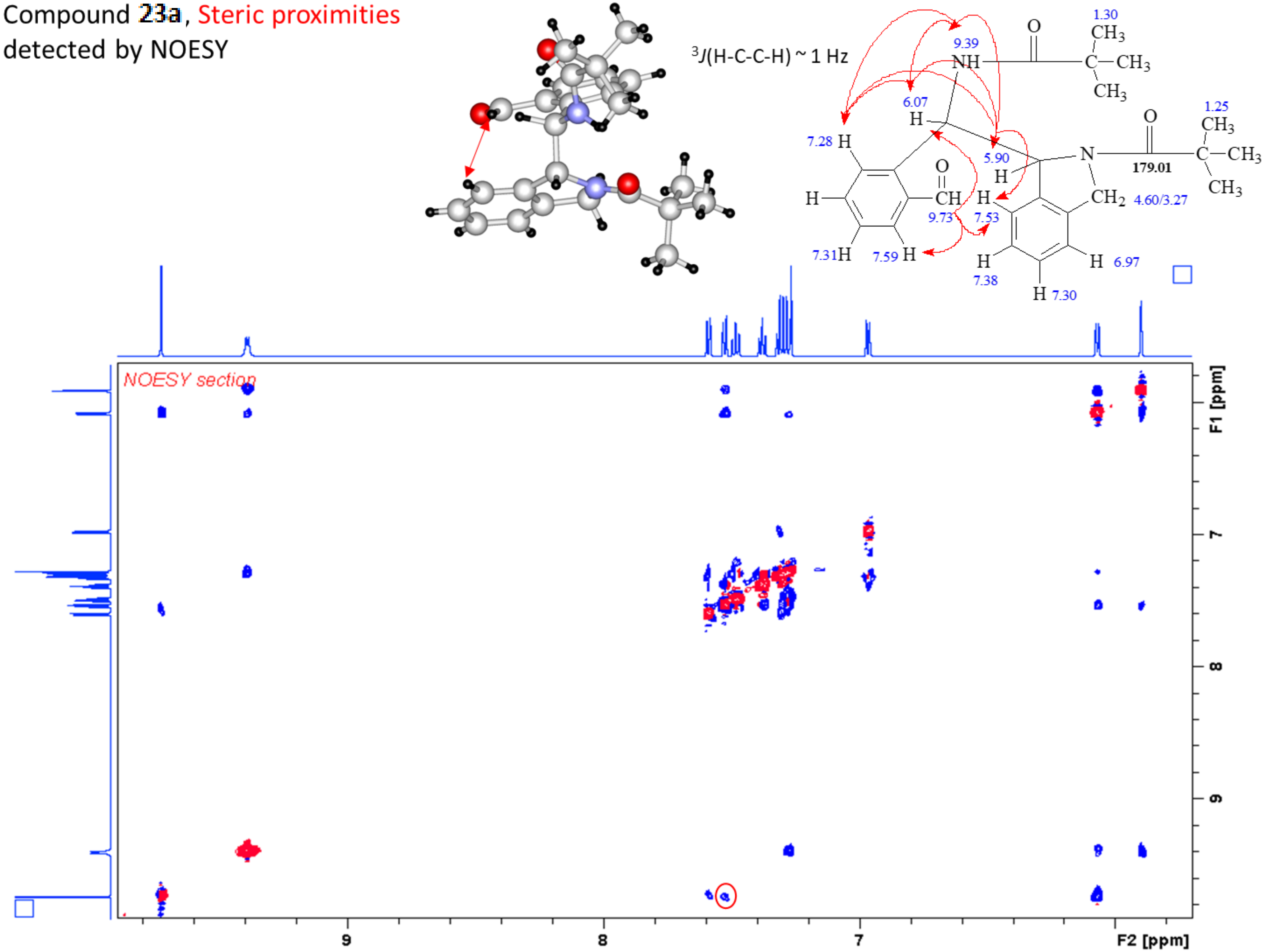

Compound **23a**, ^{13}C 150 MHz

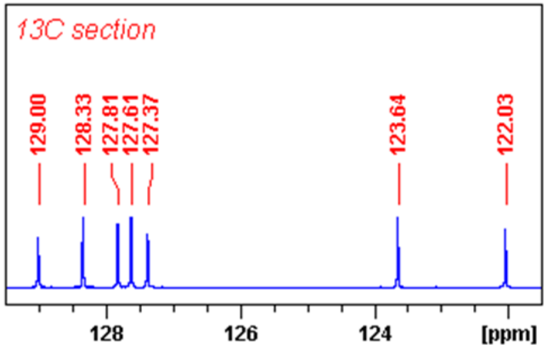

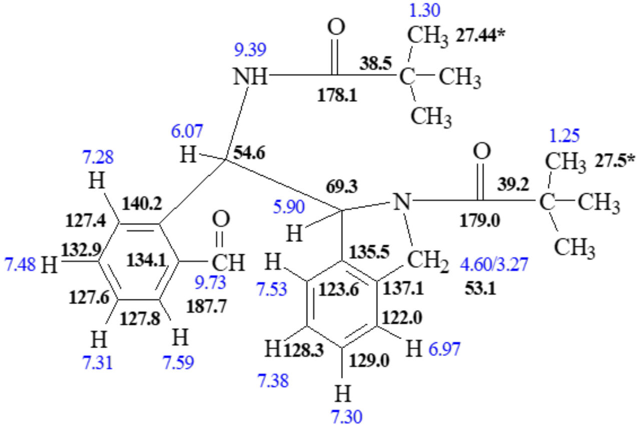

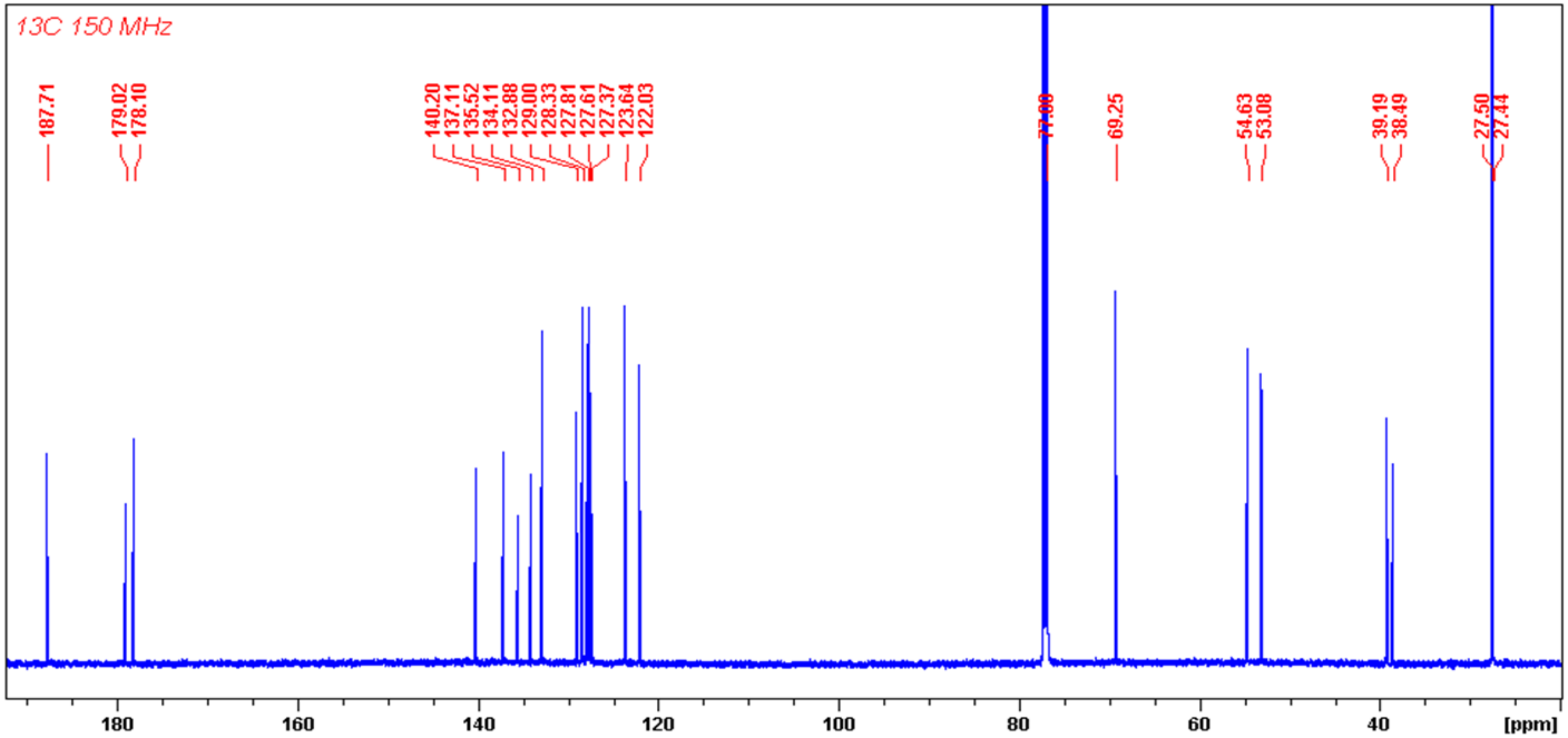

Compound **23a**, edHSQC

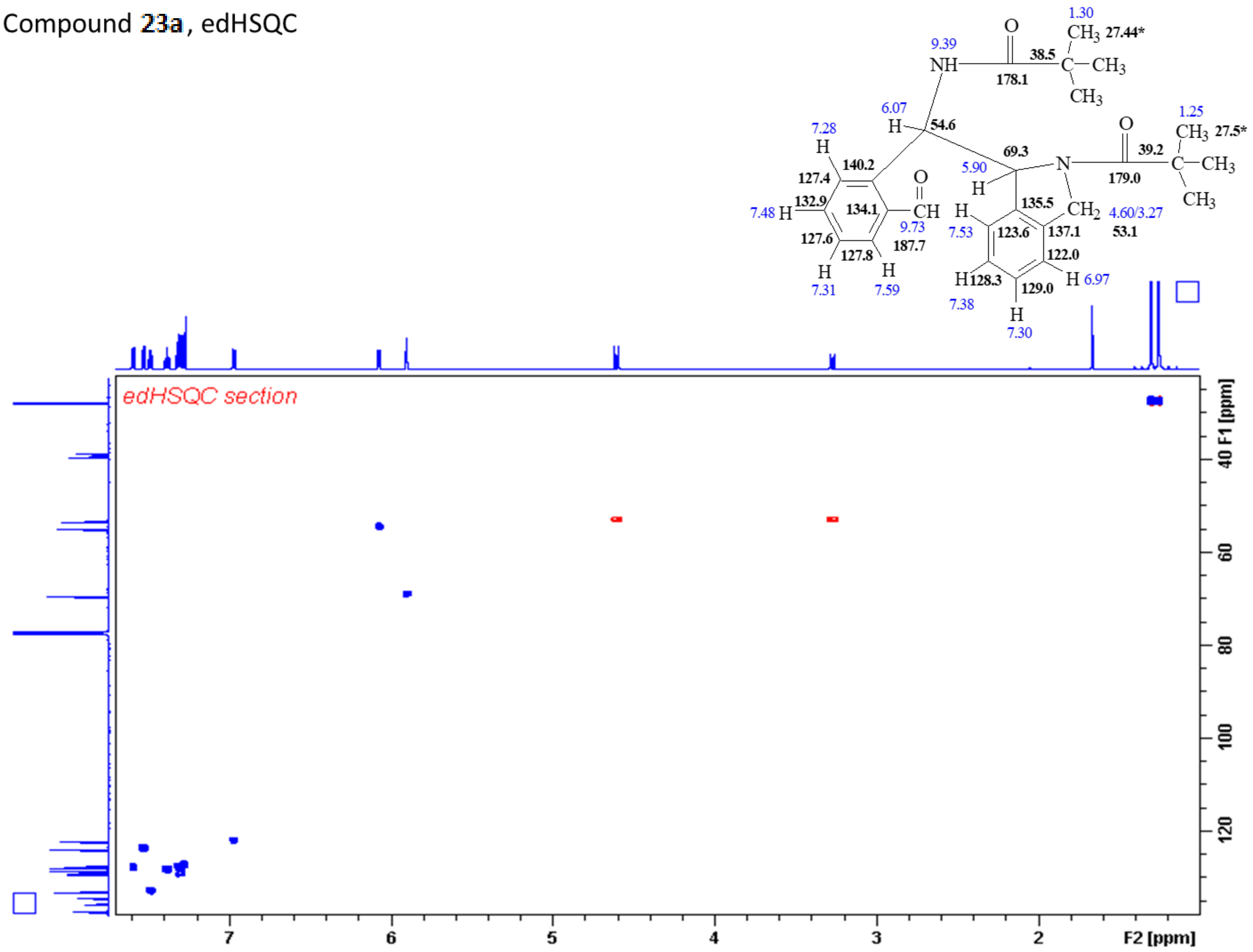

Compound **23a**, HMBC

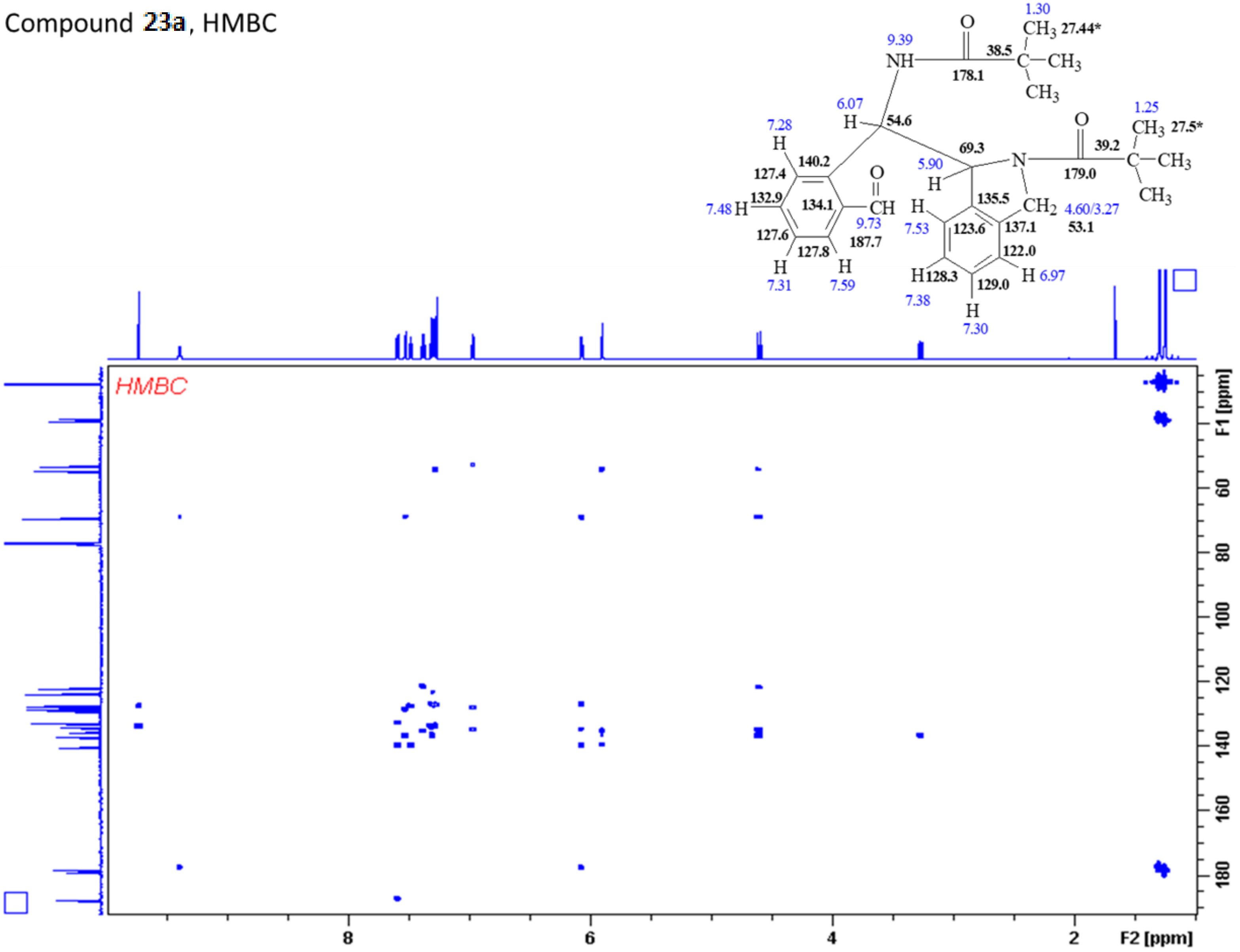

Compound **23a**, selective HSQC and selective HMBC

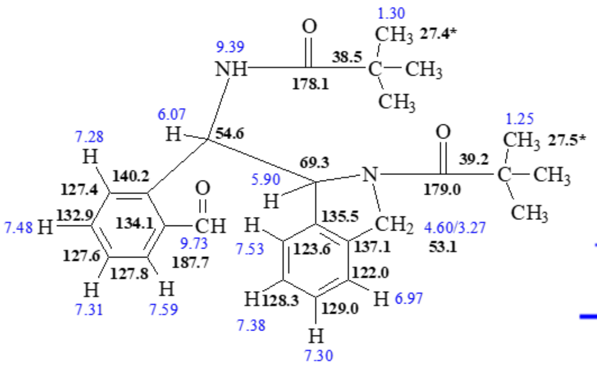

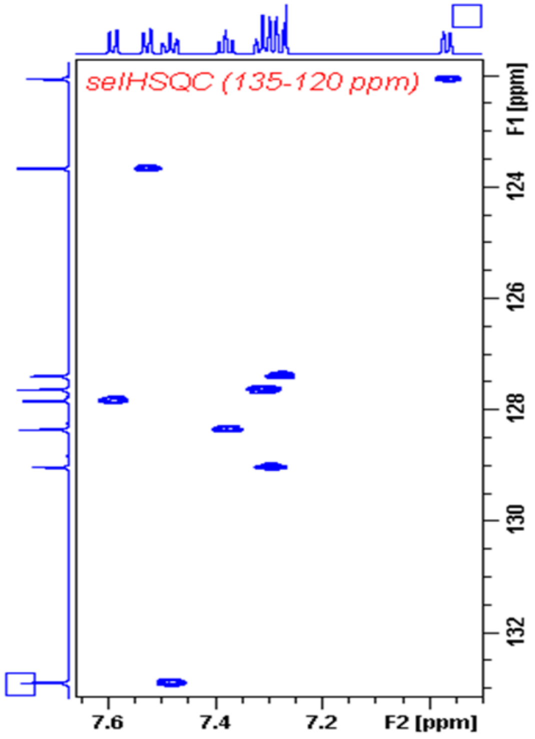

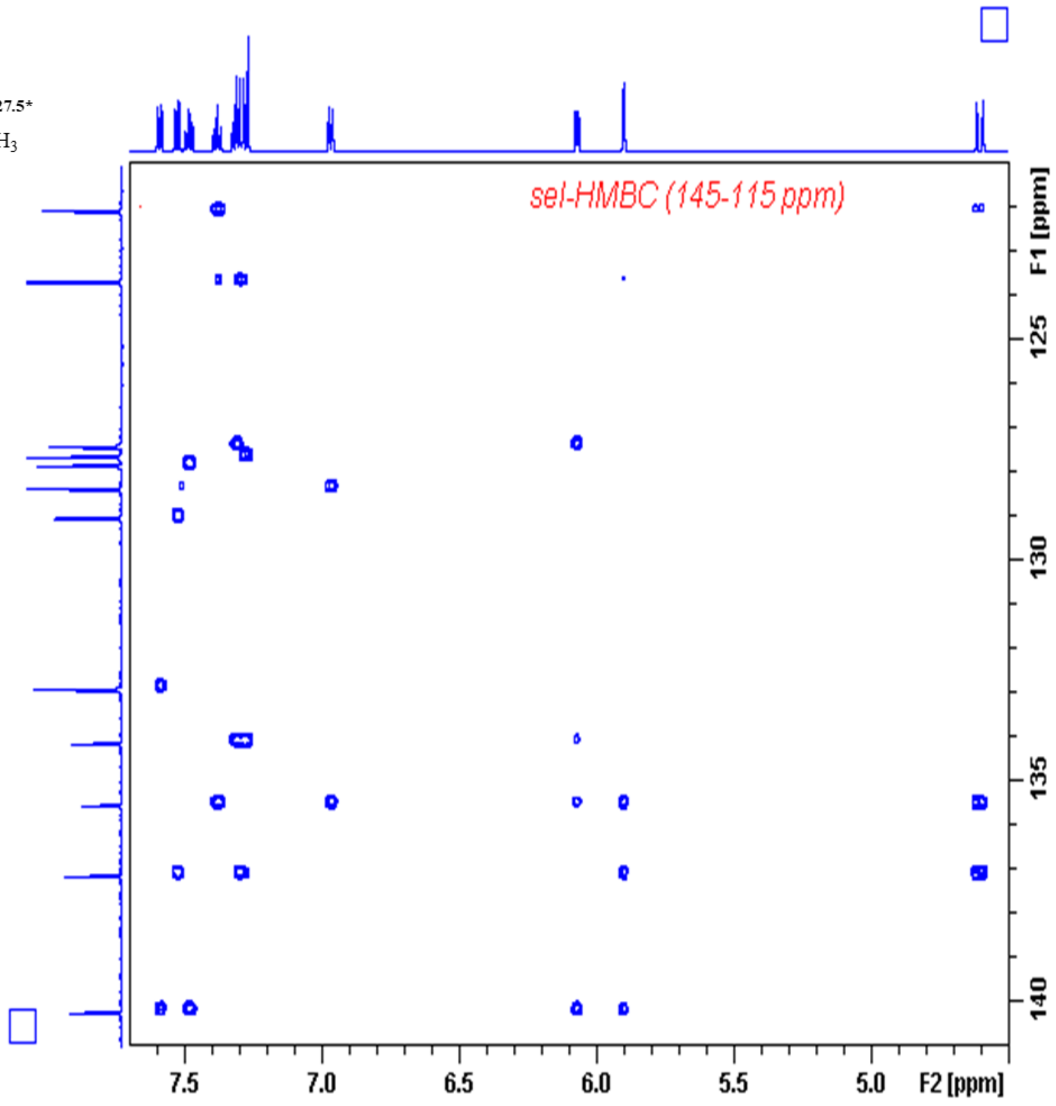

Compound **23b**, ^{1}H and DeptQ NMR (600/150 MHz in $CDCl_3$)

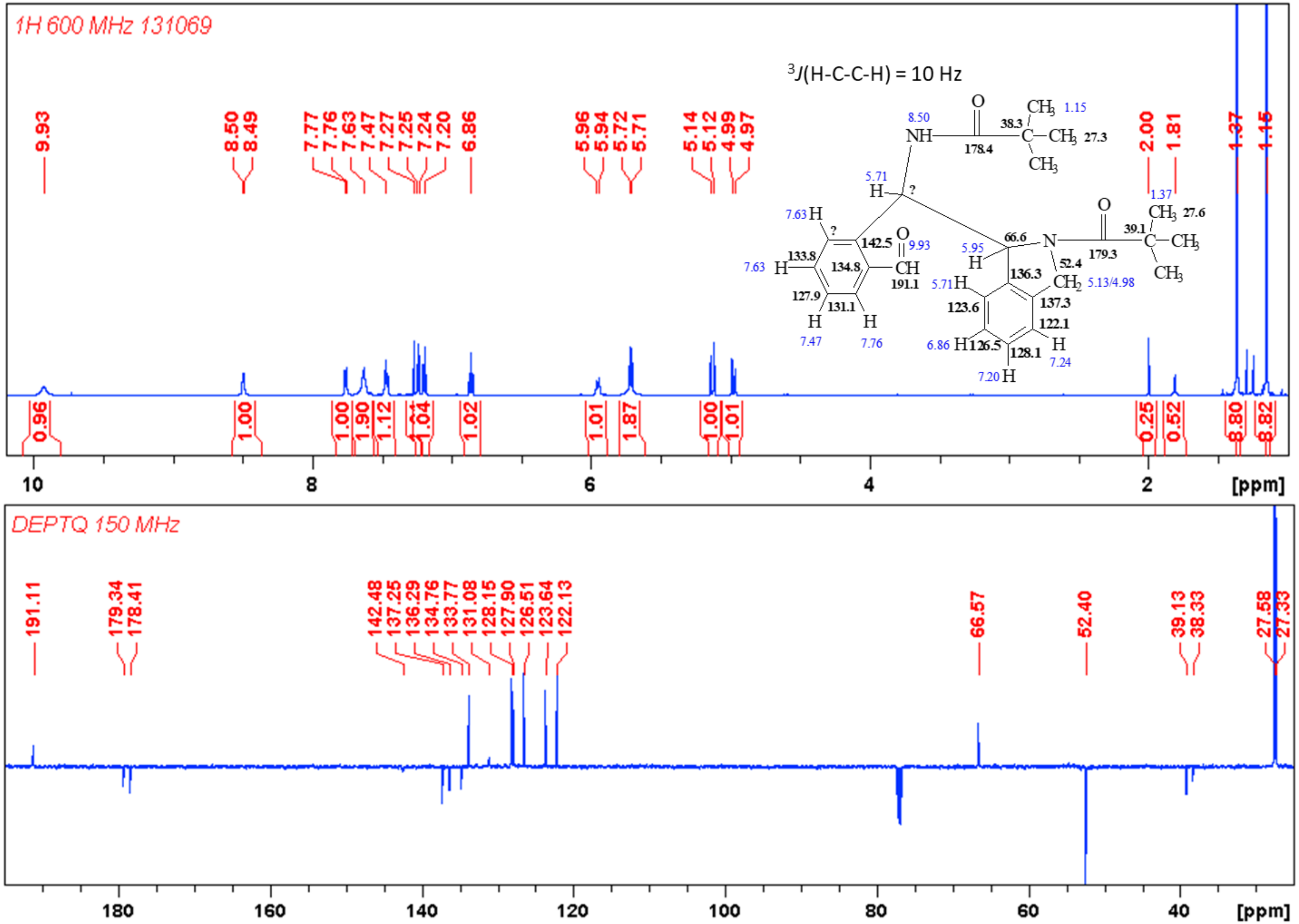

Compound **23b**, Identification of the three ^{1}H spin-systems by sel. TOCSY (τ_{mixing} = 60 ms) on signals 7.76, 7.24 and 8.50

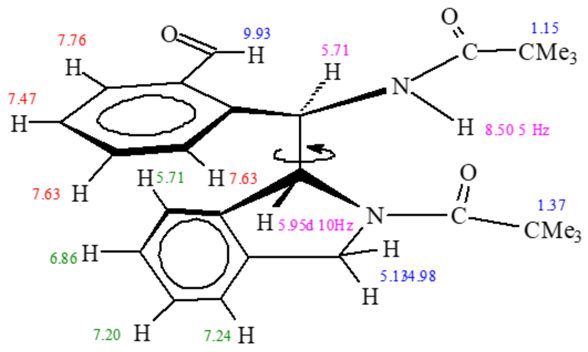

^{3}J(H-C-C-H) = 10 Hz

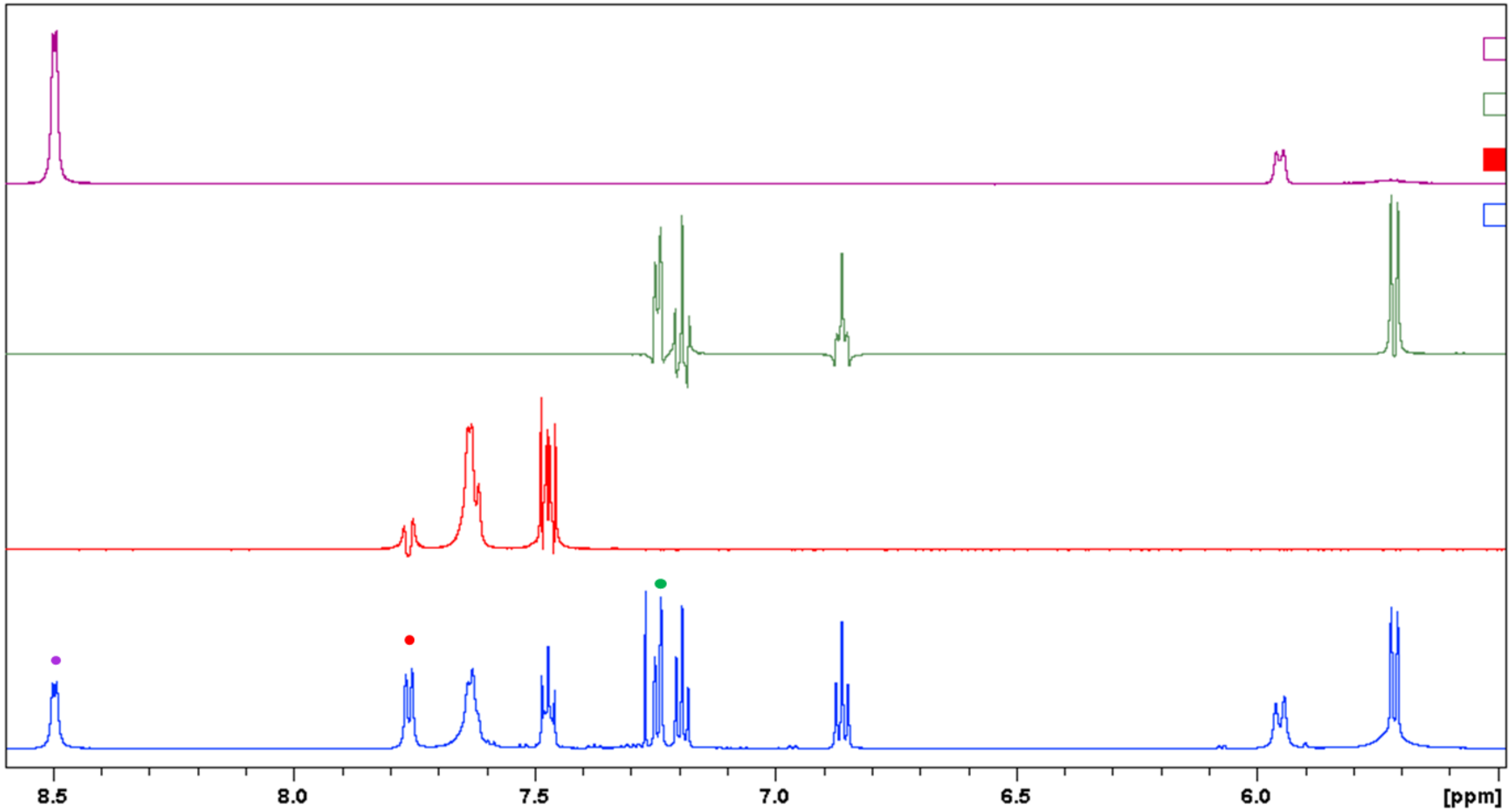

Compound **23b**, Steric proximities detected by NOESY

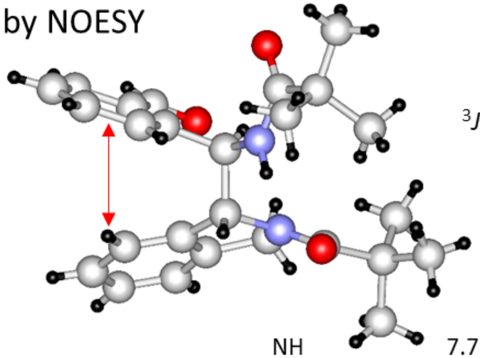

^3J(H-C-C-H) = 10 Hz

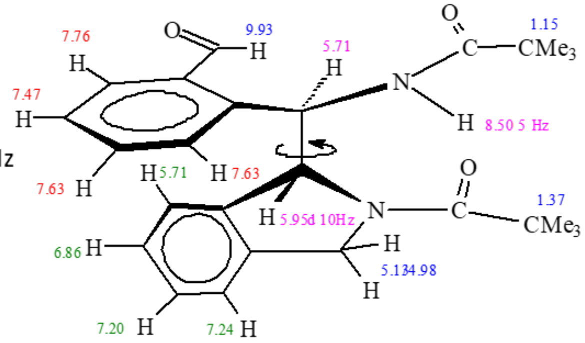

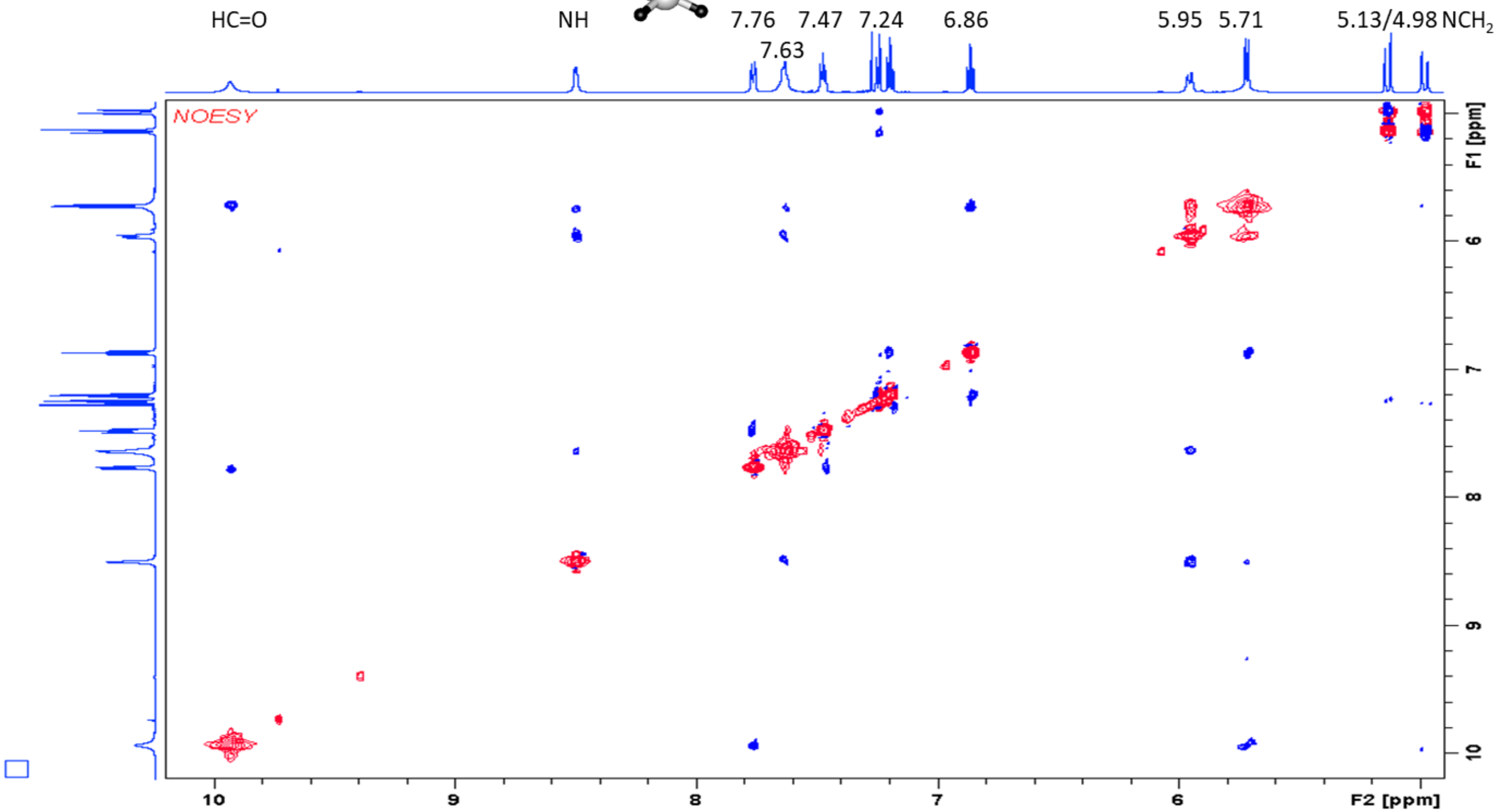

Compound **23b**, Steric proximities detected by selNOE on signals 9.93, 8.50, 7.76 and 5.95

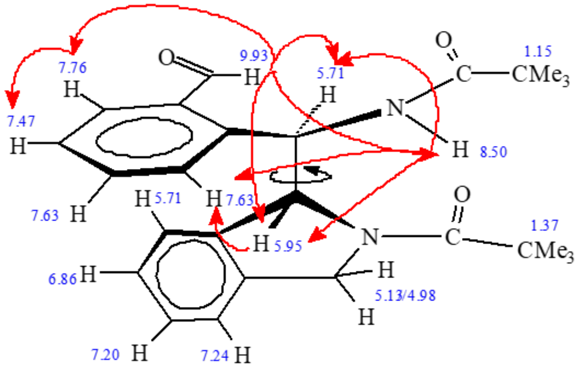

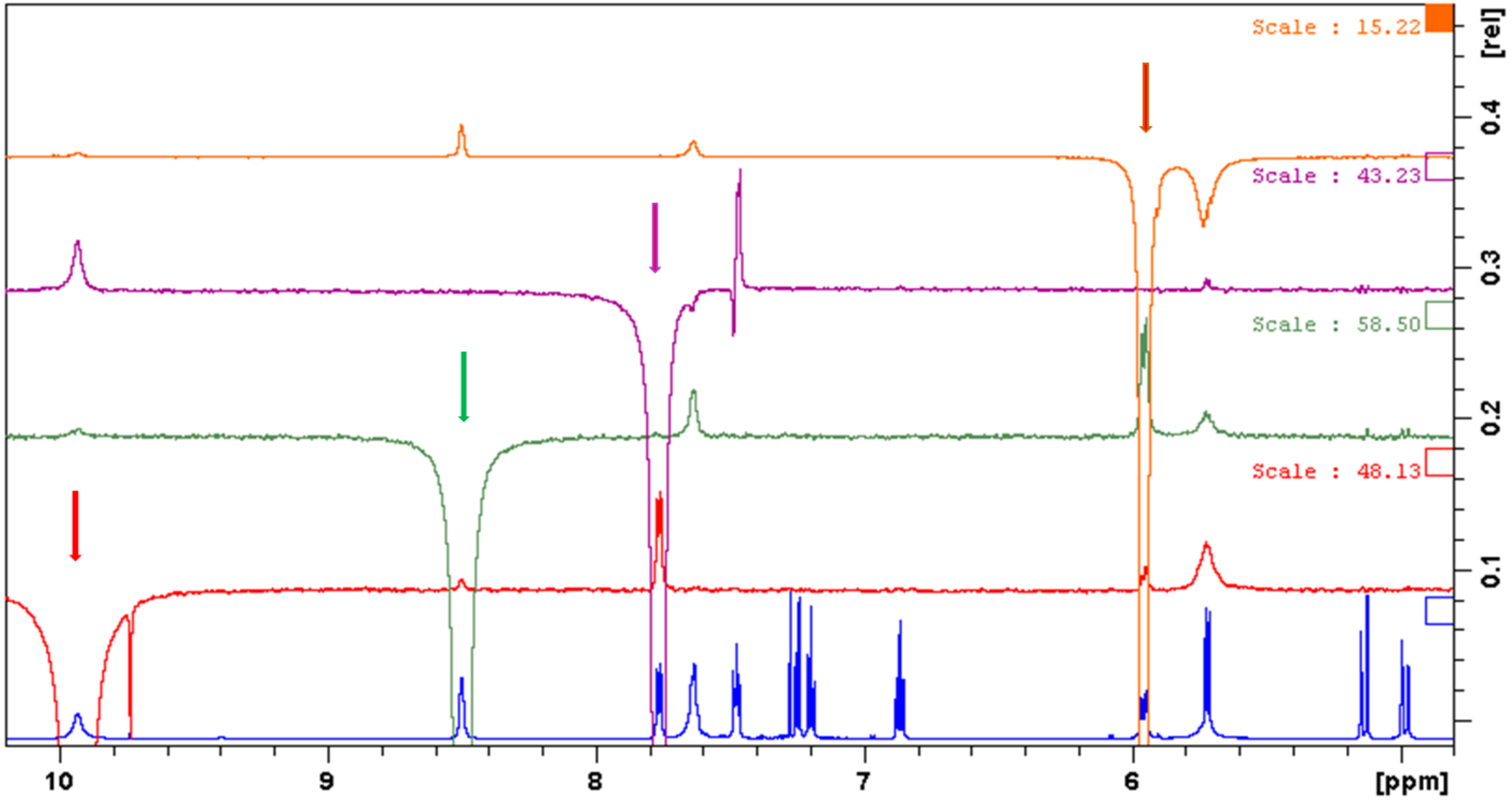

Compound **23b**, edHSQC

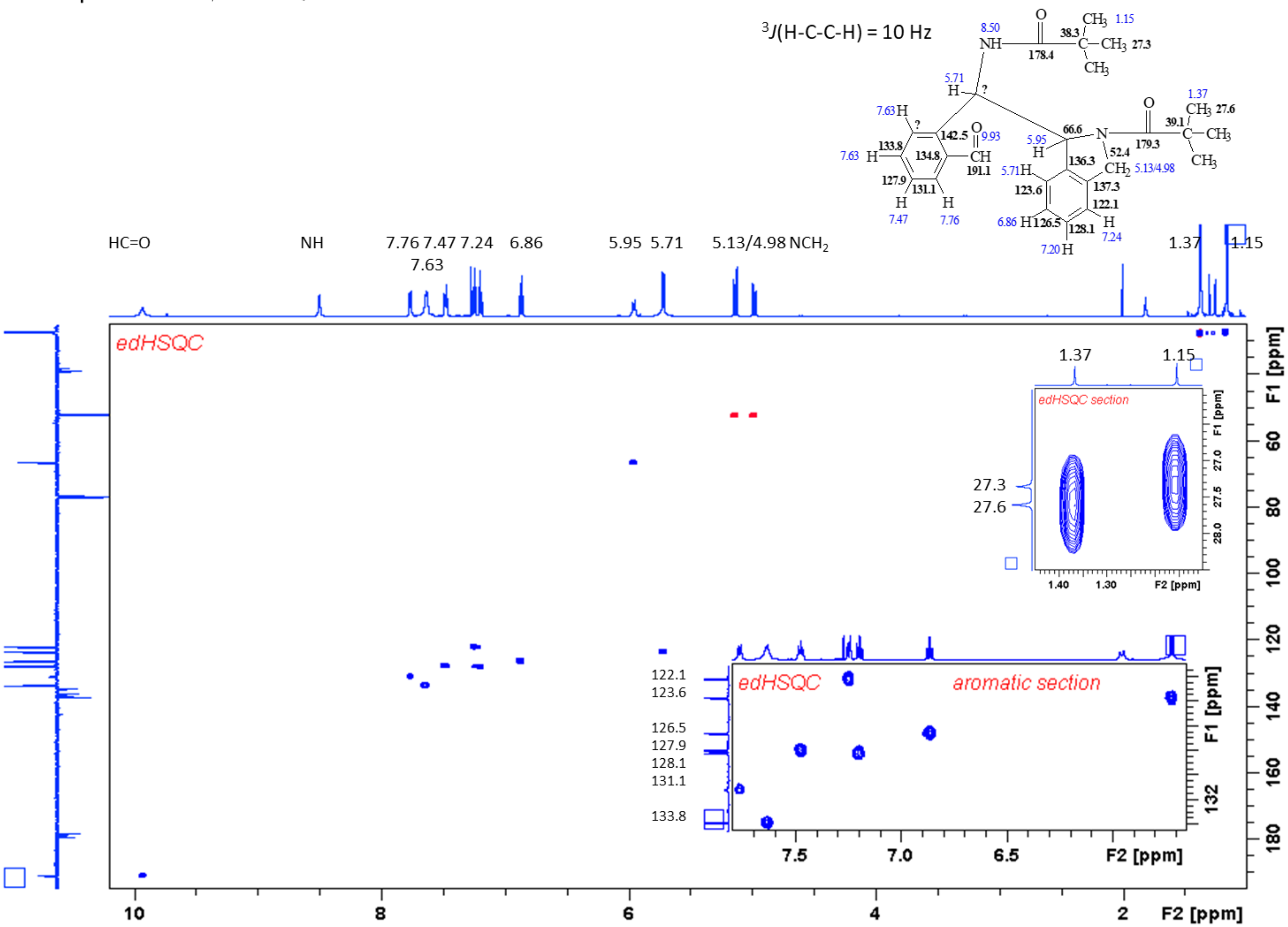

Compound **23b**, HMBC

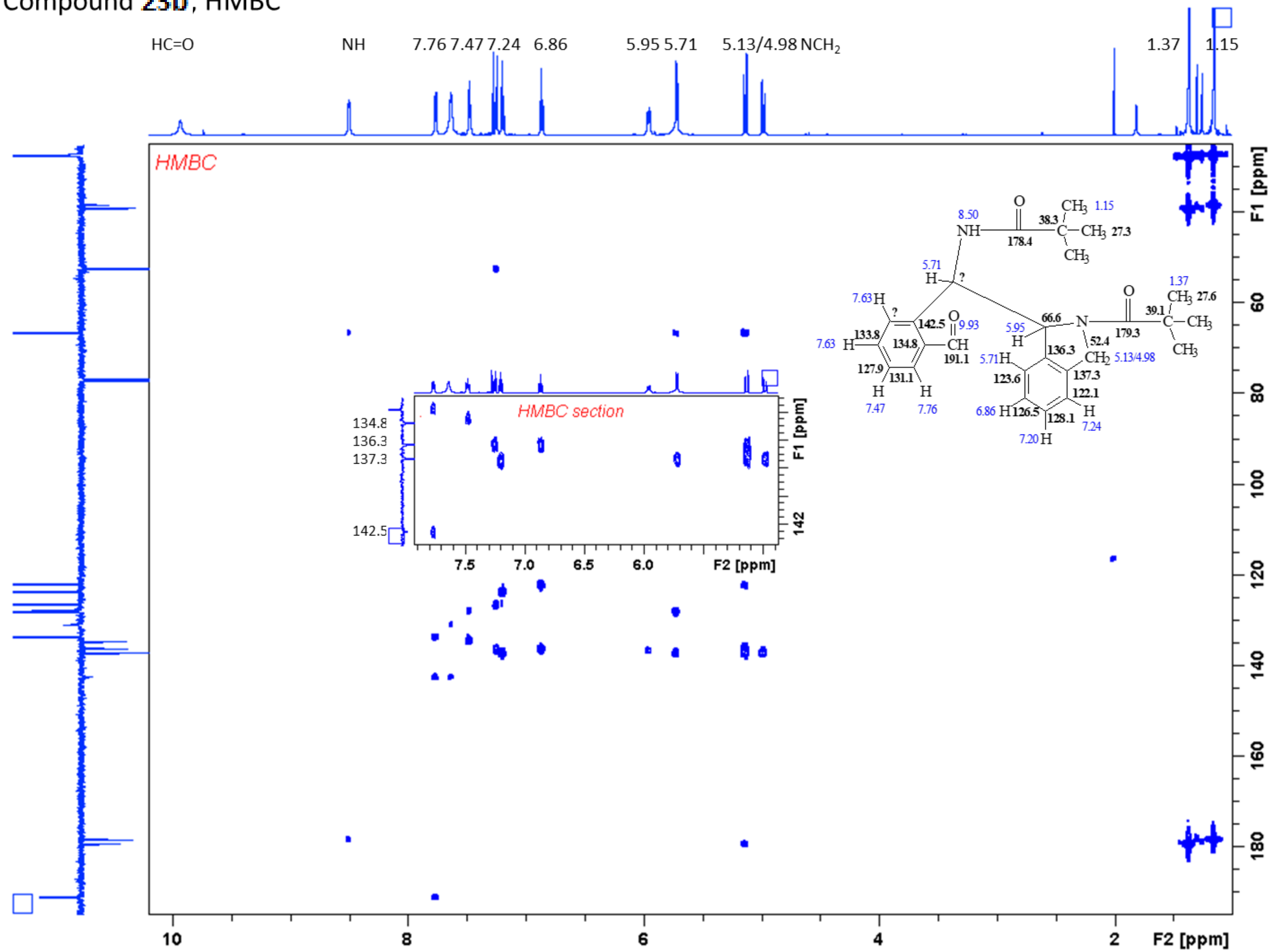

Compounds **23a,b** , HRMS

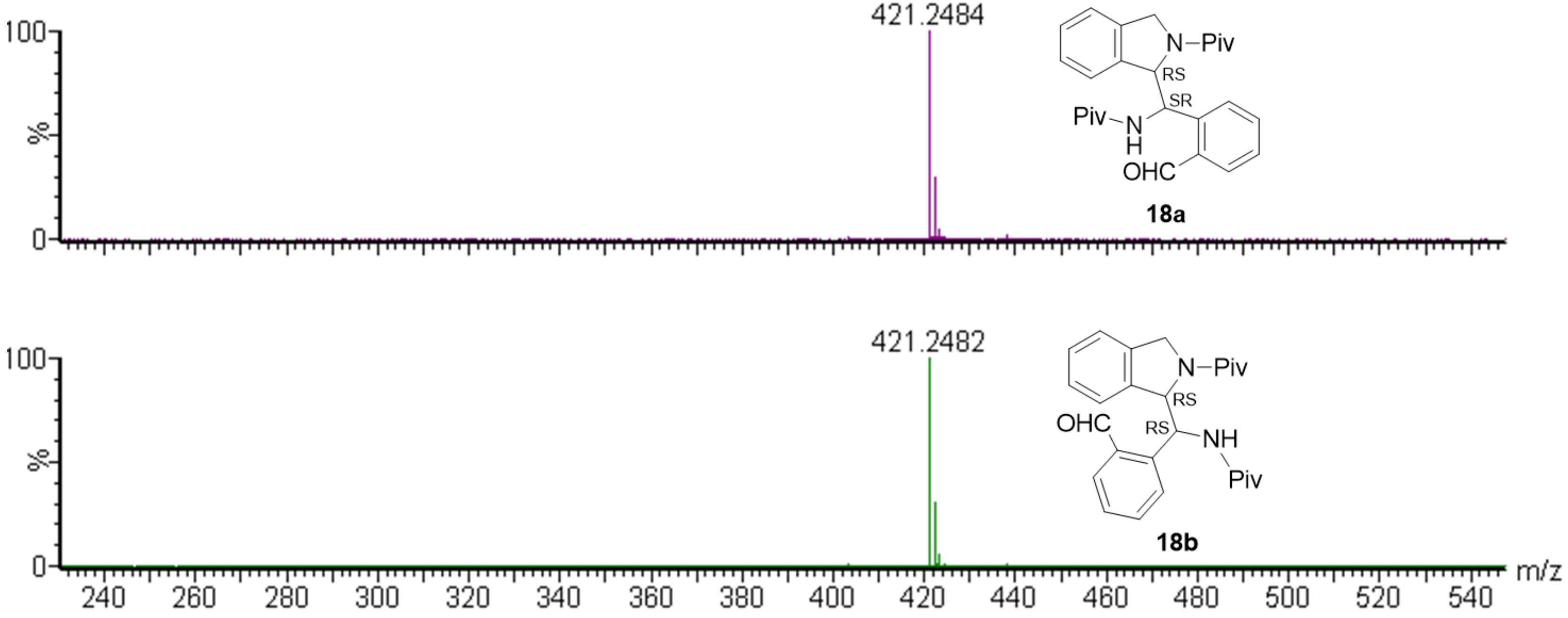

Table S1: Energy values obtained for the computation of the **1**→**2** transformation and the related dimerization. The E, ZPE, U, H and G values were computed using the B3LYP/6-31+G (d,p) method, E' values were computed by the B3LYP/6-311++G (3df,3pd) single point calculation, and G' values were calculated by the G' = E' + (G − E) equation.

ID	E	ZPE	U	H	G	S	E'	G'
a								
**1**	-1426.26669	-1425.93427	-1425.907403	-1425.906459	-1425.9977	192.034	-1426.704677	-1426.435687
**1TS**	-1426.24747	-1425.916145	-1425.891236	-1425.890291	-1425.973231	174.562	-1426.683436	-1426.409197
**9**	-1426.276005	-1425.941452	-1425.916006	-1425.915062	-1426.000767	180.382	-1426.712523	-1426.437285
**9TS**	-1426.252549	-1425.921169	-1425.895605	-1425.894661	-1425.980752	181.195	-1426.68833	-1426.416533
**11**	-1426.256578	-1425.926497	-1425.898976	-1425.898032	-1425.989697	192.926	-1426.693428	-1426.426547
**11TS**	-1349.787755	-1349.486807	-1349.463134	-1349.46219	-1349.544104	172.402	-1350.197118	-1349.953467
**4**	-1349.802293	-1349.496923	-1349.472355	-1349.47141	-1349.556591	179.277	-1350.214564	-1349.968861
**4TS**	-1349.793564	-1349.492722	-1349.469852	-1349.468908	-1349.547734	165.903	-1350.203005	-1349.957175
**12**	-1426.261541	-1425.931244	-1425.90368	-1425.902736	-1425.99497	194.124	-1426.698692	-1426.432121
**12TS**	-1426.25562	-1425.924162	-1425.898723	-1425.897779	-1425.982917	179.19	-1426.691596	-1426.418893
**14**	-1426.277314	-1425.942555	-1425.917233	-1425.916289	-1426.001081	178.459	-1426.713832	-1426.437599
**14TS**	-1426.249847	-1425.917803	-1425.89291	-1425.891965	-1425.975085	174.94	-1426.685925	-1426.411163
**2**	-1426.269665	-1425.937093	-1425.910288	-1425.909344	-1426.001157	193.236	-1426.70811	-1426.439602
b								
**1**	-1352.260894	-1351.911306	-1351.884218	-1351.883274	-1351.97459	192.191	-1352.673878	-1352.387575
**1TS**	-1352.243732	-1351.895631	-1351.870355	-1351.869411	-1351.953317	176.596	-1352.654533	-1352.364118
**9**	-1352.271787	-1351.920245	-1351.894545	-1351.893601	-1351.979149	180.051	-1352.6828	-1352.390162
**9TS**	-1352.247867	-1351.89986	-1351.873899	-1351.872955	-1351.959013	181.125	-1352.658674	-1352.36982
**11**	-1352.251237	-1351.903794	-1351.876176	-1351.875232	-1351.966305	191.68	-1352.663103	-1352.37817
**11TS**	-1275.784149	-1275.466402	-1275.442338	-1275.441394	-1275.523973	173.802	-1276.168078	-1275.907902
**4**	-1275.79773	-1275.475504	-1275.450596	-1275.449652	-1275.534941	179.507	-1276.184618	-1275.92183
**4TS**	-1275.790423	-1275.472618	-1275.448547	-1275.447603	-1275.530027	173.477	-1276.174507	-1275.914111
**12**	-1352.258066	-1351.911004	-1351.883941	-1351.882996	-1351.972292	187.939	-1352.67001	-1352.384236
**12TS**	-1352.251757	-1351.903336	-1351.877614	-1351.876669	-1351.962063	179.726	-1352.662444	-1352.37275
**14**	-1352.272843	-1351.921278	-1351.895588	-1351.894644	-1351.980071	179.795	-1352.683798	-1352.391026
**14TS**	-1352.245607	-1351.896568	-1351.871383	-1351.870439	-1351.95422	176.333	-1352.656402	-1352.365015
**2**	-1352.265695	-1351.916141	-1351.889113	-1351.888168	-1351.979184	191.558	-1352.678652	-1352.392141
c								
**1**	-1352.258805	-1351.909048	-1351.882172	-1351.881227	-1351.970945	188.826	-1352.671215	-1352.383354
**1TS**	-1352.242838	-1351.894408	-1351.869024	-1351.86808	-1351.953112	178.966	-1352.65357	-1352.363844
**9**	-1352.272232	-1351.920682	-1351.894942	-1351.893998	-1351.979665	180.302	-1352.683202	-1352.390635
**9TS**	-1352.250611	-1351.902186	-1351.876316	-1351.875372	-1351.961525	181.325	-1352.661171	-1352.372085
**11**	-1352.255883	-1351.908679	-1351.880787	-1351.879843	-1351.973047	196.164	-1352.667509	-1352.384673
**11TS**	-1275.788932	-1275.470781	-1275.446745	-1275.445801	-1275.528796	174.679	-1276.17267	-1275.912534
**4**	-1275.802444	-1275.479779	-1275.454956	-1275.454012	-1275.538893	178.648	-1276.189109	-1275.925558
**4TS**	-1275.788144	-1275.470394	-1275.446199	-1275.445254	-1275.529205	176.688	-1276.171945	-1275.913006
**12**	-1352.252292	-1351.904893	-1351.877186	-1351.876242	-1351.96721	191.458	-1352.663874	-1352.378792
**12TS**	-1352.249026	-1351.900654	-1351.874744	-1351.8738	-1351.959878	181.167	-1352.659555	-1352.370407
**14**	-1352.273042	-1351.92143	-1351.895629	-1351.894685	-1351.98094	181.539	-1352.683912	-1352.39181
**14TS**	-1352.244694	-1351.896637	-1351.871251	-1351.870307	-1351.954967	178.182	-1352.655353	-1352.365627
**2**	-1352.25733	-1351.907678	-1351.880751	-1351.879807	-1351.970041	189.915	-1352.669927	-1352.382637
d								
**1**	-1581.305223	-1580.891545	-1580.858921	-1580.857977	-1580.961049	216.933	-1581.790718	-1581.446544
**1TS**	-1581.286783	-1580.873722	-1580.842749	-1580.841805	-1580.938807	204.158	-1581.770778	-1581.422802
**9**	-1581.316012	-1580.900357	-1580.868835	-1580.867891	-1580.966914	208.41	-1581.800112	-1581.451013
**9TS**	-1581.297523	-1580.885029	-1580.853443	-1580.852498	-1580.951425	208.208	-1581.781224	-1581.435126
**11**	-1581.305886	-1580.89485	-1580.860893	-1580.859949	-1580.966488	224.231	-1581.791021	-1581.451623
**11TS**	-1504.83716	-1504.454784	-1504.425079	-1504.424134	-1504.519371	200.443	-1505.294112	-1504.976323
**4**	-1504.847876	-1504.461363	-1504.430758	-1504.429814	-1504.527922	206.485	-1505.30749	-1504.987536
**4TS**	-1504.834261	-1504.452457	-1504.422599	-1504.421655	-1504.516874	200.406	-1505.291297	-1504.97391
**12**	-1581.300898	-1580.889351	-1580.855808	-1580.854864	-1580.958865	218.888	-1581.78608	-1581.444047
**12TS**	-1581.29543	-1580.883316	-1580.851537	-1580.850593	-1580.950288	209.825	-1581.77907	-1581.433928
**14**	-1581.318135	-1580.90245	-1580.870901	-1580.869957	-1580.968747	207.923	-1581.801976	-1581.452588
**14TS**	-1581.290226	-1580.877915	-1580.84687	-1580.845926	-1580.942502	203.261	-1581.773759	-1581.426035
**2**	-1581.307268	-1580.893602	-1580.860741	-1580.859797	-1580.964324	219.996	-1581.792903	-1581.44996
**4e-22b TS**	-1269.331082	-1268.813514	-1268.785529	-1268.784584	-1268.869856	179.47	-1269.675906	-1269.21468
**22b**	-1269.348187	-1268.827684	-1268.79979	-1268.798846	-1268.88377	178.738	-1269.692153	-1269.227736
**4e-22a TS**	-1269.333203	-1268.816232	-1268.787875	-1268.786931	-1268.874312	183.908	-1269.672565	-1269.213674

**22a2**	-1269.357303	-1268.837493	-1268.809256	-1268.808311	-1268.895185	182.841	-1269.695287	-1269.233169
**mon1**	-634.4374458	-634.186565	-634.17275	-634.171806	-634.226801	115.749	-634.619519	-634.4093829
**mon2**	-634.8715921	-634.607615	-634.593566	-634.592622	-634.648209	116.992	-635.063098	-634.8382596

Table S2: Negative frequencies of the computed transition states

ID	E
**a**	
**1TS**	-269.79
**9TS**	-219.34
**11TS**	-1307.83
**4TS**	-1277.70
**12TS**	-239.91
**14TS**	-213.79
**b**	
**1TS**	-267.36
**9TS**	-206.16
**11TS**	-1276.29
**4TS**	-1253.56
**12TS**	-244.13
**14TS**	-215.36
**c**	
**1TS**	-220.56
**9TS**	-227.11
**11TS**	-1282.42
**4TS**	-1281.18
**12TS**	-205.34
**14TS**	-272.71
**d**	
**1TS**	-198.99
**9TS**	-256.17
**11TS**	-1238.77
**4TS**	-1306.62
**12TS**	-228.71
**14TS**	-271.66
**e**	
**4e-22b TS**	-300.07
**4e-22a TS**	-294.29

Coordinates of the computed structures

a

1

C	-0.70029100	-2.02962100	0.57127100
C	0.22349200	-1.70597800	-0.45787900
C	1.53201900	-2.21055000	-0.37960200
C	1.91445000	-3.03830200	0.67625400
C	1.01978600	-3.34870600	1.69461100
C	-0.28027900	-2.82868100	1.63727100
C	-0.19470900	-0.89707400	-1.61018200
H	-1.25344300	-0.94817500	-1.90289300
N	-2.11373700	-0.02326700	0.51758500
C	-3.12277300	0.89000400	0.32740200
O	-2.85975500	2.08366600	0.52026900
C	-4.52954300	0.46121000	-0.16471300
C	-5.43080100	1.71226800	-0.17882100
H	-5.54270900	2.13340700	0.82423600
H	-5.02463900	2.49261700	-0.82639200
H	-6.42320800	1.43402800	-0.54843100
C	-5.17835800	-0.58456500	0.77297600
H	-6.22648000	-0.71733900	0.48481500
H	-4.70506000	-1.56631700	0.72128800
H	-5.16062600	-0.24765000	1.81513800
C	-4.43393200	-0.08357600	-1.61051400
H	-3.85600000	-1.00874900	-1.68429800
H	-5.44084700	-0.30120700	-1.98251100
H	-3.98104700	0.65632700	-2.27855300
C	-2.12032700	-1.49075300	0.56640600
H	-2.62646900	-1.85480400	1.46570500
C	1.84584400	2.00716300	-0.05694600
O	2.21299800	1.64347300	-1.27009900
O	0.96260400	1.54022100	0.62665700
C	2.73924100	3.17393800	0.44556100
F	2.33049200	3.60748400	1.64591800
F	2.70677900	4.21478800	-0.41279100
F	4.02501500	2.77038900	0.56014700
H	-2.66730200	-1.88751900	-0.29370900
O	0.57016600	-0.19883200	-2.28235000
H	-1.27470600	0.42326100	0.87786400
H	1.63813200	0.90136600	-1.61814500
H	-0.97869200	-3.05441800	2.43740000
H	1.31446400	-3.98164300	2.52650500

C 2.71124400 -2.05540100 -1.30927500
H 2.47380600 -2.22175200 -2.36402000
H 3.17234700 -1.06178300 -1.21395200
C 3.34948400 -3.45148700 0.47259600
H 3.51548400 -4.52850700 0.57044300
H 4.02325900 -2.92986300 1.16963800
O 3.63799100 -3.07123900 -0.88462700
1TS
C 0.32520500 1.44500400 1.23421700
C -0.12461800 1.45998100 -0.09297600
C -1.27595900 2.17990700 -0.41733400
C -1.96355200 2.87419700 0.58070100
C -1.52419300 2.85875300 1.90279300
C -0.37031800 2.13444400 2.22831900
C 0.70127700 0.74709900 -1.11798700
H 1.57799200 1.27367300 -1.49940200
N 1.69576200 -0.27146900 0.30384400
C 2.89944300 -0.87675700 -0.20060000
O 2.74650200 -1.94470900 -0.76752500
C 4.27577400 -0.19456100 -0.08317300
C 5.25979300 -0.96725500 -0.98568100
H 5.35140100 -2.01084700 -0.67616200
H 4.94011800 -0.95094500 -2.03122600
H 6.24610300 -0.49795900 -0.91881400
C 4.78103000 -0.30038000 1.38071700
H 5.80868300 0.07398700 1.42148700
H 4.19159000 0.28283300 2.09047400
H 4.78927900 -1.34202100 1.71662100
C 4.23863200 1.27819600 -0.55430300
H 3.56029600 1.91129700 0.02165100
H 5.24122500 1.70478300 -0.45169900
H 3.96078600 1.34704500 -1.61124600
C 1.57619600 0.62995500 1.47515400
H 1.51145100 0.04377200 2.39681900
C -1.55352500 -2.01898400 -0.09521800
O -1.72731000 -1.37309800 -1.17946200
O -0.57625200 -2.02728300 0.66295400
C -2.77799100 -2.90994000 0.27470400
F -2.62566300 -3.53158100 1.45961100
F -2.97663500 -3.86827500 -0.66358000
F -3.91381400 -2.17393100 0.34682000
H 2.44954300 1.27645400 1.54555900
O 0.18559600 -0.08370100 -1.95344700
H 0.98094800 -1.01936300 0.37469000
H -0.70580300 -0.64978400 -1.57585800
H -0.02391300 2.10009700 3.25701000
H -2.06866400 3.38909600 2.67841900
C -2.00277900 2.36718300 -1.72666000
H -1.36002200 2.68975200 -2.55200300
H -2.52402600 1.44816000 -2.03189800
C -3.16586300 3.53738500 -0.04146700
H -3.25684600 4.60095500 0.20043700
H -4.10021100 3.03293000 0.24807600
O -2.95185800 3.41567800 -1.46036400
9
C -0.31331000 -1.72203300 -0.08394800
C 0.01270800 -1.45406300 1.25689500
C -0.80881100 -1.85192700 2.30756600
C -2.00656000 -2.54556200 2.03672400
C -2.30876000 -2.80701700 0.71144300
C -1.48315600 -2.40953700 -0.34037600
H -0.53717400 -1.63674700 3.33610500
H -2.66775200 -2.86291600 2.83536400
C 1.32166200 -0.70648000 1.32452700
H 1.17305600 0.31026500 1.69930300
H 2.05169200 -1.21561500 1.95850600
N 1.76107500 -0.68728400 -0.08938300
C 2.90363300 -0.19672300 -0.66354100
O 2.94040900 -0.13143500 -1.90675200
C 4.13702100 0.20734800 0.17852400
C 5.18505000 0.81831900 -0.77339600
H 5.46757100 0.11902800 -1.56333900
H 4.80908900 1.72918700 -1.24828800
H 6.08054900 1.07504300 -0.19853000
C 4.74121600 -1.06397300 0.82498600
H 5.64567500 -0.79162900 1.37906800
H 4.05805800 -1.54955200 1.52659500
H 5.02173000 -1.79580900 0.06030100
C 3.80069900 1.26114900 1.26078800
H 3.22709200 0.85587200 2.09524600
H 4.73751300 1.64923100 1.67372800
H 3.24718300 2.10650000 0.83878700
C 0.73391300 -1.19338700 -1.00965300
O 0.19942700 -0.16065300 -1.85994800
H 1.17317800 -1.94935700 -1.66721600
O -3.42688600 -3.42799900 0.19753100
O -2.04318600 -2.76900800 -1.53896300
C -3.14171000 -3.63985900 -1.19502400
H -4.01386900 -3.37207800 -1.79097000
H -2.83577400 -4.68136800 -1.35224800
C -1.23283300 2.40824300 -0.09069100
O -0.31485500 2.27482800 0.68900300
O -1.48359600 1.71873700 -1.18070100
C -2.31250200 3.50804200 0.10949500
F -2.12504000 4.16339200 1.26497200
F -3.55434800 2.97710700 0.12661300
F -2.26301400 4.41152600 -0.89594200
H -0.78733900 1.00283200 -1.35409300
H 0.96353500 0.13886000 -2.39069800
9TS
C -0.25534400 -1.73424500 0.00944600
C -0.13766800 -1.08241800 1.26026000
C -1.09451400 -1.22893200 2.25254800
C -2.21666500 -2.05440400 2.01716000
C -2.31789900 -2.68528500 0.78894200
C -1.36141800 -2.53927000 -0.21556900
H -0.99572500 -0.72019600 3.20524300
H -2.97651700 -2.19436000 2.77779400
C 1.12907200 -0.27107300 1.25512500
H 0.91660100 0.80268400 1.23960600
H 1.79293700 -0.51930900 2.08302500
N 1.74606600 -0.65514300 -0.04674400
C 2.98676600 -0.25001900 -0.61743200
O 3.08691800 -0.34991500 -1.83163700
C 4.15298700 0.20281300 0.27878300
C 5.36134300 0.47960500 -0.63765300
H 5.65504400 -0.41193300 -1.19707300
H 5.14262700 1.27392200 -1.35594600
H 6.20810100 0.79504500 -0.02092500
C 4.52203700 -0.94389500 1.25353700
H 5.41848900 -0.65540500 1.81094200
H 3.73907900 -1.16380400 1.98319000
H 4.74843600 -1.86582400 0.70799100
C 3.83012400 1.50502300 1.05102200
H 3.04214400 1.39410500 1.79584000
H 4.73460000 1.81669800 1.58260300
H 3.55299900 2.31513400 0.36907200
C 0.87721100 -1.37351000 -0.81039300
O 0.08623000 -0.19381700 -2.15985100
H 1.27087700 -1.95471400 -1.63070100
O -3.28732400 -3.56091500 0.35825100
O -1.69261700 -3.28942200 -1.29965600
C -3.04366100 -3.74949400 -1.04481000
H -3.74006700 -3.13795500 -1.62892700
H -3.11250300 -4.80873700 -1.28925700
C -1.17925200 2.39455600 -0.36570100
O -0.12079400 2.50602600 0.26894100
O -1.49200100 1.58500000 -1.28993300
C -2.35171000 3.36468100 -0.00089900
F -3.41541200 2.67663300 0.49574800
F -2.79639300 4.04675000 -1.08758400
F -2.01230700 4.28348900 0.92897800

H	-0.50476600	0.56218600	-1.75742900
H	0.85043800	0.21614600	-2.59723700

11

C	0.60093400	-1.88680700	-0.08137600
C	0.43660700	-1.10537100	-1.26320800
C	1.46156300	-0.94641100	-2.17753000
C	2.70458600	-1.57767000	-1.93558900
C	2.85578700	-2.32889700	-0.78124100
C	1.83272300	-2.49688500	0.14892300
H	1.33230100	-0.34509100	-3.07044000
H	3.52403700	-1.47700400	-2.63850100
C	-0.94762300	-0.52762900	-1.23839000
H	-0.91209200	0.55992600	-1.07860900
H	-1.53741100	-0.76621300	-2.12429400
N	-1.53659700	-1.15468500	-0.02165600
C	-2.87217800	-1.00763300	0.54675100
O	-3.01860200	-1.42051800	1.67684700
C	-4.01764900	-0.44078400	-0.30232100
C	-5.25174700	-0.32940300	0.61698500
H	-5.51883400	-1.29401500	1.05448200
H	-5.07657100	0.37779700	1.43274400
H	-6.09888600	0.03062600	0.02635500
C	-4.32896400	-1.45025500	-1.43894700
H	-5.18265500	-1.07675400	-2.01262800
H	-3.49655800	-1.58177900	-2.13461900
H	-4.59567800	-2.43108700	-1.03309300
C	-3.70842800	0.96235000	-0.88061600
H	-3.01242900	0.94195600	-1.71906700
H	-4.64514400	1.38845700	-1.25171700
H	-3.31047500	1.63814900	-0.11836800
C	-0.61531500	-1.85147400	0.63852500
O	0.13112200	-0.16041300	2.52888400
H	-0.86536200	-2.34561500	1.56493500
O	3.95406000	-3.04397500	-0.37168400
O	2.23307700	-3.29254800	1.16447400
C	3.65562000	-3.50712200	0.95527600
H	4.20875000	-2.91538100	1.69096300
H	3.86761000	-4.57333000	1.02985000
C	0.63560900	2.70031200	0.39474000
O	-0.35505900	2.44092200	-0.31475100
O	1.11338300	2.09745100	1.38894800
C	1.44590700	3.98981700	0.01646600
F	0.97147800	4.62897900	-1.07807400
F	2.75109400	3.69983900	-0.23854500
F	1.43671800	4.89657900	1.03149800
H	0.44360000	0.67504100	2.09262400
H	-0.59007600	0.10434600	3.11462600

11TS

C	-2.20273200	0.49733800	-0.02524600
C	-1.29234300	-0.15370800	0.87995100
C	-1.62318200	-1.37555200	1.49448500
C	-2.85681500	-1.96648200	1.21715600
C	-3.72739200	-1.32576600	0.32477700
C	-3.42741000	-0.13322700	-0.29271900
H	-0.92772200	-1.85709100	2.17235300
H	-3.14132800	-2.90737500	1.67574600
C	-0.07511400	0.62721200	0.91135500
H	0.43831400	0.80645700	1.85056500
H	0.83033500	-0.19093600	0.21675400
N	-0.38863400	1.80952800	0.14516800
C	0.36774600	3.04116400	-0.04289200
O	-0.27859700	4.00864100	-0.38903500
C	1.89902300	3.07161700	0.08157900
C	2.33026400	4.55292800	0.05267800
H	1.98417100	5.05717300	-0.85182100
H	1.93960400	5.09824500	0.91731600
H	3.42267400	4.60241900	0.08113800
C	2.46859600	2.35069800	-1.17023500
H	3.56102500	2.41800800	-1.14213700
H	2.19794800	1.29326000	-1.19915200
H	2.12334800	2.83121200	-2.09112800
C	2.45734300	2.42280100	1.36969000
H	2.42385400	1.33370400	1.34734100
H	3.50973700	2.70685500	1.46175200
H	1.94075600	2.78081000	2.26610800
C	-1.60332000	1.69604600	-0.43108800
H	-1.96349200	2.45478500	-1.10939900
O	-4.95461600	-1.77578600	-0.12283000
O	-4.43046200	0.23943200	-1.14290900
C	-5.52057700	-0.66479700	-0.83612000
H	-6.24040700	-0.14214400	-0.19464200
H	-5.96932000	-1.01429200	-1.76515000
C	2.12593600	-1.83755400	0.33494300
O	1.58767000	-0.92566900	-0.40506800
O	1.99182100	-2.03585000	1.53470600
C	3.06867100	-2.76161400	-0.49645400
F	4.10557700	-2.05505100	-1.01106700
F	2.41150200	-3.33071600	-1.53525300
F	3.58650300	-3.75540700	0.24833200

4

C	-2.03077200	0.66922800	-0.16331900
C	-1.21907600	0.32035300	1.00239600
C	-1.63054100	-0.69738800	1.92501100
C	-2.80966900	-1.37544900	1.70301400
C	-3.58190300	-1.02986000	0.56426700
C	-3.22750200	-0.06223600	-0.33082700
H	-1.01767100	-0.92791500	2.79010700
H	-3.15102700	-2.15418800	2.37630200
C	-0.10470600	1.15217400	0.96784500
H	0.70230600	1.23613100	1.67164800
H	0.54658500	-0.97155000	0.09794700
N	-0.21693500	1.97771700	-0.14659300
C	0.66268500	3.01412400	-0.61780500
O	0.34662000	3.57430000	-1.65075600
C	1.93402600	3.39286200	0.16694700
C	2.62237600	4.53971200	-0.60252400
H	2.90425200	4.23238200	-1.61282200
H	1.97244400	5.41471200	-0.68380200
H	3.52909900	4.83099700	-0.06392600
C	2.92215400	2.20037800	0.23195700
H	3.85249400	2.54304300	0.69570900
H	2.55517700	1.35565300	0.81605500
H	3.16283100	1.83968400	-0.77342500
C	1.57307700	3.91422500	1.58146200
H	1.13111700	3.15678700	2.23007500
H	2.48722200	4.27238800	2.06522300
H	0.87479300	4.75513400	1.51912700
C	-1.38516700	1.68588100	-0.84053000
H	-1.63232200	2.22023700	-1.74130600
O	-4.75204000	-1.64418100	0.14409000
O	-4.14167700	0.00409100	-1.36133200
C	-5.25869500	-0.77165400	-0.87891400
H	-6.00157900	-0.09091700	-0.44118800
H	-5.67022300	-1.36318500	-1.69572900
C	1.97077500	-2.21388600	0.33365700
O	1.00700700	-1.68167900	-0.40507400
O	2.28855900	-1.91158600	1.45818300
C	2.68961500	-3.33639700	-0.46239800
F	1.81778600	-4.29952100	-0.82755700
F	3.65264100	-3.89951500	0.27879200
F	3.25460000	-2.83800500	-1.58303200

4TS

C	-1.21645000	-1.17234100	-0.06791700
C	-1.15604400	-1.51596300	1.32880700
C	-2.31476800	-1.66882500	2.13445600
C	-3.56110800	-1.49619700	1.55793600
C	-3.60604800	-1.16498900	0.18949900
C	-2.48555000	-0.99555700	-0.60344700
H	-2.22121100	-1.91372000	3.18668800
H	-4.47708800	-1.60747700	2.12638400
C	0.20167000	-1.66171600	1.63524400
H	0.67139100	-1.89464600	2.57940200

N	0.95452600	-1.45650200	0.53241700
C	2.39385900	-1.60968300	0.60915300
O	2.87026300	-1.72993800	1.72035500
C	3.23882400	-1.70399100	-0.67215800
C	4.70257400	-1.92656900	-0.23833100
H	5.07547900	-1.08729200	0.35472100
H	4.81202300	-2.83715500	0.35593300
H	5.32521800	-2.02044100	-1.13293200
C	3.17745300	-0.40458100	-1.51328800
H	3.91030900	-0.48716400	-2.32171200
H	2.20489900	-0.22266500	-1.96948900
H	3.44076700	0.46937000	-0.90995300
C	2.78156100	-2.93042600	-1.50329800
H	1.76009600	-2.84328900	-1.87874800
H	3.44523500	-3.03263000	-2.36748200
H	2.85095700	-3.85093600	-0.91485600
C	0.12587000	-1.00565000	-0.56454700
H	0.27568300	0.40175100	-0.64166600
O	-4.73199700	-0.99866800	-0.56742200
O	-2.85810300	-0.72517400	-1.90117300
C	-4.27303800	-0.47194600	-1.83317500
H	-4.45499300	0.60879000	-1.85586700
H	-4.77767800	-0.99451400	-2.64515900
C	0.38488300	2.24604200	0.33883900
O	0.38496200	1.60436200	-0.78188600
O	0.31562100	1.80953100	1.47964700
C	0.48633900	3.78556900	0.11081800
F	1.59913700	4.10661500	-0.59252400
F	0.53241000	4.46617900	1.27095600
F	-0.58139700	4.24462200	-0.58780700
H	0.39972400	-1.34918600	-1.55551500

12

C	-0.02910900	-1.71724200	-0.23837200
C	0.35250100	-2.06853300	1.08986700
C	-0.39029200	-2.94993300	1.89813100
C	-1.55541600	-3.51311900	1.38331400
C	-1.91987200	-3.15629500	0.08235300
C	-1.18235800	-2.27515800	-0.72332300
H	-0.06495700	-3.17965300	2.90645700
H	-2.16576000	-4.19718100	1.96041700
C	1.55089500	-1.37686100	1.36255300
H	2.14493200	-1.39740200	2.26396400
N	1.96495700	-0.67331900	0.30528000
C	3.20250100	0.08431600	0.38196100
O	3.73043100	0.14120900	1.47320200
C	3.82567400	0.68738400	-0.88568700
C	5.07097600	1.48484300	-0.44606800
H	4.79912400	2.31623900	0.21035400
H	5.78852800	0.85408300	0.08357200
H	5.55863400	1.89403000	-1.33554000
C	2.86904800	1.64834000	-1.63220600
H	3.45110200	2.18822700	-2.38498600
H	2.06173800	1.13854400	-2.15738600
H	2.42864600	2.38607900	-0.95547700
C	4.27866900	-0.47129900	-1.81245000
H	3.44831800	-1.08191600	-2.17474200
H	4.77813300	-0.04343700	-2.68716800
H	4.99091400	-1.12754400	-1.30282300
C	0.95487100	-0.73681000	-0.78967600
O	-2.99789600	-3.59683900	-0.60357900
O	-1.77987200	-2.15002300	-1.94535400
C	-3.02098000	-2.87508000	-1.86008800
H	-3.85923800	-2.17325900	-1.84900100
H	-3.08465800	-3.59002900	-2.68072800
C	-1.47860400	2.22429800	0.37422000
O	-0.68741600	1.94719300	-0.55007600
O	-1.45277300	1.92798800	1.59295700
C	-2.72593100	3.05919200	-0.08382300
F	-2.37450900	4.10936100	-0.87077100
F	-3.45450600	3.56716700	0.93694200
F	-3.57409000	2.28495800	-0.82097600
H	1.40780200	-1.05002100	-1.73123900
O	0.54491500	0.62698400	2.92984100
H	-0.15018300	1.10154300	2.40755800
H	1.17682800	1.30656500	3.19770800
H	0.48880100	0.25462900	-0.89482100

12TS

C	-0.15797800	-1.61198700	-0.12636700
C	0.04478500	-1.69503500	1.27162200
C	-0.81365300	-2.39075200	2.12797000
C	-1.93249300	-3.04065200	1.58975200
C	-2.12679100	-2.94479400	0.21624600
C	-1.26434400	-2.24711600	-0.63698000
H	-0.62620900	-2.42045000	3.19556700
H	-2.62328300	-3.59595800	2.21310700
C	1.24308200	-0.94247400	1.57741100
H	1.87517300	-1.11352400	2.43629400
N	1.83759100	-0.56699200	0.40249300
C	3.06934400	0.13634700	0.45201500
O	3.41665400	0.55919900	1.54743500
C	3.94498200	0.28071000	-0.80484500
C	5.22397700	1.03927900	-0.39574000
H	4.99339800	2.04579900	-0.03652100
H	5.76821100	0.51432500	0.39311200
H	5.87769800	1.12646900	-1.26873600
C	3.23692600	1.09080300	-1.91918600
H	3.96920400	1.30326300	-2.70408700
H	2.40947700	0.55651300	-2.38681500
H	2.86234700	2.04695400	-1.54143800
C	4.34366000	-1.12734400	-1.31358400
H	3.48821000	-1.72609500	-1.63449400
H	5.00962800	-1.01551900	-2.17487800
H	4.88131200	-1.68652100	-0.54112600
C	0.93899500	-0.81270500	-0.76125000
O	-3.11842000	-3.52191400	-0.52177300
O	-1.68617600	-2.37247300	-1.93407300
C	-2.98540100	-2.99195900	-1.85812200
H	-3.76033100	-2.23608900	-2.02365800
H	-3.04118000	-3.80874100	-2.57728600
C	-1.27511300	2.28013900	0.15892400
O	-0.39282100	2.15675300	-0.70123400
O	-1.32140200	1.84862500	1.34948700
C	-2.56394700	3.07378700	-0.23824200
F	-2.80788800	4.09450400	0.62353500
F	-3.65914300	2.26633000	-0.21669300
F	-2.49768700	3.61134500	-1.47554200
H	1.46058700	-1.36001200	-1.54753300
O	0.59353100	0.64340600	2.46818200
H	-0.15666300	1.14020500	1.94112900
H	1.37613300	1.21909600	2.50368200
H	0.56849000	0.14336500	-1.14741400

14

C	0.05762700	-1.72555200	-0.00779900
C	0.01088100	-1.44174000	1.36751500
C	-0.93806900	-2.00373000	2.21803000
C	-1.90209600	-2.88887700	1.69800500
C	-1.84949000	-3.15532200	0.33916400
C	-0.89362800	-2.59049800	-0.50591900
H	-0.94557400	-1.76061400	3.27545600
H	-2.65213600	-3.34764500	2.33211900
C	1.10741600	-0.47845000	1.71302800
H	1.78724000	-0.83530900	2.49221600
N	1.84832500	-0.32888700	0.45288500
C	2.96942500	0.45752800	0.48723700
O	3.21731900	1.06032400	1.54828300
C	3.94233100	0.55234400	-0.71251900
C	5.01332600	1.60946200	-0.37444900
H	4.56823800	2.60032500	-0.24502900
H	5.55044800	1.35956900	0.54303600
H	5.73371300	1.66116400	-1.19707000
C	3.24197800	0.98358200	-2.02330500
H	4.00965600	1.23286000	-2.76297400

H	2.62084100	0.20082100	-2.46050900
H	2.62299200	1.87435700	-1.87371600
C	4.64183800	-0.81740300	-0.89430600
H	3.94377600	-1.61920600	-1.14849500
H	5.37212700	-0.74461000	-1.70707900
H	5.17712200	-1.10752800	0.01575300
C	1.17502600	-0.98922600	-0.68858400
O	-2.64406100	-4.01159700	-0.38834100
O	-1.05712200	-3.06755800	-1.78470400
C	-2.32517300	-3.75309800	-1.76736200
H	-3.09308700	-3.10210100	-2.20266300
H	-2.23370900	-4.69771100	-2.30278300
C	-1.59686900	2.20578900	-0.07009400
O	-0.80906200	1.87432700	-0.92943500
O	-1.52508100	2.00053700	1.22499000
C	-2.89858200	2.97986600	-0.41922500
F	-3.00342600	3.17690300	-1.74205600
F	-2.91736500	4.18837500	0.18607700
F	-3.99109200	2.29663800	-0.01073700
H	1.85249200	-1.66486300	-1.21710800
O	0.56407000	0.77389600	2.17922500
H	-0.68260400	1.50226700	1.49522400
H	1.34693000	1.33825300	2.33498500
H	0.78606400	-0.25254300	-1.39774500

14TS

C	-0.29110000	1.61739300	-0.00073000
C	0.01310200	1.05615900	1.25567800
C	0.99971100	1.59053600	2.08709400
C	1.72371800	2.73063900	1.69121000
C	1.40885100	3.27665900	0.46008200
C	0.42459700	2.73531000	-0.37085200
H	1.20713000	1.13165600	3.04776100
H	2.50055600	3.15371000	2.31726300
C	-0.82782500	-0.10412600	1.67673200
H	-1.81549000	0.11703800	2.08174400
N	-1.45014100	-0.44309700	-0.17656200
C	-2.60216700	-1.31134000	-0.21489600
O	-2.35502700	-2.50164600	-0.14707100
C	-4.04227700	-0.76855100	-0.28094200
C	-4.99650900	-1.94263600	0.02142700
H	-4.88540800	-2.74661400	-0.70952500
H	-4.81293300	-2.36027900	1.01518300
H	-6.02834400	-1.58036200	-0.01565400
C	-4.33531100	-0.26883700	-1.72137300
H	-5.39511400	-0.00328600	-1.78773900
H	-3.75806400	0.61311000	-2.00521100
H	-4.13943000	-1.05700400	-2.45500800
C	-4.30085900	0.34678800	0.75920100
H	-3.67500500	1.23225300	0.62869300
H	-5.34114700	0.67370400	0.66764500
H	-4.16441900	-0.02517600	1.77999200
C	-1.33529300	0.89434100	-0.81212100
H	-1.04207300	0.78790600	-1.86084800
O	2.00168700	4.33721800	-0.17363000
O	0.37031000	3.43492900	-1.54887600
C	1.20816900	4.59175900	-1.34830100
H	1.86445000	4.71718600	-2.20932100
H	0.57578400	5.47008200	-1.17637700
C	1.91691200	-1.93365300	-0.03909300
O	1.87134900	-1.85008600	1.22580200
O	1.04980700	-1.64977000	-0.88010600
C	3.28781100	-2.46299400	-0.56260900
F	3.31596700	-2.60856000	-1.90214900
F	3.58778500	-3.66809100	-0.01977400
F	4.29056900	-1.61077900	-0.22913000
H	-2.28861900	1.41889900	-0.77909700
O	-0.30697600	-1.18225900	2.16573900
H	-0.62343900	-1.01542600	-0.44299100
H	0.66527700	-1.43061300	1.76098400

2

C	-1.47925000	1.19431300	0.36890500
C	-0.12796000	0.75362000	0.56085500
C	0.94797300	1.65365600	0.43826800
C	0.76154900	3.00253600	0.12563300
C	-0.54870700	3.41283600	-0.05785000
C	-1.62937800	2.53055800	0.05906000
H	1.95503100	1.29004600	0.60278200
H	1.59270800	3.69133300	0.03278400
C	0.13340200	-0.64136800	0.89941000
H	-0.74471600	-1.28752100	1.03031600
N	-2.74299600	-0.62905600	-0.66734400
C	-3.53480600	-1.71776100	-0.93848900
O	-3.47831200	-2.19596600	-2.07881200
C	-4.42887100	-2.36422200	0.14995300
C	-5.22456600	-3.51109600	-0.50474300
H	-5.87141800	-3.14154800	-1.30524600
H	-4.56127500	-4.26681500	-0.93209500
H	-5.85318600	-3.98912500	0.25383500
C	-5.43986900	-1.34962900	0.73455200
H	-6.15053200	-1.88422800	1.37337200
H	-4.97308600	-0.57873400	1.34982600
H	-6.01131500	-0.85717900	-0.05962400
C	-3.54529000	-2.96518400	1.26968800
H	-2.99027300	-2.21034300	1.83298900
H	-4.18166400	-3.49741500	1.98481000
H	-2.82639100	-3.68199000	0.85982100
C	-2.70168000	0.30238500	0.46824800
H	-3.58847200	0.94234600	0.48326800
O	-0.99578100	4.65229100	-0.38098800
O	-2.79571600	3.20504500	-0.20157200
C	-2.44017600	4.58857000	-0.36621900
H	-2.82502300	4.95405800	-1.31929000
H	-2.81351400	5.16955000	0.48225400
C	4.36982400	-1.09363000	-0.23737900
O	3.79153700	-0.65865400	0.86166000
O	3.85493400	-1.62202100	-1.19731500
C	5.90097400	-0.83744300	-0.17312000
F	6.51172200	-1.28215200	-1.28095400
F	6.45112200	-1.46543800	0.88958100
F	6.16291600	0.48400400	-0.05242200
H	-2.68612900	-0.25053400	1.40931800
O	1.24711700	-1.16096800	1.05288400
H	-2.26437100	-0.30872400	-1.50237200
H	2.79350000	-0.82416200	0.86132500

----

b

1

C	1.62025800	1.64999900	-0.22688000
C	0.39962800	0.97308100	-0.49424900
C	-0.82256100	1.64970700	-0.40030300
C	-0.86939400	3.00063200	-0.04087700
C	0.32838400	3.67670000	0.23113300
C	1.54624500	2.99505900	0.13710000
C	0.40665500	-0.44359700	-0.89091500
H	1.39064800	-0.91168600	-1.02552700
N	3.14985500	-0.01326100	0.74575200
C	4.09811900	-0.98442100	0.95982500
O	4.10336100	-1.54704100	2.06209400
C	5.09696100	-1.40357500	-0.14931300
C	6.03516200	-2.47899100	0.43389500
H	6.60686300	-2.09186800	1.28159900
H	5.47733700	-3.35315700	0.77798100
H	6.73928900	-2.79812400	-0.34152600
C	5.96436600	-0.20847000	-0.61196400
H	6.74809600	-0.57674200	-1.28235400
H	5.40224500	0.54999500	-1.15947100
H	6.45512600	0.27523500	0.23950800
C	4.33518300	-2.02521900	-1.34482100
H	3.68745100	-1.31255700	-1.86203800
H	5.05784300	-2.39526000	-2.07990400
H	3.72166200	-2.87240600	-1.02129500

C 2.98484700 0.98857200 -0.31593700
H 3.74391500 1.77357000 -0.23173600
C -3.71305500 -1.62426000 0.21090500
O -3.21699900 -1.18827700 -0.92906100
O -3.11596700 -1.92882000 1.21838000
C -5.26131700 -1.71501700 0.11958300
F -5.78795700 -2.13308500 1.27965300
F -5.63450200 -2.58261900 -0.84730600
F -5.79981800 -0.51100200 -0.17552400
H 3.11940200 0.51930900 -1.29243800
O -0.59142300 -1.14096900 -1.08488000
H 2.62034600 0.17151700 1.59134700
H -2.21342200 -1.13391800 -0.90624700
H 2.46193300 3.53777100 0.35306300
H 0.32984100 4.72210500 0.51441200
O -2.10604200 3.56170800 0.01414300
C -2.21916700 4.94453200 0.36896600
H -3.28554800 5.16627000 0.34625400
H -1.82823400 5.12544100 1.37599900
H -1.69651200 5.57962400 -0.35450400
H -1.75211000 1.13686200 -0.61707000

1TS

C 0.10797000 1.58598400 0.85242200
C -0.22471300 1.41615200 -0.49638200
C -1.36323000 2.00483000 -1.04158300
C -2.18759900 2.79134400 -0.22076500
C -1.86430600 2.96597200 1.13351500
C -0.72099500 2.35620100 1.66249900
C 0.71820400 0.62970700 -1.35226900
H 1.58756000 1.15906300 -1.74446600
N 1.65841900 -0.12546900 0.25396300
C 2.95089900 -0.66393500 -0.07762800
O 2.94488100 -1.79648100 -0.52722200
C 4.25004300 0.15241800 0.07671400
C 5.36493600 -0.59753800 -0.68193500
H 5.51846400 -1.60086100 -0.27859500
H 5.12929700 -0.69274400 -1.74560000
H 6.29993200 -0.03725900 -0.58430100
C 4.64072900 0.20382200 1.57845400
H 5.62048300 0.68372400 1.66704500
H 3.93966300 0.77193800 2.19315200
H 4.72109300 -0.80522400 1.99470700
C 4.13478800 1.57592600 -0.51648200
H 3.37901900 2.20062400 -0.03568200
H 5.09632600 2.08349800 -0.39095400
H 3.92321200 1.54167900 -1.59026000
C 1.36125900 0.87564000 1.30859600
H 1.22063800 0.37081500 2.26955500
C -1.40522100 -2.16163200 -0.11583700
O -1.56000300 -1.66394100 -1.27585600
O -0.47511600 -2.00702300 0.68719800
C -2.59242900 -3.07814400 0.31060700
F -2.40265300 -3.64524500 1.51790800
F -2.78364000 -4.07991900 -0.58126700
F -3.74774400 -2.36921900 0.37465700
H 2.19176800 1.57138800 1.41292700
O 0.31707800 -0.33702900 -2.10170300
H 1.00591700 -0.92600100 0.35576800
H -0.55504000 -0.90087900 -1.71456000
H -0.49334900 2.48101700 2.71732300
H -2.49642400 3.55674000 1.78471300
O -3.27743500 3.33485300 -0.83065500
C -4.17409700 4.13350100 -0.05237700
H -4.95581700 4.45267700 -0.74113500
H -4.61806900 3.54884000 0.76064500
H -3.66435800 5.01283300 0.35671200
H -1.62558600 1.87134000 -2.08580100

9

C 0.50226800 -1.70717800 0.50007400
C 0.26744300 -1.63054700 -0.87395000
C 1.25392100 -2.04080300 -1.76534200
C 2.47157900 -2.52500400 -1.27037200
C 2.69557100 -2.59712700 0.11574400
C 1.69931900 -2.18591100 1.01605000
H 1.09703600 -1.98956700 -2.83896700
H 3.23474400 -2.84237100 -1.97024100
C -1.09999100 -1.06385800 -1.15627500
H -1.03135700 -0.08622700 -1.64262600
H -1.70326200 -1.72792500 -1.78094000
N -1.67551000 -0.93533100 0.20287700
C -2.91340700 -0.51965200 0.61303200
O -3.07890600 -0.31677000 1.83113900
C -4.09652900 -0.36502600 -0.37224600
C -5.29088400 0.22376900 0.40588000
H -5.57742800 -0.41231800 1.24629000
H -5.05949400 1.21775000 0.79959500
H -6.14699600 0.31272200 -0.27070500
C -4.49581500 -1.76600400 -0.89812400
H -5.36978400 -1.67061800 -1.55121300
H -3.70135000 -2.24389400 -1.47708900
H -4.76332200 -2.43237700 -0.07141300
C -3.77657300 0.58737200 -1.54909000
H -3.08698300 0.15929200 -2.27776100
H -4.70740500 0.80953100 -2.08085400
H -3.36051300 1.53642500 -1.19548300
C -0.69313500 -1.21284000 1.25862900
O -0.36492600 -0.03823400 2.02720400
H -1.10911800 -1.94255100 1.95996000
C 0.87020800 2.49872400 0.07142700
O -0.00334500 2.21372000 -0.71877900
O 1.13871300 1.93680900 1.22770500
C 1.86780500 3.65724700 -0.20730500
F 1.59421700 4.25942500 -1.37461400
F 3.13723600 3.19545600 -0.26295300
F 1.81046600 4.59105500 0.76797500
H 0.50853600 1.17109300 1.44366600
H -1.21064700 0.22400700 2.44201300
H 1.88469600 -2.24509900 2.08368400
O 3.84994800 -3.05745800 0.68285200
C 4.91189100 -3.48737600 -0.17180300
H 5.26328500 -2.66790400 -0.80881800
H 5.71618300 -3.80195700 0.49307800
H 4.60055500 -4.33340000 -0.79492500

9TS

C 0.72199500 -1.77781500 0.42027300
C 0.57984800 -1.34911300 -0.90885100
C 1.66460300 -1.42814000 -1.77296700
C 2.87612400 -1.93997600 -1.29306400
C 3.00663300 -2.37139300 0.04342600
C 1.91672000 -2.29052500 0.92001000
H 1.59060200 -1.10373800 -2.80603300
H 3.71861400 -2.00189000 -1.97055900
C -0.82055800 -0.85526400 -1.13295000
H -0.85084400 0.22896200 -1.28874500
H -1.32782800 -1.36844100 -1.95104500
N -1.46900700 -1.17898500 0.17010100
C -2.81289500 -0.95833900 0.59279400
O -3.00691100 -0.92573200 1.79914100
C -3.95153500 -0.84486800 -0.43386000
C -5.26518400 -0.65249300 0.35124900
H -5.44901600 -1.48234500 1.03803500
H -5.25007800 0.27263000 0.93367800
H -6.09614600 -0.59959000 -0.35842500
C -4.04355900 -2.16368200 -1.24187100
H -4.89292800 -2.09684400 -1.92888600
H -3.15167800 -2.36455100 -1.84009100
H -4.21133300 -3.01952700 -0.58030400
C -3.76666000 0.36986700 -1.37705300
H -2.95864200 0.24083600 -2.09736200
H -4.69103800 0.50283200 -1.94734300
H -3.58254900 1.28935900 -0.81381900
C -0.54925900 -1.57542500 1.08933700

O	-0.17664900	-0.05828900	2.30646300
H	-0.88779500	-2.10460000	1.96730000
C	0.41700700	2.58220800	0.24220300
O	-0.56829800	2.30389500	-0.45608600
O	0.89479800	1.98965700	1.25365300
C	1.23576300	3.85922000	-0.14483600
F	0.76146100	4.48286100	-1.24573000
F	2.53729600	3.55606300	-0.39462600
F	1.22824100	4.77402100	0.86069000
H	0.21206600	0.77347700	1.82557800
H	-1.05979500	0.17670700	2.63584000
H	2.02283000	-2.61116800	1.95041800
O	4.15281700	-2.88193600	0.57115100
C	5.31876700	-2.97189000	-0.25451500
H	5.62996500	-1.98228400	-0.60606000
H	6.09573500	-3.39313100	0.38271400
H	5.14699800	-3.63530100	-1.10915100

11

C	0.70734000	-1.99271500	0.32968300
C	0.61471800	-1.33232200	-0.91436000
C	1.74165800	-1.22664500	-1.71759100
C	2.93989300	-1.79168700	-1.26666400
C	3.02494000	-2.45513100	-0.01994700
C	1.89663200	-2.56019800	0.79776500
H	1.71304700	-0.71861000	-2.67571900
H	3.81794100	-1.70960900	-1.89549000
C	-0.77792300	-0.81391700	-1.08669800
H	-0.79715600	0.28554100	-1.02495500
H	-1.26842000	-1.15420600	-1.99938000
N	-1.46173000	-1.35289700	0.12087000
C	-2.85231200	-1.20861400	0.54522200
O	-3.08774500	-1.48892400	1.70005700
C	-3.93340900	-0.81108700	-0.46832400
C	-5.26319000	-0.70308500	0.30606400
H	-5.51605800	-1.64053100	0.80685300
H	-5.21961500	0.08587300	1.06200900
H	-6.06247000	-0.45966300	-0.39974200
C	-4.06189800	-1.93877300	-1.52616700
H	-4.87599300	-1.68056400	-2.21017000
H	-3.15825600	-2.07086800	-2.12586400
H	-4.30583900	-2.89611200	-1.05526500
C	-3.64922800	0.55111500	-1.14789600
H	-2.84652500	0.51129700	-1.88400400
H	-4.55598000	0.85914400	-1.67688900
H	-3.40615300	1.32630500	-0.41605000
C	-0.58423100	-1.93935100	0.92724700
O	-0.08443900	-0.01542100	2.66584900
H	-0.90512400	-2.35501600	1.87093300
C	0.41737800	2.66857500	0.30292500
O	-0.46546200	2.25569500	-0.47396800
O	0.84593700	2.19389800	1.38438100
C	1.14895400	3.99167200	-0.11676400
F	0.62652900	4.57712200	-1.21970500
F	2.46336400	3.75520200	-0.38812300
F	1.11437600	4.92380400	0.87173000
H	0.22081600	0.78980200	2.17174500
H	-0.86879400	0.26276100	3.15653900
H	1.96077700	-3.05629700	1.75950300
O	4.16295800	-3.01979000	0.46107100
C	5.37088900	-2.92036700	-0.30313000
H	5.65896900	-1.87394400	-0.44835800
H	6.13117600	-3.42928300	0.28824200
H	5.26609500	-3.41940500	-1.27244200

11TS

C	1.92406600	-1.45940500	-0.31411100
C	1.37496700	-0.54383700	0.63502000
C	2.20322600	0.45059800	1.18716700
C	3.52938600	0.50881700	0.79099900
C	4.06749600	-0.39960900	-0.17048600
C	3.27052500	-1.38342200	-0.73281500
H	1.81423000	1.16492400	1.90461600
H	4.16627200	1.27095200	1.22346600
C	-0.03710800	-0.80986900	0.77455300
H	-0.50769000	-0.79910100	1.75258400
H	-0.62872600	0.30190900	0.14934600
N	-0.23989700	-2.01368900	0.00532500
C	-1.40775900	-2.87253500	-0.11060900
O	-1.19611200	-4.00599100	-0.49276600
C	-2.82809300	-2.33617900	0.12956400
C	-3.77933000	-3.55074600	0.14292800
H	-3.71080500	-4.12686600	-0.78196200
H	-3.55732900	-4.22086600	0.97910300
H	-4.80676900	-3.19232300	0.25386700
C	-3.18144600	-1.42720600	-1.07863500
H	-4.21487000	-1.08407100	-0.96451600
H	-2.54019100	-0.54556100	-1.13800800
H	-3.11098500	-1.98135100	-2.02012300
C	-3.00874200	-1.55709300	1.45362200
H	-2.57435100	-0.55818800	1.42264400
H	-4.08141700	-1.43253800	1.62949300
H	-2.59611100	-2.10232300	2.30881500
C	0.89031900	-2.34713600	-0.65560500
H	0.89387500	-3.17708500	-1.34678800
C	-1.12197500	2.32851100	0.35926000
O	-1.11847700	1.28141200	-0.39742500
O	-0.72135500	2.45115700	1.50873500
C	-1.75526000	3.55481100	-0.36809400
F	-1.14107200	3.80292200	-1.54919600
F	-1.67738200	4.67593600	0.37203000
F	-3.06675700	3.33131600	-0.63236500
O	5.36735500	-0.35952100	-0.58713400
H	3.68239000	-2.06859600	-1.46575800
C	6.24478700	0.63922300	-0.05833300
H	7.20515300	0.46986900	-0.54491900
H	5.88911100	1.64759200	-0.29679500
H	6.36274400	0.53188800	1.02560800

4

C	0.90385100	-2.02088200	-0.57336100
C	0.69780900	-1.31619100	0.68109000
C	1.81970800	-1.02407400	1.51353600
C	3.07175000	-1.40419700	1.10065500
C	3.27412800	-2.09034900	-0.15155600
C	2.21677400	-2.39710200	-0.97745300
H	1.68779300	-0.50876900	2.46005700
H	3.92196300	-1.18383500	1.73414200
C	-0.66610900	-1.07488400	0.80790100
H	-1.21094300	-0.63919600	1.62514900
H	0.13180700	0.90042200	-0.00903300
N	-1.29360400	-1.62446000	-0.30939500
C	-2.68575300	-1.66853500	-0.65527800
O	-2.97208300	-2.16185900	-1.73136500
C	-3.76257900	-1.13333200	0.31085400
C	-5.13428900	-1.36964500	-0.35520900
H	-5.21481100	-0.83734900	-1.30639500
H	-5.30943500	-2.43152400	-0.54573700
H	-5.91942500	-1.00405100	0.31351200
C	-3.60148900	0.39050900	0.54137200
H	-4.44907300	0.74482600	1.13655200
H	-2.68995600	0.66045900	1.07536900
H	-3.61128900	0.93163600	-0.41034700
C	-3.73745500	-1.91623100	1.64822900
H	-2.81855400	-1.77819600	2.21935900
H	-4.57063800	-1.57348800	2.26999400
H	-3.86872600	-2.98914800	1.47366400
C	-0.34266400	-2.19131200	-1.15170700
H	-0.64652900	-2.64764700	-2.07843400
C	0.28538500	2.75749900	0.39374500
O	0.27297800	1.75794500	-0.47636600
O	0.13681800	2.69529600	1.59028100
C	0.53222600	4.10002000	-0.34602200
F	1.72985100	4.08653600	-0.96885000
F	0.51802200	5.12585100	0.51516400

F	-0.42010700	4.31639900	-1.27798200
O	4.51873700	-2.47260900	-0.58166300
H	2.39355600	-2.91792900	-1.91280300
C	5.66411300	-2.18840500	0.22391100
H	6.51581100	-2.58167800	-0.33160200
H	5.79470600	-1.11018200	0.36940400
H	5.60505200	-2.68971100	1.19659200

4TS

C	-1.29950900	-1.06648000	-0.36878100
C	-1.39355300	-1.46629200	1.00519100
C	-2.64920500	-1.51414400	1.65906600
C	-3.77905700	-1.18862000	0.94314700
C	-3.68242700	-0.80548100	-0.43319600
C	-2.45352700	-0.73550200	-1.08805400
H	-2.72154300	-1.79990400	2.70353000
H	-4.74530000	-1.22351100	1.42986500
C	-0.09879300	-1.76104000	1.43766400
H	0.25286500	-2.07308100	2.40979700
N	0.77765700	-1.58237600	0.42146300
C	2.18002400	-1.86461200	0.62784900
O	2.52709200	-2.11703700	1.76578800
C	3.15127100	-1.92107500	-0.56476700
C	4.54533300	-2.27250200	-0.00510300
H	4.90102600	-1.50680900	0.68948100
H	4.53875200	-3.23036200	0.52078200
H	5.25280800	-2.33909800	-0.83705800
C	3.25619000	-0.56285800	-1.29974600
H	4.05769800	-0.63519900	-2.04157600
H	2.34606300	-0.27790600	-1.82578600
H	3.51502000	0.24203700	-0.60500700
C	2.71391900	-3.05003300	-1.53301400
H	1.73986900	-2.87290400	-1.99282700
H	3.45189800	-3.12702500	-2.33770900
H	2.67630000	-4.01511900	-1.01747100
C	0.09655700	-1.01153900	-0.72113400
H	0.35591700	0.38690200	-0.72370300
C	0.60981700	2.20343700	0.28903300
O	0.54718300	1.57848500	-0.83972700
O	0.52916600	1.75843600	1.42533100
C	0.81754800	3.73453400	0.07727000
F	-0.21414200	4.27518200	-0.61641700
F	1.95091500	3.98282700	-0.62365500
F	0.91147300	4.39812300	1.24436800
H	0.43911200	-1.32606100	-1.70019300
H	-2.41986700	-0.43094900	-2.12857500
O	-4.76649700	-0.48954900	-1.18254700
C	-6.07664000	-0.52694400	-0.60068600
H	-6.75602500	-0.23705000	-1.40146500
H	-6.32548500	-1.53567700	-0.25623700
H	-6.16015200	0.18538800	0.22606100

12

C	-0.46651400	-1.62654000	-0.54156700
C	-0.30396600	-2.11209900	0.78171800
C	-1.35107800	-2.77867300	1.44442700
C	-2.54696100	-2.95682500	0.77219000
C	-2.70159900	-2.47018400	-0.55345300
C	-1.65285300	-1.79662200	-1.21962000
H	-1.22968800	-3.13937000	2.46012300
H	-3.36514000	-3.46454200	1.26565000
C	1.00157100	-1.78823900	1.19620000
H	1.48243900	-2.00479000	2.13837000
N	1.68462200	-1.17755300	0.22192900
C	3.06174200	-0.78329900	0.43987600
O	3.49833200	-0.94502400	1.56150300
C	3.92332900	-0.28007300	-0.72878500
C	5.26473400	0.19493200	-0.13238200
H	5.11991400	1.04263600	0.54357900
H	5.76462100	-0.60157000	0.42305000
H	5.91976100	0.51444400	-0.94808100
C	3.28651600	0.89883300	-1.50382900
H	4.06284300	1.35255100	-2.12700400
H	2.48134700	0.59283300	-2.17185300
H	2.89954000	1.67014200	-0.83234100
C	4.19595800	-1.47692600	-1.67833700
H	3.28729900	-1.87298700	-2.13922100
H	4.85473700	-1.13958700	-2.48450400
H	4.69711000	-2.29351400	-1.14936800
C	0.79501700	-0.92626700	-0.94595500
C	-0.81053300	2.51814800	0.28132000
O	0.00891600	2.11161100	-0.56516500
O	-1.05077900	2.12005000	1.44821500
C	-1.73011200	3.70891400	-0.16580600
F	-1.32599500	4.30586600	-1.31179900
F	-1.80146100	4.68512300	0.77566500
F	-3.00616900	3.27724600	-0.38091700
H	1.23383900	-1.31072600	-1.86737600
O	0.40375100	0.30083500	2.86960800
H	-0.09684400	0.95732000	2.32135400
H	1.14994100	0.78933300	3.24030400
H	0.62097500	0.15712000	-1.02003100
H	-1.81436900	-1.43206500	-2.22797200
O	-3.83161700	-2.60804800	-1.26491600
C	-4.97751200	-3.25650300	-0.68454300
H	-5.74458200	-3.22495100	-1.45651700
H	-4.74830900	-4.29587200	-0.43217500
H	-5.32042400	-2.71450300	0.20126700

12TS

C	-0.48528900	-1.56625900	-0.47446100
C	-0.41274500	-1.79552900	0.91148500
C	-1.48731100	-2.35925000	1.60477900
C	-2.63866900	-2.69079400	0.89693500
C	-2.70862600	-2.45466300	-0.49445700
C	-1.62162100	-1.89011500	-1.19188600
H	-1.43644200	-2.52973800	2.67520600
H	-3.47759700	-3.12711200	1.42343100
C	0.87778400	-1.34482500	1.38008100
H	1.38705400	-1.72702800	2.25276700
N	1.63644800	-0.99967700	0.29024800
C	2.96610100	-0.55892300	0.49482000
O	3.29328900	-0.30672900	1.64891200
C	3.96086300	-0.48388500	-0.67700900
C	5.31981500	-0.03051100	-0.10615000
H	5.25389500	0.96703700	0.33633500
H	5.68336000	-0.71782600	0.66171700
H	6.05195600	0.00049000	-0.91871300
C	3.52031300	0.53990000	-1.75261300
H	4.34693700	0.67308400	-2.45736000
H	2.65303300	0.21713900	-2.32937800
H	3.29565600	1.51471400	-1.30965600
C	4.12657800	-1.89469400	-1.29493900
H	3.20585400	-2.27713500	-1.74162500
H	4.88220300	-1.84799600	-2.08542000
H	4.46742200	-2.61529300	-0.54452900
C	0.80522400	-0.95639700	-0.94579500
C	-0.72427600	2.48410700	0.15679300
O	0.15704800	2.20990300	-0.66863800
O	-0.96236100	1.98092100	1.29543000
C	-1.73712800	3.61661000	-0.21827000
F	-1.75898200	4.59708400	0.72159500
F	-3.00363300	3.12937800	-0.31238900
F	-1.45671800	4.21043600	-1.39862600
H	1.27232600	-1.52875300	-1.74796300
O	0.50411500	0.24731200	2.38997200
H	-0.06196700	0.95692600	1.87287600
H	1.39134800	0.61553400	2.54202100
H	0.66533500	0.08576100	-1.25278500
H	-1.70492400	-1.72614100	-2.26095800
O	-3.79041900	-2.74805200	-1.25291700
C	-4.95894700	-3.30106000	-0.63148400
H	-5.68101700	-3.43157400	-1.43651500
H	-4.73792900	-4.27096900	-0.17449600
H	-5.36341400	-2.61456600	0.11904400

14

C	-0.39531500	-1.73012800	-0.40397300
C	-0.45641000	-1.61094200	0.98645000
C	-1.59359800	-2.01365600	1.68018500
C	-2.68038100	-2.53468300	0.96989100
C	-2.61304000	-2.65118200	-0.43039700
C	-1.46046800	-2.24812900	-1.12748800
H	-1.65023200	-1.92564200	2.76107500
H	-3.56549600	-2.84603500	1.51020100
C	0.81542300	-1.02844900	1.51876500
H	1.32144800	-1.65104800	2.26282300
N	1.66163900	-0.91366400	0.32300300
C	2.93752500	-0.45626700	0.51345900
O	3.25313800	-0.08596800	1.66028700
C	3.98767400	-0.45729000	-0.62357000
C	5.25862500	0.24143800	-0.09986600
H	5.06173300	1.28731200	0.15310500
H	5.65161100	-0.25192900	0.79168400
H	6.02632400	0.21527500	-0.87988300
C	3.51145500	0.30075700	-1.88603000
H	4.36768500	0.43698900	-2.55458200
H	2.74800900	-0.23592700	-2.45062200
H	3.12293900	1.29347400	-1.63593100
C	4.33855700	-1.92502800	-0.97186500
H	3.48267800	-2.48345000	-1.36011700
H	5.11862000	-1.93730500	-1.74045800
H	4.72106400	-2.45480600	-0.09324000
C	0.92489000	-1.22225000	-0.92339000
C	-0.84992100	2.51891800	0.00876200
O	-0.10152200	2.08351800	-0.83944000
O	-0.97588200	2.13776800	1.25905800
C	-1.83688800	3.67863800	-0.30133400
F	-1.72145300	4.08317500	-1.57523900
F	-1.59815700	4.74225500	0.49831900
F	-3.11611700	3.29027200	-0.10070100
H	1.44827100	-1.97336400	-1.52038900
O	0.57896300	0.25006300	2.14716800
H	-0.34010000	1.38373300	1.50420300
H	1.46666200	0.55406900	2.42201900
H	0.78595900	-0.32127400	-1.52797400
H	-1.43230500	-2.34765700	-2.20802400
O	-3.62076800	-3.14809900	-1.20396900
C	-4.83333500	-3.56903300	-0.57291000
H	-5.48019100	-3.91323600	-1.37968300
H	-4.65042000	-4.39276600	0.12599400
H	-5.31437800	-2.73662800	-0.04758900

14TS

C	0.00250700	1.68162300	-0.38703200
C	0.20902900	1.25593400	0.93170200
C	1.33004900	1.69643600	1.63827300
C	2.23566300	2.57518700	1.03888700
C	2.01780800	3.00412200	-0.28033600
C	0.89737700	2.54655200	-0.99787200
H	1.50052800	1.36526200	2.65789200
H	3.10041800	2.90696600	1.59922800
C	-0.82726300	0.37557700	1.54302400
H	-1.72869200	0.85220200	1.92940900
N	-1.60206400	-0.07589100	-0.23566100
C	-2.91480500	-0.66099500	-0.13050200
O	-2.93401700	-1.85443600	0.11209800
C	-4.20294900	0.17559200	-0.26082500
C	-5.37977400	-0.70410600	0.20969900
H	-5.47391500	-1.60470500	-0.40111200
H	-5.25601600	-1.01288500	1.25133700
H	-6.30792600	-0.13025000	0.12726900
C	-4.43532700	0.53191800	-1.75378500
H	-3.68535900	1.21111500	-2.16339700
H	-4.45365600	-0.37103900	-2.37204300
H	-5.40926800	1.02292600	-1.84648100
C	-4.16841000	1.44935200	0.61641100
H	-3.36677500	2.14530600	0.36065200
H	-5.11304300	1.98668000	0.48781500
H	-4.07889100	1.19584000	1.67782000
C	-1.22367100	1.10165900	-1.05616800
H	-1.02152300	0.79276900	-2.08614000
C	1.39168800	-2.21041800	0.06702700
O	1.43392400	-1.93952000	1.30585100
O	0.54878700	-1.88429600	-0.78265200
C	2.60578100	-3.06567800	-0.41049200
F	2.52176000	-3.41013100	-1.71054300
F	2.71373300	-4.21203800	0.30429200
F	3.76719700	-2.38084300	-0.24965300
H	-2.03352700	1.82889000	-1.07194800
O	-0.51702000	-0.71014600	2.17292600
H	-0.93271700	-0.84908100	-0.42300900
H	0.36553400	-1.21099100	1.79172300
O	2.84022800	3.84812100	-0.95718600
C	4.02331500	4.33033600	-0.30960200
H	4.68687700	3.50293600	-0.03700600
H	4.51709300	4.96948400	-1.04090400
H	3.77282300	4.91705700	0.58076600
H	0.76695700	2.86352400	-2.02785100

2

C	-1.56200700	1.43458700	0.34574400
C	-0.29224500	0.81818600	0.54830500
C	0.86931500	1.60792900	0.45129500
C	0.81521800	2.96431600	0.16290900
C	-0.43937800	3.56486200	-0.03894600
C	-1.61293600	2.78989200	0.05492400
H	1.83638600	1.14711900	0.61698200
H	1.73164800	3.53709900	0.10011900
C	-0.18893200	-0.59863800	0.87018100
H	-1.13223400	-1.14742400	0.98894700
N	-2.98598300	-0.26805000	-0.70133900
C	-3.86393900	-1.29116700	-0.96637400
O	-3.84673600	-1.77906700	-2.10337200
C	-4.81063700	-1.85462600	0.12375900
C	-5.67271600	-2.95967600	-0.51846800
H	-6.28504900	-2.56498000	-1.33367100
H	-5.05465100	-3.76432000	-0.92379700
H	-6.33872000	-3.38128800	0.24165100
C	-5.75853800	-0.76034600	0.67043600
H	-6.50507300	-1.22902300	1.32033600
H	-5.24718000	-0.00007800	1.26365000
H	-6.29388600	-0.25883400	-0.14305100
C	-3.98764500	-2.48990800	1.27031700
H	-3.39847600	-1.76188600	1.83404400
H	-4.66960700	-2.97000200	1.98042000
H	-3.30724200	-3.25740400	0.88725800
C	-2.87626200	0.67169900	0.42032100
H	-3.69296400	1.40074300	0.40228300
C	3.98867800	-1.50461600	-0.21459600
O	3.43467300	-0.95547100	0.84489600
O	3.44529700	-2.05811800	-1.14419600
C	5.53425600	-1.35940100	-0.14527900
F	6.11816000	-1.88362800	-1.23260300
F	6.02846400	-1.99254200	0.94200600
F	5.89428300	-0.05828300	-0.06354800
H	-2.94727700	0.13418900	1.36762800
O	0.86149000	-1.23855100	1.02450400
H	-2.48724900	0.00852600	-1.54050600
H	2.42515700	-1.04482900	0.84311200
O	-0.62558300	4.86902400	-0.32642700
C	0.51681400	5.72911600	-0.46170400
H	1.16244800	5.39100600	-1.27799200
H	0.11248500	6.71295000	-0.69525000
H	1.08273500	5.77463000	0.47375300
H	-2.56493300	3.28443500	-0.10943300

----

c

1

C	1.72720500	1.91095100	0.06130400
C	0.46014000	1.43352100	-0.38457800
C	-0.68901300	2.25950500	-0.20596800
C	-0.56597800	3.51631700	0.40662300
C	0.67974800	3.95013500	0.84490100
C	1.81933100	3.15654300	0.67727100
C	0.39821700	0.13083100	-1.04539800
H	1.37246400	-0.30869500	-1.29570400
N	2.98787400	-0.08022600	0.77476400
C	3.82820100	-1.16384800	0.86895300
O	3.70008800	-1.90458500	1.85173100
C	4.86422900	-1.48705200	-0.23797300
C	5.65595800	-2.73545100	0.19948900
H	6.19734100	-2.55801100	1.13277500
H	4.99704500	-3.59341400	0.35235800
H	6.38331500	-2.98884800	-0.57883500
C	5.86841200	-0.32632000	-0.43584900
H	6.66543800	-0.65817600	-1.10942300
H	5.42052400	0.56387600	-0.88087100
H	6.33321300	-0.03925900	0.51361900
C	4.13841700	-1.81588700	-1.56454700
H	3.59794400	-0.96292700	-1.98315200
H	4.87644300	-2.12734200	-2.31142600
H	3.42782600	-2.63774800	-1.42975200
C	3.01107900	1.10603500	-0.08918900
H	3.84515900	1.76389700	0.17469900
C	-3.53907900	-1.13564900	0.24449800
O	-3.15554900	-0.83839700	-0.97530600
O	-2.88945600	-1.11004900	1.26704000
C	-5.03519800	-1.55693100	0.24618500
F	-5.45885000	-1.83444300	1.48821200
F	-5.22916400	-2.65547800	-0.51712900
F	-5.81560300	-0.56849700	-0.24876900
H	3.15796500	0.80880800	-1.12943700
O	-0.60360000	-0.52235000	-1.35501400
H	2.41496700	0.00837500	1.60753700
H	-2.18263500	-0.56623400	-1.01607600
H	2.78200700	3.51331900	1.02862500
H	0.76477500	4.92015800	1.32510200
H	-1.43231100	4.14947800	0.54563300
O	-1.86350000	1.78595200	-0.66834400
C	-3.03676100	2.61104400	-0.59610200
H	-3.82633500	2.02530100	-1.06399800
H	-3.30089000	2.82375700	0.44419800
H	-2.89012900	3.54400300	-1.14862400

1TS

C	0.42504000	1.32886600	1.35608600
C	0.04278000	1.59186700	0.03986100
C	-0.88102100	2.62355500	-0.23626800
C	-1.42144900	3.35702700	0.82852400
C	-1.03714300	3.06557000	2.14104700
C	-0.11408100	2.05677400	2.41798200
C	0.68899200	0.81117900	-1.06288400
H	1.64171100	1.18056500	-1.44550400
N	1.48307300	-0.51041200	0.24753900
C	2.60710600	-1.21206700	-0.30763400
O	2.33125500	-2.17229100	-1.00594100
C	4.06076800	-0.75027000	-0.08095100
C	4.95060600	-1.48940900	-1.10218100
H	4.88805300	-2.57260500	-0.97788300
H	4.66261100	-1.24950600	-2.12969600
H	5.99060600	-1.18224600	-0.95475700
C	4.51004500	-1.17207900	1.34381500
H	5.57363600	-0.93962200	1.45739600
H	3.97295100	-0.65542400	2.14175000
H	4.38317900	-2.24938900	1.48927900
C	4.24223200	0.77052200	-0.29382400
H	3.66701700	1.39042600	0.39692700
H	5.29735400	1.02023300	-0.14471900
H	3.98138100	1.06028300	-1.31703300
C	1.44632500	0.22776100	1.52855300
H	1.17454000	-0.45196900	2.34211700
C	-2.02651400	-1.58303800	-0.18427100
O	-2.12893100	-0.77291100	-1.15745500
O	-1.02455700	-1.89691900	0.47438100
C	-3.38774400	-2.23362900	0.21100500
F	-4.25019400	-1.29216700	0.67356700
F	-3.25815200	-3.16568500	1.17543500
F	-3.97767900	-2.83162100	-0.85167200
H	2.42291900	0.64999500	1.75702200
O	0.01385000	0.13968500	-1.92686200
H	0.66607000	-1.14802000	0.20641100
H	-0.95389200	-0.22459700	-1.56814600
H	0.17117900	1.83184500	3.44077000
H	-1.47402000	3.63534700	2.95546400
H	-2.13931700	4.14625000	0.64488900
O	-1.16702700	2.84174900	-1.54570800
C	-2.11784300	3.85788000	-1.88679400
H	-2.18788800	3.83684200	-2.97374100
H	-3.09761800	3.63809800	-1.45059300
H	-1.77309000	4.84408000	-1.55841000

9

C	-0.08094100	1.88354700	0.12335300
C	-0.32462300	1.41856100	1.41153400
C	0.34465800	1.95250500	2.51411000
C	1.26639700	2.97553300	2.28092500
C	1.51539100	3.46471200	0.99033200
C	0.83490100	2.92140600	-0.10926600
H	0.15634400	1.59069400	3.51987800
H	1.80476200	3.41385300	3.11614100
C	-1.36690100	0.32964400	1.38784900
H	-0.93311900	-0.63384200	1.67320500
H	-2.20216800	0.55297800	2.05580700
N	-1.79301400	0.32276900	-0.02840800
C	-2.74734900	-0.42060700	-0.66974500
O	-2.75035800	-0.40540100	-1.91496100
C	-3.83408400	-1.20213000	0.10599400
C	-4.68216200	-1.98234400	-0.91896500
H	-5.13534800	-1.31662800	-1.65680500
H	-4.07883200	-2.71925000	-1.45680300
H	-5.48140400	-2.51222500	-0.39072900
C	-4.75401000	-0.19389800	0.83818400
H	-5.56365200	-0.73881100	1.33502300
H	-4.22880700	0.38465200	1.60260600
H	-5.20579000	0.50919400	0.13063000
C	-3.23609600	-2.22067900	1.10577300
H	-2.78941300	-1.75399800	1.98474400
H	-4.03987600	-2.87298100	1.46210700
H	-2.48062500	-2.85301300	0.62790000
C	-0.92615000	1.15444800	-0.87397700
O	-0.11595100	0.35719100	-1.76367500
H	-1.53993100	1.80377800	-1.50427800
O	0.98556600	3.32725900	-1.39870600
C	1.90365600	4.38865500	-1.68087700
H	1.85696900	4.53765700	-2.75932100
H	1.60837500	5.31107600	-1.16901900
C	1.94582000	-1.84892300	-0.15691500
O	0.98619900	-2.13230100	0.52724600
O	2.02418000	-0.96654200	-1.12577500
C	3.31834200	-2.54942800	0.04526800
F	3.25069000	-3.47311500	1.01590300
F	4.27045600	-1.65259500	0.38789800
F	3.71983900	-3.16445000	-1.08935200
H	1.14172300	-0.48879500	-1.29027900
H	-0.76071300	-0.10801600	-2.33159200
H	2.23209600	4.26548300	0.85716100
H	2.92345100	4.11258400	-1.39206200

9TS

C	-0.34741700	1.98456100	0.20282800

C -0.23610100 1.30043000 1.42184200
C 0.53818400 1.80554000 2.45929900
C 1.19702700 3.02052000 2.23483700
C 1.09227100 3.72277000 1.02551700
C 0.31026000 3.21106000 -0.01899000
H 0.63207600 1.28720500 3.40726900
H 1.81045800 3.44449700 3.02418800
C -1.05586800 0.04278300 1.35446700
H -0.42009500 -0.84974200 1.35101100
H -1.79148700 -0.01996700 2.15757000
N -1.72531200 0.17638400 0.03139700
C -2.62241800 -0.71046800 -0.61729200
O -2.71370300 -0.60947500 -1.83448900
C -3.48609400 -1.69018700 0.19622600
C -4.35678600 -2.48082400 -0.80076300
H -4.97926500 -1.81770700 -1.40615000
H -3.74197200 -3.08011600 -1.47786000
H -5.01099300 -3.15594500 -0.24083800
C -4.41365700 -0.88143800 1.13773500
H -5.05971600 -1.57742100 1.68191800
H -3.86481600 -0.29344300 1.87737700
H -5.05498500 -0.20084100 0.56846500
C -2.62968300 -2.69928400 1.00075800
H -2.12844800 -2.25297700 1.86003200
H -3.29268600 -3.48118600 1.38378700
H -1.87322900 -3.17644100 0.37093000
C -1.19759600 1.22064400 -0.67845100
O 0.01391900 0.48584700 -2.01044500
H -1.76873000 1.62860400 -1.49857300
O 0.12762000 3.79277300 -1.22284000
C 0.79490400 5.03293800 -1.50137500
H 0.50429300 5.29676700 -2.51727200
H 0.46872500 5.81429200 -0.80768700
C 2.02597400 -1.67453600 -0.31924700
O 1.06492700 -2.16262100 0.29155500
O 2.06659100 -0.71739700 -1.14755300
C 3.43381800 -2.31183600 -0.07206000
F 4.34500900 -1.38087300 0.31383800
F 3.91343000 -2.88946900 -1.20619300
F 3.42361100 -3.26850300 0.88152600
H 0.81092100 -0.04435700 -1.60516200
H -0.56031600 -0.14419800 -2.47787900
H 1.62243800 4.65991100 0.91236500
H 1.88083100 4.90893500 -1.44760000
11
C -1.93126200 1.13058900 0.25525200
C -1.13570400 0.81598600 1.38179100
C -0.81981900 1.78178800 2.32227500
C -1.32779000 3.07207000 2.10620100
C -2.11945800 3.40864100 0.99791500
C -2.43780100 2.43733600 0.04179600
H -0.20522100 1.56328200 3.18804500
H -1.10162800 3.85236200 2.82666500
C -0.73227300 -0.62241700 1.28104900
H 0.34852100 -0.69720700 1.07654300
H -0.99476100 -1.21374800 2.15862000
N -1.47384800 -1.07932600 0.07467000
C -1.52926400 -2.39305300 -0.54607200
O -2.00470400 -2.44067000 -1.66110500
C -1.08623400 -3.63948300 0.23428500
C -1.23127800 -4.84619700 -0.71572200
H -2.25758600 -4.95714500 -1.07338700
H -0.57653700 -4.74771800 -1.58587500
H -0.95171100 -5.75539000 -0.17583900
C -2.04112000 -3.84047500 1.44036700
H -1.76551500 -4.76823000 1.95123100
H -1.98610500 -3.03348800 2.17476700
H -3.08048200 -3.93218500 1.10985500
C 0.38672800 -3.56249100 0.70395200
H 0.55335800 -2.84741400 1.50912000
H 0.67176200 -4.54831100 1.08370100
H 1.05903800 -3.31312400 -0.12174600
C -2.07153100 -0.04214800 -0.51367500
O -0.17754000 0.55405000 -2.37303000
H -2.61372600 -0.18071400 -1.43689300
O -3.18250400 2.62720900 -1.05750300
C -3.69013700 3.94355100 -1.33996800
H -4.23302100 3.84706800 -2.27864300
H -4.36990700 4.27193100 -0.54814900
C 2.81419100 0.34953600 -0.31361700
O 2.30677800 -0.42420000 0.52141100
O 2.31913200 0.90742200 -1.32418200
C 4.32729700 0.70488100 -0.09691200
F 4.87014600 0.12630400 0.99966700
F 4.50822400 2.04669600 0.04186600
F 5.08532500 0.31475000 -1.15800900
H 0.71716700 0.64788400 -1.95756000
H -0.06340500 -0.08158700 -3.09118400
H -2.47574300 4.42545600 0.89463300
H -2.86739500 4.65462800 -1.45655200
11TS
C -2.38159800 -0.25612300 0.11174300
C -1.31189700 -0.62454200 0.98496600
C -1.31508300 -1.88280800 1.61612900
C -2.37966400 -2.72824100 1.34715400
C -3.44114200 -2.38801500 0.46611500
C -3.45477900 -1.15410700 -0.16486800
H -0.51536200 -2.18017900 2.28501000
H -2.41452900 -3.70271500 1.82575500
C -0.33743200 0.43877200 0.96877600
H 0.15245800 0.75159400 1.88450100
H 0.71310000 -0.13807900 0.23997200
N -0.97024900 1.49141700 0.20511400
C -0.56485800 2.86500700 -0.03052900
O -1.43968300 3.62091700 -0.40538800
C 0.90426800 3.30397500 0.08592800
C 0.92774900 4.84531700 0.01095300
H 0.46213000 5.21179300 -0.90600600
H 0.40412800 5.29351400 0.86104600
H 1.96809700 5.18284100 0.03215700
C 1.65193400 2.72507300 -1.14526900
H 2.68842700 3.07721900 -1.11978400
H 1.66793200 1.63328600 -1.14740800
H 1.19898900 3.07389200 -2.07882400
C 1.60985200 2.86816700 1.39152500
H 1.87056800 1.80994800 1.40415500
H 2.54681200 3.42721400 1.47135700
H 1.01132700 3.10139500 2.27839300
C -2.13224400 1.04676800 -0.32462900
H -2.70092300 1.67001400 -0.99777700
O -4.39640200 -0.70530500 -1.02495900
C -5.49744800 -1.56751800 -1.34477700
H -6.07860800 -1.80336700 -0.44743700
H -6.11337200 -1.00734700 -2.04705800
C 2.44382100 -1.32769300 0.30816600
O 1.59926800 -0.67427600 -0.41833200
O 2.49198400 -1.43753300 1.52583300
C 3.51282700 -2.04763500 -0.57111000
F 2.93211000 -2.94406500 -1.40569900
F 4.41439000 -2.71336000 0.17354400
F 4.19336500 -1.16166500 -1.33865400
H -5.14286200 -2.49006900 -1.81553900
H -4.23251800 -3.10786000 0.30011000
4
C -1.83974300 -1.28211200 0.04504800
C -1.01070900 -0.84135100 1.15159900
C -0.49638800 -1.79526900 2.08687900
C -0.80460400 -3.11590800 1.88525000
C -1.61356300 -3.57126500 0.78745200
C -2.12850900 -2.67731400 -0.12396400
H 0.11690400 -1.47921100 2.92431900
H -0.43031900 -3.86421000 2.57806900

C	-0.89846000	0.53997000	1.04550900
H	-0.42826000	1.23912800	1.71222400
H	1.04081700	-0.16818400	0.12523200
N	-1.64895400	0.94537200	-0.06363500
C	-1.88365900	2.25937200	-0.59407500
O	-2.53463200	2.33284400	-1.62026500
C	-1.34641900	3.51219600	0.12634600
C	-1.81298300	4.74130200	-0.68182700
H	-1.42440500	4.72102400	-1.70319600
H	-2.90331100	4.79479800	-0.73618100
H	-1.44631300	5.64762900	-0.19043500
C	0.20322200	3.51802400	0.15143900
H	0.54141300	4.47057500	0.57122000
H	0.63592000	2.72058300	0.75651700
H	0.60943600	3.43481600	-0.86180100
C	-1.94044500	3.62267400	1.55364800
H	-1.62224300	2.82392800	2.22478800
H	-1.62045600	4.57289600	1.99257200
H	-3.03479900	3.62027000	1.52021400
C	-2.21243400	-0.16484900	-0.67450000
H	-2.81052500	-0.05190700	-1.56220300
O	-2.90602500	-2.98158600	-1.19692200
C	-3.23338900	-4.35539300	-1.42621300
H	-3.79608100	-4.76922600	-0.58214300
H	-3.85315400	-4.36660700	-2.32244800
C	2.93865000	-0.16239100	0.32318600
O	1.85168500	-0.31502900	-0.41897100
O	2.99851100	0.12413300	1.49450100
C	4.20747900	-0.39865800	-0.54017100
F	4.17446100	-1.61221900	-1.12847800
F	5.31303500	-0.32610400	0.21267100
F	4.29699100	0.53403200	-1.51351900
H	-2.32857300	-4.94966000	-1.59489200
H	-1.80833000	-4.63270400	0.69715500

4TS

C	1.09854500	-1.54527200	-0.01490100
C	0.93636800	-1.83099200	-1.39927300
C	2.02466400	-2.24383600	-2.20965500
C	3.24972000	-2.37514800	-1.59278100
C	3.42728000	-2.10614200	-0.20577800
C	2.36970500	-1.68365700	0.58994600
H	1.89284400	-2.44371500	-3.26724200
H	4.11483700	-2.69212800	-2.16648900
C	-0.42274000	-1.62087300	-1.68841600
H	-0.94618000	-1.69962200	-2.62992500
N	-1.08281500	-1.25512700	-0.57179900
C	-2.51483900	-1.00662600	-0.62959600
O	-3.00846600	-0.93911500	-1.73574800
C	-3.34282600	-0.93124200	0.66289400
C	-4.81920500	-0.75834600	0.24982500
H	-4.97222500	0.17014800	-0.30640100
H	-5.16245800	-1.58779500	-0.37363600
H	-5.43617900	-0.72550900	1.15264700
C	-2.94119400	0.28065100	1.53995800
H	-3.65909700	0.35918300	2.36218500
H	-1.94747800	0.19400600	1.97829200
H	-2.98224400	1.21248700	0.96814300
C	-3.20358900	-2.26068900	1.44777800
H	-2.18999400	-2.44749000	1.80734900
H	-3.86259600	-2.22031600	2.32052800
H	-3.51062100	-3.11336200	0.83364000
C	-0.15008400	-1.05483000	0.51287400
H	0.04884600	0.29636100	0.61209500
O	2.43164200	-1.39297100	1.91653200
C	3.69965600	-1.48837500	2.57503800
H	4.42699000	-0.80632400	2.12176300
H	3.51726700	-1.19632200	3.60865600
C	0.30176400	2.17579400	-0.34653000
O	0.25349700	1.53242200	0.76819600
O	0.13816200	1.76337600	-1.48879000
C	0.62419600	3.68617900	-0.12341600
F	-0.28408000	4.26871300	0.69744000
F	0.62795600	4.38137300	-1.27659500
F	1.84426400	3.84400100	0.44822000
H	-0.48361700	-1.33707000	1.50523800
H	4.08085200	-2.51496700	2.54358500
H	4.41429700	-2.23772500	0.22125200

12

C	-0.17695400	1.86075900	-0.18789900
C	-0.60325000	2.11340900	1.12759000
C	-0.02961000	3.11629200	1.92820800
C	0.99176900	3.86021200	1.35361500
C	1.43151100	3.61926600	0.03618000
C	0.85585400	2.61269600	-0.75471700
H	-0.36370400	3.29036200	2.94431300
H	1.47462100	4.64720400	1.92328000
C	-1.65151500	1.18795200	1.40253300
H	-2.24213000	1.09358500	2.30129800
N	-1.92299500	0.43907900	0.33943900
C	-2.97786700	-0.56952200	0.40994100
O	-3.43927700	-0.77956100	1.51016500
C	-3.50049900	-1.23292800	-0.87082200
C	-4.54880500	-2.28060100	-0.44219200
H	-4.09705400	-3.06793000	0.16775300
H	-5.36080300	-1.82896500	0.13243000
H	-4.97248700	-2.74168600	-1.33896600
C	-2.38882500	-1.95180300	-1.67478900
H	-2.87286300	-2.59422500	-2.41620600
H	-1.73659000	-1.26942800	-2.21996200
H	-1.76690000	-2.58256200	-1.03403300
C	-4.19893100	-0.15333000	-1.73899400
H	-3.51574900	0.62488400	-2.08757400
H	-4.62245100	-0.63950600	-2.62313300
H	-5.01647900	0.32654000	-1.19201600
C	-0.95383300	0.72007900	-0.75425200
O	1.20792400	2.30535000	-2.02675500
C	1.87445200	-1.75596700	0.44897700
O	0.96913700	-1.75367900	-0.40871300
O	1.87105400	-1.37040200	1.64407600
C	3.26366900	-2.28219500	-0.05584100
F	3.15007800	-3.24445700	-1.00385700
F	4.03566000	-2.79823800	0.93000500
F	3.97666400	-1.26196200	-0.61823600
H	-1.46702900	0.95669000	-1.68739900
O	-0.31882500	-0.46228500	2.96566400
H	0.45771100	-0.80619100	2.45019100
H	-0.85182700	-1.23895900	3.18066600
H	-0.31441400	-0.16690100	-0.88218200
H	2.23355800	4.22939400	-0.36127800
C	2.28801200	3.02609900	-2.63709600
H	2.39594900	2.59955400	-3.63338900
H	2.05076300	4.09226100	-2.71558200
H	3.21604700	2.88635700	-2.07363200

12TS

C	-0.03637500	1.85812400	-0.08085100
C	-0.29682100	1.93072100	1.29043000
C	0.37220900	2.82438000	2.13570700
C	1.32250500	3.65350800	1.54705500
C	1.59996300	3.59483400	0.16939600
C	0.92309100	2.69227000	-0.66462800
H	0.16641500	2.86112100	3.19942600
H	1.87019200	4.36483300	2.15671500
C	-1.31861700	0.94387600	1.58987200
H	-1.98647200	0.97118700	2.43776100
N	-1.76061700	0.40081200	0.42475000
C	-2.79047100	-0.58391300	0.46689600
O	-3.06477000	-1.03931300	1.56750600
C	-3.55462000	-0.97381400	-0.80817000
C	-4.59196000	-2.04584300	-0.41711000
H	-4.10640100	-2.95245600	-0.04567000
H	-5.27381500	-1.68501800	0.35669600
H	-5.17946200	-2.30827800	-1.30199300

C	-2.62500100	-1.56880900	-1.89601000
H	-3.25256700	-2.03659600	-2.66066400
H	-2.01282500	-0.81936500	-2.39866900
H	-1.96153100	-2.33488300	-1.48518400
C	-4.30177500	0.27371300	-1.34477600
H	-3.62717600	1.08184500	-1.63836100
H	-4.87830200	-0.01435800	-2.22938700
H	-5.00061900	0.66596700	-0.59883000
C	-0.90462500	0.81929200	-0.71981600
O	1.11157600	2.55696300	-2.00309600
C	1.69447700	-1.88250500	0.29850400
O	0.73706800	-1.97043600	-0.48429000
O	1.76286900	-1.34757000	1.44362600
C	3.05957400	-2.47230000	-0.19007100
F	2.91578200	-3.37624900	-1.18511000
F	3.75158600	-3.08481800	0.80070300
F	3.85637300	-1.47680600	-0.67273200
H	-1.51287700	1.21944800	-1.53266000
O	-0.31565700	-0.44063300	2.59309900
H	0.50038000	-0.81115700	2.07236200
H	-0.96629500	-1.15875600	2.66381300
H	-0.32625700	-0.04115200	-1.07397400
H	2.34811900	4.26208700	-0.24084200
C	2.10260800	3.37081000	-2.64379100
H	2.08426100	3.08110200	-3.69375800
H	1.85626400	4.43386500	-2.55049800
H	3.09597600	3.17757200	-2.22587000

14

C	-0.40405000	1.82776500	0.11291200
C	-0.27307500	1.51292100	1.45921600
C	0.51475800	2.27751000	2.32282300
C	1.17792800	3.38083300	1.78401600
C	1.05773100	3.71663300	0.42615900
C	0.26112300	2.93786600	-0.42488800
H	0.61202500	2.02278700	3.37298800
H	1.80081500	4.00077800	2.42170100
C	-1.08336800	0.29006800	1.77612100
H	-1.82765200	0.44232800	2.56370700
N	-1.76487700	-0.00806800	0.51019500
C	-2.64493900	-1.05638500	0.51870300
O	-2.72379500	-1.73356500	1.56131800
C	-3.56449400	-1.36576600	-0.68705300
C	-4.33104100	-2.66850900	-0.38087700
H	-3.64871600	-3.51616300	-0.26907600
H	-4.91596500	-2.58474100	0.53765500
H	-5.01318400	-2.88342100	-1.20974600
C	-2.77683500	-1.57143600	-2.00305700
H	-3.45595200	-1.98554800	-2.75531100
H	-2.37166400	-0.64588100	-2.41393200
H	-1.95355400	-2.28115300	-1.87097700
C	-4.59079300	-0.21609400	-0.84155200
H	-4.12111600	0.74230600	-1.07734300
H	-5.28060500	-0.45599700	-1.65755100
H	-5.17991900	-0.09183500	0.07303300
C	-1.29694700	0.85233400	-0.60033000
O	0.07119500	3.16758600	-1.75568800
C	2.18165500	-1.57535700	-0.10215400
O	1.31289100	-1.45655600	-0.93843900
O	2.08836100	-1.41662900	1.19865600
C	3.63572400	-1.96016200	-0.49313200
F	3.74977800	-2.13197000	-1.81872800
F	4.01063100	-3.10985700	0.11094800
F	4.50598800	-0.99515300	-0.12057200
H	-2.13141800	1.34887900	-1.10246500
O	-0.23292100	-0.78914300	2.21142500
H	1.15121700	-1.16242000	1.49269600
H	-0.84372000	-1.53843300	2.35650400
H	-0.73778900	0.27230900	-1.34069500
H	1.58693100	4.58301500	0.04802400
C	0.72386000	4.29135500	-2.35500700
H	0.42946000	4.27815400	-3.40414800
H	0.39529500	5.22871600	-1.89280700
H	1.81281200	4.20062200	-2.27803500

14TS

C	-0.55966500	1.66100100	0.13593400
C	-0.15231300	1.13927700	1.36050000
C	0.65368000	1.87838800	2.23671500
C	1.03949700	3.16340400	1.85606300
C	0.63625000	3.70791000	0.63132600
C	-0.16840300	2.95643600	-0.23636400
H	0.96555600	1.46068400	3.18785300
H	1.66783300	3.75417400	2.51515400
C	-0.67148100	-0.21336600	1.73752800
H	-1.67628700	-0.25446100	2.16091900
N	-1.24429700	-0.60203000	-0.15284700
C	-2.20070900	-1.67347100	-0.21560500
O	-1.72920400	-2.79678000	-0.17866500
C	-3.72007100	-1.41993000	-0.27132000
C	-4.42896500	-2.76144000	0.00838200
H	-4.16264700	-3.51722700	-0.73380400
H	-4.16929700	-3.15159800	0.99639000
H	-5.51158700	-2.60563600	-0.02779300
C	-4.10707100	-0.96033600	-1.70301300
H	-5.19810000	-0.89684300	-1.76584200
H	-3.70457800	0.01831500	-1.97078300
H	-3.76847500	-1.68536500	-2.44976800
C	-4.18713800	-0.39518200	0.78883500
H	-3.74031700	0.59492200	0.67893100
H	-5.27079900	-0.27001000	0.70035000
H	-3.98250000	-0.75555600	1.80241300
C	-1.38738600	0.75311800	-0.73893300
H	-1.03016300	0.75204800	-1.77308200
O	-0.61460700	3.37938500	-1.45171700
C	2.39031800	-1.29894300	-0.08078100
O	2.34571200	-1.24647900	1.18961500
O	1.46762900	-1.21612900	-0.90221900
C	3.83877400	-1.49118300	-0.62467700
F	3.88456900	-1.54492100	-1.96988300
F	4.38566100	-2.63863900	-0.15439500
F	4.64306300	-0.47141000	-0.23354900
H	-2.43069100	1.06353900	-0.75088300
O	0.10392700	-1.15425300	2.14726300
H	-0.32370000	-0.99182000	-0.42964200
H	1.12797200	-1.14727600	1.71423700
H	0.96068200	4.70507000	0.36046000
C	-0.21837400	4.67466500	-1.91775900
H	-0.67783900	4.78602800	-2.89942000
H	-0.58460700	5.46103500	-1.24918900
H	0.87066900	4.74089200	-2.01132600

2

C	-0.99481100	1.66388000	0.43135800
C	-0.00903000	1.27322300	1.36087200
C	1.10163300	2.09025400	1.63450000
C	1.22322600	3.31575500	0.98960000
C	0.26002000	3.72590200	0.06504600
C	-0.84280300	2.90570500	-0.22081700
H	1.83367300	1.77356400	2.36915900
H	2.06706900	3.96408400	1.20286900
C	-0.14854100	0.00479900	2.10065000
H	-1.16663400	-0.34478300	2.32264600
N	-1.66275300	-0.56411100	-0.33526300
C	-2.31626600	-1.75002200	-0.57460200
O	-1.68591000	-2.64692200	-1.14779700
C	-3.77075500	-1.98899900	-0.09261100
C	-4.20098700	-3.39676300	-0.55170500
H	-4.18349300	-3.48344300	-1.64153800
H	-3.54481100	-4.16946500	-0.14408000
H	-5.22202100	-3.58944600	-0.20595200
C	-4.75311100	-0.96383500	-0.70774500
H	-5.77764200	-1.26190100	-0.46094600
H	-4.61324500	0.05104200	-0.33210100
H	-4.66707800	-0.94096800	-1.79953500

C	-3.82884600	-1.94682200	1.45325600
H	-3.58742100	-0.96422700	1.86705900
H	-4.84283000	-2.19594500	1.78401900
H	-3.14281700	-2.67945700	1.89086400
C	-2.16014100	0.75620900	0.08064100
H	-2.74317800	1.22626100	-0.71224700
O	-1.81160000	3.24198400	-1.11774600
C	2.94461700	-0.96111700	-0.04749900
O	3.10069600	-0.92534000	1.25919400
O	1.92591400	-0.80816700	-0.68454900
C	4.30824300	-1.22566600	-0.74288900
F	4.15779700	-1.34245700	-2.06996000
F	4.87819200	-2.35984600	-0.28298800
F	5.16661000	-0.20668600	-0.50668400
H	-2.81567400	0.64858000	0.94926600
O	0.80368400	-0.65883000	2.51924800
H	-0.73944800	-0.56422700	-0.76022900
H	2.22602300	-0.75796300	1.73248900
H	0.37501300	4.68140700	-0.43109300
C	-1.68977200	4.46586800	-1.85071400
H	-2.55510300	4.49575400	-2.51218700
H	-1.71186700	5.33081100	-1.17918400
H	-0.77096600	4.47797400	-2.44659600

----

d

1

C	1.65329100	-1.18876200	0.24486900
C	0.41734300	-0.62120100	0.66080000
C	-0.77057500	-1.41213800	0.57153700
C	-0.72180900	-2.71810500	0.05456600
C	0.51432100	-3.23901100	-0.38778200
C	1.68832600	-2.47777800	-0.27164900
C	0.42443000	0.72818700	1.20945400
H	1.42268400	1.12589800	1.43490600
N	3.00010500	0.66380000	-0.65836600
C	3.88842400	1.69226000	-0.85959800
O	3.77801300	2.34972800	-1.90205700
C	4.95928800	2.06029200	0.19934800
C	5.81249200	3.21341700	-0.36605100
H	6.32879000	2.91484900	-1.28249700
H	5.20058600	4.08844400	-0.59675700
H	6.56445000	3.49937900	0.37676400
C	5.90114400	0.86972200	0.50018400
H	6.73169400	1.22304300	1.12027600
H	5.41493000	0.05878000	1.04545400
H	6.32632500	0.45843700	-0.42179200
C	4.27205600	2.55609400	1.49432000
H	3.68655400	1.78080700	1.99570000
H	5.03676300	2.89200100	2.20289700
H	3.60933300	3.40239100	1.28680000
C	2.97678900	-0.43801700	0.31019200
H	3.77535600	-1.15692400	0.10112400
C	-3.31896300	1.99108300	-0.39567600
O	-3.02931500	1.85410700	0.87611200
O	-2.59621100	1.83835200	-1.35664300
C	-4.80954100	2.39831400	-0.56420100
F	-5.12122000	2.57643600	-1.85689400
F	-5.07921400	3.54929500	0.09201600
F	-5.62744300	1.44093700	-0.06733100
H	3.15137200	-0.05512700	1.31749600
O	-0.53472000	1.47231800	1.45354400
H	2.40111700	0.53526800	-1.46711600
H	-2.06951200	1.55829800	1.02102200
H	2.63195300	-2.88564400	-0.61060600
O	-1.91829200	-0.82218300	0.96793800
C	-2.95560000	-1.55207200	1.65599400
H	-3.52879500	-0.78960500	2.18408600
H	-3.59273300	-2.08689800	0.95270700
H	-2.51863800	-2.24762700	2.37778400
O	-1.88924600	-3.40476800	-0.14916800
O	0.46711300	-4.46897600	-0.94013000
C	-2.06906600	-4.64606900	0.56478200
H	-1.35393100	-5.39870800	0.22879200
H	-1.96509000	-4.48527000	1.64345200
H	-3.08589600	-4.96941500	0.33915600
C	1.67046100	-5.06179100	-1.45253000
H	1.36749400	-6.03062000	-1.84727700
H	2.09274000	-4.45175100	-2.25664500
H	2.40703800	-5.20178700	-0.65540300

1TS

C	0.13607200	-1.09151800	0.87014900
C	0.32490300	-0.86099800	-0.48852400
C	1.61037600	-0.99669400	-1.06251400
C	2.69744800	-1.33025400	-0.23603100
C	2.48539800	-1.52401900	1.15360500
C	1.20319300	-1.42239200	1.70319000
C	-0.87252200	-0.55324100	-1.33091500
H	-1.44065900	-1.39552500	-1.72812300
N	-2.02811700	-0.26490000	0.28970800
C	-3.42421500	-0.39805500	-0.02452100
O	-3.94831200	0.59110400	-0.50640100
C	-4.19359300	-1.71827700	0.18127100
C	-5.53248100	-1.60200000	-0.57685300
H	-6.13467300	-0.77155800	-0.20198100
H	-5.37196500	-1.44738600	-1.64748000
H	-6.09806700	-2.52922400	-0.44318200
C	-4.50840100	-1.89100600	1.69144700
H	-5.15163100	-2.76784900	1.81684700
H	-3.62041900	-2.04393600	2.30822700
H	-5.04707400	-1.02045100	2.07854900
C	-3.43043100	-2.94366500	-0.37373000
H	-2.46820900	-3.12863900	0.10865600
H	-4.04411900	-3.83636700	-0.21787900
H	-3.26278300	-2.84877600	-1.45164700
C	-1.28951500	-0.97300500	1.35779900
H	-1.35499800	-0.40847300	2.29331100
C	-0.16725300	2.89065200	-0.08605100
O	0.22475000	2.49803300	-1.22643100
O	-0.98430700	2.36920700	0.68908200
C	0.52752400	4.20754600	0.37985900
F	-0.01831900	4.71675400	1.50289500
F	0.46458600	5.17018500	-0.57022200
F	1.84204600	3.98238000	0.63851800
H	-1.71654400	-1.95909900	1.53010000
O	-0.95853700	0.50433900	-2.06179900
H	-1.81513000	0.74981400	0.35279300
H	-0.39349600	1.35114300	-1.69428900
H	1.03805400	-1.56105000	2.76433200
O	1.69374100	-0.73663600	-2.39443800
C	2.62390700	-1.43594400	-3.24203300
H	2.24689900	-1.28879100	-4.25494700
H	3.62869900	-1.02432300	-3.14976900
H	2.63176300	-2.50479800	-3.00743700
O	3.96734200	-1.32840200	-0.75692200
O	3.60502200	-1.76962000	1.88126200
C	4.67901600	-2.58212600	-0.75902200
H	4.86398900	-2.92907300	0.25926000
H	4.11721100	-3.34019000	-1.31665500
H	5.62585800	-2.38402500	-1.26316700
C	3.48616500	-1.93580100	3.29869800
H	4.50114100	-2.10256300	3.65807800
H	3.07425800	-1.03513200	3.76569200
H	2.86276000	-2.80273400	3.54226200

9

C	-0.76441400	-1.03423300	-0.46395900
C	-0.55530600	-0.98817400	0.90574300
C	-1.60388300	-0.77958200	1.80015000
C	-2.88915300	-0.60876500	1.27279800
C	-3.12969100	-0.69086400	-0.12645600
C	-2.05434700	-0.91006400	-1.00670600
H	-1.42417000	-0.73596500	2.86687000

C	0.90132600	-1.20174100	1.22582300
H	1.34685500	-0.29424400	1.64429900
H	1.04827600	-2.02662000	1.92751100
N	1.47670100	-1.51273700	-0.10144800
C	2.75891000	-1.82664700	-0.46338900
O	3.03873300	-1.81039800	-1.67695300
C	3.82061300	-2.25422900	0.57937200
C	5.16196200	-2.44691400	-0.15663000
H	5.08147100	-3.19180800	-0.95140100
H	5.50862900	-1.51186700	-0.60590800
H	5.91662900	-2.78405600	0.56136700
C	3.40120200	-3.60997000	1.19882500
H	4.17572000	-3.94269700	1.89805600
H	2.46039300	-3.55047500	1.75199600
H	3.29313900	-4.37694600	0.42485500
C	4.02885900	-1.19193300	1.68500200
H	3.20072100	-1.13773300	2.39253900
H	4.92427200	-1.45381200	2.25817100
H	4.18561300	-0.19559000	1.25886900
C	0.52125000	-1.26511000	-1.19157500
O	0.89786000	-0.11280700	-1.97829500
H	0.50974600	-2.11924500	-1.87369600
C	1.13824700	2.78147400	-0.16555800
O	1.64178300	2.10970300	0.70927500
O	0.67642300	2.39169500	-1.33038000
C	0.97253400	4.31878200	-0.00817700
F	1.36652500	4.72455900	1.20893400
F	-0.31450900	4.69235600	-0.17645700
F	1.71678200	4.97495900	-0.92759200
H	0.78441400	1.39144000	-1.48104000
H	1.75904500	-0.35503000	-2.37110000
O	-4.38188800	-0.43415100	-0.63355700
C	-5.41533000	-1.39381000	-0.33821800
H	-5.62038100	-1.43140000	0.73343700
H	-6.30145400	-1.05162400	-0.87504000
H	-5.12896700	-2.38837000	-0.69870000
O	-2.14348200	-0.95217900	-2.36604000
O	-3.98747600	-0.33419400	2.02928800
C	-3.82745800	-0.18370200	3.44285800
H	-4.81997300	0.05247000	3.82600300
H	-3.46863500	-1.11193600	3.90089900
H	-3.13918100	0.63600700	3.67540900
C	-3.27576300	-1.55927000	-3.01374000
H	-4.14088700	-0.89589700	-3.00601700
H	-2.95177500	-1.74377300	-4.03901600
H	-3.52857200	-2.51137100	-2.53588300

9TS

C	-0.83248000	-1.18915800	-0.36054500
C	-0.57292000	-0.77541400	0.94968700
C	-1.58332100	-0.31474600	1.78051500
C	-2.88920300	-0.29379500	1.26126500
C	-3.17761600	-0.75278500	-0.05710900
C	-2.13947800	-1.18391800	-0.89238400
H	-1.37348000	0.01529600	2.78960800
C	0.89865300	-0.90848000	1.22792100
H	1.37175300	0.07147100	1.35236900
H	1.10380400	-1.53966400	2.09389900
N	1.39438000	-1.54584700	-0.02205000
C	2.70895600	-1.91261400	-0.38117300
O	2.94552900	-2.03111800	-1.57954100
C	3.76231500	-2.22562900	0.69741700
C	5.06524500	-2.62516600	-0.02396200
H	4.91456500	-3.48514100	-0.68097100
H	5.45491900	-1.80153500	-0.62834900
H	5.81819300	-2.88853100	0.72502300
C	3.27475400	-3.42654700	1.54625700
H	4.04553800	-3.67639700	2.28213500
H	2.35184500	-3.21781700	2.09287900
H	3.10766300	-4.30839800	0.91934400
C	4.06288800	-1.00146100	1.59771900
H	3.25297800	-0.76009400	2.28679500
H	4.94502500	-1.23027200	2.20375800
H	4.28485000	-0.11100200	1.00218000
C	0.41148000	-1.56111200	-0.98703800
O	0.78856900	-0.17868400	-2.23499000
H	0.50287000	-2.27285500	-1.79388000
C	1.27517700	2.62721300	-0.38762900
O	1.99805300	2.02953100	0.42063300
O	0.64279400	2.19153400	-1.39576100
C	1.07592400	4.16507500	-0.17763300
F	1.72466200	4.64058200	0.90773600
F	-0.23770600	4.47668700	-0.01972100
F	1.52532200	4.87194800	-1.24786400
H	0.77226000	0.78223700	-1.82325800
H	1.70138000	-0.38576200	-2.50069900
O	-4.47243800	-0.72497100	-0.51329700
C	-5.30752100	-1.80201900	-0.04267300
H	-5.39909300	-1.77450200	1.04670100
H	-6.28598900	-1.64461300	-0.49852500
H	-4.90220400	-2.76896700	-0.36130200
O	-2.28278700	-1.64639600	-2.15681000
O	-3.96812800	0.12668500	1.95667900
C	-3.78850900	0.62238800	3.29067300
H	-4.78259600	0.91405500	3.62713800
H	-3.39047400	-0.15801700	3.94733600
H	-3.12643400	1.49380400	3.29645400
C	-3.25027800	-1.05938900	-3.05534500
H	-3.28189900	0.02491400	-2.92755300
H	-2.89086300	-1.30638500	-4.05488300
H	-4.24288800	-1.48114300	-2.89428500

11

C	1.79431500	-1.01557600	0.04743900
C	0.94124900	-0.21199600	-0.74772500
C	1.31744700	1.04702800	-1.16003900
C	2.59842500	1.49424700	-0.76799900
C	3.49601600	0.68359600	0.00168800
C	3.08952100	-0.57530600	0.44507900
H	0.65598800	1.67327600	-1.74300400
C	-0.34146800	-0.95540600	-0.95541700
H	-1.18667300	-0.40370300	-0.52133600
H	-0.54976900	-1.14215800	-2.01073000
N	-0.09709600	-2.23019300	-0.23400900
C	-0.96022500	-3.37074600	-0.02202300
O	-0.53434100	-4.23830100	0.71598400
C	-2.31906500	-3.47853800	-0.73304100
C	-2.95195600	-4.81618000	-0.29472100
H	-2.32569900	-5.66848800	-0.57059400
H	-3.11045600	-4.84774300	0.78628900
H	-3.92173100	-4.92469500	-0.78902700
C	-2.12478900	-3.51297100	-2.27041500
H	-3.08952900	-3.73910100	-2.73488700
H	-1.78417200	-2.56306600	-2.68597500
H	-1.41869100	-4.29591800	-2.56550000
C	-3.27095200	-2.32892000	-0.32023800
H	-2.97301600	-1.35004900	-0.69622400
H	-4.26378400	-2.54684200	-0.72639000
H	-3.36044500	-2.26353100	0.76839700
C	1.11378100	-2.19768800	0.34216300
O	-0.84038100	-0.50029300	2.35405900
H	1.44376700	-3.03085700	0.94667600
C	-2.76346300	1.86920900	0.54761900
O	-2.55582200	1.20018900	-0.48419800
O	-2.36084400	1.70103500	1.72484500
C	-3.64571700	3.15431600	0.36996200
F	-4.26253000	3.22731500	-0.83295700
F	-2.88434900	4.28019700	0.48554300
F	-4.62015700	3.24276500	1.31180000
H	-1.39072200	0.28716300	2.11770100
H	-1.14726000	-0.77075200	3.22845300
O	4.72171400	1.19192700	0.34433100
C	5.71526800	1.16671200	-0.70276900
H	5.39772000	1.77389000	-1.55447200

H	6.61987000	1.58994500	-0.26487000
H	5.90534200	0.13690400	-1.02515400
O	3.79397000	-1.44569800	1.18845900
O	3.08858200	2.69734700	-1.08783000
C	2.28266100	3.62444900	-1.83979900
H	2.89802000	4.51651400	-1.94292200
H	2.04741700	3.21768200	-2.82733000
H	1.36562600	3.86505100	-1.29553600
C	4.85377400	-1.02256300	2.07779600
H	4.54580900	-0.14439400	2.64710600
H	5.00516100	-1.87182700	2.74380700
H	5.76826300	-0.81011000	1.52353900

11TS

C	-1.43101300	-1.24103800	0.15303500
C	-0.76852000	-0.28445700	-0.67631100
C	-1.43530100	0.87112200	-1.11192400
C	-2.75781400	1.03465000	-0.71288600
C	-3.45037600	0.06670400	0.11095200
C	-2.78538000	-1.05673500	0.57210100
H	-0.92574700	1.60618100	-1.72015900
C	0.59971500	-0.70306400	-0.83214200
H	1.09644000	-0.60924100	-1.79031200
H	1.28918200	0.27411200	-0.04164300
N	0.64986900	-2.01230100	-0.21522300
C	1.69977100	-2.99791200	-0.16643300
O	1.38731100	-4.10093000	0.24614400
C	3.15671600	-2.65765700	-0.53570700
C	3.92325700	-3.99390600	-0.63815400
H	3.85602400	-4.56938900	0.28699600
H	3.53615900	-4.61371400	-1.45291800
H	4.97715800	-3.78093100	-0.84007600
C	3.75152900	-1.82511500	0.63029100
H	4.80505200	-1.62020900	0.41260000
H	3.24309800	-0.86783700	0.76089500
H	3.70242700	-2.38033100	1.57250500
C	3.32493500	-1.89882400	-1.87363500
H	3.09257300	-0.83673700	-1.79296400
H	4.37393200	-1.97007700	-2.17597200
H	2.72153500	-2.33805800	-2.67483500
C	-0.53400700	-2.27317000	0.40053700
H	-0.65598200	-3.16505100	0.99489200
C	2.05037600	2.20882500	-0.01780300
O	1.84906900	1.10383600	0.62309200
O	1.73848700	2.50375300	-1.16278000
C	2.81148000	3.24492100	0.86490100
F	2.15024900	3.48926100	2.02129300
F	2.97248500	4.42205800	0.23336400
F	4.04428500	2.78476700	1.19143200
O	-4.75662800	0.31514800	0.46038200
C	-5.72761300	0.00267100	-0.55772200
H	-6.70069300	0.25338600	-0.13273700
H	-5.55358000	0.59946000	-1.45751000
H	-5.69574500	-1.06473300	-0.80470000
O	-3.28820800	-2.04284900	1.35316800
O	-3.52538100	2.08958200	-1.05789400
C	-2.96078900	3.11779000	-1.88378300
H	-2.10925100	3.59249400	-1.38670600
H	-2.65089900	2.71128000	-2.85176900
H	-3.75842100	3.84583200	-2.02606200
C	-4.33224900	-1.77995500	2.31326800
H	-5.30938500	-1.72555000	1.83162100
H	-4.29886300	-2.62619500	3.00039600
H	-4.13277600	-0.85078200	2.85183200

4

C	1.10661600	-1.33488100	-0.39029100
C	0.61845600	-0.66518600	0.79701400
C	1.47455900	0.19897400	1.54657100
C	2.76652500	0.35719200	1.10302200
C	3.27986700	-0.33275200	-0.07585400
C	2.45946000	-1.14440900	-0.82783700
H	1.10460200	0.71005500	2.42594100
C	-0.71189100	-1.03598400	0.95150200
H	-1.39100300	-0.81639500	1.75463700
H	-0.94603300	0.88571800	-0.06521100
N	-1.02925700	-1.93137700	-0.08241400
C	-2.24900800	-2.62391400	-0.36787600
O	-2.29471900	-3.26829800	-1.40169400
C	-3.43561700	-2.57601900	0.61678900
C	-4.55033900	-3.47276400	0.03846600
H	-4.88663500	-3.11524400	-0.93812300
H	-4.21406000	-4.50622400	-0.07755000
H	-5.40357100	-3.46156500	0.72361700
C	-4.00191700	-1.13927700	0.74641400
H	-4.91391200	-1.17733500	1.35060200
H	-3.31916000	-0.43905000	1.22858100
H	-4.26860500	-0.73440400	-0.23528000
C	-3.02213200	-3.15008100	1.99519300
H	-2.26091300	-2.55718900	2.50415400
H	-3.90531900	-3.18479500	2.64105700
H	-2.64201700	-4.17191600	1.89267500
C	0.07709500	-2.09657200	-0.90270400
H	0.02264200	-2.73095200	-1.77036400
C	-1.64996200	2.65397500	0.09683200
O	-1.19331400	1.65358600	-0.63995700
O	-1.77762700	2.68910800	1.29746100
C	-2.02641200	3.85452700	-0.81335900
F	-0.94625700	4.29551100	-1.49266500
F	-2.50704400	4.87093500	-0.08470000
F	-2.96747800	3.49739400	-1.71339300
O	4.59073100	-0.11724200	-0.43836200
C	5.55235500	-0.89774000	0.29537800
H	6.53155700	-0.62453600	-0.10164900
H	5.51023600	-0.66377600	1.36337800
H	5.37660100	-1.96865400	0.14076700
O	2.81459600	-1.84047000	-1.94394500
O	3.69820400	1.14533300	1.70293500
C	3.31658300	1.87023900	2.87411000
H	2.50000100	2.56690200	2.65474700
H	3.01451200	1.18797600	3.67668300
H	4.20260100	2.42700100	3.17856800
C	3.72340200	-1.26280600	-2.90065000
H	4.76122900	-1.36824100	-2.58095000
H	3.55635700	-1.81864300	-3.82463400
H	3.49388900	-0.20491700	-3.05574300

4TS

C	0.80593000	-0.92507800	0.45196000
C	0.98748000	-1.40954900	-0.87203600
C	2.25823100	-1.41236100	-1.50140000
C	3.32786000	-0.94337700	-0.76747600
C	3.15996600	-0.44168800	0.58217600
C	1.90341700	-0.42666500	1.18733700
H	2.37294200	-1.78807100	-2.50942200
C	-0.27331800	-1.82659700	-1.31661500
H	-0.56882500	-2.22806700	-2.27430900
N	-1.19509300	-1.64250500	-0.34214800
C	-2.56874300	-2.03311800	-0.56794400
O	-2.85552700	-2.39798000	-1.69157400
C	-3.57753300	-2.06110100	0.59444000
C	-4.92487600	-2.54534200	0.01944500
H	-5.30171800	-1.86086100	-0.74526500
H	-4.83825400	-3.53861900	-0.42781100
H	-5.65735500	-2.59328400	0.83078800
C	-3.79586100	-0.66170400	1.21851300
H	-4.61504400	-0.73338100	1.94092000
H	-2.92458600	-0.27720600	1.74710900
H	-4.08458500	0.06923000	0.45698400
C	-3.10583800	-3.08137000	1.66241700
H	-2.16657600	-2.80133900	2.14308100
H	-3.87029600	-3.14887800	2.44277400
H	-2.98152900	-4.07754100	1.22558400
C	-0.59803800	-0.94268200	0.76981800
H	-0.94618600	0.39251600	0.63932700

C	-1.39873000	2.03949900	-0.61155800
O	-1.22658200	1.60601000	0.59013200
O	-1.33683600	1.43137000	-1.67277900
C	-1.72919300	3.56418500	-0.62415700
F	-1.90648800	4.03559200	-1.87264200
F	-0.73090100	4.28589900	-0.05684000
F	-2.86267000	3.82512500	0.07276400
H	-0.94377200	-1.20661900	1.76230600
O	4.25311200	-0.09380500	1.32166700
C	5.01461100	1.06329900	0.90662400
H	5.80082800	1.17711100	1.65346900
H	5.45206700	0.91089600	-0.08051700
H	4.37534700	1.95244600	0.90458700
O	4.61698200	-0.93939900	-1.19961300
O	1.63746400	-0.04034800	2.46735700
C	4.89452500	-1.44625800	-2.50867900
H	4.37854400	-0.85709700	-3.27454200
H	5.97245200	-1.35273900	-2.63707500
H	4.60284900	-2.49913400	-2.58927100
C	2.26862900	1.13029300	3.02094700
H	2.26335200	1.94762300	2.29296800
H	1.65701100	1.40656400	3.88071100
H	3.28949500	0.91535200	3.33871200

12

C	0.69930300	-1.03123600	0.58962900
C	0.88717800	-1.68128800	-0.64712000
C	2.14958000	-1.77410800	-1.26250300
C	3.23295600	-1.21072300	-0.60303100
C	3.06319100	-0.59508700	0.68600200
C	1.77349000	-0.47145400	1.26057300
H	2.25520800	-2.23982300	-2.23319800
C	-0.37848900	-2.13641300	-1.06911800
H	-0.63305700	-2.67814000	-1.96793400
N	-1.32568100	-1.86128800	-0.16537500
C	-2.69326300	-2.27668600	-0.40047100
O	-2.92641500	-2.77918300	-1.48153500
C	-3.74932200	-2.15435100	0.70996500
C	-5.10172400	-2.57614200	0.09851000
H	-5.39984400	-1.90296200	-0.71024200
H	-5.06472200	-3.59193000	-0.30151600
H	-5.86798500	-2.53745100	0.87809400
C	-3.89030500	-0.71446000	1.25997700
H	-4.79403300	-0.67326000	1.87535300
H	-3.05808200	-0.40630100	1.89236900
H	-4.00042600	0.01684000	0.45408500
C	-3.38987800	-3.14612200	1.84763300
H	-2.44912900	-2.90407200	2.34763000
H	-4.18159400	-3.11134000	2.60236000
H	-3.32574300	-4.17184600	1.47112700
C	-0.75216300	-1.04648300	0.93959100
O	1.59421200	0.08994800	2.49283400
C	-1.09013000	2.66650600	-0.61544500
O	-1.41508400	1.98866000	0.38090000
O	-0.80581800	2.32185900	-1.78716300
C	-1.05065900	4.21175200	-0.34258300
F	-2.29912800	4.68946700	-0.07739900
F	-0.56732000	4.94271600	-1.37311300
F	-0.28093400	4.50995700	0.73925800
H	-0.96737500	-1.48596900	1.91409600
O	-0.92054300	-0.16272800	-2.92383600
H	-0.89490600	0.71735400	-2.47097400
H	-1.76551700	-0.18769700	-3.39079000
H	-1.16224400	-0.02685500	0.87738500
C	1.72024800	1.53156400	2.55993600
H	1.54070300	1.79235800	3.60324000
H	2.72454400	1.84473500	2.26514400
H	0.96581900	2.00265500	1.92311200
O	4.06557200	-0.04524900	1.39080400
O	4.49407500	-1.16945800	-1.10022500
C	5.39107200	-0.62759100	1.44509100
H	5.32815400	-1.71751200	1.47062900
H	5.99000500	-0.30385700	0.59517200
H	5.81537100	-0.25682900	2.37820300
C	4.74297800	-1.75206500	-2.38595000
H	5.80426800	-1.59609500	-2.57506400
H	4.52245800	-2.82478100	-2.37774200
H	4.15195000	-1.25191500	-3.15998800

12TS

C	0.54737200	-1.03274300	0.47638600
C	0.63530200	-1.34409100	-0.88205100
C	1.85646300	-1.43802200	-1.55965100
C	3.01993700	-1.21991900	-0.82129300
C	2.94928000	-0.93865300	0.57598400
C	1.70560800	-0.82015500	1.22276600
H	1.88503100	-1.64910500	-2.62059300
C	-0.71434100	-1.50515000	-1.38413400
H	-0.99130500	-2.09908500	-2.24252000
N	-1.58243100	-1.44018700	-0.33173200
C	-2.97158700	-1.60632700	-0.57655500
O	-3.32953000	-1.57861000	-1.74703800
C	-3.94745500	-1.88450000	0.57916800
C	-5.35643500	-2.03629600	-0.02910400
H	-5.68166400	-1.11349000	-0.51704200
H	-5.39107000	-2.84063600	-0.76789600
H	-6.06405600	-2.27029400	0.77186900
C	-3.98469100	-0.72778300	1.60896600
H	-4.82766800	-0.89976100	2.28520800
H	-3.08636500	-0.66546000	2.22357100
H	-4.13669100	0.23928700	1.12048000
C	-3.55412900	-3.21840400	1.26269500
H	-2.56801300	-3.18567600	1.73201300
H	-4.28528200	-3.44218200	2.04578000
H	-3.56432300	-4.04525800	0.54524300
C	-0.88711100	-0.99696500	0.90918100
O	1.60360700	-0.58838500	2.56804200
C	-0.86095300	2.70986400	-0.42080500
O	-1.60805900	2.13736500	0.38419000
O	-0.38197700	2.30374400	-1.52126700
C	-0.39438400	4.16040400	-0.06412800
F	0.95353500	4.20870400	0.11466900
F	-0.95671700	4.63618400	1.06834700
F	-0.69445700	5.04055800	-1.05371700
H	-1.09724700	-1.67803700	1.73476500
O	-0.98379200	0.05999500	-2.51800100
H	-0.77288300	0.96448700	-2.04668800
H	-1.93906800	0.02743100	-2.69446400
H	-1.21024600	0.01718100	1.16678800
C	2.01613900	0.71911500	3.02672500
H	1.83459900	0.72429900	4.10190600
H	3.07606700	0.88431000	2.82302600
H	1.41096800	1.49450600	2.54597600
O	4.08353000	-0.71100300	1.29881900
O	4.27546400	-1.25341700	-1.33724000
C	4.93539800	-1.85329600	1.53935200
H	4.38475400	-2.62091600	2.09278300
H	5.31624200	-2.25961600	0.59991200
H	5.76076400	-1.48138100	2.14686200
C	4.43107600	-1.50711200	-2.73832400
H	5.50424400	-1.47560900	-2.92316000
H	4.04013600	-2.49534500	-3.00369300
H	3.93198700	-0.73466000	-3.33279400

14

C	0.44751800	-1.26158500	0.31716600
C	0.52834500	-1.04417000	-1.05054500
C	1.74900400	-0.92927900	-1.71713600
C	2.92259600	-1.04941900	-0.96380100
C	2.85768600	-1.30026000	0.43227000
C	1.61403700	-1.38806200	1.07733700
H	1.77922400	-0.74305000	-2.78335200
C	-0.84853100	-0.95398100	-1.63729700
H	-1.04569000	-1.68681300	-2.42607200
N	-1.72634500	-1.22315300	-0.49103700

C	-3.07006700	-1.26739600	-0.74544300
O	-3.44852000	-0.97729100	-1.89676800
C	-4.09466600	-1.72435200	0.32038400
C	-5.50871400	-1.50421400	-0.25467100
H	-5.69929500	-0.44417900	-0.44655400
H	-5.65107600	-2.04618100	-1.19195500
H	-6.24764900	-1.86037500	0.47026900
C	-3.98816200	-0.92997800	1.64386100
H	-4.86869500	-1.15287700	2.25500600
H	-3.11245000	-1.19358700	2.23837000
H	-3.97282400	0.15002200	1.46414000
C	-3.89675000	-3.23882700	0.57839600
H	-2.91469200	-3.47194100	0.99850200
H	-4.65250800	-3.58564500	1.29114100
H	-4.01679900	-3.81187900	-0.34694600
C	-0.98043200	-1.32545500	0.78439200
O	1.50963600	-1.66153400	2.41945500
C	-0.55126600	2.88990400	0.03026300
O	-1.12375200	2.19480800	0.84113700
O	-0.29214700	2.62697600	-1.23036500
C	-0.00832300	4.29616700	0.40793000
F	-0.23147800	4.56810200	1.70249800
F	-0.60779200	5.25714000	-0.33072100
F	1.32258300	4.37632300	0.18760700
H	-1.20353300	-2.26109600	1.30330900
O	-1.07993100	0.34627600	-2.21498500
H	-0.63151600	1.71374200	-1.51472200
H	-2.00815900	0.31381800	-2.52103400
H	-1.22024600	-0.48955700	1.44776000
C	1.96601800	-0.62584500	3.31452000
H	1.77705000	-0.99881000	4.32206500
H	3.03320300	-0.43688100	3.17828600
H	1.39506200	0.29449700	3.14965600
O	4.00871500	-1.39990400	1.17429400
O	4.18024900	-0.95075300	-1.47766700
C	4.71884000	-2.64699500	1.04188200
H	4.08495300	-3.48164200	1.36077900
H	5.04995900	-2.79781200	0.01059500
H	5.58539500	-2.57111700	1.70030200
C	4.32471200	-0.69861400	-2.87807500
H	5.39844600	-0.65241600	-3.05868900
H	3.88662500	-1.50876600	-3.47172300
H	3.86473700	0.25607600	-3.15573100

14TS

C	0.15287900	-1.24479800	0.29323400
C	0.27859800	-0.70777900	-0.98328500
C	1.50961000	-0.67303800	-1.64903100
C	2.63431400	-1.21489000	-1.01732900
C	2.51544100	-1.79700100	0.26894500
C	1.27642900	-1.78660600	0.92927300
H	1.57935400	-0.23167100	-2.63502000
C	-0.96716500	-0.21746700	-1.65169300
H	-1.59989800	-0.96661300	-2.12990900
N	-1.97576800	-0.20223400	0.08844400
C	-3.39938800	-0.16677600	-0.10912200
O	-3.87093300	0.93828400	-0.31451000
C	-4.26299900	-1.44341700	-0.11544100
C	-5.64172900	-1.07174800	-0.70031000
H	-6.13909400	-0.30694800	-0.09981300
H	-5.55241500	-0.69391300	-1.72272300
H	-6.27370000	-1.96504500	-0.71719700
C	-4.47300300	-1.92153300	1.34675700
H	-5.18417600	-2.75363000	1.34244700
H	-3.55814000	-2.27358200	1.82762900
H	-4.89590500	-1.12074100	1.96142200
C	-3.65695200	-2.56847400	-0.98682500
H	-2.67383000	-2.90991500	-0.65649600
H	-4.32510100	-3.43453900	-0.95130900
H	-3.58125200	-2.25885100	-2.03436200
C	-1.21998300	-1.17660700	0.91519300
H	-1.18300700	-0.83517700	1.95345200
C	0.04624800	2.90246000	0.08842500
O	0.25529200	2.73456500	-1.15534900
O	-0.67807200	2.25579900	0.85630400
C	0.85303900	4.09858300	0.67937500
F	0.60893800	4.29050400	1.99014300
F	0.55679200	5.25517700	0.03868100
F	2.18699400	3.89206700	0.54303200
H	-1.70796000	-2.14928000	0.90347400
O	-1.03802500	0.94585400	-2.19732700
H	-1.67421200	0.75750000	0.34031300
H	-0.41783200	1.73268400	-1.71638400
O	3.61505900	-2.30859300	0.90716300
C	4.07350300	-3.58716300	0.42138000
H	4.38564000	-3.51621300	-0.62397900
H	4.92739000	-3.85490800	1.04493500
H	3.28495600	-4.33936300	0.53121900
O	3.88237500	-1.23398200	-1.55470800
O	1.10518500	-2.35076000	2.16776200
C	4.07726200	-0.66525700	-2.85429200
H	5.13804200	-0.78835900	-3.07052500
H	3.48433900	-1.19497800	-3.60791300
H	3.82271300	0.39983700	-2.85916400
C	1.81907500	-1.73793300	3.26484700
H	1.51514800	-2.28722100	4.15663000
H	2.89775900	-1.81950100	3.12003400
H	1.52891100	-0.68633100	3.36212400

2

C	-1.55827200	-0.82511700	-0.38608800
C	-0.23813300	-0.34782200	-0.56671200
C	0.86999200	-1.20209700	-0.41367100
C	0.69117700	-2.54392300	-0.09597700
C	-0.62368700	-3.04918000	0.05620700
C	-1.72805900	-2.18278900	-0.05923600
H	1.86398900	-0.79778000	-0.54727500
C	-0.00829600	1.05512800	-0.91991900
H	-0.89853600	1.67559200	-1.08365900
N	-2.88159200	0.98987500	0.60038900
C	-3.71961400	2.04507300	0.85985800
O	-3.69598400	2.53000000	1.99906200
C	-4.62606100	2.65292300	-0.24078500
C	-5.46320400	3.77883400	0.39819700
H	-6.10299100	3.39570900	1.19788100
H	-4.82720700	4.55880000	0.82358200
H	-6.10143900	4.23011600	-0.36877700
C	-5.59852700	1.60110800	-0.82534800
H	-6.31989500	2.10680700	-1.47580800
H	-5.10087600	0.83959600	-1.42810300
H	-6.16188300	1.09814700	-0.03188000
C	-3.75459800	3.27294200	-1.35951500
H	-3.17583000	2.53055600	-1.91534500
H	-4.40224700	3.78248800	-2.08115100
H	-3.05886100	4.01286600	-0.95065800
C	-2.78659000	0.05589900	-0.53352800
H	-3.66120800	-0.59469000	-0.57853400
C	4.21472200	1.81181900	0.24467000
O	3.66670500	1.21472200	-0.79381700
O	3.66631700	2.43174600	1.12738100
C	5.75672000	1.62420200	0.21637300
F	6.33202700	2.19981900	1.28149700
F	6.28725900	2.17648700	-0.89713200
F	6.08346000	0.31168700	0.22200700
H	-2.74071100	0.61039700	-1.47260800
O	1.09509600	1.59640100	-1.05082000
H	-2.38699000	0.70199900	1.43775700
H	2.66641400	1.34049200	-0.81955100
O	-0.85073200	-4.34952600	0.38637400
C	-0.51509400	-5.34571400	-0.60708800
H	0.55440100	-5.33459300	-0.82525300
H	-0.79866400	-6.30216800	-0.16782200
H	-1.09448500	-5.17037200	-1.51905500
O	1.69342300	-3.43927200	0.08913300

O	-2.99486900	-2.69360200	0.06593000
C	3.04778400	-2.99257500	-0.05689500
H	3.66555200	-3.86799000	0.13971700
H	3.23350000	-2.63033300	-1.07342400
H	3.28078100	-2.20736900	0.66966200
C	-3.40298100	-3.06598400	1.39979400
H	-4.42173100	-3.44288400	1.30321900
H	-2.75272200	-3.84637800	1.80121400
H	-3.39526600	-2.19019100	2.05782800

4e-22b TS

C	0.30892300	1.09716400	-1.64202700
C	1.54509600	1.72992400	-1.29400000
C	1.79935300	3.08328100	-1.62682300
C	0.82545800	3.76442000	-2.32918200
C	-0.39123000	3.12815400	-2.70233500
C	-0.66586400	1.81169000	-2.36527000
H	2.73405300	3.55942000	-1.34734900
H	0.98627900	4.79973400	-2.61335400
C	2.33776400	0.75911900	-0.66148100
H	3.32383600	0.87582100	-0.24460400
N	1.65711300	-0.41689500	-0.62082400
C	2.09006900	-1.72331900	-0.17553400
O	1.21131000	-2.55253400	-0.03641200
C	3.57963000	-2.03348300	0.05219100
C	3.69796100	-3.55501000	0.27796100
H	3.12325400	-3.88211000	1.14820200
H	3.35060400	-4.11927600	-0.59241400
H	4.75040400	-3.80105800	0.44863500
C	4.09964000	-1.31811100	1.32587500
H	5.13193500	-1.63825500	1.49956800
H	4.10465900	-0.22906100	1.25031900
H	3.50988700	-1.60113000	2.20386500
C	4.42346000	-1.66074400	-1.19061600
H	4.49325700	-0.58593800	-1.36936100
H	5.44096100	-2.03536400	-1.04208100
H	4.02238400	-2.13277500	-2.09388600
C	0.33329900	-0.22836900	-1.10955000
H	-0.15503500	-1.08370700	-1.55172300
C	-1.23183800	1.87728300	1.55281500
C	-0.34589600	0.78381500	1.53631900
C	0.84605600	0.80792100	2.27390300
C	1.13305800	1.95175700	3.02344400
C	0.25734300	3.04631700	3.02214500
C	-0.93717600	3.01812400	2.28839100
H	1.51798100	-0.04333800	2.28827900
H	2.04230500	1.99151500	3.61546600
C	-0.91441600	-0.25276700	0.68365200
H	-0.67138900	-1.30104100	0.78073100
N	-2.19619300	0.12872500	0.35720000
C	-3.28141600	-0.59076100	-0.20448200
O	-4.21088300	0.08527700	-0.62350800
C	-3.35203600	-2.13537400	-0.16774500
C	-4.75108500	-2.54078500	-0.67481900
H	-5.54588100	-2.10723200	-0.06188800
H	-4.91246900	-2.22513300	-1.70932200
H	-4.83727100	-3.63120200	-0.63042100
C	-3.19795200	-2.62870200	1.29098300
H	-3.36624300	-3.71036000	1.31813600
H	-2.20359800	-2.44101300	1.70355000
H	-3.93719700	-2.15701500	1.94764400
C	-2.30160400	-2.80296900	-1.08383600
H	-1.28055400	-2.71247100	-0.70919000
H	-2.52393600	-3.87358200	-1.14322900
H	-2.34445900	-2.39877200	-2.10125300
C	-2.44287700	1.54873100	0.73243400
H	-3.37531800	1.60851400	1.30093800
H	-2.54992300	2.17674900	-0.15518200
H	-1.61831700	3.86374000	2.31048100
H	0.50102400	3.92575900	3.61104600
H	-1.12338800	3.69485300	-3.27031600
H	-1.60001400	1.34513100	-2.66103100

22b

C	0.14885300	1.21259400	-1.50836600
C	1.45207200	1.76283700	-1.45276200
C	1.76278100	3.02513400	-1.99172200
C	0.73344200	3.72320800	-2.60762000
C	-0.55799000	3.16564700	-2.69039400
C	-0.86710700	1.91151400	-2.14684500
H	2.76998800	3.42478400	-1.93527900
H	0.92176700	4.69921500	-3.04257700
C	2.29483600	0.78911800	-0.86768500
H	3.36012800	0.87550600	-0.71426400
N	1.61848500	-0.30870400	-0.54593100
C	2.13637400	-1.60420000	-0.12950300
O	1.30370800	-2.47608000	-0.00393200
C	3.64201600	-1.82862800	0.08902900
C	3.81200500	-3.28792000	0.56001100
H	3.27604500	-3.47684700	1.49429900
H	3.45334500	-3.99858600	-0.18914300
H	4.87619200	-3.47478700	0.73047000
C	4.20731600	-0.89843500	1.19058900
H	5.25266500	-1.17282800	1.36278900
H	4.19037000	0.16316000	0.93554500
H	3.66697200	-1.03394600	2.13252000
C	4.40710900	-1.66507500	-1.25020900
H	4.45542800	-0.63572500	-1.61279900
H	5.43662900	-2.00421900	-1.09919800
H	3.96226700	-2.28537500	-2.03555300
C	0.15710700	-0.13014100	-0.83689400
H	-0.14537800	-0.92721700	-1.51771200
C	-1.32795400	1.74809900	1.64970800
C	-0.34278900	0.76251300	1.54943100
C	0.78535400	0.79983300	2.37240800
C	0.92245900	1.85657400	3.28030900
C	-0.05327500	2.85802000	3.35973800
C	-1.19119500	2.80697500	2.54691000
H	1.54151700	0.02226200	2.33532900
H	1.78941400	1.89344200	3.93363100
C	-0.72801300	-0.24992000	0.48694400
H	-0.61818900	-1.27650500	0.83183200
N	-2.13396400	0.08929400	0.21523100
C	-3.19621400	-0.68951500	-0.21485300
O	-4.26667000	-0.11019100	-0.42623400
C	-3.13013300	-2.23942900	-0.31620300
C	-4.47282100	-2.72812300	-0.89749400
H	-5.32054900	-2.40377200	-0.29005900
H	-4.62814900	-2.35713200	-1.91547300
H	-4.46691100	-3.82304600	-0.92970300
C	-2.98463900	-2.81421200	1.11468700
H	-3.00767500	-3.90868900	1.06847700
H	-2.04795200	-2.52442200	1.59900900
H	-3.81218700	-2.48699300	1.75363100
C	-2.00582500	-2.77204800	-1.22934800
H	-1.01146000	-2.67890400	-0.79176700
H	-2.17039800	-3.84142800	-1.40076200
H	-2.01636900	-2.28005000	-2.20890800
C	-2.47971900	1.43753900	0.73851400
H	-3.43309800	1.38003700	1.27061700
H	-2.60339700	2.17443700	-0.06249000
H	-1.96034000	3.57035300	2.62718000
H	0.06529100	3.67102700	4.07031000
H	-1.33958700	3.72347200	-3.19762900
H	-1.86863900	1.50705800	-2.23635700

4e-22a TS

C	0.44023300	-1.73079700	1.08570200
C	-0.42602100	-1.14319900	2.06744600
C	-0.02787500	-1.03774700	3.42436600
C	1.20699500	-1.54472700	3.77541900
C	2.05664300	-2.15233800	2.80675000

C	1.69618800	-2.24799000	1.47332300
H	-0.68287800	-0.58403700	4.16205900
H	1.53956200	-1.49288200	4.80766400
C	-1.61515000	-0.79367900	1.40935500
H	-2.48721300	-0.30926000	1.81486900
N	-1.50761900	-1.13071800	0.09586000
C	-2.49001900	-1.07654700	-0.96877000
O	-2.06089000	-1.24759400	-2.09171300
C	-3.98586200	-0.89494700	-0.65834900
C	-4.75137200	-1.11906800	-1.97869000
H	-4.45458200	-0.39806700	-2.74469200
H	-4.58575400	-2.12525600	-2.37482300
H	-5.82192200	-0.99758100	-1.78757600
C	-4.28811000	0.54314100	-0.16840800
H	-5.37299000	0.64503100	-0.06013900
H	-3.83997200	0.78407100	0.79745700
H	-3.94597500	1.28514400	-0.89495200
C	-4.46427800	-1.95051600	0.36804500
H	-4.05552300	-1.80118600	1.36940100
H	-5.55453800	-1.88905900	0.44460000
H	-4.20913100	-2.96440500	0.04054500
C	-0.21013300	-1.63270200	-0.17209200
H	-0.08469900	-2.27992300	-1.02621700
C	3.06129600	0.15897900	-0.71622300
C	2.13117900	-0.53336200	-1.50954600
C	2.52387500	-1.59045500	-2.34253200
C	3.87416700	-1.93334600	-2.36438900
C	4.80458900	-1.24230800	-1.56688900
C	4.40903900	-0.18956100	-0.73439600
H	1.80121800	-2.12036500	-2.95541900
H	4.21513700	-2.74107600	-3.00465200
C	0.82588100	0.04770700	-1.28245400
H	-0.01617800	0.00314300	-1.95932800
N	0.96661400	1.15555900	-0.48821200
C	-0.09809800	2.09919700	-0.39543300
O	-1.13680100	1.81813200	-0.97421300
C	0.07809100	3.44331500	0.34311600
C	-1.32957900	4.04142200	0.54587500
H	-1.85757600	4.16844800	-0.40152200
H	-1.94336200	3.40868200	1.19540100
H	-1.22992700	5.02197000	1.02139500
C	0.88580600	4.39972000	-0.57211300
H	0.95270000	5.37887000	-0.08618200
H	1.90593200	4.05172500	-0.75669300
H	0.38796300	4.53381200	-1.53800400
C	0.75066600	3.31792900	1.72941100
H	1.82653200	3.14651000	1.67763900
H	0.60425600	4.25944100	2.26825200
H	0.29690500	2.52089200	2.32832700
C	2.35508000	1.24683300	0.03999300
H	2.36820900	1.07463700	1.12006300
H	2.77668300	2.23277700	-0.16287600
H	5.13803000	0.34159300	-0.12967600
H	5.85164400	-1.52824400	-1.60361100
H	3.01255000	-2.55390100	3.13060400
H	2.35407200	-2.71942300	0.75085900

22a

C	0.40397700	-1.60599000	1.19073400
C	-0.66849500	-1.38043600	2.08436700
C	-0.54671800	-1.56692000	3.47213300
C	0.68563700	-1.99337600	3.95162600
C	1.75211900	-2.23268800	3.06419300
C	1.62852000	-2.04442100	1.68084200
H	-1.38643300	-1.38968600	4.13637800
H	0.82928100	-2.15485800	5.01500500
C	-1.80471400	-1.03521100	1.30450200
H	-2.80006300	-0.85011800	1.68078800
N	-1.51402600	-1.00635700	0.01562500
C	-2.41594000	-0.91326900	-1.13070500
O	-1.91045200	-1.18050600	-2.19767800
C	-3.90686200	-0.57980200	-0.95422100
C	-4.48473600	-0.37068600	-2.36992500
H	-3.99726700	0.46514500	-2.88038000
H	-4.37290800	-1.26337300	-2.99005100
H	-5.55129800	-0.14366700	-2.28098600
C	-4.14877300	0.71233400	-0.14075600
H	-5.21759500	0.94372100	-0.18538400
H	-3.88430300	0.63039600	0.91517600
H	-3.59331700	1.55226400	-0.56369200
C	-4.62600500	-1.79800300	-0.31288700
H	-4.32226500	-1.99343600	0.71912600
H	-5.70157000	-1.59527700	-0.30560800
H	-4.45849600	-2.70831200	-0.89813200
C	-0.06310000	-1.31806600	-0.20998100
H	-0.02626900	-2.20989900	-0.84232000
C	3.06727100	0.09688800	-0.79843200
C	2.07374700	-0.66375100	-1.41417600
C	2.39963300	-1.69815800	-2.29221400
C	3.75081200	-1.95932500	-2.54422800
C	4.75078100	-1.19784500	-1.92277800
C	4.41631100	-0.16098800	-1.04445300
H	1.62594600	-2.28572900	-2.77915500
H	4.02652900	-2.75513500	-3.22994500
C	0.70595100	-0.18617000	-0.98903900
H	0.07872500	0.08118500	-1.84041500
N	1.00540000	1.02825600	-0.20150900
C	0.04616900	2.01152400	-0.14548200
O	-1.05745700	1.78891800	-0.66367400
C	0.30888100	3.37902900	0.54254000
C	-1.06401200	4.01159700	0.85627700
H	-1.67220500	4.12569000	-0.04312000
H	-1.62904100	3.40485300	1.57217100
H	-0.90737500	5.00056400	1.29965400
C	1.04735900	4.30577700	-0.45442100
H	1.17076900	5.29630100	-0.00242800
H	2.04245500	3.93962300	-0.72251900
H	0.47018000	4.42556700	-1.37747500
C	1.08769000	3.27341300	1.87311500
H	2.15028300	3.06353300	1.74443300
H	1.01399000	4.23188400	2.39775800
H	0.66068700	2.50611600	2.52901200
C	2.45587900	1.14560700	0.09239100
H	2.66409500	0.94581300	1.14954400
H	2.82791100	2.14224100	-0.14692400
H	5.19395200	0.43085200	-0.56926900
H	5.79563700	-1.41036600	-2.13031700
H	2.69982700	-2.58201700	3.46310500
H	2.46549700	-2.25085300	1.02384600

mon1

C	-1.92645500	0.78219900	-0.00013900
C	-1.69050800	-0.65376300	0.00032700
C	-2.79818700	-1.55781800	0.00044800
C	-4.06851300	-1.03847900	0.00006900
C	-4.30257200	0.38167700	-0.00043700
C	-3.26627100	1.28146100	-0.00054400
H	-2.63175000	-2.63172400	0.00082900
H	-4.92527100	-1.70686100	0.00014600
C	-0.31981700	-0.84654900	0.00060500
H	0.24250500	-1.76163300	0.00088500
N	0.28516200	0.40803800	0.00033100
C	1.67616400	0.75755800	0.00054700
O	1.94988700	1.94473000	0.00124600
C	2.76871000	-0.33162700	-0.00026400
C	4.13438700	0.38492500	-0.00096400
H	4.25960800	1.01544600	-0.88567800
H	4.26057900	1.01532000	0.88369600
H	4.92803000	-0.36916100	-0.00145100
C	2.68518100	-1.19713900	-1.28189100
H	3.54600300	-1.87404300	-1.30296300
H	1.78284500	-1.80720800	-1.34194400
H	2.73017500	-0.56972200	-2.17891400

C	2.68668000	-1.19748200	1.28122700
H	1.78405400	-1.80701300	1.34251200
H	3.54714000	-1.87489100	1.30077800
H	2.73341200	-0.57034700	2.17835700
C	-0.68928800	1.40095400	-0.00008900
H	-0.40763700	2.44075000	-0.00026100
H	-5.32852200	0.73958600	-0.00073800
H	-3.45345900	2.35189200	-0.00090300

mon2

C	1.98877800	0.77323300	-0.00257500
C	1.72057600	-0.61662000	-0.00887500
C	2.74868700	-1.57805100	-0.00509000
C	4.05519600	-1.10884700	0.00541200
C	4.32183200	0.27593300	0.01040600
C	3.29868300	1.23241500	0.00614900
H	2.52425600	-2.63968800	-0.00885000
H	4.88360400	-1.80968400	0.01015000
C	0.30990200	-0.77370200	-0.01483200
H	-0.22508000	-1.71213500	-0.02304200
N	-0.30445800	0.40175600	-0.01015700
C	-1.72381600	0.73685000	-0.00328400
O	-1.96334000	1.92359700	-0.00275600
C	-2.79471600	-0.36128800	0.00415000
C	-4.16610000	0.34313300	0.02969300
H	-4.28483000	0.96406500	0.92191800
H	-4.31102000	0.97707400	-0.84933800
H	-4.94993900	-0.41994800	0.03573200
C	-2.67392400	-1.23405500	1.27902000
H	-3.52954200	-1.91567100	1.31006500
H	-1.77037000	-1.84557800	1.31674000
H	-2.70532300	-0.61380000	2.18096200
C	-2.70932500	-1.20911300	-1.29000700
H	-1.80010700	-1.80911800	-1.36799400
H	-3.55819200	-1.89971700	-1.30541900
H	-2.77616400	-0.57261200	-2.17851200
C	0.68813700	1.50840200	-0.00498600
H	0.53357400	2.13032300	0.88181600
H	0.53875300	2.13251800	-0.89131200
H	3.53001200	2.29257300	0.01060900
H	5.35483800	0.61058900	0.01852200

Synthesis and further characterization of compounds **1d**, **1e**, **2b**, **3b**, **8b**, and **23b**

***N*-[(2-Formyl-3,4,5-trimethoxyphenyl)methyl]-2,2-dimethylpropanamide (1d).** A solution of BuLi (1.6 M in hexane, 33.4 mL, 53.5 mmol) was added to a solution of 2,2-dimethyl-*N*-[(3,4,5-trimethoxyphenyl)methyl]propanamide (5.01 g, 17.83 mmol) in THF (60 mL) at 0 °C. After stirring for 1 h at 0 °C, DMF (4.1 mL, 5.9 g, 53 mmol) was added. The mixture was stirred for 1 h at room temperature. The reaction mixture was diluted with water (20 mL). The layers were separated and the aqueous layer was extracted with diethyl ether (20 and 2 × 10 mL). The combined organic layer was dried over $MgSO_4$. The solvents were evaporated and the residue purified by elution on silica gel with 10–50% EtOAc in hexane to afford the title compound (4.42 g, 80%) as pale yellow solid. Mp 100–102 °C (hexane). IR (KBr): νNH 3307; νCH 2965; νHC=O 1674; νC=O (amide) 1639; νC=C (Ar) 1595, 1564; ν_{as}C−O−C 1309; ν_sC−O−C 1123 cm^{-1}. HRMS calcd. for $C_{16}H_{24}NO_5^+$ ($[M+H]^+$): 310.1649, found: 310.1647. Anal. calcd. for $C_{16}H_{23}NO_5$ (309.36): N 4.53, H 7.49, C 62.12%. Found: N 4.46, H 7.40, C 62.35%. The assignment of ^{1}H and ^{13}C chemical shifts, ^{1}H, DeptQ, edited HSQC and HMBC spectra with the structure can be found on pages S3–S5).

***N*-[(2-Formylphenyl)methyl]-2,2-dimethylpropanamide (1e).** A solution of BuLi (1.6 M in hexane, 100 mL, 160 mmol) was added to a solution of **21** (14.4 g, 53 mmol) in THF (170 mL) at −40 °C. After stirring for 30 min at −40 °C, DMF (12.4 mL, 11.7 g, 160 mmol) was added. The mixture was stirred for 1 h at room temperature. The reaction mixture was diluted with water (60 mL). The layers were separated and the aqueous layer was extracted with diethyl ether (40 and 2 × 30 mL). The combined organic layer was dried over $MgSO_4$. The solvents were evaporated and the residue purified by flash chromatography (8–20% EtOAc in hexane) to afford the title compound (6.51 g, 56%) as white solid. Mp 82–84 °C (EtOAc/hexane). IR (KBr): νNH 3336; νCH 3074; νHC=O 1695; νC=O (amide) 1637; νC=C (Ar) 1602, 1574 cm^{-1}. HRMS calcd. for $C_{13}H_{18}NO_2^+$ ($[M+H]^+$): 220.1332, found: 220.1347. Anal. calcd. for $C_{13}H_{17}NO_2$ (219.28): N 6.39, H 7.81, C 71.21%. Found: N 6.48, H 7.50, C 71.30%. Pages S6 and S7 contain the ^{1}H, ^{13}C NMR spectra, and the aromatic section of edited HSQC and HMBC spectra.

***N*-[(2-Formyl-5-methoxyphenyl)methyl]-2,2-dimethylpropanamide (2b).** *Method A:* This compound was prepared according to general procedure I using **1b** [2] (1.01 g, 4.06 mmol) and TFA (31 µL, 46 mg, 0.41 mmol). The title compound (62 mg, 6%) was isolated as white solid. Mp 98–100 °C (EtOAc/hexane). IR (KBr): νNH 3327; νCH 3084; νHC=O 1684; νC=O (amide)

1640; νC=C (Ar) 1608, 1568; ν_{as}C−O−C 1286; ν_sC−O−C 1041 cm^{-1}. HRMS calcd. for $C_{14}H_{20}NO_3^+$ ([M+H]$^+$): 250.1438, found: 250.1440. Anal. calcd. for $C_{14}H_{19}NO_3$ (249.31): N 5.62, H 7.68, C 67.47%. Found: N 5.67, H 7.54, C 67.25%. Pages S8–S10 contain the ^{1}H, sel-NOE on 4.59 and 9.92 signals, ^{13}C, Dept-135, HSQC and HMBC spectra. *Method B:* This compound was prepared according to general procedure II using **1b** [2] (1.01 g, 4.06 mmol) and TFA (31 µL, 46 mg, 0.41 mmol). The title compound (349 mg, 35%) was isolated as white solid. Analytical data were identical with those described in Method A.

***N*-[(*SR*)-[(1*RS*)-2-(2,2-Dimethylpropanoyl)-5-methoxy-2,3-dihydro-1*H*-isoindol-1-yl](2-formyl-5-methoxyphenyl)methyl]-2,2-dimethylpropanamide (3b).** *Method A:* This compound was prepared according to general procedure I using **1b** [2] (1.01 g, 4.06 mmol) and TFA (31 µL, 46 mg, 0.41 mmol). The title compound (432 mg, 43%) was isolated as white solid. Mp 184–186 °C (EtOAc/hexane). IR (KBr): νNH 3325; νCH 2963; νHC=O 1681; νC=O (amide) 1653; νC=C (Ar) 1597, 1499; ν_{as}C−O−C 1232; ν_sC−O−C 1030 cm^{-1}. HRMS calcd. for $C_{28}H_{37}N_2O_5^+$ ([M+H]$^+$): 481.2697, found: 481.2695. Anal. calcd. for $C_{28}H_{36}N_2O_5$ (480.61): N 5.83, H 7.55, C 69.98%. Found: N 5.76, H 7.20, C 69.77%. To elucidate the dimeric type structure of **3b** and to achieve its complete NMR signal assignment, we have run, in addition to the ^{1}H NMR measurements, a series of one-dimensional sel-NOE and ^{1}H,^{1}H-COSY experiments (see pages S11–S14). The H–C–C–H dihedral angle between the two asymmetric centers are not far from 90°, which is reflected by the detected low ^3J(H,H) coupling constants (ca. 1 Hz). In the case of the ^{13}C assignment, Dept-135, HSQC and HMBC spectra were taken with the required expansions. Even the very close ^{13}C signals (δ 27.39 and δ 27.47) of the two $\underline{Me_3}$C groups were unambiguously distinguished and clearly assigned. To achieve the required extreme high ^{13}C chemical shift resolution, the band-selective HSQC experiment proved to be the method of choice (pages S15–S18). *Method B:* This compound was prepared according to general procedure II using **1b** [2] (1.01 g, 4.06 mmol) and TFA (31 µL, 46 mg, 0.41 mmol). The title compound (188 mg, 19%) was isolated as white solid. Analytical data were identical with those described in Method A.

***N*-[(*SR*)-[(1*RS*)-2-(2,2-Dimethylpropanoyl)-5,6,7-trimethoxy-2,3-dihydro-1*H*-isoindol-1-yl](2-formyl-3,4,5-trimethoxyphenyl)methyl]-2,2-dimethylpropanamide (8b).** *Method A:* This compound was prepared according to general procedure I using **1d** (1.00 g, 3.24 mmol) and TFA (25 µL, 37 mg, 0.32 mmol). The title compound (104 mg, 10%) was isolated as white solid. Mp 180−182 °C (EtOAc/hexane). IR (KBr): νNH 3292; νCH 2962; νHC=O 1692; νC=O (amide) 1659; νC=C (Ar) 1614, 1588; ν_{as}C−O−C 1310; ν_sC−O−C 1120 cm^{-1}. HRMS calcd. for

$C_{32}H_{45}N_2O_9^+$ ([M+H]$^+$): 601.3112, found: 601.3100. Anal. calcd. for $C_{32}H_{44}N_2O_9$ (600.71): N 4.66, H 7.38, C 63.98%. Found: N 4.64, H 7.14, C 63.83%. ^{1}H and ^{1}H,^{1}H-COSY spectra are shown on page S25. One-dimensional sel-NOE experiments on signals 9.79, 9.06, 6.67, 5.81 and 3.42 together with two-dimensional NOESY elucidated the characteristic steric proximities (pages S26–S28) and proved the *SR*-*RS* type structure. The ^{13}C signal assignment (page S29) was supported by HSQC and HMBC measurements (pages S30, S31). *Method B:* This compound was prepared according to general procedure II using **1d** (1.00 g, 3.24 mmol) and TFA (25 µL, 37 mg, 0.32 mmol). The title compound (143 mg, 14%) was isolated as white solid. Analytical data were identical with those described in Method A.

***N*-[(*RS*)-[(1*RS*)-2-(2,2-dimethylpropanoyl)-2,3-dihydro-1*H*-isoindol-1-yl](2-formylphenyl)methyl]-2,2-dimethylpropanamide (23b).** TFA (210 µL, 312 mg, 2.74 mmol) was added to a solution of **1e** (6.00 g, 27.40 mmol) in DCM (120 mL). After stirring for 24 h at room temperature, an aqueous sodium carbonate solution (5%, 35 mL) was added. The aqueous layer was extracted with DCM (2 × 8 mL). The combined organic layer was dried over $MgSO_4$. The solvent was evaporated and the residue was purified by flash chromatography (5–15% EtOAc in hexane). The corresponding fractions were collected, evaporated, and recrystallized from EtOAc/hexane. The mother liquor of the recrystallization was evaporated and purified by flash chromatography (5–15% EtOAc in hexane). The corresponding fractions were collected and evaporated to afford 1.15 g diastereomeric mixture containing 37% of the *RS*-*SR* and 45% of the *RR*-*SS* type product (HPLC, 254 nm). 250 mg of this mixture were injected in 9 mL DMSO onto a Gemini-NX C18 AXIA preparative HPLC column (250 × 50 mm, guard column 15 × 30 mm, particle size 10 µm) equilibrated in acetonitrile/water 1:1. The same composition was used for isocratic elution at a flow rate of 50 mL/min at 25 °C. The UV detection wavelength was set to 254 nm. A pure fraction of the more retained isomer was heart-cut between 61.5–72 min. Most of the acetonitrile was removed under reduced pressure (100 mbar) on a rotary evaporator at 30 °C. The residue was extracted with DCM (3 × 30 mL), the combined organic phases were dried over $MgSO_4$, and evaporated to dryness under vacuum on a 30 °C bath to afford 86 mg of **23b** as white solid foam. HPLC purity: 97% (254 nm). IR (KBr): νNH 3347; νCH 2967; νHC=O 1694; νC=O (amide) 1660; νC=C (Ar) 1610, 1511 cm^{-1}. HRMS calcd. for $C_{26}H_{33}N_2O_3^+$ ([M+H]$^+$): 421.2491, found: 421.2482. The ^{1}H and ^{13}C spectra are shown on page S38. The identification of the three different ^{1}H spin systems was achieved by selective one-dimensional TOCSY measurement on signals 7.76, 7.24 and 8.50 (page S39). These experiments unambiguously proved that the

signal appearing at δ 5.71 ppm corresponded to two hydrogen atoms. The broad signal (indicating a hindered rotation) can be assigned to the HN–CH–CH three-spin system, and the ^{3}J(H,H) coupling constant is now 10 Hz, i.e., these hydrogen atoms are antiperiplanar. The second δ 5.71 signal is a doublet (7.5 Hz) and is surprisingly part of the aromatic four-spin system of the isoindoline moiety (H-7). The characteristic steric proximities were detected utilizing the two-dimensional NOESY and one-dimensional sel-NOE experiments (pages S40, S41). The ^{13}C signal assignment was supported by HSQC and HMBC measurements (pages S42, S43).
