# Supplementary material for: Rearrangement of o-(pivaloylaminomethyl)benzaldehydes: an experimental and computational study
Source: Beilstein J Org Chem. 2020 Jul 13;16:1636–48. doi: 10.3762/bjoc.16.136 (PMC7372232; doi:10.3762/bjoc.16.136)
Supplement: File 2 — Crystallographic information files for compounds 3a, 3b, 8b, 23a, and 23b. [file Beilstein_J_Org_Chem-16-1636-s002.zip › compound+3b+X-ray+structure+report.pdf]

127034

HCS0449_1B

Submitted by: Hargitai Csilla
Operator: Dancso Andras

X-ray Structure Report

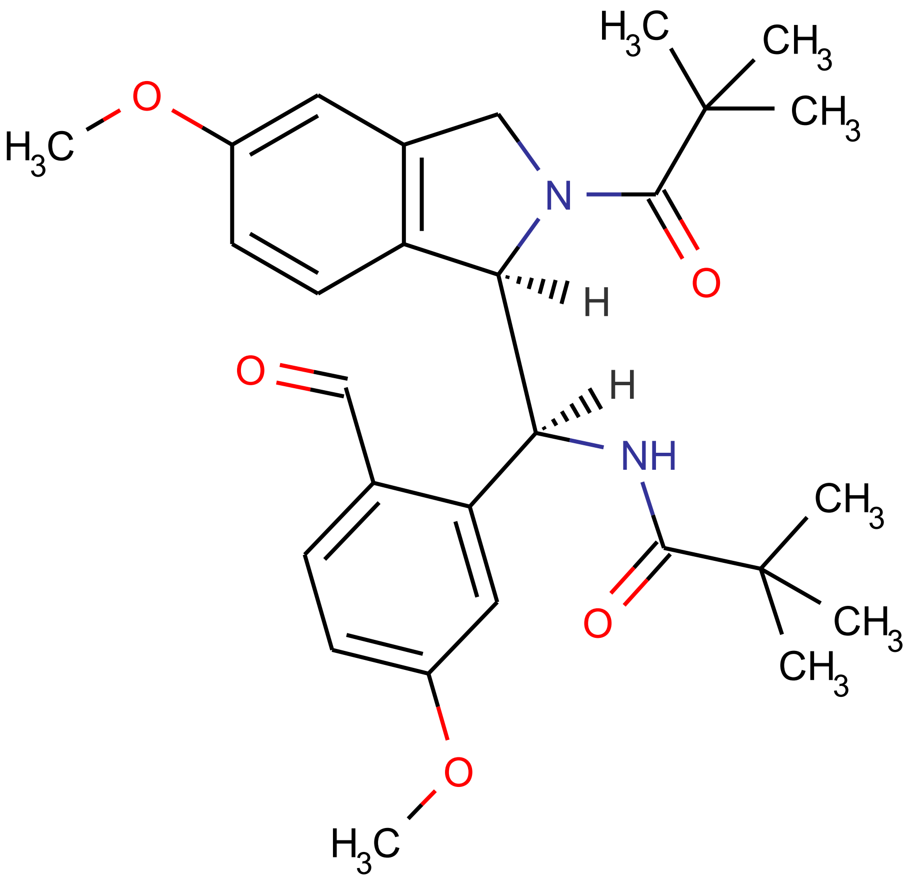

October 9, 2018

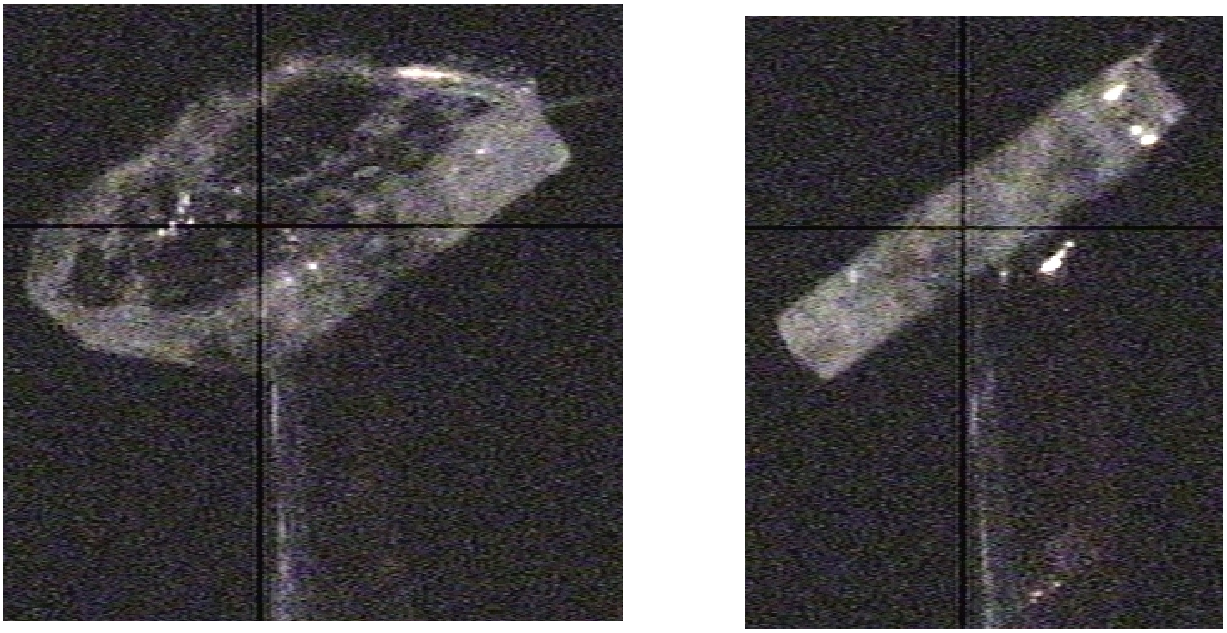

Fig. 1. The crystal

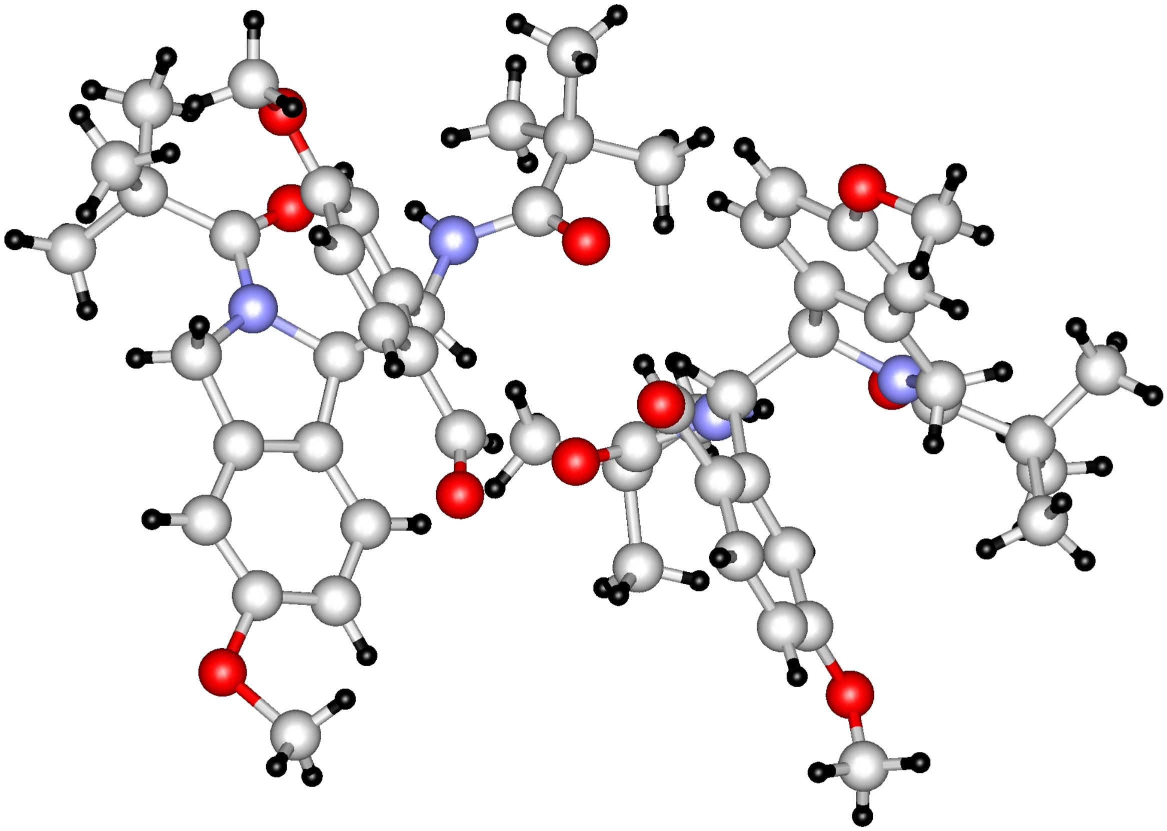

Fig. 2. Molecules in pair (hydrogens were generated by the software)

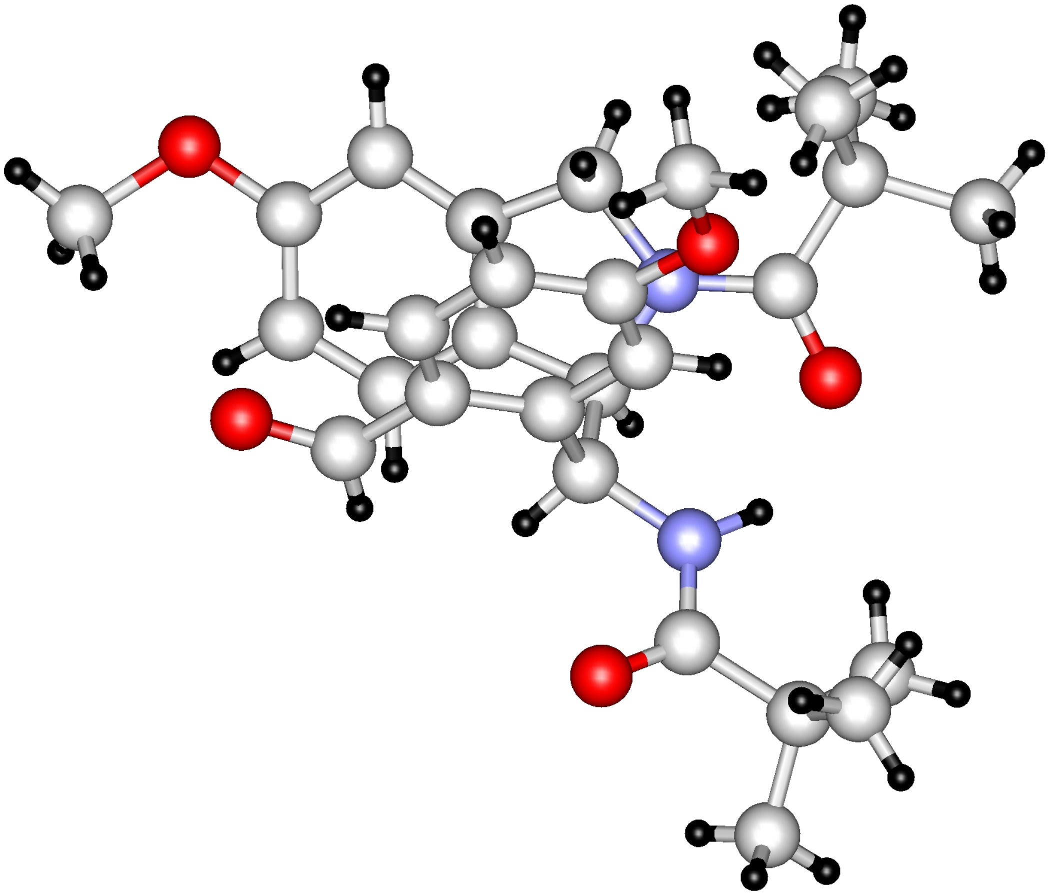

Fig. 3. Fragment 1

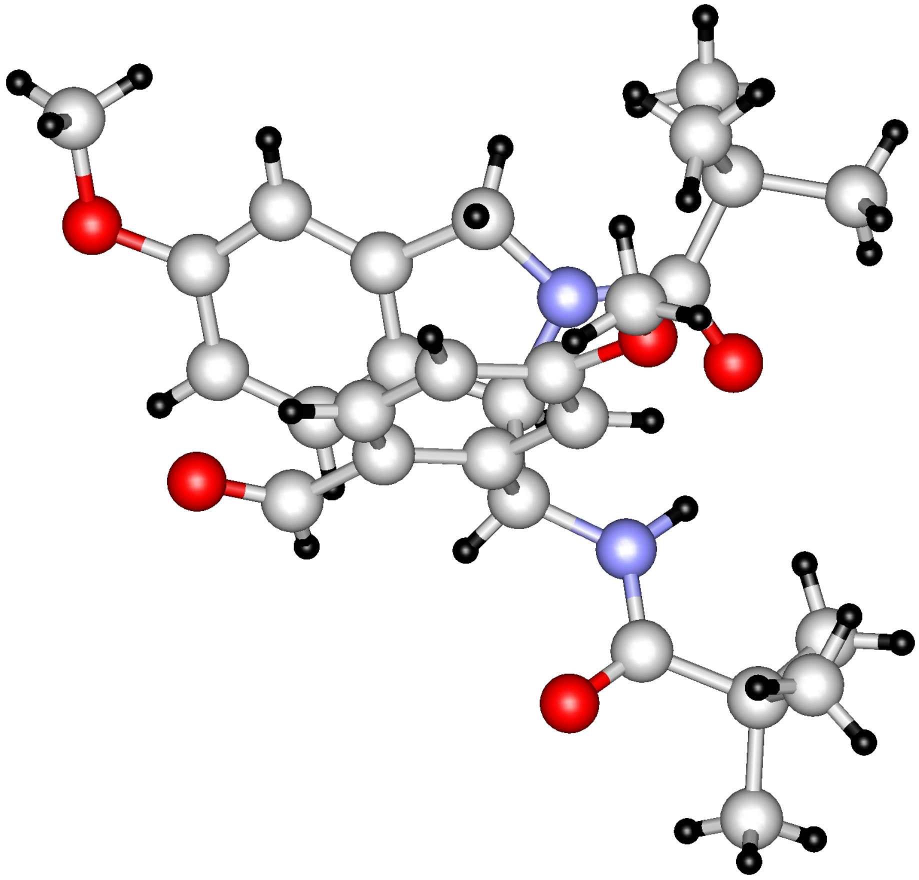

Fig. 4. Fragment 2

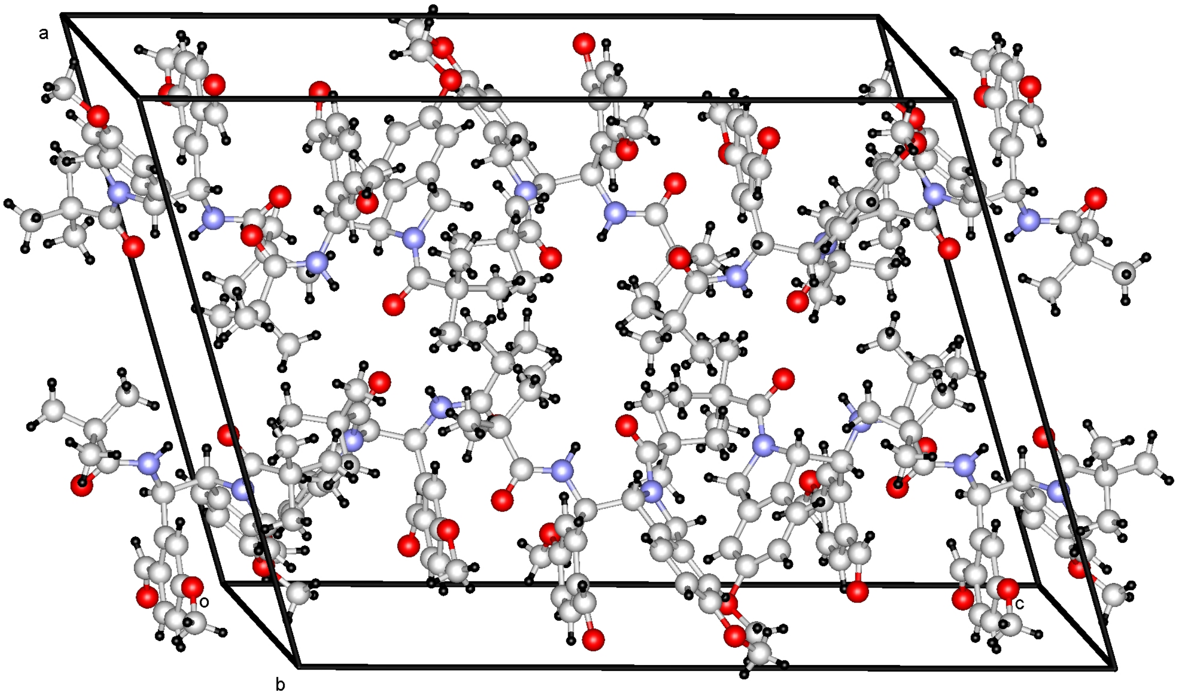

Fig. 5. Packing

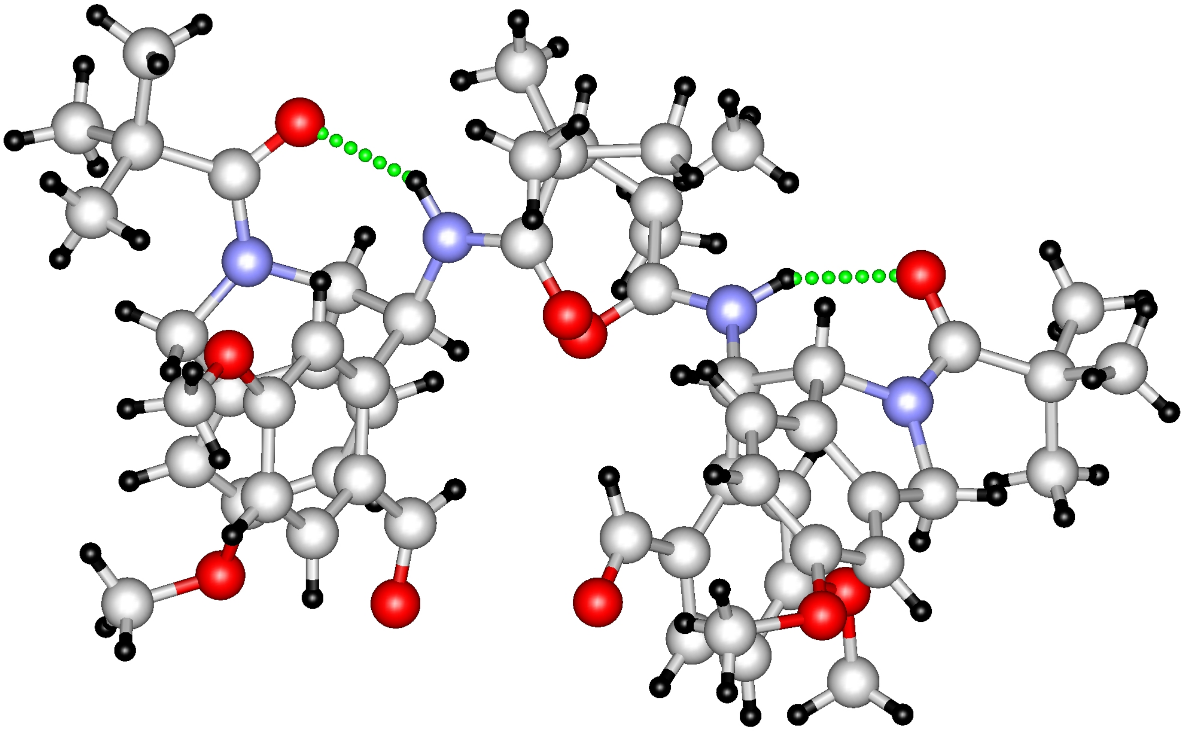

Fig. 6. Hydrogen bonds

Experimental

Data Collection

A colorless block crystal of $C_{28}H_{36}N_2O_5$ having approximate dimensions of 0.79 x 0.71 x 0.15 mm was mounted on a cactus needle. All measurements were made on a Rigaku RAXIS RAPID imaging plate area detector with graphite monochromated Cu-Kα radiation.

Indexing was performed from 4 oscillations that were exposed for 60 seconds. The crystal-to-detector distance was 127.40 mm.

Cell constants and an orientation matrix for data collection corresponded to a primitive monoclinic cell with dimensions:

a = 19.8504(5) Å

b = 10.5243(2) Å β = 109.1019(13)^{o}

c = 26.8357(6) Å

V = 5297.6(2) Å^{3}

For Z = 8 and F.W. = 480.60, the calculated density is 1.205 g/cm^{3}. The systematic absences of:

h0l: l ± 2n
0k0: k ± 2n

uniquely determine the space group to be:

$P2_1/c$ (#14)

The data were collected at a temperature of -99 ± 1°C to a maximum 2θ value of 143.6^{o}. A total of 180 oscillation images were collected. A sweep of data was done using ω scans from 20.0 to 200.0^{o} in 5.0^{o} step, at χ=0.0^{o} and ϕ = 0.0^{o}. The exposure rate was 12.0 [sec./^{o}]. A second sweep was performed using ω scans from 20.0 to 200.0^{o} in 5.0^{o} step, at χ=54.0^{o} and ϕ = 0.0^{o}. The exposure rate was 12.0 [sec./^{o}]. Another sweep was performed using ω scans from 20.0 to 200.0^{o} in 5.0^{o} step, at χ=54.0^{o} and ϕ = 90.0^{o}. The exposure rate was 12.0 [sec./^{o}]. Another sweep was performed using ω scans from 20.0 to 200.0^{o} in 5.0^{o} step, at χ=54.0^{o} and ϕ = 180.0^{o}. The exposure rate was 12.0 [sec./^{o}]. Another sweep was performed using ω scans from 20.0 to 200.0^{o} in 5.0^{o} step, at χ=54.0^{o} and ϕ = 270.0^{o}. The exposure rate was 12.0 [sec./^{o}]. The crystal-to-detector distance was 127.40 mm. Readout was performed in the 0.100 mm pixel mode.

Data Reduction

Of the 59019 reflections that were collected, 10181 were unique (R_{int} = 0.065).

The linear absorption coefficient, μ, for Cu-Kα radiation is 6.668 cm^{-1}. An empirical absorption correction was applied which resulted in transmission factors ranging from 0.702 to 0.905. The data were corrected for Lorentz and polarization effects.

Structure Solution and Refinement

The structure was solved by direct methods[1] and expanded using Fourier techniques[2]. Some non-hydrogen atoms were refined anisotropically, while the rest were refined isotropically. Hydrogen atoms were refined using the riding model. The final cycle of full-matrix least-squares refinement[3] on F was based on 29487 observed reflections ($I > 2.00\sigma(I)$) and 638 variable parameters and converged (largest parameter shift was 0.00 times its esd) with unweighted and weighted agreement factors of:

$$R = \Sigma \, ||Fo| - |Fc|| \, / \, \Sigma \, |Fo| = 0.1023$$

$$R_w = [\, \Sigma \, w \, (|Fo| - |Fc|)^2 \, / \, \Sigma \, w \, Fo^2]^{1/2} = 0.1012$$

The standard deviation of an observation of unit weight[4] was 6.84. Unit weights were used. Plots of $\Sigma \, w \, (|Fo| - |Fc|)^2$ versus |Fo|, reflection order in data collection, sin θ/λ and various classes of indices showed no unusual trends. The maximum and minimum peaks on the final difference Fourier map corresponded to 14.20 and -52.50 $e^-/Å^3$, respectively.

Neutral atom scattering factors were taken from Cromer and Waber[5]. Anomalous dispersion effects were included in Fcalc[6]; the values for Δf' and Δf" were those of Creagh and McAuley[7]. The values for the mass attenuation coefficients are those of Creagh and Hubbell[8]. All calculations were performed using the CrystalStructure[9,10] crystallographic software package.

References

(1) SIR92: Altomare, A., Cascarano, G., Giacovazzo, C., Guagliardi, A., Burla, M., Polidori, G., and Camalli, M. (1994) J. Appl. Cryst., 27, 435.

(2) DIRDIF99: Beurskens, P.T., Admiraal, G., Beurskens, G., Bosman, W.P., de Gelder, R., Israel, R. and Smits, J.M.M.(1999). The DIRDIF-99 program system, Technical Report of the Crystallography Laboratory, University of Nijmegen, The Netherlands.

(3) Least Squares function minimized:

$$\Sigma w(|F_o|-|F_c|)^2 \quad \text{where w = Least Squares weights.}$$

(4) Standard deviation of an observation of unit weight:

$$[\Sigma w(|F_o|-|F_c|)^2/(N_o-N_v)]^{1/2}$$

where: N_o = number of observations
N_v = number of variables

(5) Cromer, D. T. & Waber, J. T.; "International Tables for X-ray Crystallography", Vol. IV, The Kynoch Press, Birmingham, England, Table 2.2 A (1974).

(6) Ibers, J. A. & Hamilton, W. C.; Acta Crystallogr., 17, 781 (1964).

(7) Creagh, D. C. & McAuley, W.J .; "International Tables for Crystallography", Vol C, (A.J.C. Wilson, ed.), Kluwer Academic Publishers, Boston, Table 4.2.6.8, pages 219-222 (1992).

(8) Creagh, D. C. & Hubbell, J.H..; "International Tables for Crystallography", Vol C, (A.J.C. Wilson, ed.), Kluwer Academic Publishers, Boston, Table 4.2.4.3, pages 200-206 (1992).

(9) CrystalStructure 3.7.0: Crystal Structure Analysis Package, Rigaku and Rigaku/MSC (2000-2005). 9009 New Trails Dr. The Woodlands TX 77381 USA.

(10) CRYSTALS Issue 10: Watkin, D.J., Prout, C.K. Carruthers, J.R. & Betteridge, P.W. Chemical Crystallography Laboratory, Oxford, UK. (1996)

EXPERIMENTAL DETAILS

A. Crystal Data

Empirical Formula	$C_{28}H_{36}N_2O_5$
Formula Weight	480.60
Crystal Color, Habit	colorless, block
Crystal Dimensions	0.79 X 0.71 X 0.15 mm
Crystal System	monoclinic
Lattice Type	Primitive
Indexing Images	4 oscillations @ 60.0 seconds
Detector Position	127.40 mm
Pixel Size	0.100 mm
Lattice Parameters	a = 19.8504(5) Å<br>b = 10.5243(2) Å<br>c = 26.8357(6) Å<br>β = 109.1019(13) ^{o}<br>V = 5297.6(2) Å^3
Space Group	$P2_1/c$ (#14)
Z value	8
D_{calc}	1.205 g/cm^3
F_{000}	2064.00
μ(CuKα)	6.668 cm^{-1}

B. Intensity Measurements

Diffractometer	Rigaku RAXIS-RAPID
Radiation	CuKα (λ = 1.54187 Å)<br>graphite monochromated
Detector Aperture	280 mm x 256 mm
Data Images	180 exposures
ω oscillation Range (χ=0.0, ϕ=0.0)	20.0 - 200.0^{o}
Exposure Rate	12.0 sec./^{o}
ω oscillation Range (χ=54.0, ϕ=0.0)	20.0 - 200.0^{o}
Exposure Rate	12.0 sec./^{o}
ω oscillation Range (χ=54.0, ϕ=90.0)	20.0 - 200.0^{o}
Exposure Rate	12.0 sec./^{o}
ω oscillation Range (χ=54.0, ϕ=180.0)	20.0 - 200.0^{o}
Exposure Rate	12.0 sec./^{o}
ω oscillation Range (χ=54.0, ϕ=270.0)	20.0 - 200.0^{o}
Exposure Rate	12.0 sec./^{o}
Detector Position	127.40 mm
Pixel Size	0.100 mm
$2\theta_{max}$	143.6^{o}
No. of Reflections Measured	Total: 59019<br>Unique: 10181 (R_{int} = 0.065)
Corrections	Lorentz-polarization<br>Absorption<br>(trans. factors: 0.702 - 0.905)

C. Structure Solution and Refinement

Structure Solution	Direct Methods (SIR92)
Refinement	Full-matrix least-squares on F
Function Minimized	Σ w (|Fo| - |Fc|)^2
Least Squares Weights	1
$2\theta_{max}$ cutoff	143.6^o
Anomalous Dispersion	All non-hydrogen atoms
No. Observations ($I>2.00\sigma(I)$)	29487
No. Variables	638
Reflection/Parameter Ratio	46.22
Residuals: R ($I>2.00\sigma(I)$)	0.1023
Residuals: Rw ($I>2.00\sigma(I)$)	0.1012
Goodness of Fit Indicator	6.839
Max Shift/Error in Final Cycle	0.000
Maximum peak in Final Diff. Map	14.20 $e^-/Å^3$
Minimum peak in Final Diff. Map	-52.50 $e^-/Å^3$

Table 1. Atomic coordinates and B_{iso}/B_{eq}

atom	x	y	z	B_{eq}
O(1)	0.70334(14)	0.6189(2)	0.11585(10)	3.86(8)
O(2)	0.61986(13)	0.1883(2)	-0.00541(10)	3.64(8)
O(3)	0.60491(13)	0.7755(2)	0.25599(11)	3.94(8)
O(4)	0.73026(13)	0.3339(2)	0.18242(10)	3.64(7)
O(5)	0.84933(18)	-0.0771(3)	0.10852(12)	6.69(8)
O(6)	0.93575(16)	0.4812(2)	0.25805(11)	5.42(7)
O(8)	0.82478(13)	1.0503(2)	0.24106(10)	4.44(6)
O(9)	0.95107(17)	0.5003(2)	0.12986(11)	5.76(7)
O(10)	0.91955(16)	0.6664(2)	-0.03438(11)	5.26(7)
O(11)	0.9424(2)	0.3752(3)	0.42956(13)	8.42(9)
N(1)	0.71452(18)	0.7249(3)	0.30675(12)	2.89(9)
N(2)	0.72559(19)	0.2564(3)	-0.00874(12)	2.85(7)
N(3)	0.66379(16)	0.6726(2)	0.18283(12)	2.77(8)
N(13)	0.68488(16)	0.2879(2)	0.09600(12)	2.96(9)
C(1)	0.7803(2)	0.5874(4)	0.02074(14)	3.46(13)
C(2)	0.6823(2)	0.2896(3)	0.14524(17)	2.62(11)
C(16)	0.7703(2)	0.5287(4)	0.31298(16)	2.51(11)
C(17)	0.7221(2)	0.3713(3)	0.02388(14)	3.16(11)
C(18)	0.7831(2)	0.8428(4)	0.23063(13)	3.21(12)
C(19)	0.8047(2)	0.1302(4)	0.09981(13)	3.48(12)
C(20)	0.7931(2)	0.7109(4)	0.23006(12)	2.51(11)
C(21)	0.7871(2)	0.4033(4)	0.30990(16)	3.41(12)
C(22)	0.8811(2)	0.3163(4)	0.11799(13)	2.82(11)
C(23)	0.7280(2)	0.6269(3)	0.22310(13)	2.52(10)
C(24)	0.8626(2)	0.6638(4)	0.24074(14)	2.87(11)
C(25)	0.6167(2)	0.2270(4)	0.15337(17)	3.16(12)
C(26)	0.7450(2)	0.3450(3)	0.08424(13)	2.64(11)
C(27)	0.7136(2)	0.6074(3)	0.27562(14)	2.77(11)
C(29)	0.8654(2)	0.0531(4)	0.11206(16)	3.63(13)
C(30)	0.8604(2)	0.4637(4)	-0.03314(16)	3.93(13)
C(31)	0.8115(2)	0.2633(4)	0.10224(13)	2.74(11)
C(32)	0.8750(2)	0.5273(4)	0.23933(14)	4.22(10)
C(33)	0.8297(2)	0.6521(4)	0.00562(17)	4.16(13)
C(34)	0.8115(2)	0.6019(4)	0.35469(16)	2.99(12)
C(35)	0.7711(2)	0.4629(4)	0.00984(16)	2.87(12)
C(36)	0.6692(2)	0.1729(4)	-0.02283(16)	3.53(13)
C(37)	0.6557(2)	0.8014(4)	0.29415(18)	3.11(12)

Table 1. Atomic coordinates and B_{iso}/B_{eq} (continued)

atom	x	y	z	B_{eq}
C(38)	0.9090(2)	0.8768(4)	0.25217(14)	3.60(12)
C(39)	0.9399(2)	0.2366(4)	0.13097(14)	3.57(12)
C(40)	0.8412(2)	0.9247(4)	0.24113(14)	3.13(12)
C(41)	0.6487(2)	0.8731(3)	0.38243(14)	5.23(14)
C(42)	0.5811(2)	0.6969(4)	0.09282(16)	3.55(12)
C(43)	0.9328(2)	0.1083(4)	0.12758(14)	3.63(13)
C(44)	0.8092(2)	0.4020(4)	-0.01657(16)	2.95(12)
C(45)	0.6549(2)	0.9159(4)	0.32979(18)	3.48(12)
C(46)	0.6554(2)	0.6592(3)	0.13119(18)	3.13(12)
C(47)	0.8711(2)	0.5890(5)	-0.02082(18)	4.12(14)
C(48)	0.5869(2)	0.9925(3)	0.30087(16)	5.27(14)
C(49)	0.9182(2)	0.7484(4)	0.25089(16)	4.00(13)
C(50)	0.8925(2)	0.4530(4)	0.12466(14)	4.42(10)
C(51)	0.6693(2)	0.0624(4)	-0.06016(19)	3.78(13)
C(52)	0.7841(2)	0.7356(3)	0.35102(14)	3.59(12)
C(53)	0.5938(2)	0.3037(3)	0.19313(17)	5.51(15)
C(54)	0.6545(2)	0.1101(4)	-0.11640(17)	6.22(16)
C(55)	0.8827(2)	1.1426(3)	0.25029(14)	5.17(11)
C(56)	0.7894(2)	0.2645(3)	-0.02496(13)	3.32(11)
C(57)	0.8439(2)	0.3489(4)	0.34833(17)	3.97(13)
C(58)	0.7180(2)	1.0054(3)	0.33813(14)	4.82(13)
C(59)	0.7385(2)	-0.0142(3)	-0.04235(17)	5.81(15)
C(60)	0.8696(2)	0.5524(4)	0.39397(16)	3.97(13)
C(61)	0.5256(2)	0.7147(6)	0.1172(2)	13.6(2)
C(62)	0.5534(2)	0.2139(4)	0.10259(16)	6.39(16)
C(63)	0.6409(2)	0.0953(4)	0.17628(17)	5.92(16)
C(64)	0.5881(2)	0.8197(5)	0.0674(2)	12.1(2)
C(65)	0.9589(2)	0.6041(4)	-0.06296(16)	6.30(12)
C(66)	0.8836(2)	0.4261(5)	0.38856(19)	5.49(16)
C(67)	0.6082(2)	-0.0242(4)	-0.05984(18)	6.97(17)
C(69)	0.9783(5)	0.2673(9)	0.4103(3)	28.0(4)
C(70)	0.5585(2)	0.5989(5)	0.0511(2)	14.9(2)
C(71)	0.9002(5)	-0.1535(8)	0.1228(3)	24.4(3)
H(1)	0.7523	0.6288	0.0386	4.16
H(2)	0.7365	0.8765	0.2234	3.89
H(3)	0.7587	0.0926	0.0899	3.98
H(4)	0.7595	0.3544	0.2806	3.99

Table 1. Atomic coordinates and B_{iso}/B_{eq} (continued)

atom	x	y	z	B_{eq}
H(5)	0.8872	0.4209	-0.0516	4.63
H(6)	0.8371	0.7403	0.0128	4.62
H(7)	0.9487	0.9327	0.2601	4.11
H(8)	0.9860	0.2737	0.1427	4.27
H(9)	0.9739	0.0558	0.1349	4.37
H(10)	0.9648	0.7145	0.2581	4.59
H(11)	0.8555	0.2614	0.3477	4.71
H(12)	0.8979	0.6024	0.4226	4.56
H(13)	0.6748	0.4034	0.0118	3.72
H(14)	0.7380	0.5466	0.2110	2.93
H(15)	0.7551	0.4240	0.1023	3.26
H(16)	0.6689	0.5662	0.2691	3.14
H(17)	0.8151	0.7941	0.3425	4.12
H(18)	0.7765	0.7617	0.3827	4.12
H(19)	0.8263	0.2107	-0.0041	4.01
H(20)	0.7776	0.2414	-0.0610	4.01
H(21)	0.6282	0.7142	0.1934	3.16
H(22)	0.6483	0.2480	0.0683	3.57
H(23)	0.5996	0.8665	0.3787	6.50
H(24)	0.6710	0.9325	0.4095	6.51
H(25)	0.6708	0.7924	0.3914	6.49
H(26)	0.5979	1.0540	0.2788	6.71
H(27)	0.5694	1.0341	0.3256	6.72
H(28)	0.5517	0.9359	0.2800	6.72
H(29)	0.6183	0.2747	0.2279	7.06
H(30)	0.5439	0.2948	0.1859	7.06
H(31)	0.6049	0.3905	0.1901	7.05
H(32)	0.6044	0.1095	-0.1339	7.52
H(33)	0.6771	0.0587	-0.1354	7.52
H(34)	0.6717	0.1947	-0.1147	7.51
H(35)	0.9023	1.1591	0.2870	6.22
H(36)	0.8649	1.2195	0.2322	6.20
H(37)	0.9186	1.1090	0.2378	6.23
H(38)	0.7565	0.9803	0.3682	5.64
H(39)	0.7045	1.0901	0.3427	5.65
H(40)	0.7323	1.0011	0.3077	5.65
H(41)	0.7730	0.0211	-0.0560	7.29

Table 1. Atomic coordinates and B_{iso}/B_{eq} (continued)

atom	x	y	z	B_{eq}
H(42)	0.7299	-0.1002	-0.0533	7.29
H(43)	0.7558	-0.0105	-0.0049	7.29
H(44)	0.5010	0.6377	0.1180	16.04
H(45)	0.4929	0.7761	0.0969	16.03
H(46)	0.5469	0.7453	0.1521	16.04
H(47)	0.5239	0.2871	0.0970	7.63
H(48)	0.5266	0.1412	0.1055	7.61
H(49)	0.5706	0.2032	0.0737	7.62
H(50)	0.6327	0.0358	0.1483	7.59
H(51)	0.6151	0.0704	0.1989	7.58
H(52)	0.6904	0.0979	0.1958	7.60
H(53)	0.5810	0.8880	0.0882	12.61
H(54)	0.5541	0.8248	0.0330	12.61
H(55)	0.6348	0.8246	0.0651	12.61
H(56)	1.0014	0.5704	-0.0386	7.61
H(57)	0.9709	0.6636	-0.0853	7.62
H(58)	0.9316	0.5371	-0.0837	7.56
H(59)	0.6249	-0.0860	-0.0328	8.86
H(60)	0.5879	-0.0657	-0.0928	8.86
H(61)	0.5731	0.0273	-0.0528	8.86
H(62)	0.9572	0.1873	0.4124	33.60
H(63)	1.0276	0.2647	0.4303	33.59
H(64)	0.9729	0.2856	0.3745	33.60
H(65)	0.5745	0.6168	0.0222	14.12
H(66)	0.5080	0.5926	0.0390	14.12
H(67)	0.5786	0.5209	0.0669	14.13
H(68)	0.9147	-0.1723	0.0932	29.34
H(69)	0.8882	-0.2300	0.1366	29.34
H(70)	0.9382	-0.1126	0.1491	29.33
H(71)	0.8358	0.4723	0.2238	5.08
H(72)	0.8540	0.5065	0.1247	5.31

$B_{eq} = 8/3\ \pi^2(U_{11}(aa^*)^2 + U_{22}(bb^*)^2 + U_{33}(cc^*)^2 + 2U_{12}(aa^*bb^*)\cos\gamma + 2U_{13}(aa^*cc^*)\cos\beta + 2U_{23}(bb^*cc^*)\cos\alpha)$

Table 2. Anisotropic displacement parameters

atom	U_{11}	U_{22}	U_{33}	U_{12}	U_{13}	U_{23}
O(1)	0.053(2)	0.058(2)	0.0364(18)	0.0089(18)	0.0142(16)	0.0028(15)
O(2)	0.037(2)	0.054(2)	0.052(2)	-0.0038(17)	0.0218(16)	-0.0159(16)
O(3)	0.034(2)	0.064(2)	0.0464(19)	0.0146(17)	0.0057(15)	-0.0064(17)
O(4)	0.046(2)	0.049(2)	0.0397(18)	-0.0112(16)	0.0080(15)	-0.0069(15)
N(1)	0.036(2)	0.042(2)	0.030(2)	-0.005(2)	0.0085(18)	0.0022(19)
N(3)	0.021(2)	0.046(2)	0.033(2)	0.0068(17)	0.0019(16)	-0.0012(18)
N(13)	0.028(2)	0.051(2)	0.035(2)	-0.0182(18)	0.0107(18)	-0.0093(18)
C(1)	0.044(3)	0.047(3)	0.042(2)	-0.005(2)	0.015(2)	0.006(2)
C(2)	0.040(3)	0.025(2)	0.035(2)	0.000(2)	0.013(2)	0.005(2)
C(16)	0.028(2)	0.037(3)	0.029(2)	0.004(2)	0.008(2)	0.004(2)
C(17)	0.042(3)	0.035(2)	0.041(2)	0.007(2)	0.010(2)	0.002(2)
C(18)	0.033(3)	0.050(3)	0.041(2)	-0.007(2)	0.014(2)	0.005(2)
C(19)	0.041(3)	0.052(3)	0.033(2)	-0.014(2)	0.004(2)	0.002(2)
C(20)	0.029(3)	0.045(3)	0.023(2)	-0.003(2)	0.011(2)	0.006(2)
C(21)	0.034(3)	0.056(3)	0.036(2)	0.004(2)	0.007(2)	0.004(2)
C(22)	0.035(3)	0.039(3)	0.035(2)	-0.008(2)	0.014(2)	-0.002(2)
C(23)	0.028(2)	0.032(2)	0.033(2)	0.002(2)	0.007(2)	-0.001(2)
C(24)	0.024(2)	0.038(3)	0.045(2)	0.009(2)	0.007(2)	0.004(2)
C(25)	0.036(3)	0.045(3)	0.040(2)	-0.015(2)	0.014(2)	0.001(2)
C(26)	0.036(3)	0.033(2)	0.035(2)	0.001(2)	0.016(2)	-0.002(2)
C(27)	0.027(2)	0.043(3)	0.030(2)	-0.011(2)	0.003(2)	-0.000(2)
C(29)	0.042(3)	0.050(3)	0.044(3)	-0.018(3)	0.012(2)	-0.000(2)
C(30)	0.037(3)	0.064(3)	0.046(3)	-0.001(3)	0.010(2)	0.011(2)
C(31)	0.038(3)	0.039(3)	0.029(2)	-0.001(2)	0.012(2)	-0.000(2)
C(33)	0.059(3)	0.040(3)	0.048(3)	-0.002(2)	0.002(2)	0.004(2)
C(34)	0.029(3)	0.054(3)	0.035(2)	0.013(2)	0.017(2)	0.012(2)
C(35)	0.035(3)	0.035(3)	0.042(2)	-0.005(2)	0.016(2)	-0.004(2)
C(36)	0.044(3)	0.044(3)	0.039(3)	-0.004(2)	0.004(2)	-0.001(2)
C(37)	0.037(3)	0.043(3)	0.042(3)	0.007(2)	0.018(2)	0.009(2)
C(38)	0.033(3)	0.048(3)	0.050(2)	-0.009(2)	0.005(2)	-0.006(2)
C(39)	0.047(3)	0.046(3)	0.042(2)	0.001(2)	0.014(2)	0.010(2)
C(40)	0.043(3)	0.035(3)	0.049(2)	0.000(2)	0.025(2)	0.001(2)
C(41)	0.087(4)	0.069(3)	0.049(3)	0.027(3)	0.031(2)	0.002(2)
C(42)	0.037(3)	0.053(3)	0.032(2)	0.008(2)	-0.006(2)	0.007(2)
C(43)	0.036(3)	0.057(3)	0.047(2)	0.009(2)	0.016(2)	0.009(2)
C(44)	0.025(3)	0.045(3)	0.038(2)	0.003(2)	0.004(2)	0.008(2)
C(45)	0.034(3)	0.046(3)	0.049(3)	0.013(2)	0.009(2)	0.002(2)

Table 2. Anisotropic displacement parameters (continued)

atom	U_{11}	U_{22}	U_{33}	U_{12}	U_{13}	U_{23}
C(46)	0.044(3)	0.028(2)	0.039(3)	-0.000(2)	0.002(2)	0.001(2)
C(47)	0.035(3)	0.068(4)	0.048(3)	-0.018(3)	0.005(2)	0.024(3)
C(48)	0.089(4)	0.061(3)	0.062(3)	0.009(3)	0.041(3)	0.001(2)
C(49)	0.024(3)	0.056(3)	0.065(3)	0.008(2)	0.005(2)	-0.002(2)
C(51)	0.052(3)	0.042(3)	0.058(3)	-0.004(2)	0.028(2)	-0.019(2)
C(52)	0.031(3)	0.061(3)	0.040(2)	-0.002(2)	0.004(2)	-0.002(2)
C(53)	0.064(3)	0.068(3)	0.091(3)	-0.006(2)	0.044(3)	-0.017(3)
C(54)	0.085(4)	0.093(4)	0.059(3)	-0.009(3)	0.025(3)	-0.033(3)
C(56)	0.031(2)	0.057(3)	0.040(2)	-0.003(2)	0.014(2)	-0.001(2)
C(57)	0.055(3)	0.059(3)	0.036(2)	0.015(2)	0.014(2)	0.003(2)
C(58)	0.066(3)	0.048(3)	0.065(3)	0.009(2)	0.015(2)	0.001(2)
C(59)	0.080(4)	0.054(3)	0.098(4)	0.007(3)	0.043(3)	-0.023(3)
C(60)	0.043(3)	0.060(3)	0.042(3)	0.007(2)	0.006(2)	0.003(2)
C(61)	0.034(3)	0.375(10)	0.099(4)	0.066(5)	0.007(3)	0.093(5)
C(62)	0.046(3)	0.118(4)	0.077(3)	-0.032(3)	0.017(2)	0.000(3)
C(63)	0.094(4)	0.060(3)	0.085(4)	-0.012(3)	0.050(3)	0.010(3)
C(64)	0.094(5)	0.109(5)	0.196(6)	0.016(4)	-0.034(4)	0.080(5)
C(66)	0.062(4)	0.089(4)	0.043(3)	0.043(3)	-0.002(2)	0.017(3)
C(67)	0.078(4)	0.064(3)	0.138(5)	-0.042(3)	0.056(3)	-0.051(3)
C(70)	0.130(5)	0.134(5)	0.184(6)	0.065(4)	-0.113(4)	-0.108(5)

The general temperature factor expression: $\exp(-2\pi^2(a^{*2}U_{11}h^2 + b^{*2}U_{22}k^2 + c^{*2}U_{33}l^2 + 2a^*b^*U_{12}hk + 2a^*c^*U_{13}hl + 2b^*c^*U_{23}kl))$

Table 3. Bond lengths (Å)

atom	atom	distance	atom	atom	distance
O(1)	C(46)	1.230(6)	O(2)	C(36)	1.225(6)
O(3)	C(37)	1.210(4)	O(4)	C(2)	1.225(4)
O(5)	C(29)	1.403(6)	O(5)	C(71)	1.249(10)
O(6)	C(32)	1.243(5)	O(8)	C(40)	1.362(5)
O(8)	C(55)	1.463(4)	O(9)	C(50)	1.229(5)
O(10)	C(47)	1.396(6)	O(10)	C(65)	1.422(5)
O(11)	C(66)	1.421(5)	O(11)	C(69)	1.519(11)
N(1)	C(27)	1.489(5)	N(1)	C(37)	1.366(5)
N(1)	C(52)	1.502(4)	N(2)	C(17)	1.508(5)
N(2)	C(36)	1.376(5)	N(2)	C(56)	1.470(5)
N(3)	C(23)	1.457(4)	N(3)	C(46)	1.348(5)
N(3)	H(21)	0.950	N(13)	C(2)	1.339(5)
N(13)	C(26)	1.459(5)	N(13)	H(22)	0.950
C(1)	C(33)	1.361(7)	C(1)	C(35)	1.342(6)
C(1)	H(1)	0.950	C(2)	C(25)	1.536(6)
C(16)	C(21)	1.371(6)	C(16)	C(27)	1.489(5)
C(16)	C(34)	1.385(5)	C(17)	C(26)	1.557(5)
C(17)	C(35)	1.503(6)	C(17)	H(13)	0.950
C(18)	C(20)	1.403(6)	C(18)	C(40)	1.392(6)
C(18)	H(2)	0.950	C(19)	C(29)	1.399(6)
C(19)	C(31)	1.406(6)	C(19)	H(3)	0.950
C(20)	C(23)	1.526(5)	C(20)	C(24)	1.404(6)
C(21)	C(57)	1.380(5)	C(21)	H(4)	0.950
C(22)	C(31)	1.420(6)	C(22)	C(39)	1.386(6)
C(22)	C(50)	1.458(6)	C(23)	C(27)	1.541(6)
C(23)	H(14)	0.950	C(24)	C(32)	1.460(6)
C(24)	C(49)	1.374(6)	C(25)	C(53)	1.521(6)
C(25)	C(62)	1.528(5)	C(25)	C(63)	1.529(6)
C(26)	C(31)	1.516(5)	C(26)	H(15)	0.950
C(27)	H(16)	0.950	C(29)	C(43)	1.392(6)
C(30)	C(44)	1.396(7)	C(30)	C(47)	1.360(7)
C(30)	H(5)	0.950	C(32)	H(71)	0.950
C(33)	C(47)	1.414(7)	C(33)	H(6)	0.950
C(34)	C(52)	1.500(6)	C(34)	C(60)	1.385(5)
C(35)	C(44)	1.355(7)	C(36)	C(51)	1.536(6)
C(37)	C(45)	1.542(6)	C(38)	C(40)	1.376(6)
C(38)	C(49)	1.366(6)	C(38)	H(7)	0.950

Table 3. Bond lengths (Å) (continued)

atom	atom	distance	atom	atom	distance
C(39)	C(43)	1.357(6)	C(39)	H(8)	0.950
C(41)	C(45)	1.525(6)	C(41)	H(23)	0.950
C(41)	H(24)	0.950	C(41)	H(25)	0.950
C(42)	C(46)	1.548(5)	C(42)	C(61)	1.466(8)
C(42)	C(64)	1.489(7)	C(42)	C(70)	1.480(7)
C(43)	H(9)	0.950	C(44)	C(56)	1.498(6)
C(45)	C(48)	1.545(5)	C(45)	C(58)	1.525(6)
C(48)	H(26)	0.950	C(48)	H(27)	0.950
C(48)	H(28)	0.950	C(49)	H(10)	0.950
C(50)	H(72)	0.950	C(51)	C(54)	1.525(6)
C(51)	C(59)	1.528(6)	C(51)	C(67)	1.520(6)
C(52)	H(17)	0.950	C(52)	H(18)	0.950
C(53)	H(29)	0.950	C(53)	H(30)	0.950
C(53)	H(31)	0.950	C(54)	H(32)	0.950
C(54)	H(33)	0.950	C(54)	H(34)	0.950
C(55)	H(35)	0.950	C(55)	H(36)	0.950
C(55)	H(37)	0.950	C(56)	H(19)	0.950
C(56)	H(20)	0.950	C(57)	C(66)	1.375(6)
C(57)	H(11)	0.950	C(58)	H(38)	0.950
C(58)	H(39)	0.950	C(58)	H(40)	0.950
C(59)	H(41)	0.950	C(59)	H(42)	0.950
C(59)	H(43)	0.950	C(60)	C(66)	1.375(7)
C(60)	H(12)	0.950	C(61)	H(44)	0.950
C(61)	H(45)	0.950	C(61)	H(46)	0.950
C(62)	H(47)	0.950	C(62)	H(48)	0.950
C(62)	H(49)	0.950	C(63)	H(50)	0.950
C(63)	H(51)	0.950	C(63)	H(52)	0.950
C(64)	H(53)	0.950	C(64)	H(54)	0.950
C(64)	H(55)	0.950	C(65)	H(56)	0.950
C(65)	H(57)	0.950	C(65)	H(58)	0.950
C(67)	H(59)	0.950	C(67)	H(60)	0.950
C(67)	H(61)	0.950	C(69)	H(62)	0.950
C(69)	H(63)	0.950	C(69)	H(64)	0.950
C(70)	H(65)	0.950	C(70)	H(66)	0.950
C(70)	H(67)	0.950	C(71)	H(68)	0.950
C(71)	H(69)	0.950	C(71)	H(70)	0.950

Table 4. Bond angles (^{o})

atom	atom	atom	angle	atom	atom	atom	angle
C(29)	O(5)	C(71)	117.7(5)	C(40)	O(8)	C(55)	118.1(3)
C(47)	O(10)	C(65)	114.8(3)	C(66)	O(11)	C(69)	112.1(4)
C(27)	N(1)	C(37)	119.9(3)	C(27)	N(1)	C(52)	110.2(3)
C(37)	N(1)	C(52)	129.9(3)	C(17)	N(2)	C(36)	118.9(3)
C(17)	N(2)	C(56)	109.5(3)	C(36)	N(2)	C(56)	131.2(3)
C(23)	N(3)	C(46)	120.8(3)	C(23)	N(3)	H(21)	119.1
C(46)	N(3)	H(21)	120.1	C(2)	N(13)	C(26)	120.4(2)
C(2)	N(13)	H(22)	120.7	C(26)	N(13)	H(22)	118.9
C(33)	C(1)	C(35)	118.9(4)	C(33)	C(1)	H(1)	121.2
C(35)	C(1)	H(1)	119.9	O(4)	C(2)	N(13)	122.5(4)
O(4)	C(2)	C(25)	121.1(4)	N(13)	C(2)	C(25)	116.3(3)
C(21)	C(16)	C(27)	129.8(3)	C(21)	C(16)	C(34)	119.6(3)
C(27)	C(16)	C(34)	110.5(3)	N(2)	C(17)	C(26)	114.1(3)
N(2)	C(17)	C(35)	102.3(3)	N(2)	C(17)	H(13)	108.4
C(26)	C(17)	C(35)	112.5(3)	C(26)	C(17)	H(13)	109.7
C(35)	C(17)	H(13)	109.7	C(20)	C(18)	C(40)	120.3(4)
C(20)	C(18)	H(2)	119.9	C(40)	C(18)	H(2)	119.8
C(29)	C(19)	C(31)	120.3(4)	C(29)	C(19)	H(3)	119.9
C(31)	C(19)	H(3)	119.8	C(18)	C(20)	C(23)	117.2(3)
C(18)	C(20)	C(24)	118.7(3)	C(23)	C(20)	C(24)	123.8(3)
C(16)	C(21)	C(57)	120.7(3)	C(16)	C(21)	H(4)	119.0
C(57)	C(21)	H(4)	120.3	C(31)	C(22)	C(39)	119.6(4)
C(31)	C(22)	C(50)	121.5(4)	C(39)	C(22)	C(50)	118.8(4)
N(3)	C(23)	C(20)	113.6(3)	N(3)	C(23)	C(27)	110.1(3)
N(3)	C(23)	H(14)	105.7	C(20)	C(23)	C(27)	111.6(2)
C(20)	C(23)	H(14)	107.1	C(27)	C(23)	H(14)	108.5
C(20)	C(24)	C(32)	120.3(3)	C(20)	C(24)	C(49)	118.9(4)
C(32)	C(24)	C(49)	120.7(4)	C(2)	C(25)	C(53)	109.5(3)
C(2)	C(25)	C(62)	113.6(3)	C(2)	C(25)	C(63)	106.1(3)
C(53)	C(25)	C(62)	109.0(3)	C(53)	C(25)	C(63)	109.0(3)
C(62)	C(25)	C(63)	109.6(3)	N(13)	C(26)	C(17)	108.5(2)
N(13)	C(26)	C(31)	112.7(3)	N(13)	C(26)	H(15)	107.5
C(17)	C(26)	C(31)	111.1(3)	C(17)	C(26)	H(15)	108.4
C(31)	C(26)	H(15)	108.5	N(1)	C(27)	C(16)	102.5(2)
N(1)	C(27)	C(23)	115.5(3)	N(1)	C(27)	H(16)	108.7
C(16)	C(27)	C(23)	111.4(3)	C(16)	C(27)	H(16)	108.9
C(23)	C(27)	H(16)	109.5	O(5)	C(29)	C(19)	113.1(4)

Table 4. Bond angles (^{o}) (continued)

atom	atom	atom	angle	atom	atom	atom	angle
O(5)	C(29)	C(43)	127.0(4)	C(19)	C(29)	C(43)	119.9(4)
C(44)	C(30)	C(47)	116.5(4)	C(44)	C(30)	H(5)	122.4
C(47)	C(30)	H(5)	121.1	C(19)	C(31)	C(22)	118.3(3)
C(19)	C(31)	C(26)	119.3(3)	C(22)	C(31)	C(26)	122.2(3)
O(6)	C(32)	C(24)	121.5(3)	O(6)	C(32)	H(71)	119.1
C(24)	C(32)	H(71)	119.4	C(1)	C(33)	C(47)	120.6(4)
C(1)	C(33)	H(6)	120.5	C(47)	C(33)	H(6)	118.9
C(16)	C(34)	C(52)	111.3(3)	C(16)	C(34)	C(60)	122.0(4)
C(52)	C(34)	C(60)	126.6(3)	C(1)	C(35)	C(17)	128.6(4)
C(1)	C(35)	C(44)	121.1(4)	C(17)	C(35)	C(44)	110.3(4)
O(2)	C(36)	N(2)	119.4(3)	O(2)	C(36)	C(51)	121.4(4)
N(2)	C(36)	C(51)	119.2(4)	O(3)	C(37)	N(1)	119.0(4)
O(3)	C(37)	C(45)	121.7(4)	N(1)	C(37)	C(45)	119.3(3)
C(40)	C(38)	C(49)	119.1(4)	C(40)	C(38)	H(7)	120.2
C(49)	C(38)	H(7)	120.7	C(22)	C(39)	C(43)	121.7(4)
C(22)	C(39)	H(8)	118.3	C(43)	C(39)	H(8)	119.9
O(8)	C(40)	C(18)	114.8(4)	O(8)	C(40)	C(38)	124.9(4)
C(18)	C(40)	C(38)	120.3(4)	C(45)	C(41)	H(23)	108.4
C(45)	C(41)	H(24)	110.8	C(45)	C(41)	H(25)	109.2
H(23)	C(41)	H(24)	109.5	H(23)	C(41)	H(25)	109.5
H(24)	C(41)	H(25)	109.5	C(46)	C(42)	C(61)	115.3(3)
C(46)	C(42)	C(64)	108.4(3)	C(46)	C(42)	C(70)	108.1(3)
C(61)	C(42)	C(64)	106.9(4)	C(61)	C(42)	C(70)	109.9(4)
C(64)	C(42)	C(70)	108.0(4)	C(29)	C(43)	C(39)	120.2(4)
C(29)	C(43)	H(9)	119.7	C(39)	C(43)	H(9)	120.2
C(30)	C(44)	C(35)	122.4(4)	C(30)	C(44)	C(56)	125.8(4)
C(35)	C(44)	C(56)	111.8(4)	C(37)	C(45)	C(41)	111.4(3)
C(37)	C(45)	C(48)	106.4(3)	C(37)	C(45)	C(58)	113.5(4)
C(41)	C(45)	C(48)	107.3(4)	C(41)	C(45)	C(58)	110.9(3)
C(48)	C(45)	C(58)	106.9(3)	O(1)	C(46)	N(3)	122.1(3)
O(1)	C(46)	C(42)	122.6(4)	N(3)	C(46)	C(42)	115.3(4)
O(10)	C(47)	C(30)	124.6(4)	O(10)	C(47)	C(33)	114.8(4)
C(30)	C(47)	C(33)	120.5(4)	C(45)	C(48)	H(26)	109.2
C(45)	C(48)	H(27)	110.4	C(45)	C(48)	H(28)	108.8
H(26)	C(48)	H(27)	109.5	H(26)	C(48)	H(28)	109.5
H(27)	C(48)	H(28)	109.5	C(24)	C(49)	C(38)	122.7(4)
C(24)	C(49)	H(10)	117.5	C(38)	C(49)	H(10)	119.7

Table 4. Bond angles (°) (continued)

atom	atom	atom	angle	atom	atom	atom	angle
O(9)	C(50)	C(22)	121.2(4)	O(9)	C(50)	H(72)	119.4
C(22)	C(50)	H(72)	119.4	C(36)	C(51)	C(54)	110.8(3)
C(36)	C(51)	C(59)	112.9(3)	C(36)	C(51)	C(67)	106.2(4)
C(54)	C(51)	C(59)	110.3(4)	C(54)	C(51)	C(67)	107.9(3)
C(59)	C(51)	C(67)	108.6(3)	N(1)	C(52)	C(34)	101.6(2)
N(1)	C(52)	H(17)	111.0	N(1)	C(52)	H(18)	110.8
C(34)	C(52)	H(17)	112.0	C(34)	C(52)	H(18)	111.9
H(17)	C(52)	H(18)	109.5	C(25)	C(53)	H(29)	110.2
C(25)	C(53)	H(30)	109.6	C(25)	C(53)	H(31)	108.6
H(29)	C(53)	H(30)	109.5	H(29)	C(53)	H(31)	109.5
H(30)	C(53)	H(31)	109.5	C(51)	C(54)	H(32)	108.5
C(51)	C(54)	H(33)	111.7	C(51)	C(54)	H(34)	108.2
H(32)	C(54)	H(33)	109.5	H(32)	C(54)	H(34)	109.5
H(33)	C(54)	H(34)	109.5	O(8)	C(55)	H(35)	109.2
O(8)	C(55)	H(36)	109.8	O(8)	C(55)	H(37)	109.4
H(35)	C(55)	H(36)	109.5	H(35)	C(55)	H(37)	109.5
H(36)	C(55)	H(37)	109.5	N(2)	C(56)	C(44)	102.8(3)
N(2)	C(56)	H(19)	111.4	N(2)	C(56)	H(20)	109.8
C(44)	C(56)	H(19)	111.9	C(44)	C(56)	H(20)	111.4
H(19)	C(56)	H(20)	109.5	C(21)	C(57)	C(66)	117.4(4)
C(21)	C(57)	H(11)	122.1	C(66)	C(57)	H(11)	120.5
C(45)	C(58)	H(38)	110.3	C(45)	C(58)	H(39)	110.2
C(45)	C(58)	H(40)	107.8	H(38)	C(58)	H(39)	109.5
H(38)	C(58)	H(40)	109.5	H(39)	C(58)	H(40)	109.5
C(51)	C(59)	H(41)	111.1	C(51)	C(59)	H(42)	110.7
C(51)	C(59)	H(43)	106.6	H(41)	C(59)	H(42)	109.5
H(41)	C(59)	H(43)	109.5	H(42)	C(59)	H(43)	109.5
C(34)	C(60)	C(66)	115.5(3)	C(34)	C(60)	H(12)	122.2
C(66)	C(60)	H(12)	122.3	C(42)	C(61)	H(44)	111.6
C(42)	C(61)	H(45)	107.7	C(42)	C(61)	H(46)	109.1
H(44)	C(61)	H(45)	109.5	H(44)	C(61)	H(46)	109.5
H(45)	C(61)	H(46)	109.5	C(25)	C(62)	H(47)	110.6
C(25)	C(62)	H(48)	108.6	C(25)	C(62)	H(49)	109.1
H(47)	C(62)	H(48)	109.5	H(47)	C(62)	H(49)	109.5
H(48)	C(62)	H(49)	109.5	C(25)	C(63)	H(50)	109.2
C(25)	C(63)	H(51)	110.1	C(25)	C(63)	H(52)	109.1
H(50)	C(63)	H(51)	109.5	H(50)	C(63)	H(52)	109.5

Table 4. Bond angles (^{o}) (continued)

atom	atom	atom	angle	atom	atom	atom	angle
H(51)	C(63)	H(52)	109.5	C(42)	C(64)	H(53)	109.4
C(42)	C(64)	H(54)	110.6	C(42)	C(64)	H(55)	108.4
H(53)	C(64)	H(54)	109.5	H(53)	C(64)	H(55)	109.5
H(54)	C(64)	H(55)	109.5	O(10)	C(65)	H(56)	108.7
O(10)	C(65)	H(57)	109.3	O(10)	C(65)	H(58)	110.4
H(56)	C(65)	H(57)	109.5	H(56)	C(65)	H(58)	109.5
H(57)	C(65)	H(58)	109.5	O(11)	C(66)	C(57)	120.0(4)
O(11)	C(66)	C(60)	115.2(3)	C(57)	C(66)	C(60)	124.8(4)
C(51)	C(67)	H(59)	109.6	C(51)	C(67)	H(60)	111.5
C(51)	C(67)	H(61)	107.3	H(59)	C(67)	H(60)	109.5
H(59)	C(67)	H(61)	109.5	H(60)	C(67)	H(61)	109.5
O(11)	C(69)	H(62)	112.1	O(11)	C(69)	H(63)	109.9
O(11)	C(69)	H(64)	106.3	H(62)	C(69)	H(63)	109.5
H(62)	C(69)	H(64)	109.5	H(63)	C(69)	H(64)	109.5
C(42)	C(70)	H(65)	112.4	C(42)	C(70)	H(66)	109.4
C(42)	C(70)	H(67)	106.6	H(65)	C(70)	H(66)	109.5
H(65)	C(70)	H(67)	109.5	H(66)	C(70)	H(67)	109.5
O(5)	C(71)	H(68)	108.3	O(5)	C(71)	H(69)	112.5
O(5)	C(71)	H(70)	107.6	H(68)	C(71)	H(69)	109.5
H(68)	C(71)	H(70)	109.5	H(69)	C(71)	H(70)	109.5

Table 5. Torsion Angles(o)

atom1	atom2	atom3	atom4	angle	atom1	atom2	atom3	atom4	angle
C(71)	O(5)	C(29)	C(19)	-174.4(6)	C(71)	O(5)	C(29)	C(43)	3.3(8)
C(55)	O(8)	C(40)	C(18)	-178.3(3)	C(55)	O(8)	C(40)	C(38)	3.8(5)
C(65)	O(10)	C(47)	C(30)	-0.1(4)	C(65)	O(10)	C(47)	C(33)	-177.4(3)
C(69)	O(11)	C(66)	C(57)	31.3(8)	C(69)	O(11)	C(66)	C(60)	-150.8(6)
C(27)	N(1)	C(37)	O(3)	-3.7(6)	C(27)	N(1)	C(37)	C(45)	174.4(4)
C(37)	N(1)	C(27)	C(16)	-157.5(4)	C(37)	N(1)	C(27)	C(23)	81.2(4)
C(27)	N(1)	C(52)	C(34)	-18.1(4)	C(52)	N(1)	C(27)	C(16)	19.9(4)
C(52)	N(1)	C(27)	C(23)	-101.4(3)	C(37)	N(1)	C(52)	C(34)	159.0(4)
C(52)	N(1)	C(37)	O(3)	179.4(4)	C(52)	N(1)	C(37)	C(45)	-2.5(7)
C(17)	N(2)	C(36)	O(2)	-5.4(5)	C(17)	N(2)	C(36)	C(51)	174.5(3)
C(36)	N(2)	C(17)	C(26)	83.4(4)	C(36)	N(2)	C(17)	C(35)	-154.8(3)
C(17)	N(2)	C(56)	C(44)	-17.6(3)	C(56)	N(2)	C(17)	C(26)	-103.2(3)
C(56)	N(2)	C(17)	C(35)	18.5(3)	C(36)	N(2)	C(56)	C(44)	154.7(3)
C(56)	N(2)	C(36)	O(2)	-177.1(3)	C(56)	N(2)	C(36)	C(51)	2.8(5)
C(23)	N(3)	C(46)	O(1)	-6.6(5)	C(23)	N(3)	C(46)	C(42)	172.9(3)
C(46)	N(3)	C(23)	C(20)	75.0(4)	C(46)	N(3)	C(23)	C(27)	-159.0(3)
C(2)	N(13)	C(26)	C(17)	-160.5(3)	C(2)	N(13)	C(26)	C(31)	76.1(4)
C(26)	N(13)	C(2)	O(4)	-2.6(5)	C(26)	N(13)	C(2)	C(25)	180.0(2)
C(33)	C(1)	C(35)	C(17)	177.9(3)	C(33)	C(1)	C(35)	C(44)	-1.2(5)
C(35)	C(1)	C(33)	C(47)	-0.4(5)	O(4)	C(2)	C(25)	C(53)	40.3(5)
O(4)	C(2)	C(25)	C(62)	162.5(3)	O(4)	C(2)	C(25)	C(63)	-77.1(4)
N(13)	C(2)	C(25)	C(53)	-142.2(3)	N(13)	C(2)	C(25)	C(62)	-20.1(5)
N(13)	C(2)	C(25)	C(63)	100.3(3)	C(21)	C(16)	C(27)	N(1)	169.1(4)
C(21)	C(16)	C(27)	C(23)	-66.8(6)	C(27)	C(16)	C(21)	C(57)	177.7(4)
C(21)	C(16)	C(34)	C(52)	-179.6(4)	C(21)	C(16)	C(34)	C(60)	-0.2(6)
C(34)	C(16)	C(21)	C(57)	1.0(7)	C(27)	C(16)	C(34)	C(52)	3.1(5)
C(27)	C(16)	C(34)	C(60)	-177.5(4)	C(34)	C(16)	C(27)	N(1)	-13.9(5)
C(34)	C(16)	C(27)	C(23)	110.1(4)	N(2)	C(17)	C(26)	N(13)	-80.8(4)
N(2)	C(17)	C(26)	C(31)	43.6(4)	N(2)	C(17)	C(35)	C(1)	168.7(3)
N(2)	C(17)	C(35)	C(44)	-12.2(3)	C(26)	C(17)	C(35)	C(1)	-68.5(5)
C(26)	C(17)	C(35)	C(44)	110.6(3)	C(35)	C(17)	C(26)	N(13)	163.3(3)
C(35)	C(17)	C(26)	C(31)	-72.3(4)	C(20)	C(18)	C(40)	O(8)	-179.4(3)
C(20)	C(18)	C(40)	C(38)	-1.4(6)	C(40)	C(18)	C(20)	C(23)	174.7(3)
C(40)	C(18)	C(20)	C(24)	0.6(5)	C(29)	C(19)	C(31)	C(22)	-0.8(5)
C(29)	C(19)	C(31)	C(26)	174.7(3)	C(31)	C(19)	C(29)	O(5)	179.0(3)
C(31)	C(19)	C(29)	C(43)	1.1(6)	C(18)	C(20)	C(23)	N(3)	43.2(4)
C(18)	C(20)	C(23)	C(27)	-82.0(3)	C(18)	C(20)	C(24)	C(32)	-178.6(3)

Table 5. Torsion angles (^{o}) (continued)

atom1	atom2	atom3	atom4	angle	atom1	atom2	atom3	atom4	angle
C(18)	C(20)	C(24)	C(49)	-0.6(5)	C(23)	C(20)	C(24)	C(32)	7.7(5)
C(23)	C(20)	C(24)	C(49)	-174.3(3)	C(24)	C(20)	C(23)	N(3)	-143.0(3)
C(24)	C(20)	C(23)	C(27)	91.9(4)	C(16)	C(21)	C(57)	C(66)	-1.7(7)
C(31)	C(22)	C(39)	C(43)	2.0(6)	C(39)	C(22)	C(31)	C(19)	-0.7(5)
C(39)	C(22)	C(31)	C(26)	-176.1(3)	C(31)	C(22)	C(50)	O(9)	-168.1(3)
C(50)	C(22)	C(31)	C(19)	-176.1(3)	C(50)	C(22)	C(31)	C(26)	8.6(6)
C(39)	C(22)	C(50)	O(9)	16.5(5)	C(50)	C(22)	C(39)	C(43)	177.5(4)
N(3)	C(23)	C(27)	N(1)	-79.2(3)	N(3)	C(23)	C(27)	C(16)	164.4(3)
C(20)	C(23)	C(27)	N(1)	47.9(4)	C(20)	C(23)	C(27)	C(16)	-68.5(4)
C(20)	C(24)	C(32)	O(6)	-166.2(4)	C(20)	C(24)	C(49)	C(38)	1.5(6)
C(32)	C(24)	C(49)	C(38)	179.5(3)	C(49)	C(24)	C(32)	O(6)	15.9(6)
N(13)	C(26)	C(31)	C(19)	34.7(4)	N(13)	C(26)	C(31)	C(22)	-150.0(3)
C(17)	C(26)	C(31)	C(19)	-87.3(4)	C(17)	C(26)	C(31)	C(22)	88.0(4)
O(5)	C(29)	C(43)	C(39)	-177.5(4)	C(19)	C(29)	C(43)	C(39)	0.2(5)
C(44)	C(30)	C(47)	O(10)	-179.5(3)	C(44)	C(30)	C(47)	C(33)	-2.3(5)
C(47)	C(30)	C(44)	C(35)	0.8(5)	C(47)	C(30)	C(44)	C(56)	-179.1(3)
C(1)	C(33)	C(47)	O(10)	179.7(3)	C(1)	C(33)	C(47)	C(30)	2.3(6)
C(16)	C(34)	C(52)	N(1)	9.1(5)	C(16)	C(34)	C(60)	C(66)	0.2(6)
C(52)	C(34)	C(60)	C(66)	179.5(4)	C(60)	C(34)	C(52)	N(1)	-170.3(4)
C(1)	C(35)	C(44)	C(30)	1.0(5)	C(1)	C(35)	C(44)	C(56)	-179.1(3)
C(17)	C(35)	C(44)	C(30)	-178.2(3)	C(17)	C(35)	C(44)	C(56)	1.7(4)
O(2)	C(36)	C(51)	C(54)	106.7(4)	O(2)	C(36)	C(51)	C(59)	-129.1(4)
O(2)	C(36)	C(51)	C(67)	-10.2(5)	N(2)	C(36)	C(51)	C(54)	-73.3(4)
N(2)	C(36)	C(51)	C(59)	51.0(5)	N(2)	C(36)	C(51)	C(67)	169.9(3)
O(3)	C(37)	C(45)	C(41)	106.8(5)	O(3)	C(37)	C(45)	C(48)	-9.9(6)
O(3)	C(37)	C(45)	C(58)	-127.1(4)	N(1)	C(37)	C(45)	C(41)	-71.3(5)
N(1)	C(37)	C(45)	C(48)	172.0(4)	N(1)	C(37)	C(45)	C(58)	54.8(5)
C(40)	C(38)	C(49)	C(24)	-2.3(6)	C(49)	C(38)	C(40)	O(8)	-180.0(3)
C(49)	C(38)	C(40)	C(18)	2.2(6)	C(22)	C(39)	C(43)	C(29)	-1.7(6)
C(61)	C(42)	C(46)	O(1)	166.4(4)	C(61)	C(42)	C(46)	N(3)	-13.0(6)
C(64)	C(42)	C(46)	O(1)	-73.8(5)	C(64)	C(42)	C(46)	N(3)	106.7(4)
C(70)	C(42)	C(46)	O(1)	43.0(6)	C(70)	C(42)	C(46)	N(3)	-136.4(4)
C(30)	C(44)	C(56)	N(2)	-170.2(3)	C(35)	C(44)	C(56)	N(2)	9.9(3)
C(21)	C(57)	C(66)	O(11)	179.4(4)	C(21)	C(57)	C(66)	C(60)	1.8(8)
C(34)	C(60)	C(66)	O(11)	-178.8(4)	C(34)	C(60)	C(66)	C(57)	-1.0(8)

The sign is positive if when looking from atom 2 to atom 3 a clock-wise motion of atom 1 would superimpose it on atom 4.

Table 6. Distances beyond the asymmetric unit out to 3.60 Å

atom	atom	distance	atom	atom	distance
O(1)	O(4)	3.441(3)	O(1)	N(13)	3.524(4)
O(1)	C(1)	3.393(5)	O(1)	C(26)	3.189(4)
O(1)	H(1)	2.564	O(1)	H(13)	3.496
O(1)	H(15)	2.375	O(1)	H(72)	3.152
O(2)	C(61)[1]	3.565(5)	O(2)	H(45)[1]	2.754
O(2)	H(54)[1]	3.289	O(2)	H(66)[1]	3.328
O(3)	H(51)[2]	3.496	O(4)	O(1)	3.441(3)
O(4)	C(21)	3.314(4)	O(4)	C(23)	3.276(4)
O(4)	C(32)	3.444(4)	O(4)	C(55)[3]	3.594(4)
O(4)	H(4)	2.513	O(4)	H(14)	2.354
O(4)	H(36)[3]	2.836	O(4)	H(71)	2.494
O(5)	H(6)[3]	3.154	O(5)	H(63)[4]	3.390
O(6)	O(9)	3.557(4)	O(6)	C(38)[4]	3.366(5)
O(6)	C(43)[5]	3.574(4)	O(6)	C(50)	3.410(4)
O(6)	H(7)[4]	2.548	O(6)	H(9)[5]	2.952
O(6)	H(10)[4]	3.539	O(6)	H(35)[3]	3.588
O(6)	H(36)[3]	3.068	O(6)	H(37)[4]	3.157
O(6)	H(70)[5]	3.060	O(6)	H(72)	3.422
O(8)	C(63)[2]	3.523(4)	O(8)	H(11)[2]	3.514
O(8)	H(52)[2]	2.593	O(9)	O(6)	3.557(4)
O(9)	C(65)[6]	3.118(6)	O(9)	C(69)[5]	3.465(11)
O(9)	H(7)[4]	3.055	O(9)	H(35)[4]	3.463
O(9)	H(56)[6]	2.993	O(9)	H(57)[6]	2.832
O(9)	H(58)[6]	3.001	O(9)	H(62)[5]	3.134
O(9)	H(63)[5]	3.313	O(9)	H(64)[5]	3.380
O(9)	H(68)[2]	3.591	O(9)	H(69)[2]	3.129
O(10)	C(52)[7]	3.513(4)	O(10)	C(60)[7]	3.492(5)
O(10)	C(69)[5]	3.451(9)	O(10)	H(12)[7]	2.666
O(10)	H(17)[7]	3.303	O(10)	H(18)[7]	3.075
O(10)	H(56)[6]	3.240	O(10)	H(62)[5]	3.393
O(10)	H(63)[5]	2.838	O(11)	H(5)[8]	3.396
O(11)	H(9)[5]	3.354	O(11)	H(19)[8]	3.462
O(11)	H(20)[8]	3.580	O(11)	H(68)[5]	3.130
N(1)	H(33)[8]	3.554	N(3)	H(31)	3.220
N(13)	O(1)	3.524(4)	N(13)	H(67)	3.161
C(1)	O(1)	3.393(5)	C(1)	H(24)[7]	3.071
C(2)	H(4)	3.515	C(2)	H(14)	3.221

Table 6. Distances beyond the asymmetric unit out to 3.60 Å (continued)

atom	atom	distance	atom	atom	distance
C(2)	H(67)	3.427	C(16)	H(33)[8]	2.805
C(16)	H(41)[8]	3.539	C(17)	H(24)[7]	3.561
C(18)	H(52)[2]	3.216	C(19)	H(36)[3]	3.485
C(21)	O(4)	3.314(4)	C(21)	H(33)[8]	3.031
C(21)	H(36)[3]	3.548	C(22)	H(36)[3]	3.343
C(22)	H(57)[6]	3.337	C(23)	O(4)	3.276(4)
C(23)	H(31)	3.395	C(24)	H(69)[2]	3.201
C(24)	H(72)	3.482	C(26)	O(1)	3.189(4)
C(27)	H(31)	3.448	C(27)	H(33)[8]	3.228
C(29)	H(37)[3]	3.243	C(30)	H(38)[7]	2.835
C(30)	H(56)[6]	2.811	C(30)	H(62)[9]	3.193
C(31)	H(36)[3]	3.328	C(32)	O(4)	3.444(4)
C(32)	C(50)	3.302(6)	C(32)	H(36)[3]	3.248
C(32)	H(72)	2.974	C(33)	H(18)[7]	3.247
C(33)	H(24)[7]	3.473	C(33)	H(42)[2]	3.343
C(33)	H(63)[5]	3.032	C(33)	H(68)[2]	3.031
C(33)	H(69)[2]	3.546	C(34)	H(33)[8]	3.242
C(34)	H(41)[8]	3.035	C(34)	H(42)[8]	3.366
C(35)	H(24)[7]	2.986	C(36)	H(45)[1]	3.226
C(38)	O(6)[5]	3.366(5)	C(38)	C(71)[2]	3.434(10)
C(38)	H(8)[5]	3.103	C(38)	H(69)[2]	3.196
C(38)	H(70)[2]	3.008	C(39)	C(49)[4]	3.487(5)
C(39)	C(65)[6]	3.547(7)	C(39)	H(10)[4]	2.969
C(39)	H(36)[3]	3.503	C(39)	H(37)[3]	3.316
C(39)	H(57)[6]	2.676	C(40)	H(52)[2]	3.373
C(41)	H(42)[8]	3.464	C(41)	H(60)[8]	3.591
C(43)	O(6)[4]	3.574(4)	C(43)	H(10)[4]	3.273
C(43)	H(37)[3]	3.063	C(43)	H(57)[6]	3.481
C(44)	H(24)[7]	3.307	C(44)	H(38)[7]	3.173
C(44)	H(56)[6]	3.574	C(46)	H(15)	3.414
C(46)	H(31)	3.541	C(47)	H(18)[7]	3.085
C(47)	H(38)[7]	3.185	C(47)	H(56)[6]	3.020
C(47)	H(63)[5]	3.188	C(48)	H(30)[10]	3.436
C(48)	H(44)[10]	3.552	C(48)	H(51)[2]	3.079
C(49)	C(39)[5]	3.487(5)	C(49)	C(71)[2]	3.494(10)
C(49)	H(8)[5]	2.878	C(49)	H(37)[4]	3.478
C(49)	H(69)[2]	2.935	C(49)	H(70)[2]	3.235

Table 6. Distances beyond the asymmetric unit out to 3.60 Å (continued)

atom	atom	distance	atom	atom	distance
C(50)	O(6)	3.410(4)	C(50)	C(32)	3.302(6)
C(50)	H(57)[6)]	3.441	C(50)	H(69)[2)]	3.355
C(50)	H(71)	3.216	C(51)	H(45)[1)]	3.487
C(52)	O(10)[11)]	3.513(4)	C(52)	H(42)[8)]	3.403
C(53)	H(4)	3.400	C(53)	H(16)	3.468
C(53)	H(26)[3)]	3.474	C(53)	H(28)[12)]	3.488
C(54)	H(39)[7)]	3.586	C(54)	H(45)[1)]	3.357
C(55)	O(4)[2)]	3.594(4)	C(55)	H(4)[2)]	3.592
C(55)	H(10)[5)]	3.199	C(55)	H(11)[2)]	3.099
C(55)	H(64)[2)]	3.561	C(57)	H(20)[8)]	3.264
C(57)	H(35)[3)]	3.047	C(57)	H(36)[3)]	3.549
C(57)	H(41)[8)]	3.581	C(59)	C(60)[9)]	3.573(7)
C(59)	H(6)[3)]	3.285	C(59)	H(18)[9)]	3.521
C(59)	H(25)[9)]	3.460	C(60)	O(10)[11)]	3.492(5)
C(60)	C(59)[8)]	3.573(7)	C(60)	H(9)[5)]	3.440
C(60)	H(41)[8)]	2.788	C(60)	H(42)[8)]	3.537
C(60)	H(57)[11)]	3.543	C(61)	O(2)[1)]	3.565(5)
C(61)	H(27)[12)]	3.381	C(61)	H(32)[1)]	3.322
C(61)	H(61)[1)]	3.461	C(62)	H(53)[3)]	3.514
C(62)	H(54)[1)]	3.588	C(62)	H(60)[13)]	3.145
C(62)	H(61)[13)]	3.513	C(62)	H(67)	3.452
C(63)	O(8)[3)]	3.523(4)	C(63)	H(2)[3)]	2.989
C(63)	H(26)[3)]	3.165	C(63)	H(40)[3)]	3.538
C(63)	H(53)[3)]	3.150	C(64)	H(50)[2)]	3.069
C(64)	H(59)[2)]	3.166	C(64)	H(61)[1)]	3.488
C(65)	O(9)[6)]	3.118(6)	C(65)	C(39)[6)]	3.547(7)
C(65)	H(5)[6)]	3.564	C(65)	H(8)[6)]	2.994
C(65)	H(12)[7)]	3.294	C(65)	H(17)[7)]	3.317
C(65)	H(56)[6)]	3.164	C(65)	H(62)[9)]	3.135
C(66)	H(9)[5)]	3.381	C(66)	H(20)[8)]	3.351
C(66)	H(41)[8)]	3.081	C(67)	H(25)[9)]	3.505
C(67)	H(45)[1)]	3.245	C(67)	H(48)[13)]	2.833
C(67)	H(54)[3)]	3.411	C(67)	H(55)[3)]	3.591
C(69)	O(9)[4)]	3.465(11)	C(69)	O(10)[4)]	3.451(9)
C(69)	C(71)[5)]	2.948(16)	C(69)	H(5)[8)]	3.075
C(69)	H(6)[4)]	3.580	C(69)	H(9)[5)]	3.513
C(69)	H(35)[3)]	3.354	C(69)	H(58)[8)]	3.354

Table 6. Distances beyond the asymmetric unit out to 3.60 Å (continued)

atom	atom	distance	atom	atom	distance
C(69)	H(68)[5]	2.247	C(69)	H(69)[5]	3.284
C(69)	H(70)[5]	2.935	C(70)	H(13)	3.502
C(70)	H(65)[1]	3.549	C(70)	H(66)[1]	3.092
C(71)	C(38)[3]	3.434(10)	C(71)	C(49)[3]	3.494(10)
C(71)	C(69)[4]	2.948(16)	C(71)	H(6)[3]	3.019
C(71)	H(63)[4]	2.482	C(71)	H(64)[4]	2.577
H(1)	O(1)	2.564	H(1)	H(24)[7]	3.373
H(1)	H(55)	3.358	H(1)	H(65)	3.413
H(1)	H(69)[2]	3.428	H(2)	C(63)[2]	2.989
H(2)	H(50)[2]	2.899	H(2)	H(51)[2]	3.061
H(2)	H(52)[2]	2.525	H(4)	O(4)	2.513
H(4)	C(2)	3.515	H(4)	C(53)	3.400
H(4)	C(55)[3]	3.592	H(4)	H(29)	2.822
H(4)	H(31)	3.252	H(4)	H(33)[8]	3.320
H(4)	H(35)[3]	3.459	H(4)	H(36)[3]	3.140
H(4)	H(39)[3]	3.593	H(4)	H(52)	3.507
H(5)	O(11)[9]	3.396	H(5)	C(65)[6]	3.564
H(5)	C(69)[9]	3.075	H(5)	H(11)[9]	3.206
H(5)	H(38)[7]	2.964	H(5)	H(56)[6]	2.693
H(5)	H(62)[9]	2.246	H(5)	H(63)[9]	3.564
H(6)	O(5)[2]	3.154	H(6)	C(59)[2]	3.285
H(6)	C(69)[5]	3.580	H(6)	C(71)[2]	3.019
H(6)	H(12)[7]	3.459	H(6)	H(18)[7]	3.300
H(6)	H(41)[2]	3.495	H(6)	H(42)[2]	2.832
H(6)	H(43)[2]	3.033	H(6)	H(63)[5]	2.632
H(6)	H(68)[2]	2.387	H(6)	H(69)[2]	3.157
H(7)	O(6)[5]	2.548	H(7)	O(9)[5]	3.055
H(7)	H(8)[5]	3.010	H(7)	H(10)[5]	3.540
H(7)	H(69)[2]	3.572	H(7)	H(70)[2]	2.957
H(8)	C(38)[4]	3.103	H(8)	C(49)[4]	2.878
H(8)	C(65)[6]	2.994	H(8)	H(7)[4]	3.010
H(8)	H(10)[4]	2.594	H(8)	H(56)[6]	3.321
H(8)	H(57)[6]	2.099	H(8)	H(58)[6]	3.294
H(9)	O(6)[4]	2.952	H(9)	O(11)[4]	3.354
H(9)	C(60)[4]	3.440	H(9)	C(66)[4]	3.381
H(9)	C(69)[4]	3.513	H(9)	H(10)[4]	3.199
H(9)	H(12)[4]	3.417	H(9)	H(37)[3]	3.336

Table 6. Distances beyond the asymmetric unit out to 3.60 Å (continued)

atom	atom	distance	atom	atom	distance
H(9)	H(57)[6]	3.557	H(9)	H(63)[4]	3.524
H(9)	H(64)[4]	3.072	H(10)	O(6)[5]	3.539
H(10)	C(39)[5]	2.969	H(10)	C(43)[5]	3.273
H(10)	C(55)[4]	3.199	H(10)	H(7)[4]	3.540
H(10)	H(8)[5]	2.594	H(10)	H(9)[5]	3.199
H(10)	H(35)[4]	3.294	H(10)	H(36)[4]	3.305
H(10)	H(37)[4]	2.537	H(10)	H(69)[2]	3.167
H(10)	H(70)[2]	3.336	H(11)	O(8)[3]	3.514
H(11)	C(55)[3]	3.099	H(11)	H(5)[8]	3.206
H(11)	H(20)[8]	3.298	H(11)	H(35)[3]	2.379
H(11)	H(36)[3]	3.196	H(11)	H(39)[3]	3.464
H(11)	H(40)[3]	3.595	H(12)	O(10)[11]	2.666
H(12)	C(65)[11]	3.294	H(12)	H(6)[11]	3.459
H(12)	H(9)[5]	3.417	H(12)	H(41)[8]	3.018
H(12)	H(42)[8]	3.599	H(12)	H(57)[11]	2.899
H(13)	O(1)	3.496	H(13)	C(70)	3.502
H(13)	H(24)[7]	3.225	H(13)	H(65)	3.072
H(13)	H(66)[1]	3.433	H(13)	H(67)	3.035
H(14)	O(4)	2.354	H(14)	C(2)	3.221
H(14)	H(15)	3.306	H(14)	H(31)	3.004
H(15)	O(1)	2.375	H(15)	C(46)	3.414
H(15)	H(14)	3.306	H(15)	H(67)	3.470
H(15)	H(71)	3.168	H(16)	C(53)	3.468
H(16)	H(29)	3.304	H(16)	H(31)	2.787
H(16)	H(33)[8]	2.838	H(17)	O(10)[11]	3.303
H(17)	C(65)[11]	3.317	H(17)	H(57)[11]	3.098
H(17)	H(58)[11]	3.072	H(18)	O(10)[11]	3.075
H(18)	C(33)[11]	3.247	H(18)	C(47)[11]	3.085
H(18)	C(59)[8]	3.521	H(18)	H(6)[11]	3.300
H(18)	H(41)[8]	3.414	H(18)	H(42)[8]	2.781
H(18)	H(58)[11]	3.600	H(19)	O(11)[9]	3.462
H(20)	O(11)[9]	3.580	H(20)	C(57)[9]	3.264
H(20)	C(66)[9]	3.351	H(20)	H(11)[9]	3.298
H(20)	H(38)[7]	3.440	H(20)	H(39)[7]	3.077
H(21)	H(31)	3.435	H(22)	H(67)	3.183
H(23)	H(30)[10]	2.908	H(23)	H(44)[10]	3.501
H(23)	H(47)[10]	2.860	H(23)	H(48)[10]	3.573

Table 6. Distances beyond the asymmetric unit out to 3.60 Å (continued)

atom	atom	distance	atom	atom	distance
H(23)	H(60)[8]	3.282	H(24)	C(1)[11]	3.071
H(24)	C(17)[11]	3.561	H(24)	C(33)[11]	3.473
H(24)	C(35)[11]	2.986	H(24)	C(44)[11]	3.307
H(24)	H(1)[11]	3.373	H(24)	H(13)[11]	3.225
H(25)	C(59)[8]	3.460	H(25)	C(67)[8]	3.505
H(25)	H(42)[8]	2.555	H(25)	H(59)[8]	3.302
H(25)	H(60)[8]	3.006	H(26)	C(53)[2]	3.474
H(26)	C(63)[2]	3.165	H(26)	H(29)[2]	2.790
H(26)	H(30)[2]	3.469	H(26)	H(51)[2]	2.285
H(26)	H(52)[2]	3.349	H(27)	C(61)[10]	3.381
H(27)	H(30)[10]	3.323	H(27)	H(34)[11]	3.565
H(27)	H(44)[10]	2.613	H(27)	H(46)[10]	3.396
H(28)	C(53)[10]	3.488	H(28)	H(30)[10]	2.790
H(28)	H(31)[10]	3.489	H(28)	H(51)[2]	3.180
H(29)	H(4)	2.822	H(29)	H(16)	3.304
H(29)	H(26)[3]	2.790	H(29)	H(39)[3]	3.571
H(30)	C(48)[12]	3.436	H(30)	H(23)[12]	2.908
H(30)	H(26)[3]	3.469	H(30)	H(27)[12]	3.323
H(30)	H(28)[12]	2.790	H(31)	N(3)	3.220
H(31)	C(23)	3.395	H(31)	C(27)	3.448
H(31)	C(46)	3.541	H(31)	H(4)	3.252
H(31)	H(14)	3.004	H(31)	H(16)	2.787
H(31)	H(21)	3.435	H(31)	H(28)[12]	3.489
H(31)	H(44)	3.487	H(31)	H(67)	3.457
H(32)	C(61)[1]	3.322	H(32)	H(44)[1]	3.496
H(32)	H(45)[1]	2.723	H(32)	H(46)[1]	3.260
H(33)	N(1)[9]	3.554	H(33)	C(16)[9]	2.805
H(33)	C(21)[9]	3.031	H(33)	C(27)[9]	3.228
H(33)	C(34)[9]	3.242	H(33)	H(4)[9]	3.320
H(33)	H(16)[9]	2.838	H(34)	H(27)[7]	3.565
H(34)	H(39)[7]	2.710	H(34)	H(45)[1]	3.468
H(35)	O(6)[2]	3.588	H(35)	O(9)[5]	3.463
H(35)	C(57)[2]	3.047	H(35)	C(69)[2]	3.354
H(35)	H(4)[2]	3.459	H(35)	H(10)[5]	3.294
H(35)	H(11)[2]	2.379	H(35)	H(62)[2]	3.192
H(35)	H(64)[2]	2.664	H(36)	O(4)[2]	2.836
H(36)	O(6)[2]	3.068	H(36)	C(19)[2]	3.485

Table 6. Distances beyond the asymmetric unit out to 3.60 Å (continued)

atom	atom	distance	atom	atom	distance
H(36)	C(21)[2]	3.548	H(36)	C(22)[2]	3.343
H(36)	C(31)[2]	3.328	H(36)	C(32)[2]	3.248
H(36)	C(39)[2]	3.503	H(36)	C(57)[2]	3.549
H(36)	H(4)[2]	3.140	H(36)	H(10)[5]	3.305
H(36)	H(11)[2]	3.196	H(36)	H(52)[2]	3.518
H(36)	H(71)[2]	2.716	H(37)	O(6)[5]	3.157
H(37)	C(29)[2]	3.243	H(37)	C(39)[2]	3.316
H(37)	C(43)[2]	3.063	H(37)	C(49)[5]	3.478
H(37)	H(9)[2]	3.336	H(37)	H(10)[5]	2.537
H(37)	H(70)[2]	3.444	H(38)	C(30)[11]	2.835
H(38)	C(44)[11]	3.173	H(38)	C(47)[11]	3.185
H(38)	H(5)[11]	2.964	H(38)	H(20)[11]	3.440
H(38)	H(58)[11]	3.293	H(39)	C(54)[11]	3.586
H(39)	H(4)[2]	3.593	H(39)	H(11)[2]	3.464
H(39)	H(20)[11]	3.077	H(39)	H(29)[2]	3.571
H(39)	H(34)[11]	2.710	H(40)	C(63)[2]	3.538
H(40)	H(11)[2]	3.595	H(40)	H(51)[2]	3.166
H(40)	H(52)[2]	3.019	H(41)	C(16)[9]	3.539
H(41)	C(34)[9]	3.035	H(41)	C(57)[9]	3.581
H(41)	C(60)[9]	2.788	H(41)	C(66)[9]	3.081
H(41)	H(6)[3]	3.495	H(41)	H(12)[9]	3.018
H(41)	H(18)[9]	3.414	H(42)	C(33)[3]	3.343
H(42)	C(34)[9]	3.366	H(42)	C(41)[9]	3.464
H(42)	C(52)[9]	3.403	H(42)	C(60)[9]	3.537
H(42)	H(6)[3]	2.832	H(42)	H(12)[9]	3.599
H(42)	H(18)[9]	2.781	H(42)	H(25)[9]	2.555
H(43)	H(6)[3]	3.033	H(44)	C(48)[12]	3.552
H(44)	H(23)[12]	3.501	H(44)	H(27)[12]	2.613
H(44)	H(31)	3.487	H(44)	H(32)[1]	3.496
H(45)	O(2)[1]	2.754	H(45)	C(36)[1]	3.226
H(45)	C(51)[1]	3.487	H(45)	C(54)[1]	3.357
H(45)	C(67)[1]	3.245	H(45)	H(32)[1]	2.723
H(45)	H(34)[1]	3.468	H(45)	H(60)[1]	3.429
H(45)	H(61)[1]	2.527	H(46)	H(27)[12]	3.396
H(46)	H(32)[1]	3.260	H(46)	H(50)[2]	3.517
H(47)	H(23)[12]	2.860	H(47)	H(54)[1]	3.523
H(47)	H(59)[13]	3.584	H(47)	H(60)[13]	3.196

Table 6. Distances beyond the asymmetric unit out to 3.60 Å (continued)

atom	atom	distance	atom	atom	distance
H(47)	H(65)[1]	3.313	H(47)	H(66)	3.539
H(47)	H(67)	2.906	H(48)	C(67)[13]	2.833
H(48)	H(23)[12]	3.573	H(48)	H(53)[3]	2.968
H(48)	H(54)[1]	3.551	H(48)	H(59)[13]	3.057
H(48)	H(60)[13]	2.325	H(48)	H(61)[13]	2.689
H(49)	H(53)[3]	3.339	H(49)	H(54)[1]	3.127
H(49)	H(67)	3.354	H(50)	C(64)[3]	3.069
H(50)	H(2)[3]	2.899	H(50)	H(46)[3]	3.517
H(50)	H(53)[3]	2.234	H(50)	H(55)[3]	3.161
H(51)	O(3)[3]	3.496	H(51)	C(48)[3]	3.079
H(51)	H(2)[3]	3.061	H(51)	H(26)[3]	2.285
H(51)	H(28)[3]	3.180	H(51)	H(40)[3]	3.166
H(51)	H(53)[3]	3.412	H(52)	O(8)[3]	2.593
H(52)	C(18)[3]	3.216	H(52)	C(40)[3]	3.373
H(52)	H(2)[3]	2.525	H(52)	H(4)	3.507
H(52)	H(26)[3]	3.349	H(52)	H(36)[3]	3.518
H(52)	H(40)[3]	3.019	H(53)	C(62)[2]	3.514
H(53)	C(63)[2]	3.150	H(53)	H(48)[2]	2.968
H(53)	H(49)[2]	3.339	H(53)	H(50)[2]	2.234
H(53)	H(51)[2]	3.412	H(53)	H(61)[1]	3.025
H(54)	O(2)[1]	3.289	H(54)	C(62)[1]	3.588
H(54)	C(67)[2]	3.411	H(54)	H(47)[1]	3.523
H(54)	H(48)[1]	3.551	H(54)	H(49)[1]	3.127
H(54)	H(59)[2]	2.755	H(54)	H(61)[2]	3.248
H(54)	H(61)[1]	3.156	H(55)	C(67)[2]	3.591
H(55)	H(1)	3.358	H(55)	H(50)[2]	3.161
H(55)	H(59)[2]	2.736	H(56)	O(9)[6]	2.993
H(56)	O(10)[6]	3.240	H(56)	C(30)[6]	2.811
H(56)	C(44)[6]	3.574	H(56)	C(47)[6]	3.020
H(56)	C(65)[6]	3.164	H(56)	H(5)[6]	2.693
H(56)	H(8)[6]	3.321	H(56)	H(56)[6]	2.561
H(56)	H(58)[6]	3.309	H(56)	H(62)[5]	3.441
H(56)	H(62)[9]	3.017	H(57)	O(9)[6]	2.832
H(57)	C(22)[6]	3.337	H(57)	C(39)[6]	2.676
H(57)	C(43)[6]	3.481	H(57)	C(50)[6]	3.441
H(57)	C(60)[7]	3.543	H(57)	H(8)[6]	2.099
H(57)	H(9)[6]	3.557	H(57)	H(12)[7]	2.899

Table 6. Distances beyond the asymmetric unit out to 3.60 Å (continued)

atom	atom	distance	atom	atom	distance
H(57)	H(17)[7)]	3.098	H(58)	O(9)[6)]	3.001
H(58)	C(69)[9)]	3.354	H(58)	H(8)[6)]	3.294
H(58)	H(17)[7)]	3.072	H(58)	H(18)[7)]	3.600
H(58)	H(38)[7)]	3.293	H(58)	H(56)[6)]	3.309
H(58)	H(62)[9)]	2.425	H(59)	C(64)[3)]	3.166
H(59)	H(25)[9)]	3.302	H(59)	H(47)[13)]	3.584
H(59)	H(48)[13)]	3.057	H(59)	H(54)[3)]	2.755
H(59)	H(55)[3)]	2.736	H(60)	C(41)[9)]	3.591
H(60)	C(62)[13)]	3.145	H(60)	H(23)[9)]	3.282
H(60)	H(25)[9)]	3.006	H(60)	H(45)[1)]	3.429
H(60)	H(47)[13)]	3.196	H(60)	H(48)[13)]	2.325
H(61)	C(61)[1)]	3.461	H(61)	C(62)[13)]	3.513
H(61)	C(64)[1)]	3.488	H(61)	H(45)[1)]	2.527
H(61)	H(48)[13)]	2.689	H(61)	H(53)[1)]	3.025
H(61)	H(54)[3)]	3.248	H(61)	H(54)[1)]	3.156
H(62)	O(9)[4)]	3.134	H(62)	O(10)[4)]	3.393
H(62)	C(30)[8)]	3.193	H(62)	C(65)[8)]	3.135
H(62)	H(5)[8)]	2.246	H(62)	H(35)[3)]	3.192
H(62)	H(56)[4)]	3.441	H(62)	H(56)[8)]	3.017
H(62)	H(58)[8)]	2.425	H(62)	H(68)[5)]	2.986
H(63)	O(5)[5)]	3.390	H(63)	O(9)[4)]	3.313
H(63)	O(10)[4)]	2.838	H(63)	C(33)[4)]	3.032
H(63)	C(47)[4)]	3.188	H(63)	C(71)[5)]	2.482
H(63)	H(5)[8)]	3.564	H(63)	H(6)[4)]	2.632
H(63)	H(9)[5)]	3.524	H(63)	H(68)[5)]	1.619
H(63)	H(69)[5)]	2.826	H(63)	H(70)[5)]	2.760
H(64)	O(9)[4)]	3.380	H(64)	C(55)[3)]	3.561
H(64)	C(71)[5)]	2.577	H(64)	H(9)[5)]	3.072
H(64)	H(35)[3)]	2.664	H(64)	H(68)[5)]	2.159
H(64)	H(69)[5)]	2.874	H(64)	H(70)[5)]	2.323
H(65)	C(70)[1)]	3.549	H(65)	H(1)	3.413
H(65)	H(13)	3.072	H(65)	H(47)[1)]	3.313
H(65)	H(66)[1)]	2.911	H(65)	H(67)[1)]	3.507
H(66)	O(2)[1)]	3.328	H(66)	C(70)[1)]	3.092
H(66)	H(13)[1)]	3.433	H(66)	H(47)	3.539
H(66)	H(65)[1)]	2.911	H(66)	H(66)[1)]	2.802
H(66)	H(67)[1)]	3.044	H(67)	N(13)	3.161

Table 6. Distances beyond the asymmetric unit out to 3.60 Å (continued)

atom	atom	distance	atom	atom	distance
H(67)	C(2)	3.427	H(67)	C(62)	3.452
H(67)	H(13)	3.035	H(67)	H(15)	3.470
H(67)	H(22)	3.183	H(67)	H(31)	3.457
H(67)	H(47)	2.906	H(67)	H(49)	3.354
H(67)	H(65)[1]	3.507	H(67)	H(66)[1]	3.044
H(68)	O(9)[3]	3.591	H(68)	O(11)[4]	3.130
H(68)	C(33)[3]	3.031	H(68)	C(69)[4]	2.247
H(68)	H(6)[3]	2.387	H(68)	H(62)[4]	2.986
H(68)	H(63)[4]	1.619	H(68)	H(64)[4]	2.159
H(69)	O(9)[3]	3.129	H(69)	C(24)[3]	3.201
H(69)	C(33)[3]	3.546	H(69)	C(38)[3]	3.196
H(69)	C(49)[3]	2.935	H(69)	C(50)[3]	3.355
H(69)	C(69)[4]	3.284	H(69)	H(1)[3]	3.428
H(69)	H(6)[3]	3.157	H(69)	H(7)[3]	3.572
H(69)	H(10)[3]	3.167	H(69)	H(63)[4]	2.826
H(69)	H(64)[4]	2.874	H(69)	H(72)[3]	2.848
H(70)	O(6)[4]	3.060	H(70)	C(38)[3]	3.008
H(70)	C(49)[3]	3.235	H(70)	C(69)[4]	2.935
H(70)	H(7)[3]	2.957	H(70)	H(10)[3]	3.336
H(70)	H(37)[3]	3.444	H(70)	H(63)[4]	2.760
H(70)	H(64)[4]	2.323	H(71)	O(4)	2.494
H(71)	C(50)	3.216	H(71)	H(15)	3.168
H(71)	H(36)[3]	2.716	H(71)	H(72)	2.821
H(72)	O(1)	3.152	H(72)	O(6)	3.422
H(72)	C(24)	3.482	H(72)	C(32)	2.974
H(72)	H(69)[2]	2.848	H(72)	H(71)	2.821

Symmetry Operators:

(1) -X+1,-Y+1,-Z
(2) X,Y+1,Z
(3) X,Y-1,Z
(4) -X+2,Y+1/2-1,-Z+1/2
(5) -X+2,Y+1/2,-Z+1/2
(6) -X+2,-Y+1,-Z
(7) X,-Y+1/2+1,Z+1/2-1
(8) X,-Y+1/2,Z+1/2
(9) X,-Y+1/2,Z+1/2-1
(10) -X+1,Y+1/2,-Z+1/2
(11) X,-Y+1/2+1,Z+1/2
(12) -X+1,Y+1/2-1,-Z+1/2
(13) -X+1,-Y,-Z

Intramolecular and Intermolecular Hydrogen bonds

D	H	A	D...A	D-H	H...A	D-H...A
N(3)	H(21)	O(3)	2.810(4)	0.950(4)	1.990(4)	143.4(2)
N(13)	H(22)	O(2)	2.806(3)	0.950(3)	1.975(4)	144.9(3)

Note) 1. The symmetry operations are applied to the acceptors.
2. Estimated standard deviations (esd's) are shown in the parentheses.
They are not calculated when all atoms have an esd=0.0.
