# Supplementary material for: Rearrangement of o-(pivaloylaminomethyl)benzaldehydes: an experimental and computational study
Source: Beilstein J Org Chem. 2020 Jul 13;16:1636–48. doi: 10.3762/bjoc.16.136 (PMC7372232; doi:10.3762/bjoc.16.136)
Supplement: File 2 — Crystallographic information files for compounds 3a, 3b, 8b, 23a, and 23b. [file Beilstein_J_Org_Chem-16-1636-s002.zip › compound+8b+X-ray+structure+report.pdf]

127043

HCS0452_1A

Submitted by: Hargitai Csilla
Operator: Dancso Andras

X-ray Structure Report

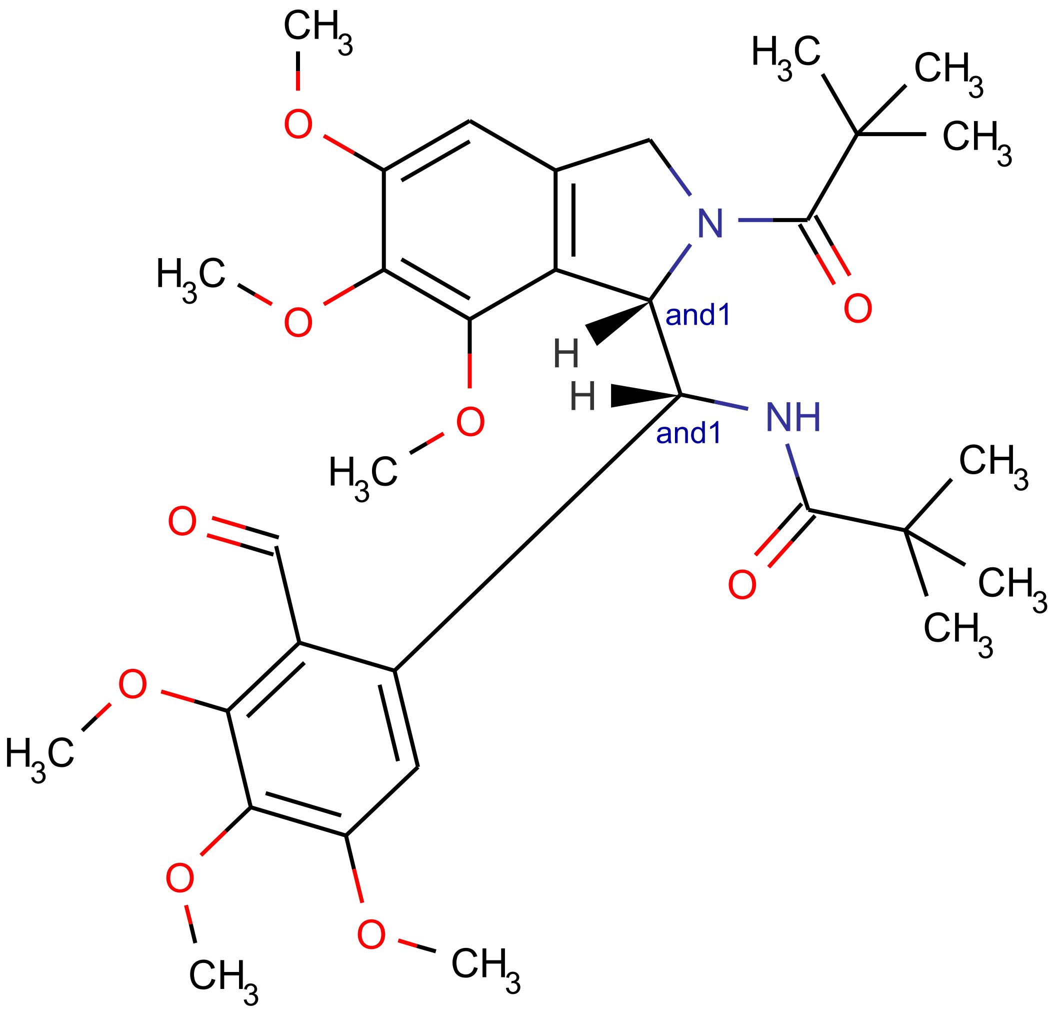

September 14, 2018

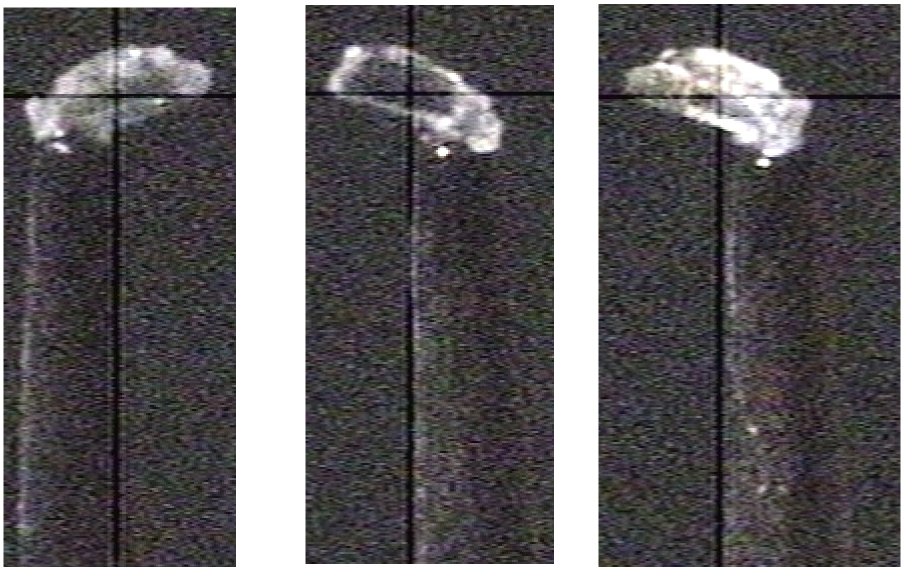

Fig. 1. The crystal

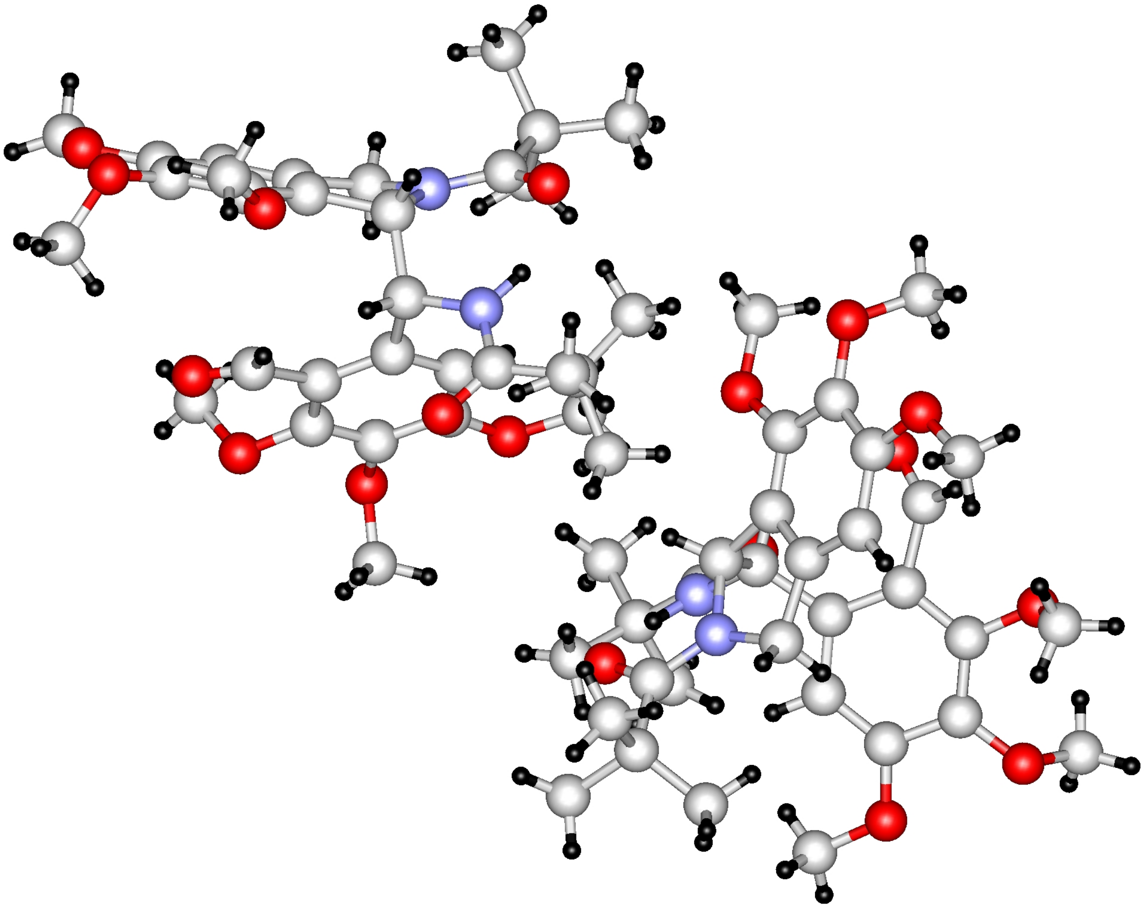

Fig. 2. Molecules in pair (hydrogens were generated by the software)

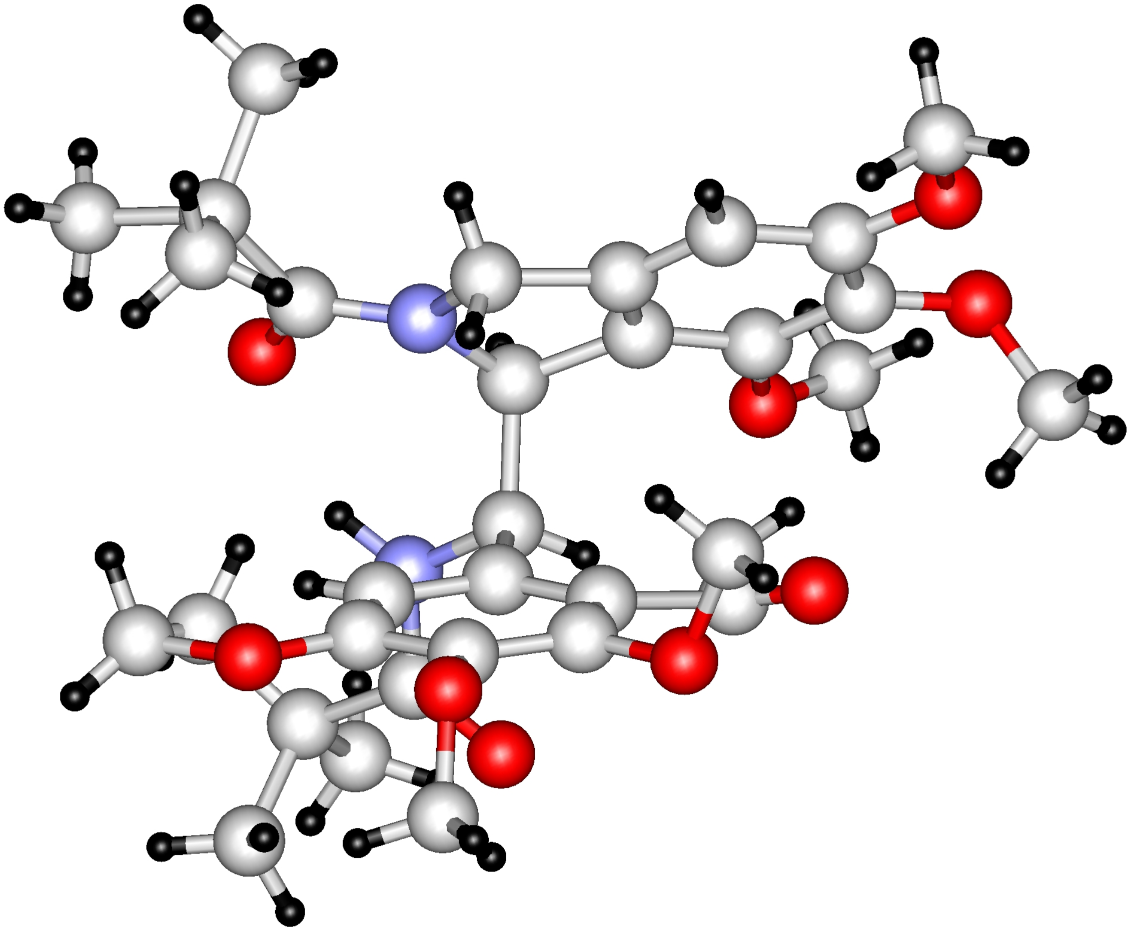

Fig. 3. Fragment 1

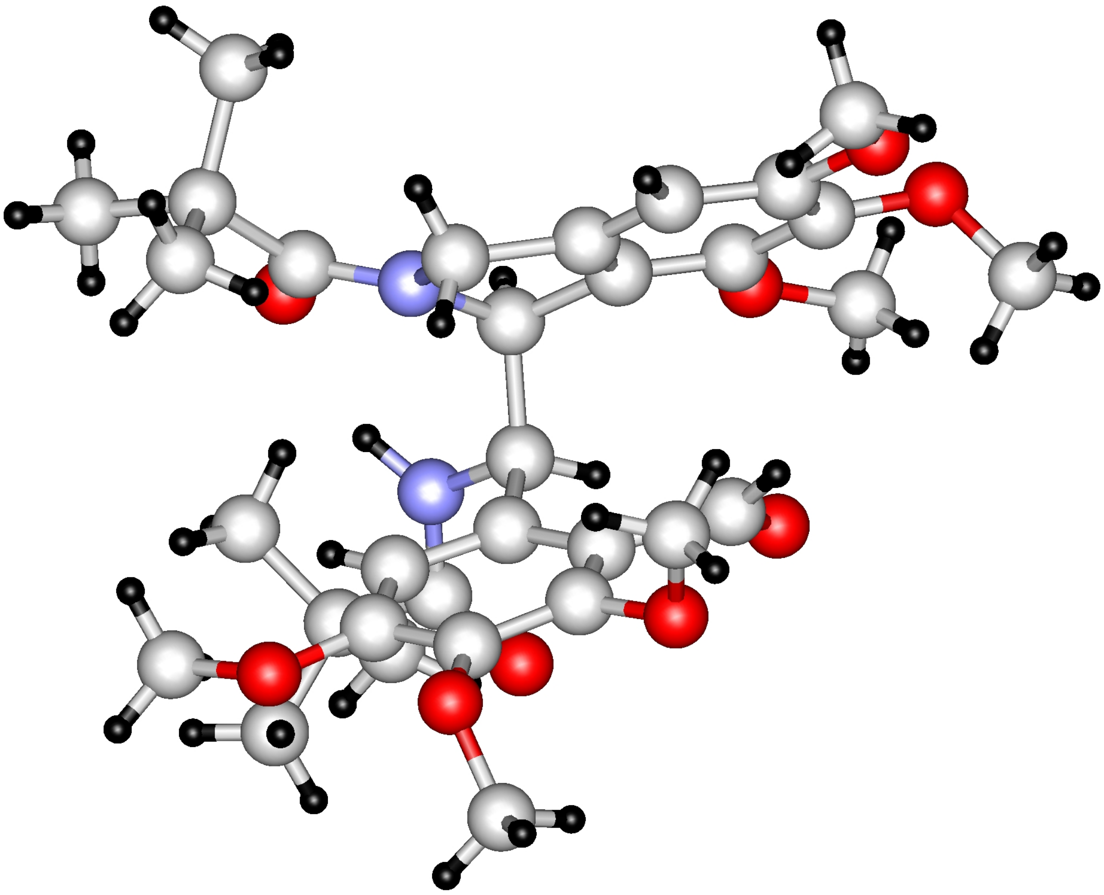

Fig. 4. Fragment 2

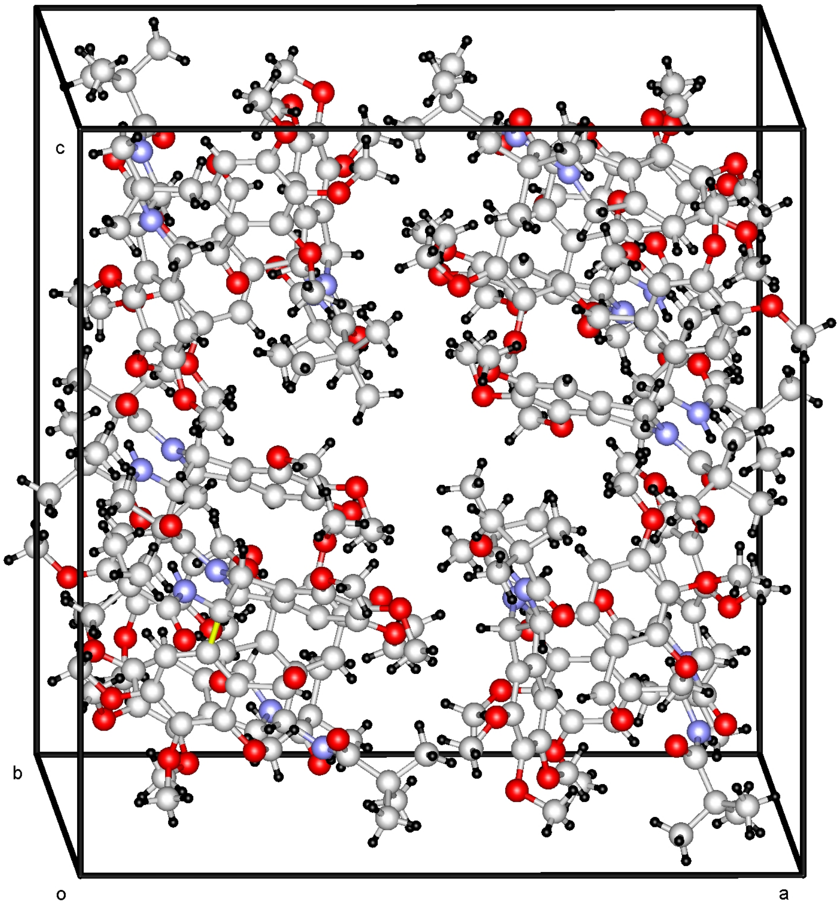

Fig. 5. Packing

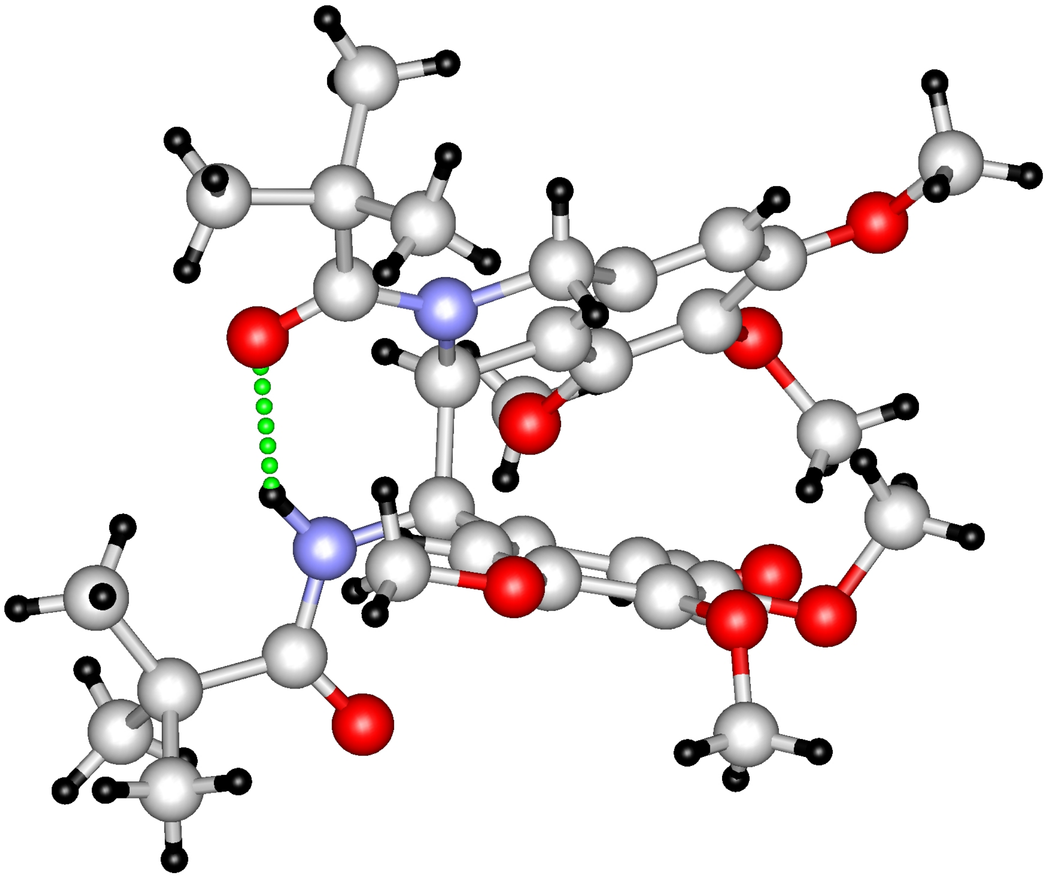

Fig. 6. Hydrogen-bond 1

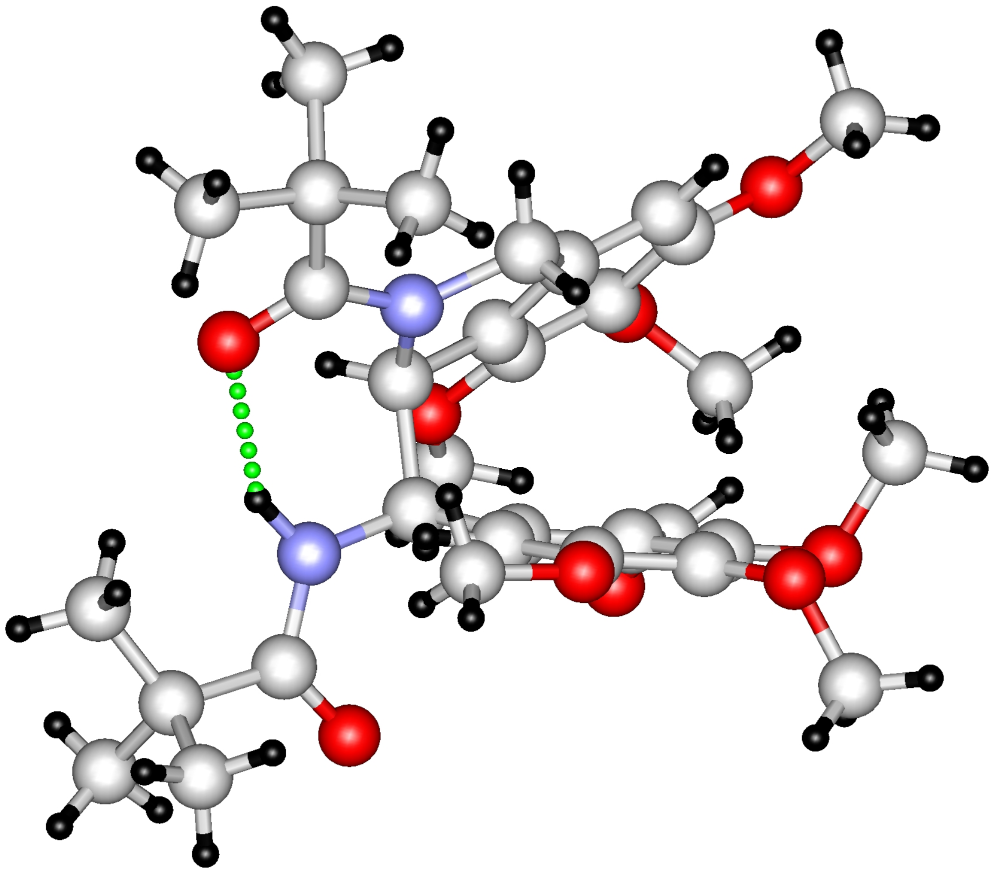

Fig. 7. Hydrogen-bond 2

Experimental

Data Collection

A colorless chunk crystal of $C_{32}H_{44}N_2O_9$ having approximate dimensions of 0.23 x 0.14 x 0.11 mm was mounted on a cactus needle. All measurements were made on a Rigaku RAXIS RAPID imaging plate area detector with graphite monochromated Cu-Kα radiation.

Indexing was performed from 4 oscillations that were exposed for 60 seconds. The crystal-to-detector distance was 127.40 mm.

Cell constants and an orientation matrix for data collection corresponded to a primitive monoclinic cell with dimensions:

a = 22.4931(9) Å

b = 12.0338(4) Å   β = 92.0900(14)^{o}

c = 24.3085(8) Å

V = 6575.4(4) Å^{3}

For Z = 8 and F.W. = 600.71, the calculated density is 1.214 g/cm^{3}. The systematic absences of:

h0l: h+l ± 2n
0k0: k ± 2n

uniquely determine the space group to be:

$$P2_1/n \text{ (\#14)}$$

The data were collected at a temperature of 20 $\pm$ 1°C to a maximum 2θ value of 143.5^{o}. A total of 180 oscillation images were collected. A sweep of data was done using ω scans from 20.0 to 200.0^{o} in 5.0^{o} step, at χ=0.0^{o} and φ = 0.0^{o}. The exposure rate was 60.0 [sec./^{o}]. A second sweep was performed using ω scans from 20.0 to 200.0^{o} in 5.0^{o} step, at χ=54.0^{o} and φ = 0.0^{o}. The exposure rate was 60.0 [sec./^{o}]. Another sweep was performed using ω scans from 20.0 to 200.0^{o} in 5.0^{o} step, at χ=54.0^{o} and φ = 90.0^{o}. The exposure rate was 60.0 [sec./^{o}]. Another sweep was performed using ω scans from 20.0 to 200.0^{o} in 5.0^{o} step, at χ=54.0^{o} and φ = 180.0^{o}. The exposure rate was 60.0 [sec./^{o}]. Another sweep was performed using ω scans from 20.0 to 200.0^{o} in 5.0^{o} step, at χ=54.0^{o} and φ = 270.0^{o}. The exposure rate was 60.0 [sec./^{o}]. The crystal-to-detector distance was 127.40 mm. Readout was performed in the 0.100 mm pixel mode.

Data Reduction

Of the 73934 reflections that were collected, 12419 were unique (R_{int} = 0.087).

The linear absorption coefficient, μ, for Cu-Kα radiation is 7.303 cm^{-1}. An empirical absorption correction was applied which resulted in transmission factors ranging from 0.806 to 0.926. The data were corrected for Lorentz and polarization effects.

Structure Solution and Refinement

The structure was solved by direct methods[1] and expanded using Fourier techniques[2]. The non-hydrogen atoms were refined anisotropically. Hydrogen atoms were refined using the riding model. The final cycle of full-matrix least-squares refinement[3] on F was based on 30790 observed reflections ($I > 2.00\sigma(I)$) and 863 variable parameters and converged (largest parameter shift was 0.00 times its esd) with unweighted and weighted agreement factors of:

$$R = \Sigma ||Fo| - |Fc|| / \Sigma |Fo| = 0.0700$$

$$R_W = [\Sigma w (|Fo| - |Fc|)^2 / \Sigma w Fo^2]^{1/2} = 0.0731$$

The standard deviation of an observation of unit weight[4] was 2.21. Unit weights were used. Plots of $\Sigma w (|Fo| - |Fc|)^2$ versus |Fo|, reflection order in data collection, sin θ/λ and various classes of indices showed no unusual trends. The maximum and minimum peaks on the final difference Fourier map corresponded to 9.14 and -21.50 $e^-/Å^3$, respectively.

Neutral atom scattering factors were taken from Cromer and Waber[5]. Anomalous dispersion effects were included in Fcalc[6]; the values for Δf' and Δf" were those of Creagh and McAuley[7]. The values for the mass attenuation coefficients are those of Creagh and Hubbell[8]. All calculations were performed using the CrystalStructure[9,10] crystallographic software package.

References

(1) SIR92: Altomare, A., Cascarano, G., Giacovazzo, C., Guagliardi, A., Burla, M., Polidori, G., and Camalli, M. (1994) J. Appl. Cryst., 27, 435.

(2) DIRDIF99: Beurskens, P.T., Admiraal, G., Beurskens, G., Bosman, W.P., de Gelder, R., Israel, R. and Smits, J.M.M.(1999). The DIRDIF-99 program system, Technical Report of the Crystallography Laboratory, University of Nijmegen, The Netherlands.

(3) Least Squares function minimized:

$$\Sigma w(|F_o|-|F_c|)^2 \quad \text{where w = Least Squares weights.}$$

(4) Standard deviation of an observation of unit weight:

$$[\Sigma w(|F_o|-|F_c|)^2/(N_o-N_v)]^{1/2}$$

where: N_o = number of observations
N_v = number of variables

(5) Cromer, D. T. & Waber, J. T.; "International Tables for X-ray Crystallography", Vol. IV, The Kynoch Press, Birmingham, England, Table 2.2 A (1974).

(6) Ibers, J. A. & Hamilton, W. C.; Acta Crystallogr., 17, 781 (1964).

(7) Creagh, D. C. & McAuley, W.J .; "International Tables for Crystallography", Vol C, (A.J.C. Wilson, ed.), Kluwer Academic Publishers, Boston, Table 4.2.6.8, pages 219-222 (1992).

(8) Creagh, D. C. & Hubbell, J.H..; "International Tables for Crystallography", Vol C, (A.J.C. Wilson, ed.), Kluwer Academic Publishers, Boston, Table 4.2.4.3, pages 200-206 (1992).

(9) CrystalStructure 3.7.0: Crystal Structure Analysis Package, Rigaku and Rigaku/MSC (2000-2005). 9009 New Trails Dr. The Woodlands TX 77381 USA.

(10) CRYSTALS Issue 10: Watkin, D.J., Prout, C.K. Carruthers, J.R. & Betteridge, P.W. Chemical Crystallography Laboratory, Oxford, UK. (1996)

EXPERIMENTAL DETAILS

A. Crystal Data

Empirical Formula	$C_{32}H_{44}N_2O_9$
Formula Weight	600.71
Crystal Color, Habit	colorless, chunk
Crystal Dimensions	0.23 X 0.14 X 0.11 mm
Crystal System	monoclinic
Lattice Type	Primitive
Indexing Images	4 oscillations @ 60.0 seconds
Detector Position	127.40 mm
Pixel Size	0.100 mm
Lattice Parameters	a = 22.4931(9) Å<br>b = 12.0338(4) Å<br>c = 24.3085(8) Å<br>β = 92.0900(14) ^{o}<br>V = 6575.4(4) $Å^3$
Space Group	$P2_1/n$ (#14)
Z value	8
D_{calc}	1.214 g/cm^3
F_{000}	2576.00
$\mu(CuK\alpha)$	7.303 cm^{-1}

B. Intensity Measurements

Diffractometer	Rigaku RAXIS-RAPID
Radiation	CuKα (λ = 1.54187 Å)<br>graphite monochromated
Detector Aperture	280 mm x 256 mm
Data Images	180 exposures
ω oscillation Range (χ=0.0, φ=0.0)	20.0 - 200.0^{o}
Exposure Rate	60.0 sec./^{o}
ω oscillation Range (χ=54.0, φ=0.0)	20.0 - 200.0^{o}
Exposure Rate	60.0 sec./^{o}
ω oscillation Range (χ=54.0, φ=90.0)	20.0 - 200.0^{o}
Exposure Rate	60.0 sec./^{o}
ω oscillation Range (χ=54.0, φ=180.0)	20.0 - 200.0^{o}
Exposure Rate	60.0 sec./^{o}
ω oscillation Range (χ=54.0, φ=270.0)	20.0 - 200.0^{o}
Exposure Rate	60.0 sec./^{o}
Detector Position	127.40 mm
Pixel Size	0.100 mm
$2\theta_{max}$	143.5^{o}
No. of Reflections Measured	Total: 73934<br>Unique: 12419 (R_{int} = 0.087)
Corrections	Lorentz-polarization<br>Absorption<br>(trans. factors: 0.806 - 0.926)

C. Structure Solution and Refinement

Structure Solution	Direct Methods (SIR92)
Refinement	Full-matrix least-squares on F
Function Minimized	Σ w (|Fo| - |Fc|)^2
Least Squares Weights	1
$2\theta_{max}$ cutoff	143.5^o
Anomalous Dispersion	All non-hydrogen atoms
No. Observations (I>2.00σ(I))	30790
No. Variables	863
Reflection/Parameter Ratio	35.68
Residuals: R (I>2.00σ(I))	0.0700
Residuals: Rw (I>2.00σ(I))	0.0731
Goodness of Fit Indicator	2.214
Max Shift/Error in Final Cycle	0.000
Maximum peak in Final Diff. Map	9.14 e^-/Å^3
Minimum peak in Final Diff. Map	-21.50 e^-/Å^3

Table 1. Atomic coordinates and B_{iso}/B_{eq}

atom	x	y	z	B_{eq}
O(1)	0.84306(12)	0.0832(2)	0.26568(13)	5.81(9)
O(2)	0.55678(12)	-0.1365(2)	0.69781(12)	6.09(9)
O(3)	0.86791(11)	-0.1156(2)	0.54994(11)	5.89(8)
O(4)	0.65666(12)	-0.1853(2)	0.37774(11)	6.84(9)
O(5)	0.71400(12)	0.1728(2)	0.70690(12)	7.96(11)
O(6)	0.74880(16)	-0.0130(3)	0.21073(18)	10.98(14)
O(7)	0.56596(12)	0.1917(2)	0.41057(12)	6.87(10)
O(8)	0.87290(13)	-0.0712(2)	0.85509(16)	7.35(11)
O(9)	0.66094(12)	0.2271(2)	0.09396(12)	6.16(9)
O(11)	0.60605(12)	0.0364(2)	0.10784(12)	6.23(10)
O(12)	0.56030(12)	0.0440(2)	0.63571(12)	6.12(9)
O(13)	0.87122(11)	0.2558(2)	0.64279(12)	6.29(9)
O(14)	0.67203(11)	0.1010(2)	0.58566(11)	5.67(8)
O(15)	0.80330(12)	0.1914(2)	0.44569(14)	6.61(10)
O(16)	0.76000(12)	0.0139(2)	0.83075(11)	5.05(8)
O(18)	0.87697(13)	0.1810(2)	0.36260(12)	7.08(10)
O(19)	0.57379(13)	-0.0300(2)	0.21685(12)	7.15(10)
O(20)	0.95114(16)	-0.0882(2)	0.77685(12)	7.08(10)
N(1)	0.61546(12)	-0.0184(2)	0.38601(12)	4.17(9)
N(2)	0.61452(13)	0.2306(2)	0.33428(16)	4.35(10)
N(3)	0.86095(12)	0.0883(2)	0.60453(12)	4.08(9)
N(4)	0.80238(12)	-0.1482(2)	0.61548(12)	3.67(9)
C(1)	0.72403(18)	0.1041(2)	0.39070(18)	4.23(12)
C(2)	0.80882(14)	0.0600(2)	0.63555(17)	3.60(11)
C(3)	0.88919(19)	0.1865(3)	0.61105(18)	4.11(13)
C(4)	0.61035(19)	-0.1144(3)	0.67585(17)	4.27(13)
C(5)	0.7217(2)	-0.0280(5)	0.2481(2)	10.8(2)
C(25)	0.8014(2)	0.0029(3)	0.79046(19)	4.17(14)
C(27)	0.7444(2)	0.0472(3)	0.2991(2)	4.30(13)
C(28)	0.70524(19)	0.0554(2)	0.34151(19)	3.86(12)
C(29)	0.61349(14)	0.1379(3)	0.24984(19)	3.44(12)
C(30)	0.84618(17)	-0.1816(3)	0.58205(18)	3.93(12)
C(31)	0.77570(16)	-0.0369(2)	0.60593(14)	3.56(11)
C(32)	0.63926(17)	0.0219(2)	0.33467(17)	4.13(12)
C(33)	0.82556(18)	0.0334(2)	0.69543(19)	3.43(12)
C(34)	0.62635(19)	-0.1216(3)	0.4033(2)	4.74(14)
C(35)	0.71347(18)	-0.0541(3)	0.62714(14)	3.58(11)

Table 1. Atomic coordinates and B_{iso}/B_{eq} (continued)

atom	x	y	z	B_{eq}
C(36)	0.8206(2)	0.1403(3)	0.3563(2)	4.82(15)
C(37)	0.6640(2)	0.0122(3)	0.61945(16)	4.00(12)
C(38)	0.59116(19)	0.2597(4)	0.3824(2)	4.91(15)
C(39)	0.78548(19)	0.0418(3)	0.7381(2)	4.03(13)
C(40)	0.64735(17)	0.2043(3)	0.1466(2)	4.27(14)
C(41)	0.61136(19)	-0.0167(3)	0.64438(18)	4.50(13)
C(42)	0.7810(2)	0.1449(3)	0.3988(2)	4.79(15)
C(43)	0.6006(2)	-0.1555(3)	0.4585(2)	5.49(15)
C(44)	0.94384(19)	0.2058(3)	0.5771(2)	4.93(13)
C(45)	0.66012(17)	0.2711(3)	0.1923(2)	4.76(13)
C(46)	0.8808(2)	0.0134(4)	0.89494(19)	10.1(2)
C(47)	0.5939(2)	0.3837(3)	0.4012(2)	5.61(15)
C(48)	0.71124(18)	-0.1500(3)	0.65691(16)	3.72(12)
C(49)	0.66011(18)	-0.1824(2)	0.68346(14)	4.18(11)
C(50)	0.8976(2)	-0.0469(3)	0.7603(2)	5.24(15)
C(51)	0.8030(2)	0.0920(3)	0.3071(2)	4.75(14)
C(52)	0.64229(17)	0.2368(3)	0.2427(2)	3.95(13)
C(53)	0.76902(14)	-0.2110(2)	0.65714(14)	4.12(11)
C(54)	0.88046(18)	-0.0133(2)	0.70799(18)	4.01(12)
C(55)	0.8578(2)	-0.0383(3)	0.8030(2)	4.58(14)
C(56)	0.81796(18)	-0.3758(2)	0.55399(14)	6.96(14)
C(57)	0.7231(2)	0.0914(3)	0.73316(18)	6.27(16)
C(58)	0.61883(19)	0.1010(3)	0.1530(2)	4.62(14)
C(60)	0.65051(17)	0.2956(2)	0.29686(17)	4.83(12)
C(61)	0.69213(18)	0.3273(3)	0.08376(16)	8.18(16)
C(62)	0.55586(18)	0.4545(3)	0.36067(17)	7.82(15)
C(63)	0.8667(2)	-0.3047(3)	0.5828(2)	4.99(14)
C(64)	0.60094(18)	0.0684(3)	0.2051(2)	4.45(14)
C(65)	0.60167(16)	0.1198(3)	0.31017(17)	4.00(12)
C(66)	0.55132(16)	-0.2301(3)	0.73161(17)	8.03(16)
C(67)	0.76693(19)	0.2007(3)	0.49084(18)	7.87(17)
C(68)	0.73221(18)	-0.0887(3)	0.84478(17)	8.28(16)
C(69)	0.84195(17)	0.1745(3)	0.22842(17)	8.20(16)
C(70)	0.63411(18)	0.1923(3)	0.58163(17)	8.62(16)
C(71)	0.88301(16)	-0.3470(2)	0.64021(18)	7.01(15)
C(72)	0.5655(2)	0.3896(3)	0.45714(19)	10.03(18)
C(74)	0.92246(17)	-0.3111(2)	0.54836(17)	8.55(16)

Table 1. Atomic coordinates and B_{iso}/B_{eq} (continued)

atom	x	y	z	B_{eq}
C(75)	0.5627(2)	-0.2559(4)	0.4474(2)	16.6(2)
C(76)	0.6572(2)	0.4292(3)	0.40701(17)	8.53(17)
C(77)	0.6542(2)	-0.0314(3)	0.09073(18)	10.12(19)
C(78)	0.91855(18)	0.1074(4)	0.38359(18)	9.89(18)
C(79)	0.99466(19)	-0.0950(3)	0.73692(19)	8.93(18)
C(80)	0.54253(18)	0.1066(3)	0.68209(19)	8.63(17)
C(81)	0.5652(2)	-0.0684(3)	0.48597(19)	13.0(2)
C(82)	0.5357(2)	-0.0785(4)	0.1828(2)	12.4(2)
C(83)	0.6504(2)	-0.1910(4)	0.49608(19)	13.7(2)
C(84)	0.95879(19)	0.1160(4)	0.5382(2)	13.3(2)
C(85)	0.99596(19)	0.2175(4)	0.6164(2)	14.9(2)
C(86)	0.9361(2)	0.3107(4)	0.5464(2)	16.5(2)
H(1)	0.5929	0.0328	0.4065	5.06
H(2)	0.6972	0.1071	0.4200	5.10
H(3)	0.6806	0.3394	0.1879	5.71
H(4)	0.6372	-0.0384	0.3094	4.95
H(5)	0.5606	0.1047	0.3141	4.78
H(6)	0.6367	0.3701	0.2946	5.80
H(7)	0.6911	0.2951	0.3093	5.80
H(8)	0.6643	0.3855	0.0770	9.90
H(9)	0.7165	0.3195	0.0529	9.90
H(10)	0.7163	0.3445	0.1155	9.89
H(11)	0.5793	0.4797	0.3314	9.42
H(12)	0.5385	0.5166	0.3782	9.40
H(13)	0.5253	0.4069	0.3464	9.40
H(14)	0.7462	0.2695	0.4895	9.42
H(15)	0.7897	0.1957	0.5244	9.41
H(16)	0.7392	0.1413	0.4886	9.42
H(17)	0.8142	0.1597	0.1989	9.92
H(18)	0.8803	0.1859	0.2143	9.92
H(19)	0.8300	0.2393	0.2475	9.93
H(20)	0.5244	0.4044	0.4509	12.14
H(21)	0.5826	0.4454	0.4804	12.15
H(22)	0.5704	0.3190	0.4743	12.15
H(23)	0.6734	0.4128	0.4427	10.17
H(24)	0.6590	0.5071	0.4008	10.18
H(25)	0.6795	0.3917	0.3802	10.18

Table 1. Atomic coordinates and B_{iso}/B_{eq} (continued)

atom	x	y	z	B_{eq}
H(26)	0.6773	0.0088	0.0655	12.24
H(27)	0.6390	-0.0968	0.0735	12.23
H(28)	0.6783	-0.0511	0.1221	12.24
H(29)	0.9191	0.1122	0.4226	11.86
H(30)	0.9571	0.1229	0.3709	11.85
H(31)	0.9069	0.0347	0.3725	11.86
H(32)	0.4969	-0.0526	0.1903	14.84
H(33)	0.5373	-0.1568	0.1876	14.84
H(34)	0.5447	-0.0604	0.1460	14.84
H(35)	0.6889	-0.0775	0.2494	13.12
H(36)	0.8747	0.0344	0.5794	4.95
H(37)	0.6591	-0.2481	0.7051	5.02
H(38)	0.9070	-0.0216	0.6788	4.81
H(39)	0.6903	0.0556	0.7492	7.42
H(40)	0.7839	0.1237	0.6347	4.33
H(41)	0.7730	-0.0240	0.5674	4.27
H(42)	0.7644	-0.2865	0.6465	4.95
H(43)	0.7889	-0.2072	0.6922	4.94
H(44)	0.9209	0.0380	0.8958	12.13
H(45)	0.8710	-0.0121	0.9305	12.12
H(46)	0.8553	0.0732	0.8843	12.13
H(47)	0.7906	-0.4013	0.5800	8.38
H(48)	0.8345	-0.4377	0.5357	8.38
H(49)	0.7979	-0.3292	0.5278	8.37
H(50)	0.5391	-0.2911	0.7091	9.74
H(51)	0.5228	-0.2177	0.7588	9.72
H(52)	0.5888	-0.2460	0.7490	9.73
H(53)	0.6982	-0.1008	0.8212	10.09
H(54)	0.7206	-0.0871	0.8820	10.09
H(55)	0.7600	-0.1472	0.8401	10.09
H(56)	0.6046	0.1787	0.5535	10.41
H(57)	0.6554	0.2580	0.5733	10.40
H(58)	0.6156	0.2012	0.6159	10.42
H(59)	0.9236	-0.3308	0.6487	8.45
H(60)	0.8766	-0.4248	0.6433	8.44
H(61)	0.8587	-0.3088	0.6652	8.45
H(62)	0.9103	-0.3257	0.5112	10.43

Table 1. Atomic coordinates and B_{iso}/B_{eq} (continued)

atom	x	y	z	B_{eq}
H(63)	0.9489	-0.3678	0.5611	10.43
H(64)	0.9420	-0.2413	0.5505	10.46
H(65)	0.9917	-0.1640	0.7180	10.70
H(66)	1.0332	-0.0880	0.7538	10.71
H(67)	0.9881	-0.0359	0.7115	10.72
H(68)	0.5169	0.0624	0.7034	10.48
H(69)	0.5224	0.1725	0.6707	10.47
H(70)	0.5771	0.1255	0.7037	10.48
H(71)	0.9848	0.0643	0.5563	16.24
H(72)	0.9774	0.1452	0.5069	16.22
H(73)	0.9229	0.0796	0.5270	16.23
H(74)	0.9995	0.2936	0.6264	17.87
H(75)	1.0319	0.1932	0.6009	17.86
H(76)	0.9885	0.1745	0.6482	17.86
H(77)	0.9166	0.2994	0.5115	20.18
H(78)	0.9737	0.3444	0.5414	20.18
H(79)	0.9126	0.3577	0.5682	20.19
H(80)	0.5231	-0.2338	0.4379	20.12
H(81)	0.5625	-0.3060	0.4775	20.11
H(82)	0.5796	-0.2914	0.4167	20.12
H(83)	0.5907	-0.0227	0.5083	15.91
H(84)	0.5356	-0.1005	0.5080	15.89
H(85)	0.5466	-0.0246	0.4577	15.90
H(86)	0.6579	-0.2682	0.4917	16.47
H(87)	0.6427	-0.1760	0.5335	16.46
H(88)	0.6842	-0.1498	0.4856	16.46

$B_{eq} = 8/3\ \pi^2(U_{11}(aa^*)^2 + U_{22}(bb^*)^2 + U_{33}(cc^*)^2 + 2U_{12}(aa^*bb^*)\cos\gamma + 2U_{13}(aa^*cc^*)\cos\beta + 2U_{23}(bb^*cc^*)\cos\alpha)$

Table 2. Anisotropic displacement parameters

atom	U_{11}	U_{22}	U_{33}	U_{12}	U_{13}	U_{23}
O(1)	0.054(2)	0.088(2)	0.080(2)	0.0159(17)	0.0164(18)	0.0204(19)
O(2)	0.053(2)	0.084(2)	0.096(2)	-0.0080(17)	0.0239(18)	0.0284(19)
O(3)	0.092(2)	0.0523(19)	0.083(2)	-0.0089(16)	0.0513(18)	0.0021(16)
O(4)	0.130(2)	0.0475(19)	0.083(2)	0.0259(18)	0.022(2)	0.0016(17)
O(5)	0.092(2)	0.099(2)	0.112(3)	0.007(2)	0.008(2)	-0.001(2)
O(6)	0.096(3)	0.159(3)	0.161(4)	0.016(2)	-0.017(2)	-0.015(3)
O(7)	0.112(2)	0.071(2)	0.081(2)	-0.0020(18)	0.044(2)	0.0004(18)
O(8)	0.120(2)	0.098(2)	0.060(3)	0.009(2)	-0.014(2)	0.019(2)
O(9)	0.104(2)	0.072(2)	0.058(2)	-0.0170(18)	0.011(2)	0.0096(19)
O(11)	0.099(2)	0.072(2)	0.065(2)	-0.0211(19)	-0.002(2)	-0.0168(19)
O(12)	0.040(2)	0.085(2)	0.108(2)	0.0102(16)	0.0069(18)	0.012(2)
O(13)	0.069(2)	0.0505(19)	0.122(2)	-0.0068(16)	0.0310(18)	-0.0218(18)
O(14)	0.058(2)	0.061(2)	0.096(2)	0.0126(16)	0.0065(17)	0.0335(18)
O(15)	0.080(2)	0.108(2)	0.062(2)	-0.0100(19)	0.003(2)	-0.018(2)
O(16)	0.084(2)	0.0507(19)	0.059(2)	-0.0073(16)	0.0215(17)	0.0032(16)
O(18)	0.046(2)	0.112(2)	0.110(2)	-0.005(2)	-0.015(2)	0.013(2)
O(19)	0.109(2)	0.073(2)	0.089(2)	-0.043(2)	-0.013(2)	-0.003(2)
O(20)	0.063(2)	0.119(2)	0.087(3)	0.012(2)	-0.007(2)	0.006(2)
N(1)	0.055(2)	0.045(2)	0.060(2)	0.0108(17)	0.0215(19)	-0.011(2)
N(2)	0.058(2)	0.044(2)	0.063(3)	-0.0034(18)	0.008(2)	0.009(2)
N(3)	0.061(2)	0.040(2)	0.056(2)	0.0013(18)	0.0256(19)	-0.0022(18)
N(4)	0.039(2)	0.037(2)	0.064(2)	0.0040(17)	0.0054(18)	0.0071(19)
C(1)	0.052(3)	0.051(2)	0.059(3)	-0.007(2)	0.012(2)	-0.002(2)
C(2)	0.036(2)	0.045(2)	0.056(3)	0.005(2)	0.011(2)	-0.003(2)
C(3)	0.064(3)	0.030(2)	0.063(3)	0.009(2)	0.011(2)	0.008(2)
C(4)	0.048(3)	0.058(3)	0.057(3)	-0.014(2)	0.006(2)	0.011(2)
C(5)	0.099(5)	0.231(7)	0.086(5)	0.125(5)	0.088(4)	0.099(4)
C(25)	0.079(4)	0.045(2)	0.036(3)	-0.025(2)	0.013(3)	0.003(2)
C(27)	0.072(3)	0.056(3)	0.036(3)	0.019(2)	0.019(2)	0.005(2)
C(28)	0.054(3)	0.041(2)	0.052(3)	0.004(2)	0.003(2)	-0.005(2)
C(29)	0.032(2)	0.049(3)	0.050(3)	0.000(2)	0.010(2)	0.011(2)
C(30)	0.030(2)	0.052(3)	0.068(3)	-0.008(2)	0.009(2)	-0.009(2)
C(31)	0.046(2)	0.050(2)	0.040(3)	0.002(2)	0.006(2)	0.003(2)
C(32)	0.048(2)	0.050(2)	0.060(3)	-0.000(2)	0.006(2)	-0.005(2)
C(33)	0.034(2)	0.043(2)	0.054(3)	-0.003(2)	0.002(2)	0.003(2)
C(34)	0.075(3)	0.012(2)	0.092(4)	0.012(2)	-0.015(3)	-0.007(2)
C(35)	0.055(3)	0.028(2)	0.052(3)	-0.004(2)	-0.004(2)	0.005(2)

Table 2. Anisotropic displacement parameters (continued)

atom	U_{11}	U_{22}	U_{33}	U_{12}	U_{13}	U_{23}
C(36)	0.038(3)	0.073(3)	0.071(4)	-0.005(2)	-0.003(3)	-0.001(3)
C(37)	0.069(3)	0.033(2)	0.050(3)	0.012(2)	-0.001(2)	0.011(2)
C(38)	0.055(3)	0.085(4)	0.048(4)	0.011(2)	0.015(2)	0.010(3)
C(39)	0.040(3)	0.043(2)	0.071(4)	-0.003(2)	0.007(2)	0.002(2)
C(40)	0.048(3)	0.054(3)	0.060(4)	0.009(2)	-0.000(2)	0.009(3)
C(41)	0.033(2)	0.074(3)	0.065(3)	-0.000(2)	0.006(2)	0.002(2)
C(42)	0.060(3)	0.063(3)	0.058(4)	-0.008(2)	-0.003(3)	0.005(2)
C(43)	0.098(4)	0.062(3)	0.051(3)	-0.004(3)	0.034(3)	0.025(2)
C(44)	0.045(3)	0.067(3)	0.077(4)	-0.010(2)	0.033(2)	-0.000(2)
C(45)	0.066(3)	0.056(3)	0.059(3)	-0.011(2)	0.001(2)	0.002(3)
C(46)	0.155(5)	0.171(5)	0.057(4)	-0.045(4)	-0.021(3)	0.002(4)
C(47)	0.106(4)	0.047(3)	0.060(4)	0.021(3)	0.005(3)	-0.010(2)
C(48)	0.044(2)	0.041(2)	0.056(3)	0.006(2)	-0.001(2)	-0.008(2)
C(49)	0.042(2)	0.054(2)	0.064(3)	0.011(2)	0.008(2)	0.018(2)
C(50)	0.044(3)	0.084(3)	0.070(4)	-0.009(2)	-0.002(3)	0.007(3)
C(51)	0.044(3)	0.061(3)	0.076(4)	0.002(2)	0.006(3)	0.019(3)
C(52)	0.052(2)	0.054(3)	0.044(3)	-0.004(2)	0.005(2)	0.007(2)
C(53)	0.051(2)	0.043(2)	0.064(3)	-0.001(2)	0.014(2)	0.012(2)
C(54)	0.051(3)	0.054(2)	0.047(3)	0.002(2)	0.006(2)	0.003(2)
C(55)	0.054(3)	0.060(3)	0.059(4)	0.001(2)	-0.013(3)	0.005(2)
C(56)	0.110(4)	0.063(3)	0.092(4)	-0.001(2)	0.012(3)	-0.012(2)
C(57)	0.137(5)	0.043(3)	0.055(3)	-0.026(3)	-0.038(3)	0.012(2)
C(58)	0.068(3)	0.044(3)	0.063(4)	0.004(2)	-0.008(3)	-0.012(3)
C(60)	0.072(3)	0.053(2)	0.059(3)	-0.008(2)	0.007(2)	-0.008(2)
C(61)	0.127(4)	0.108(4)	0.078(4)	-0.028(3)	0.030(3)	0.014(3)
C(62)	0.125(4)	0.068(3)	0.104(4)	0.028(3)	0.008(3)	-0.012(3)
C(63)	0.061(3)	0.054(3)	0.077(4)	0.010(2)	0.026(3)	0.007(2)
C(64)	0.047(3)	0.047(3)	0.074(4)	-0.008(2)	-0.015(2)	-0.007(3)
C(65)	0.044(2)	0.041(2)	0.067(3)	-0.002(2)	-0.000(2)	0.000(2)
C(66)	0.061(3)	0.131(4)	0.115(4)	-0.011(3)	0.035(2)	0.037(3)
C(67)	0.109(4)	0.125(4)	0.063(4)	0.006(3)	-0.016(3)	-0.011(3)
C(68)	0.131(4)	0.071(3)	0.117(4)	-0.014(3)	0.067(3)	0.001(3)
C(69)	0.098(4)	0.126(4)	0.090(4)	0.030(3)	0.029(3)	0.053(3)
C(70)	0.101(4)	0.069(3)	0.159(5)	0.017(3)	0.029(3)	0.055(3)
C(71)	0.064(3)	0.078(3)	0.125(4)	0.024(2)	0.008(3)	0.017(3)
C(72)	0.197(5)	0.100(4)	0.087(4)	0.032(3)	0.044(4)	-0.022(3)
C(74)	0.102(4)	0.082(3)	0.146(4)	0.020(2)	0.072(3)	0.006(3)

Table 2. Anisotropic displacement parameters (continued)

atom	U_{11}	U_{22}	U_{33}	U_{12}	U_{13}	U_{23}
C(75)	0.301(8)	0.191(6)	0.145(5)	-0.158(6)	0.082(5)	-0.008(4)
C(76)	0.119(4)	0.088(3)	0.114(4)	-0.007(3)	-0.032(3)	-0.024(3)
C(77)	0.181(5)	0.099(4)	0.106(4)	0.008(4)	0.039(4)	-0.040(3)
C(78)	0.050(3)	0.223(5)	0.102(4)	0.012(3)	-0.011(3)	0.002(4)
C(79)	0.060(3)	0.152(4)	0.127(5)	0.047(3)	-0.007(3)	-0.015(3)
C(80)	0.099(4)	0.107(4)	0.125(5)	0.038(3)	0.047(3)	0.002(3)
C(81)	0.245(6)	0.139(5)	0.119(5)	0.078(4)	0.124(4)	0.033(3)
C(82)	0.172(5)	0.174(5)	0.124(5)	-0.130(4)	-0.004(4)	0.007(4)
C(83)	0.174(5)	0.272(7)	0.074(4)	0.069(5)	0.011(4)	0.085(4)
C(84)	0.135(4)	0.173(5)	0.205(6)	-0.065(4)	0.135(4)	-0.086(5)
C(85)	0.040(3)	0.403(9)	0.123(5)	-0.058(4)	0.012(3)	-0.042(5)
C(86)	0.160(5)	0.159(5)	0.321(8)	0.047(4)	0.152(5)	0.147(6)

The general temperature factor expression: $\exp(-2\pi^2(a^{*2}U_{11}h^2 + b^{*2}U_{22}k^2 + c^{*2}U_{33}l^2 + 2a^*b^*U_{12}hk + 2a^*c^*U_{13}hl + 2b^*c^*U_{23}kl))$

Table 3. Bond lengths (Å)

atom	atom	distance	atom	atom	distance
O(1)	C(51)	1.379(6)	O(1)	C(69)	1.423(5)
O(2)	C(4)	1.362(5)	O(2)	C(66)	1.402(5)
O(3)	C(30)	1.227(4)	O(4)	C(34)	1.213(5)
O(5)	C(57)	1.183(5)	O(6)	C(5)	1.126(7)
O(7)	C(38)	1.221(5)	O(8)	C(46)	1.412(5)
O(8)	C(55)	1.358(6)	O(9)	C(40)	1.354(6)
O(9)	C(61)	1.421(5)	O(11)	C(58)	1.367(6)
O(11)	C(77)	1.429(5)	O(12)	C(41)	1.371(5)
O(12)	C(80)	1.425(5)	O(13)	C(3)	1.215(5)
O(14)	C(37)	1.364(4)	O(14)	C(70)	1.391(4)
O(15)	C(42)	1.350(6)	O(15)	C(67)	1.397(5)
O(16)	C(25)	1.382(5)	O(16)	C(68)	1.431(4)
O(18)	C(36)	1.363(5)	O(18)	C(78)	1.372(5)
O(19)	C(64)	1.368(5)	O(19)	C(82)	1.307(5)
O(20)	C(50)	1.350(5)	O(20)	C(79)	1.406(5)
N(1)	C(32)	1.459(5)	N(1)	C(34)	1.332(4)
N(1)	H(1)	0.950	N(2)	C(38)	1.345(6)
N(2)	C(60)	1.465(5)	N(2)	C(65)	1.481(5)
N(3)	C(2)	1.457(4)	N(3)	C(3)	1.349(5)
N(3)	H(36)	0.950	N(4)	C(30)	1.360(5)
N(4)	C(31)	1.483(4)	N(4)	C(53)	1.488(4)
C(1)	C(28)	1.384(6)	C(1)	C(42)	1.379(6)
C(1)	H(2)	0.950	C(2)	C(31)	1.546(5)
C(2)	C(33)	1.524(6)	C(2)	H(40)	0.950
C(3)	C(44)	1.524(6)	C(4)	C(41)	1.404(6)
C(4)	C(49)	1.393(5)	C(5)	C(27)	1.604(7)
C(5)	H(35)	0.950	C(25)	C(39)	1.390(6)
C(25)	C(55)	1.387(6)	C(27)	C(28)	1.384(6)
C(27)	C(51)	1.431(6)	C(28)	C(32)	1.541(5)
C(29)	C(52)	1.369(5)	C(29)	C(64)	1.393(6)
C(29)	C(65)	1.516(6)	C(30)	C(63)	1.552(6)
C(31)	C(35)	1.523(5)	C(31)	H(41)	0.950
C(32)	C(65)	1.556(5)	C(32)	H(4)	0.950
C(33)	C(39)	1.403(6)	C(33)	C(54)	1.381(5)
C(34)	C(43)	1.534(7)	C(35)	C(37)	1.377(5)
C(35)	C(48)	1.364(5)	C(36)	C(42)	1.390(7)
C(36)	C(51)	1.374(7)	C(37)	C(41)	1.393(6)

Table 3. Bond lengths (Å) (continued)

atom	atom	distance	atom	atom	distance
C(38)	C(47)	1.561(6)	C(39)	C(57)	1.526(6)
C(40)	C(45)	1.393(6)	C(40)	C(58)	1.411(6)
C(43)	C(75)	1.497(7)	C(43)	C(81)	1.490(7)
C(43)	C(83)	1.483(7)	C(44)	C(84)	1.482(6)
C(44)	C(85)	1.492(6)	C(44)	C(86)	1.474(7)
C(45)	C(52)	1.367(6)	C(45)	H(3)	0.950
C(46)	H(44)	0.950	C(46)	H(45)	0.950
C(46)	H(46)	0.950	C(47)	C(62)	1.539(6)
C(47)	C(72)	1.524(6)	C(47)	C(76)	1.528(6)
C(48)	C(49)	1.395(5)	C(48)	C(53)	1.492(5)
C(49)	H(37)	0.950	C(50)	C(54)	1.376(6)
C(50)	C(55)	1.399(7)	C(52)	C(60)	1.499(6)
C(53)	H(42)	0.950	C(53)	H(43)	0.950
C(54)	H(38)	0.950	C(56)	C(63)	1.539(5)
C(56)	H(47)	0.950	C(56)	H(48)	0.950
C(56)	H(49)	0.950	C(57)	H(39)	0.950
C(58)	C(64)	1.398(7)	C(60)	H(6)	0.950
C(60)	H(7)	0.950	C(61)	H(8)	0.950
C(61)	H(9)	0.950	C(61)	H(10)	0.950
C(62)	H(11)	0.950	C(62)	H(12)	0.950
C(62)	H(13)	0.950	C(63)	C(71)	1.517(6)
C(63)	C(74)	1.535(6)	C(65)	H(5)	0.950
C(66)	H(50)	0.950	C(66)	H(51)	0.950
C(66)	H(52)	0.950	C(67)	H(14)	0.950
C(67)	H(15)	0.950	C(67)	H(16)	0.950
C(68)	H(53)	0.950	C(68)	H(54)	0.950
C(68)	H(55)	0.950	C(69)	H(17)	0.950
C(69)	H(18)	0.950	C(69)	H(19)	0.950
C(70)	H(56)	0.950	C(70)	H(57)	0.950
C(70)	H(58)	0.950	C(71)	H(59)	0.950
C(71)	H(60)	0.950	C(71)	H(61)	0.950
C(72)	H(20)	0.950	C(72)	H(21)	0.950
C(72)	H(22)	0.950	C(74)	H(62)	0.950
C(74)	H(63)	0.950	C(74)	H(64)	0.950
C(75)	H(80)	0.950	C(75)	H(81)	0.950
C(75)	H(82)	0.950	C(76)	H(23)	0.950
C(76)	H(24)	0.950	C(76)	H(25)	0.950

Table 3. Bond lengths (Å) (continued)

atom	atom	distance	atom	atom	distance
C(77)	H(26)	0.950	C(77)	H(27)	0.950
C(77)	H(28)	0.950	C(78)	H(29)	0.950
C(78)	H(30)	0.950	C(78)	H(31)	0.950
C(79)	H(65)	0.950	C(79)	H(66)	0.950
C(79)	H(67)	0.950	C(80)	H(68)	0.950
C(80)	H(69)	0.950	C(80)	H(70)	0.950
C(81)	H(83)	0.950	C(81)	H(84)	0.950
C(81)	H(85)	0.950	C(82)	H(32)	0.950
C(82)	H(33)	0.950	C(82)	H(34)	0.950
C(83)	H(86)	0.950	C(83)	H(87)	0.950
C(83)	H(88)	0.950	C(84)	H(71)	0.950
C(84)	H(72)	0.950	C(84)	H(73)	0.950
C(85)	H(74)	0.950	C(85)	H(75)	0.950
C(85)	H(76)	0.950	C(86)	H(77)	0.950
C(86)	H(78)	0.950	C(86)	H(79)	0.950

Table 4. Bond angles (^{o})

atom	atom	atom	angle	atom	atom	atom	angle
C(51)	O(1)	C(69)	114.1(3)	C(4)	O(2)	C(66)	119.0(3)
C(46)	O(8)	C(55)	116.8(3)	C(40)	O(9)	C(61)	117.8(3)
C(58)	O(11)	C(77)	114.8(3)	C(41)	O(12)	C(80)	114.7(3)
C(37)	O(14)	C(70)	124.5(3)	C(42)	O(15)	C(67)	119.1(3)
C(25)	O(16)	C(68)	113.4(2)	C(36)	O(18)	C(78)	115.4(3)
C(64)	O(19)	C(82)	122.8(3)	C(50)	O(20)	C(79)	116.9(3)
C(32)	N(1)	C(34)	120.8(3)	C(32)	N(1)	H(1)	116.9
C(34)	N(1)	H(1)	122.3	C(38)	N(2)	C(60)	130.0(3)
C(38)	N(2)	C(65)	120.1(3)	C(60)	N(2)	C(65)	109.8(3)
C(2)	N(3)	C(3)	121.9(3)	C(2)	N(3)	H(36)	117.3
C(3)	N(3)	H(36)	120.8	C(30)	N(4)	C(31)	118.1(3)
C(30)	N(4)	C(53)	130.6(3)	C(31)	N(4)	C(53)	110.7(2)
C(28)	C(1)	C(42)	121.8(4)	C(28)	C(1)	H(2)	118.8
C(42)	C(1)	H(2)	119.4	N(3)	C(2)	C(31)	108.6(3)
N(3)	C(2)	C(33)	111.6(2)	N(3)	C(2)	H(40)	106.5
C(31)	C(2)	C(33)	112.6(2)	C(31)	C(2)	H(40)	108.9
C(33)	C(2)	H(40)	108.4	O(13)	C(3)	N(3)	120.7(3)
O(13)	C(3)	C(44)	122.2(3)	N(3)	C(3)	C(44)	117.1(3)
O(2)	C(4)	C(41)	114.2(3)	O(2)	C(4)	C(49)	123.5(3)
C(41)	C(4)	C(49)	122.4(3)	O(6)	C(5)	C(27)	111.6(5)
O(6)	C(5)	H(35)	124.9	C(27)	C(5)	H(35)	123.5
O(16)	C(25)	C(39)	117.3(4)	O(16)	C(25)	C(55)	120.9(4)
C(39)	C(25)	C(55)	121.7(4)	C(5)	C(27)	C(28)	115.0(4)
C(5)	C(27)	C(51)	125.7(4)	C(28)	C(27)	C(51)	118.7(4)
C(1)	C(28)	C(27)	119.4(3)	C(1)	C(28)	C(32)	117.8(3)
C(27)	C(28)	C(32)	122.6(3)	C(52)	C(29)	C(64)	120.4(4)
C(52)	C(29)	C(65)	110.3(3)	C(64)	C(29)	C(65)	129.2(3)
O(3)	C(30)	N(4)	120.2(3)	O(3)	C(30)	C(63)	120.0(3)
N(4)	C(30)	C(63)	119.7(3)	N(4)	C(31)	C(2)	115.0(2)
N(4)	C(31)	C(35)	101.3(2)	N(4)	C(31)	H(41)	108.2
C(2)	C(31)	C(35)	112.2(2)	C(2)	C(31)	H(41)	110.4
C(35)	C(31)	H(41)	109.3	N(1)	C(32)	C(28)	112.1(3)
N(1)	C(32)	C(65)	111.6(3)	N(1)	C(32)	H(4)	106.7
C(28)	C(32)	C(65)	110.5(2)	C(28)	C(32)	H(4)	107.0
C(65)	C(32)	H(4)	108.5	C(2)	C(33)	C(39)	123.3(3)
C(2)	C(33)	C(54)	119.0(3)	C(39)	C(33)	C(54)	117.3(4)
O(4)	C(34)	N(1)	121.9(4)	O(4)	C(34)	C(43)	121.1(3)

Table 4. Bond angles (^{o}) (continued)

atom	atom	atom	angle	atom	atom	atom	angle
N(1)	C(34)	C(43)	116.9(3)	C(31)	C(35)	C(37)	128.6(3)
C(31)	C(35)	C(48)	110.3(3)	C(37)	C(35)	C(48)	121.1(3)
O(18)	C(36)	C(42)	121.4(4)	O(18)	C(36)	C(51)	119.1(4)
C(42)	C(36)	C(51)	119.5(4)	O(14)	C(37)	C(35)	114.3(3)
O(14)	C(37)	C(41)	126.2(3)	C(35)	C(37)	C(41)	119.5(3)
O(7)	C(38)	N(2)	121.2(4)	O(7)	C(38)	C(47)	119.3(4)
N(2)	C(38)	C(47)	119.4(4)	C(25)	C(39)	C(33)	120.1(4)
C(25)	C(39)	C(57)	114.2(4)	C(33)	C(39)	C(57)	125.7(4)
O(9)	C(40)	C(45)	126.1(3)	O(9)	C(40)	C(58)	113.7(4)
C(45)	C(40)	C(58)	120.1(4)	O(12)	C(41)	C(4)	120.0(3)
O(12)	C(41)	C(37)	121.4(3)	C(4)	C(41)	C(37)	118.5(3)
O(15)	C(42)	C(1)	125.6(4)	O(15)	C(42)	C(36)	114.6(4)
C(1)	C(42)	C(36)	119.8(4)	C(34)	C(43)	C(75)	106.7(3)
C(34)	C(43)	C(81)	115.5(3)	C(34)	C(43)	C(83)	108.4(4)
C(75)	C(43)	C(81)	109.8(4)	C(75)	C(43)	C(83)	106.9(4)
C(81)	C(43)	C(83)	109.3(4)	C(3)	C(44)	C(84)	116.1(3)
C(3)	C(44)	C(85)	107.3(3)	C(3)	C(44)	C(86)	108.8(3)
C(84)	C(44)	C(85)	106.6(3)	C(84)	C(44)	C(86)	109.1(4)
C(85)	C(44)	C(86)	108.6(4)	C(40)	C(45)	C(52)	118.9(3)
C(40)	C(45)	H(3)	119.8	C(52)	C(45)	H(3)	121.4
O(8)	C(46)	H(44)	109.5	O(8)	C(46)	H(45)	111.3
O(8)	C(46)	H(46)	107.5	H(44)	C(46)	H(45)	109.5
H(44)	C(46)	H(46)	109.5	H(45)	C(46)	H(46)	109.5
C(38)	C(47)	C(62)	109.1(3)	C(38)	C(47)	C(72)	107.0(3)
C(38)	C(47)	C(76)	113.3(3)	C(62)	C(47)	C(72)	107.7(3)
C(62)	C(47)	C(76)	110.8(3)	C(72)	C(47)	C(76)	108.6(3)
C(35)	C(48)	C(49)	121.9(3)	C(35)	C(48)	C(53)	111.6(3)
C(49)	C(48)	C(53)	126.4(3)	C(4)	C(49)	C(48)	116.5(3)
C(4)	C(49)	H(37)	121.7	C(48)	C(49)	H(37)	121.7
O(20)	C(50)	C(54)	126.9(4)	O(20)	C(50)	C(55)	113.2(4)
C(54)	C(50)	C(55)	119.9(4)	O(1)	C(51)	C(27)	119.7(4)
O(1)	C(51)	C(36)	119.6(4)	C(27)	C(51)	C(36)	120.7(4)
C(29)	C(52)	C(45)	122.1(4)	C(29)	C(52)	C(60)	110.0(3)
C(45)	C(52)	C(60)	127.9(3)	N(4)	C(53)	C(48)	102.1(2)
N(4)	C(53)	H(42)	110.7	N(4)	C(53)	H(43)	110.8
C(48)	C(53)	H(42)	112.5	C(48)	C(53)	H(43)	111.2
H(42)	C(53)	H(43)	109.5	C(33)	C(54)	C(50)	122.8(4)

Table 4. Bond angles (^{o}) (continued)

atom	atom	atom	angle	atom	atom	atom	angle
C(33)	C(54)	H(38)	117.3	C(50)	C(54)	H(38)	119.9
O(8)	C(55)	C(25)	120.3(4)	O(8)	C(55)	C(50)	121.7(4)
C(25)	C(55)	C(50)	117.9(4)	C(63)	C(56)	H(47)	110.2
C(63)	C(56)	H(48)	111.3	C(63)	C(56)	H(49)	106.9
H(47)	C(56)	H(48)	109.5	H(47)	C(56)	H(49)	109.5
H(48)	C(56)	H(49)	109.5	O(5)	C(57)	C(39)	120.5(4)
O(5)	C(57)	H(39)	118.4	C(39)	C(57)	H(39)	121.0
O(11)	C(58)	C(40)	119.7(4)	O(11)	C(58)	C(64)	120.6(3)
C(40)	C(58)	C(64)	119.6(4)	N(2)	C(60)	C(52)	103.9(3)
N(2)	C(60)	H(6)	110.6	N(2)	C(60)	H(7)	110.2
C(52)	C(60)	H(6)	111.4	C(52)	C(60)	H(7)	111.2
H(6)	C(60)	H(7)	109.5	O(9)	C(61)	H(8)	109.2
O(9)	C(61)	H(9)	110.9	O(9)	C(61)	H(10)	108.3
H(8)	C(61)	H(9)	109.5	H(8)	C(61)	H(10)	109.5
H(9)	C(61)	H(10)	109.5	C(47)	C(62)	H(11)	110.2
C(47)	C(62)	H(12)	112.1	C(47)	C(62)	H(13)	106.0
H(11)	C(62)	H(12)	109.5	H(11)	C(62)	H(13)	109.5
H(12)	C(62)	H(13)	109.5	C(30)	C(63)	C(56)	108.6(3)
C(30)	C(63)	C(71)	113.2(3)	C(30)	C(63)	C(74)	106.9(3)
C(56)	C(63)	C(71)	112.1(3)	C(56)	C(63)	C(74)	107.8(3)
C(71)	C(63)	C(74)	108.1(3)	O(19)	C(64)	C(29)	115.8(4)
O(19)	C(64)	C(58)	125.3(4)	C(29)	C(64)	C(58)	118.8(4)
N(2)	C(65)	C(29)	102.4(3)	N(2)	C(65)	C(32)	115.8(3)
N(2)	C(65)	H(5)	108.0	C(29)	C(65)	C(32)	111.5(3)
C(29)	C(65)	H(5)	109.3	C(32)	C(65)	H(5)	109.4
O(2)	C(66)	H(50)	108.3	O(2)	C(66)	H(51)	110.9
O(2)	C(66)	H(52)	109.2	H(50)	C(66)	H(51)	109.5
H(50)	C(66)	H(52)	109.5	H(51)	C(66)	H(52)	109.5
O(15)	C(67)	H(14)	110.1	O(15)	C(67)	H(15)	111.0
O(15)	C(67)	H(16)	107.3	H(14)	C(67)	H(15)	109.5
H(14)	C(67)	H(16)	109.5	H(15)	C(67)	H(16)	109.5
O(16)	C(68)	H(53)	109.7	O(16)	C(68)	H(54)	110.3
O(16)	C(68)	H(55)	108.5	H(53)	C(68)	H(54)	109.5
H(53)	C(68)	H(55)	109.5	H(54)	C(68)	H(55)	109.5
O(1)	C(69)	H(17)	109.3	O(1)	C(69)	H(18)	110.3
O(1)	C(69)	H(19)	108.8	H(17)	C(69)	H(18)	109.5
H(17)	C(69)	H(19)	109.5	H(18)	C(69)	H(19)	109.5

Table 4. Bond angles (o) (continued)

atom	atom	atom	angle	atom	atom	atom	angle
O(14)	C(70)	H(56)	109.0	O(14)	C(70)	H(57)	111.1
O(14)	C(70)	H(58)	108.4	H(56)	C(70)	H(57)	109.5
H(56)	C(70)	H(58)	109.5	H(57)	C(70)	H(58)	109.5
C(63)	C(71)	H(59)	109.2	C(63)	C(71)	H(60)	111.8
C(63)	C(71)	H(61)	107.4	H(59)	C(71)	H(60)	109.5
H(59)	C(71)	H(61)	109.5	H(60)	C(71)	H(61)	109.5
C(47)	C(72)	H(20)	107.7	C(47)	C(72)	H(21)	113.0
C(47)	C(72)	H(22)	107.7	H(20)	C(72)	H(21)	109.5
H(20)	C(72)	H(22)	109.5	H(21)	C(72)	H(22)	109.5
C(63)	C(74)	H(62)	108.4	C(63)	C(74)	H(63)	111.9
C(63)	C(74)	H(64)	108.1	H(62)	C(74)	H(63)	109.5
H(62)	C(74)	H(64)	109.5	H(63)	C(74)	H(64)	109.5
C(43)	C(75)	H(80)	109.9	C(43)	C(75)	H(81)	113.1
C(43)	C(75)	H(82)	105.3	H(80)	C(75)	H(81)	109.5
H(80)	C(75)	H(82)	109.5	H(81)	C(75)	H(82)	109.5
C(47)	C(76)	H(23)	109.6	C(47)	C(76)	H(24)	112.5
C(47)	C(76)	H(25)	106.3	H(23)	C(76)	H(24)	109.5
H(23)	C(76)	H(25)	109.5	H(24)	C(76)	H(25)	109.5
O(11)	C(77)	H(26)	109.6	O(11)	C(77)	H(27)	109.7
O(11)	C(77)	H(28)	109.1	H(26)	C(77)	H(27)	109.5
H(26)	C(77)	H(28)	109.5	H(27)	C(77)	H(28)	109.5
O(18)	C(78)	H(29)	108.5	O(18)	C(78)	H(30)	111.8
O(18)	C(78)	H(31)	108.1	H(29)	C(78)	H(30)	109.5
H(29)	C(78)	H(31)	109.5	H(30)	C(78)	H(31)	109.5
O(20)	C(79)	H(65)	110.3	O(20)	C(79)	H(66)	110.1
O(20)	C(79)	H(67)	108.0	H(65)	C(79)	H(66)	109.5
H(65)	C(79)	H(67)	109.5	H(66)	C(79)	H(67)	109.5
O(12)	C(80)	H(68)	109.2	O(12)	C(80)	H(69)	110.8
O(12)	C(80)	H(70)	108.5	H(68)	C(80)	H(69)	109.5
H(68)	C(80)	H(70)	109.5	H(69)	C(80)	H(70)	109.5
C(43)	C(81)	H(83)	110.0	C(43)	C(81)	H(84)	111.3
C(43)	C(81)	H(85)	107.1	H(83)	C(81)	H(84)	109.5
H(83)	C(81)	H(85)	109.5	H(84)	C(81)	H(85)	109.5
O(19)	C(82)	H(32)	108.6	O(19)	C(82)	H(33)	110.1
O(19)	C(82)	H(34)	109.7	H(32)	C(82)	H(33)	109.5
H(32)	C(82)	H(34)	109.5	H(33)	C(82)	H(34)	109.5
C(43)	C(83)	H(86)	110.2	C(43)	C(83)	H(87)	112.0

Table 4. Bond angles (^{o}) (continued)

atom	atom	atom	angle	atom	atom	atom	angle
C(43)	C(83)	H(88)	106.1	H(86)	C(83)	H(87)	109.5
H(86)	C(83)	H(88)	109.5	H(87)	C(83)	H(88)	109.5
C(44)	C(84)	H(71)	109.4	C(44)	C(84)	H(72)	111.0
C(44)	C(84)	H(73)	108.0	H(71)	C(84)	H(72)	109.5
H(71)	C(84)	H(73)	109.5	H(72)	C(84)	H(73)	109.5
C(44)	C(85)	H(74)	108.2	C(44)	C(85)	H(75)	112.1
C(44)	C(85)	H(76)	108.1	H(74)	C(85)	H(75)	109.5
H(74)	C(85)	H(76)	109.5	H(75)	C(85)	H(76)	109.5
C(44)	C(86)	H(77)	111.8	C(44)	C(86)	H(78)	110.0
C(44)	C(86)	H(79)	106.6	H(77)	C(86)	H(78)	109.5
H(77)	C(86)	H(79)	109.5	H(78)	C(86)	H(79)	109.5

Table 5. Torsion Angles(^{o})

atom1	atom2	atom3	atom4	angle	atom1	atom2	atom3	atom4	angle
C(69)	O(1)	C(51)	C(27)	90.5(4)	C(69)	O(1)	C(51)	C(36)	-91.6(4)
C(66)	O(2)	C(4)	C(41)	-177.8(3)	C(66)	O(2)	C(4)	C(49)	2.4(5)
C(46)	O(8)	C(55)	C(25)	-71.6(5)	C(46)	O(8)	C(55)	C(50)	109.9(4)
C(61)	O(9)	C(40)	C(45)	1.3(5)	C(61)	O(9)	C(40)	C(58)	-177.3(3)
C(77)	O(11)	C(58)	C(40)	83.5(4)	C(77)	O(11)	C(58)	C(64)	-100.1(4)
C(80)	O(12)	C(41)	C(4)	74.7(4)	C(80)	O(12)	C(41)	C(37)	-109.8(4)
C(70)	O(14)	C(37)	C(35)	-164.6(3)	C(70)	O(14)	C(37)	C(41)	17.2(5)
C(67)	O(15)	C(42)	C(1)	-0.2(5)	C(67)	O(15)	C(42)	C(36)	179.5(3)
C(68)	O(16)	C(25)	C(39)	106.1(3)	C(68)	O(16)	C(25)	C(55)	-78.2(4)
C(78)	O(18)	C(36)	C(42)	87.9(4)	C(78)	O(18)	C(36)	C(51)	-90.8(5)
C(82)	O(19)	C(64)	C(29)	147.2(4)	C(82)	O(19)	C(64)	C(58)	-35.7(6)
C(79)	O(20)	C(50)	C(54)	1.8(6)	C(79)	O(20)	C(50)	C(55)	-177.3(3)
C(32)	N(1)	C(34)	O(4)	0.8(5)	C(32)	N(1)	C(34)	C(43)	178.6(3)
C(34)	N(1)	C(32)	C(28)	-79.3(4)	C(34)	N(1)	C(32)	C(65)	156.1(3)
C(38)	N(2)	C(60)	C(52)	-158.4(4)	C(60)	N(2)	C(38)	O(7)	-174.1(3)
C(60)	N(2)	C(38)	C(47)	8.6(6)	C(38)	N(2)	C(65)	C(29)	158.0(3)
C(38)	N(2)	C(65)	C(32)	-80.4(4)	C(65)	N(2)	C(38)	O(7)	10.0(6)
C(65)	N(2)	C(38)	C(47)	-167.3(3)	C(60)	N(2)	C(65)	C(29)	-18.7(3)
C(60)	N(2)	C(65)	C(32)	102.9(3)	C(65)	N(2)	C(60)	C(52)	17.8(3)
C(2)	N(3)	C(3)	O(13)	-1.1(5)	C(2)	N(3)	C(3)	C(44)	178.9(3)
C(3)	N(3)	C(2)	C(31)	161.6(3)	C(3)	N(3)	C(2)	C(33)	-73.7(4)
C(30)	N(4)	C(31)	C(2)	-86.7(4)	C(30)	N(4)	C(31)	C(35)	152.1(3)
C(31)	N(4)	C(30)	O(3)	9.3(5)	C(31)	N(4)	C(30)	C(63)	-168.1(3)
C(30)	N(4)	C(53)	C(48)	-152.2(3)	C(53)	N(4)	C(30)	O(3)	179.7(3)
C(53)	N(4)	C(30)	C(63)	2.3(5)	C(31)	N(4)	C(53)	C(48)	18.8(3)
C(53)	N(4)	C(31)	C(2)	101.0(3)	C(53)	N(4)	C(31)	C(35)	-20.2(3)
C(28)	C(1)	C(42)	O(15)	-178.5(3)	C(28)	C(1)	C(42)	C(36)	1.7(6)
C(42)	C(1)	C(28)	C(27)	0.0(4)	C(42)	C(1)	C(28)	C(32)	-174.6(3)
N(3)	C(2)	C(31)	N(4)	78.5(3)	N(3)	C(2)	C(31)	C(35)	-166.4(2)
N(3)	C(2)	C(33)	C(39)	156.9(3)	N(3)	C(2)	C(33)	C(54)	-30.9(4)
C(31)	C(2)	C(33)	C(39)	-80.7(4)	C(31)	C(2)	C(33)	C(54)	91.5(3)
C(33)	C(2)	C(31)	N(4)	-45.5(4)	C(33)	C(2)	C(31)	C(35)	69.5(3)
O(13)	C(3)	C(44)	C(84)	-177.2(4)	O(13)	C(3)	C(44)	C(85)	63.6(5)
O(13)	C(3)	C(44)	C(86)	-53.7(5)	N(3)	C(3)	C(44)	C(84)	2.7(5)
N(3)	C(3)	C(44)	C(85)	-116.4(4)	N(3)	C(3)	C(44)	C(86)	126.2(4)
O(2)	C(4)	C(41)	O(12)	-3.3(5)	O(2)	C(4)	C(41)	C(37)	-179.0(3)
O(2)	C(4)	C(49)	C(48)	178.2(3)	C(41)	C(4)	C(49)	C(48)	-1.6(5)

Table 5. Torsion angles (^{o}) (continued)

atom1	atom2	atom3	atom4	angle
C(49)	C(4)	C(41)	O(12)	176.4(3)
O(6)	C(5)	C(27)	C(28)	164.4(4)
O(16)	C(25)	C(39)	C(33)	179.8(3)
O(16)	C(25)	C(55)	O(8)	1.6(6)
C(39)	C(25)	C(55)	O(8)	177.1(3)
C(55)	C(25)	C(39)	C(33)	4.1(6)
C(5)	C(27)	C(28)	C(1)	170.3(3)
C(5)	C(27)	C(51)	O(1)	8.2(6)
C(28)	C(27)	C(51)	O(1)	179.1(3)
C(51)	C(27)	C(28)	C(1)	-1.5(5)
C(1)	C(28)	C(32)	N(1)	-35.8(4)
C(27)	C(28)	C(32)	N(1)	149.7(3)
C(52)	C(29)	C(64)	O(19)	177.9(3)
C(64)	C(29)	C(52)	C(45)	-0.4(5)
C(52)	C(29)	C(65)	N(2)	12.5(3)
C(65)	C(29)	C(52)	C(45)	177.5(3)
C(64)	C(29)	C(65)	N(2)	-169.8(3)
C(65)	C(29)	C(64)	O(19)	0.4(5)
O(3)	C(30)	C(63)	C(56)	-104.4(4)
O(3)	C(30)	C(63)	C(74)	11.6(5)
N(4)	C(30)	C(63)	C(71)	-52.2(4)
N(4)	C(31)	C(35)	C(37)	-165.9(3)
C(2)	C(31)	C(35)	C(37)	71.0(4)
N(1)	C(32)	C(65)	N(2)	81.3(3)
C(28)	C(32)	C(65)	N(2)	-44.2(4)
C(2)	C(33)	C(39)	C(25)	171.8(3)
C(2)	C(33)	C(54)	C(50)	-175.4(3)
C(54)	C(33)	C(39)	C(25)	-0.6(5)
O(4)	C(34)	C(43)	C(75)	-58.8(5)
O(4)	C(34)	C(43)	C(83)	55.9(5)
N(1)	C(34)	C(43)	C(81)	1.0(6)
C(31)	C(35)	C(37)	O(14)	4.1(5)
C(31)	C(35)	C(48)	C(49)	176.5(3)
C(37)	C(35)	C(48)	C(49)	-3.7(5)
C(48)	C(35)	C(37)	O(14)	-175.7(3)
O(18)	C(36)	C(42)	O(15)	-0.5(6)
O(18)	C(36)	C(51)	O(1)	1.4(6)

atom1	atom2	atom3	atom4	angle
C(49)	C(4)	C(41)	C(37)	0.8(6)
O(6)	C(5)	C(27)	C(51)	-24.5(7)
O(16)	C(25)	C(39)	C(57)	-0.2(4)
O(16)	C(25)	C(55)	C(50)	-179.8(3)
C(39)	C(25)	C(55)	C(50)	-4.2(6)
C(55)	C(25)	C(39)	C(57)	-175.9(3)
C(5)	C(27)	C(28)	C(32)	-15.3(5)
C(5)	C(27)	C(51)	C(36)	-169.6(4)
C(28)	C(27)	C(51)	C(36)	1.2(6)
C(51)	C(27)	C(28)	C(32)	172.9(3)
C(1)	C(28)	C(32)	C(65)	89.4(4)
C(27)	C(28)	C(32)	C(65)	-85.1(4)
C(52)	C(29)	C(64)	C(58)	0.7(5)
C(64)	C(29)	C(52)	C(60)	-180(179)
C(52)	C(29)	C(65)	C(32)	-112.0(3)
C(65)	C(29)	C(52)	C(60)	-1.9(4)
C(64)	C(29)	C(65)	C(32)	65.8(4)
C(65)	C(29)	C(64)	C(58)	-176.9(3)
O(3)	C(30)	C(63)	C(71)	130.5(4)
N(4)	C(30)	C(63)	C(56)	72.9(4)
N(4)	C(30)	C(63)	C(74)	-171.0(3)
N(4)	C(31)	C(35)	C(48)	13.9(3)
C(2)	C(31)	C(35)	C(48)	-109.2(3)
N(1)	C(32)	C(65)	C(29)	-162.1(2)
C(28)	C(32)	C(65)	C(29)	72.3(4)
C(2)	C(33)	C(39)	C(57)	-8.2(5)
C(39)	C(33)	C(54)	C(50)	-2.7(5)
C(54)	C(33)	C(39)	C(57)	179.4(3)
O(4)	C(34)	C(43)	C(81)	178.9(4)
N(1)	C(34)	C(43)	C(75)	123.3(4)
N(1)	C(34)	C(43)	C(83)	-121.9(4)
C(31)	C(35)	C(37)	C(41)	-177.5(3)
C(31)	C(35)	C(48)	C(53)	-2.9(4)
C(37)	C(35)	C(48)	C(53)	176.9(3)
C(48)	C(35)	C(37)	C(41)	2.7(5)
O(18)	C(36)	C(42)	C(1)	179.3(3)
O(18)	C(36)	C(51)	C(27)	179.3(3)

Table 5. Torsion angles (o) (continued)

atom1	atom2	atom3	atom4	angle	atom1	atom2	atom3	atom4	angle
C(42)	C(36)	C(51)	O(1)	-177.3(3)	C(42)	C(36)	C(51)	C(27)	0.5(6)
C(51)	C(36)	C(42)	O(15)	178.3(3)	C(51)	C(36)	C(42)	C(1)	-1.9(6)
O(14)	C(37)	C(41)	O(12)	1.3(6)	O(14)	C(37)	C(41)	C(4)	176.9(3)
C(35)	C(37)	C(41)	O(12)	-176.8(3)	C(35)	C(37)	C(41)	C(4)	-1.2(5)
O(7)	C(38)	C(47)	C(62)	-112.3(4)	O(7)	C(38)	C(47)	C(72)	4.0(5)
O(7)	C(38)	C(47)	C(76)	123.8(4)	N(2)	C(38)	C(47)	C(62)	65.1(5)
N(2)	C(38)	C(47)	C(72)	-178.6(3)	N(2)	C(38)	C(47)	C(76)	-58.9(5)
C(25)	C(39)	C(57)	O(5)	139.6(4)	C(33)	C(39)	C(57)	O(5)	-40.5(6)
O(9)	C(40)	C(45)	C(52)	178.8(3)	O(9)	C(40)	C(58)	O(11)	-1.9(5)
O(9)	C(40)	C(58)	C(64)	-178.3(3)	C(45)	C(40)	C(58)	O(11)	179.5(3)
C(45)	C(40)	C(58)	C(64)	3.0(6)	C(58)	C(40)	C(45)	C(52)	-2.8(5)
C(40)	C(45)	C(52)	C(29)	1.5(5)	C(40)	C(45)	C(52)	C(60)	-179.2(3)
C(35)	C(48)	C(49)	C(4)	3.0(5)	C(35)	C(48)	C(53)	N(4)	-9.5(3)
C(49)	C(48)	C(53)	N(4)	171.1(3)	C(53)	C(48)	C(49)	C(4)	-177.7(3)
O(20)	C(50)	C(54)	C(33)	-176.5(3)	O(20)	C(50)	C(55)	O(8)	-1.3(5)
O(20)	C(50)	C(55)	C(25)	-179.9(3)	C(54)	C(50)	C(55)	O(8)	179.6(3)
C(54)	C(50)	C(55)	C(25)	1.0(6)	C(55)	C(50)	C(54)	C(33)	2.5(6)
C(29)	C(52)	C(60)	N(2)	-9.6(4)	C(45)	C(52)	C(60)	N(2)	170.9(3)
O(11)	C(58)	C(64)	O(19)	4.7(6)	O(11)	C(58)	C(64)	C(29)	-178.4(3)
C(40)	C(58)	C(64)	O(19)	-178.9(3)	C(40)	C(58)	C(64)	C(29)	-2.0(6)

The sign is positive if when looking from atom 2 to atom 3 a clock-wise motion of atom 1 would superimpose it on atom 4.

Table 6. Distances beyond the asymmetric unit out to 3.60 Å

atom	atom	distance	atom	atom	distance
O(1)	H(3)[1]	3.195	O(1)	H(6)[1]	2.998
O(1)	H(11)[1]	3.236	O(1)	H(66)[2]	2.841
O(2)	C(65)[3]	3.568(4)	O(2)	H(5)[3]	2.673
O(3)	C(61)[1]	3.541(4)	O(3)	H(8)[1]	3.144
O(3)	H(9)[1]	3.181	O(4)	C(61)[1]	3.498(4)
O(4)	C(69)[1]	3.084(4)	O(4)	H(9)[1]	3.261
O(4)	H(10)[1]	2.879	O(4)	H(17)[1]	2.733
O(4)	H(18)[1]	2.821	O(4)	H(19)[1]	3.200
O(5)	C(53)[4]	3.596(4)	O(5)	C(68)[4]	3.375(5)
O(5)	H(43)[4]	2.848	O(5)	H(53)[4]	3.448
O(5)	H(55)[4]	2.528	O(5)	H(61)[4]	3.573
O(6)	C(60)[1]	3.240(5)	O(6)	H(3)[1]	3.385
O(6)	H(6)[1]	2.942	O(6)	H(7)[1]	2.729
O(6)	H(24)[1]	3.482	O(6)	H(25)[1]	3.011
O(7)	H(56)	3.553	O(7)	H(84)[3]	3.268
O(8)	C(70)[5]	3.242(5)	O(8)	H(57)[5]	2.782
O(8)	H(58)[5]	2.838	O(9)	H(49)[6]	3.206
O(9)	H(62)[6]	3.035	O(9)	H(75)[7]	3.068
O(11)	H(62)[6]	3.344	O(11)	H(74)[7]	3.195
O(12)	H(5)[3]	3.511	O(12)	H(80)[3]	3.420
O(12)	H(83)	3.295	O(12)	H(84)	3.585
O(12)	H(85)[3]	3.255	O(13)	C(66)[4]	3.464(4)
O(13)	C(68)[4]	3.009(4)	O(13)	H(15)	3.432
O(13)	H(51)[4]	3.330	O(13)	H(52)[4]	2.748
O(13)	H(53)[4]	2.507	O(13)	H(54)[4]	2.848
O(13)	H(55)[4]	3.214	O(14)	C(67)	3.416(5)
O(14)	H(14)	3.555	O(14)	H(15)	3.286
O(14)	H(16)	2.888	O(14)	H(83)	2.975
O(15)	C(77)[6]	3.591(5)	O(15)	H(27)[6]	2.906
O(15)	H(28)[6]	3.541	O(15)	H(73)	3.544
O(15)	H(77)	3.232	O(16)	C(53)[4]	3.389(4)
O(16)	H(37)[4]	3.520	O(16)	H(42)[4]	2.529
O(16)	H(43)[4]	3.569	O(16)	H(47)[4]	2.687
O(16)	H(60)[4]	3.244	O(16)	H(61)[4]	3.422
O(18)	H(27)[6]	3.121	O(18)	H(28)[6]	3.481
O(18)	H(33)[6]	3.031	O(18)	H(34)[6]	3.586
O(19)	H(68)[3]	2.893	O(20)	C(62)[8]	3.455(5)

Table 6. Distances beyond the asymmetric unit out to 3.60 Å (continued)

atom	atom	distance	atom	atom	distance
O(20)	H(11)$^{8)}$	3.391	O(20)	H(12)$^{8)}$	3.212
O(20)	H(13)$^{8)}$	3.194	O(20)	H(50)$^{4)}$	3.597
O(20)	H(69)$^{5)}$	3.197	O(20)	H(70)$^{5)}$	3.538
N(3)	H(15)	2.793	C(1)	H(10)$^{1)}$	3.406
C(2)	H(15)	3.172	C(3)	H(15)	3.018
C(3)	H(52)$^{4)}$	3.514	C(5)	H(3)$^{1)}$	3.090
C(5)	H(7)$^{1)}$	3.244	C(5)	H(19)$^{1)}$	3.036
C(25)	H(37)$^{4)}$	3.127	C(25)	H(42)$^{4)}$	3.335
C(27)	H(3)$^{1)}$	3.026	C(27)	H(10)$^{1)}$	3.302
C(28)	H(10)$^{1)}$	3.243	C(29)	H(51)$^{3)}$	3.212
C(30)	H(9)$^{1)}$	3.523	C(31)	H(15)	3.449
C(33)	H(37)$^{4)}$	3.581	C(33)	H(52)$^{4)}$	3.519
C(35)	H(87)	3.099	C(36)	H(8)$^{1)}$	3.480
C(37)	H(83)	3.142	C(37)	H(87)	3.107
C(39)	H(37)$^{4)}$	3.120	C(40)	C(85)$^{7)}$	3.583(5)
C(40)	H(74)$^{7)}$	3.343	C(40)	H(75)$^{7)}$	3.048
C(41)	H(83)	3.324	C(41)	H(87)	3.402
C(42)	H(8)$^{1)}$	3.400	C(46)	H(20)$^{8)}$	3.597
C(46)	H(21)$^{5)}$	3.217	C(46)	H(57)$^{5)}$	3.279
C(48)	H(87)	3.337	C(49)	H(46)$^{5)}$	3.384
C(50)	H(50)$^{4)}$	3.461	C(51)	H(3)$^{1)}$	3.063
C(51)	H(10)$^{1)}$	3.558	C(53)	O(5)$^{5)}$	3.596(4)
C(53)	O(16)$^{5)}$	3.389(4)	C(53)	C(57)$^{5)}$	3.573(5)
C(54)	H(52)$^{4)}$	3.444	C(55)	H(37)$^{4)}$	3.518
C(56)	H(9)$^{1)}$	3.568	C(56)	H(26)$^{1)}$	3.226
C(56)	H(54)$^{5)}$	3.120	C(57)	C(53)$^{4)}$	3.573(5)
C(57)	H(37)$^{4)}$	3.567	C(57)	H(42)$^{4)}$	3.276
C(57)	H(43)$^{4)}$	3.045	C(57)	H(61)$^{4)}$	3.357
C(58)	H(74)$^{7)}$	3.017	C(58)	H(75)$^{7)}$	3.374
C(60)	O(6)$^{6)}$	3.240(5)	C(61)	O(3)$^{6)}$	3.541(4)
C(61)	O(4)$^{6)}$	3.498(4)	C(61)	H(31)$^{6)}$	3.536
C(61)	H(49)$^{6)}$	3.316	C(61)	H(88)$^{6)}$	3.317
C(62)	O(20)$^{7)}$	3.455(5)	C(62)	H(44)$^{7)}$	3.184
C(62)	H(50)$^{3)}$	3.324	C(62)	H(66)$^{7)}$	3.080
C(62)	H(82)$^{9)}$	3.381	C(64)	H(51)$^{3)}$	3.452
C(64)	H(74)$^{7)}$	3.361	C(65)	O(2)$^{3)}$	3.568(4)
C(65)	H(51)$^{3)}$	3.419	C(65)	H(68)$^{3)}$	3.460

Table 6. Distances beyond the asymmetric unit out to 3.60 Å (continued)

atom	atom	distance	atom	atom	distance
C(66)	O(13)[5]	3.464(4)	C(66)	H(5)[3]	3.106
C(66)	H(13)[3]	3.296	C(66)	H(76)[5]	3.294
C(67)	O(14)	3.416(5)	C(67)	H(41)	3.282
C(67)	H(57)	3.340	C(67)	H(77)	3.588
C(68)	O(5)[5]	3.375(5)	C(68)	O(13)[5]	3.009(4)
C(68)	H(40)[5]	3.517	C(68)	H(47)[4]	2.960
C(68)	H(60)[4]	3.165	C(69)	O(4)[6]	3.084(4)
C(69)	H(11)[1]	3.307	C(69)	H(35)[6]	3.115
C(69)	H(66)[2]	3.011	C(70)	O(8)[4]	3.242(5)
C(70)	H(14)	3.556	C(70)	H(16)	3.386
C(70)	H(22)	3.305	C(70)	H(45)[4]	3.571
C(70)	H(55)[4]	3.563	C(70)	H(80)[3]	3.586
C(70)	H(83)	3.271	C(71)	H(32)[10]	3.045
C(71)	H(39)[5]	3.412	C(72)	H(45)[4]	3.258
C(72)	H(56)	3.542	C(72)	H(81)[3]	3.488
C(74)	H(72)[2]	3.330	C(74)	H(78)[2]	3.278
C(75)	H(12)[11]	3.250	C(75)	H(69)[3]	3.536
C(77)	O(15)[1]	3.591(5)	C(77)	H(48)[6]	3.292
C(77)	H(77)[1]	3.545	C(78)	C(79)[2]	3.582(6)
C(78)	H(8)[1]	3.413	C(78)	H(33)[6]	3.486
C(78)	H(65)[2]	3.317	C(78)	H(66)[2]	3.557
C(78)	H(67)[2]	3.294	C(78)	H(71)[2]	3.302
C(78)	H(72)	3.263	C(78)	H(73)	3.499
C(79)	C(78)[2]	3.582(6)	C(79)	H(11)[8]	3.242
C(79)	H(13)[8]	3.543	C(79)	H(18)[2]	3.205
C(79)	H(30)[2]	2.892	C(79)	H(31)[2]	3.595
C(79)	H(33)[10]	3.371	C(80)	H(5)[3]	3.445
C(80)	H(32)[3]	3.322	C(80)	H(80)[3]	3.566
C(81)	C(81)[3]	3.452(7)	C(81)	H(56)	3.494
C(81)	H(84)[3]	3.052	C(81)	H(85)[3]	3.114
C(82)	H(59)[12]	2.842	C(82)	H(63)[12]	3.543
C(82)	H(65)[12]	3.373	C(82)	H(68)[3]	3.055
C(83)	H(9)[1]	3.264	C(84)	H(29)	2.917
C(84)	H(64)[2]	3.501	C(84)	H(71)[2]	3.437
C(85)	C(40)[8]	3.583(5)	C(85)	H(51)[4]	3.175
C(86)	H(15)	3.595	C(86)	H(27)[6]	3.495
H(3)	O(1)[6]	3.195	H(3)	O(6)[6]	3.385

Table 6. Distances beyond the asymmetric unit out to 3.60 Å (continued)

atom	atom	distance	atom	atom	distance
H(3)	C(5)$^{6)}$	3.090	H(3)	C(27)$^{6)}$	3.026
H(3)	C(51)$^{6)}$	3.063	H(3)	H(31)$^{6)}$	3.367
H(3)	H(35)$^{6)}$	3.407	H(4)	H(18)$^{1)}$	3.388
H(4)	H(19)$^{1)}$	3.112	H(4)	H(68)$^{3)}$	3.482
H(5)	O(2)$^{3)}$	2.673	H(5)	O(12)$^{3)}$	3.511
H(5)	C(66)$^{3)}$	3.106	H(5)	C(80)$^{3)}$	3.445
H(5)	H(50)$^{3)}$	3.208	H(5)	H(51)$^{3)}$	2.875
H(5)	H(68)$^{3)}$	2.685	H(6)	O(1)$^{6)}$	2.998
H(6)	O(6)$^{6)}$	2.942	H(7)	O(6)$^{6)}$	2.729
H(7)	C(5)$^{6)}$	3.244	H(7)	H(35)$^{6)}$	3.458
H(8)	O(3)$^{6)}$	3.144	H(8)	C(36)$^{6)}$	3.480
H(8)	C(42)$^{6)}$	3.400	H(8)	C(78)$^{6)}$	3.413
H(8)	H(29)$^{6)}$	3.311	H(8)	H(31)$^{6)}$	2.728
H(8)	H(75)$^{7)}$	3.198	H(9)	O(3)$^{6)}$	3.181
H(9)	O(4)$^{6)}$	3.261	H(9)	C(30)$^{6)}$	3.523
H(9)	C(56)$^{6)}$	3.568	H(9)	C(83)$^{6)}$	3.264
H(9)	H(41)$^{6)}$	3.493	H(9)	H(49)$^{6)}$	2.666
H(9)	H(86)$^{6)}$	3.239	H(9)	H(88)$^{6)}$	2.481
H(10)	O(4)$^{6)}$	2.879	H(10)	C(1)$^{6)}$	3.406
H(10)	C(27)$^{6)}$	3.302	H(10)	C(28)$^{6)}$	3.243
H(10)	C(51)$^{6)}$	3.558	H(10)	H(88)$^{6)}$	3.385
H(11)	O(1)$^{6)}$	3.236	H(11)	O(20)$^{7)}$	3.391
H(11)	C(69)$^{6)}$	3.307	H(11)	C(79)$^{7)}$	3.242
H(11)	H(17)$^{6)}$	3.331	H(11)	H(18)$^{6)}$	2.880
H(11)	H(66)$^{7)}$	2.488	H(11)	H(67)$^{7)}$	3.568
H(11)	H(82)$^{9)}$	3.447	H(12)	O(20)$^{7)}$	3.212
H(12)	C(75)$^{9)}$	3.250	H(12)	H(18)$^{6)}$	3.583
H(12)	H(44)$^{7)}$	2.773	H(12)	H(66)$^{7)}$	3.140
H(12)	H(80)$^{9)}$	3.360	H(12)	H(81)$^{9)}$	3.255
H(12)	H(82)$^{9)}$	2.647	H(13)	O(20)$^{7)}$	3.194
H(13)	C(66)$^{3)}$	3.296	H(13)	C(79)$^{7)}$	3.543
H(13)	H(44)$^{7)}$	2.756	H(13)	H(50)$^{3)}$	2.393
H(13)	H(51)$^{3)}$	3.564	H(13)	H(66)$^{7)}$	3.143
H(14)	O(14)	3.555	H(14)	C(70)	3.556
H(14)	H(27)$^{6)}$	3.448	H(14)	H(57)	2.940
H(15)	O(13)	3.432	H(15)	O(14)	3.286
H(15)	N(3)	2.793	H(15)	C(2)	3.172

Table 6. Distances beyond the asymmetric unit out to 3.60 Å (continued)

atom	atom	distance	atom	atom	distance
H(15)	C(3)	3.018	H(15)	C(31)	3.449
H(15)	C(86)	3.595	H(15)	H(36)	3.004
H(15)	H(40)	2.823	H(15)	H(41)	2.871
H(15)	H(54)$^{4)}$	3.479	H(15)	H(57)	3.370
H(15)	H(73)	3.304	H(15)	H(77)	3.142
H(15)	H(79)	3.515	H(16)	O(14)	2.888
H(16)	C(70)	3.386	H(16)	H(41)	2.845
H(16)	H(56)	3.492	H(16)	H(57)	3.169
H(17)	O(4)$^{6)}$	2.733	H(17)	H(11)$^{1)}$	3.331
H(17)	H(24)$^{1)}$	3.118	H(17)	H(35)$^{6)}$	3.404
H(18)	O(4)$^{6)}$	2.821	H(18)	C(79)$^{2)}$	3.205
H(18)	H(4)$^{6)}$	3.388	H(18)	H(11)$^{1)}$	2.880
H(18)	H(12)$^{1)}$	3.583	H(18)	H(33)$^{6)}$	3.520
H(18)	H(35)$^{6)}$	3.377	H(18)	H(65)$^{2)}$	3.274
H(18)	H(66)$^{2)}$	2.380	H(18)	H(82)$^{6)}$	3.352
H(19)	O(4)$^{6)}$	3.200	H(19)	C(5)$^{6)}$	3.036
H(19)	H(4)$^{6)}$	3.112	H(19)	H(33)$^{6)}$	3.552
H(19)	H(35)$^{6)}$	2.247	H(19)	H(66)$^{2)}$	3.575
H(20)	C(46)$^{7)}$	3.597	H(20)	H(20)$^{13)}$	3.519
H(20)	H(21)$^{13)}$	3.485	H(20)	H(44)$^{7)}$	2.732
H(20)	H(80)$^{3)}$	3.588	H(20)	H(81)$^{3)}$	2.915
H(21)	C(46)$^{4)}$	3.217	H(21)	H(20)$^{13)}$	3.485
H(21)	H(44)$^{4)}$	3.214	H(21)	H(45)$^{4)}$	2.424
H(21)	H(57)	3.548	H(21)	H(81)$^{9)}$	3.027
H(21)	H(82)$^{9)}$	3.526	H(22)	C(70)	3.305
H(22)	H(45)$^{4)}$	3.318	H(22)	H(56)	2.654
H(22)	H(57)	3.107	H(22)	H(80)$^{3)}$	3.219
H(22)	H(81)$^{3)}$	3.255	H(22)	H(84)$^{3)}$	3.587
H(23)	H(26)$^{6)}$	3.564	H(23)	H(45)$^{4)}$	3.396
H(24)	O(6)$^{6)}$	3.482	H(24)	H(17)$^{6)}$	3.118
H(24)	H(82)$^{9)}$	3.045	H(24)	H(86)$^{9)}$	3.493
H(25)	O(6)$^{6)}$	3.011	H(25)	H(28)$^{6)}$	3.273
H(26)	C(56)$^{6)}$	3.226	H(26)	H(23)$^{1)}$	3.564
H(26)	H(48)$^{6)}$	2.549	H(26)	H(49)$^{6)}$	3.058
H(26)	H(62)$^{6)}$	3.326	H(27)	O(15)$^{1)}$	2.906
H(27)	O(18)$^{1)}$	3.121	H(27)	C(86)$^{1)}$	3.495
H(27)	H(14)$^{1)}$	3.448	H(27)	H(48)$^{6)}$	3.343

Table 6. Distances beyond the asymmetric unit out to 3.60 Å (continued)

atom	atom	distance	atom	atom	distance
H(27)	H(77)[1)]	2.683	H(28)	O(15)[1)]	3.541
H(28)	O(18)[1)]	3.481	H(28)	H(25)[1)]	3.273
H(29)	C(84)	2.917	H(29)	H(8)[1)]	3.311
H(29)	H(64)[2)]	3.527	H(29)	H(71)	3.567
H(29)	H(71)[2)]	3.060	H(29)	H(72)	2.423
H(29)	H(73)	2.565	H(29)	H(77)	3.123
H(30)	C(79)[2)]	2.892	H(30)	H(33)[6)]	3.013
H(30)	H(38)[2)]	3.543	H(30)	H(64)[2)]	3.241
H(30)	H(65)[2)]	2.534	H(30)	H(66)[2)]	3.076
H(30)	H(67)[2)]	2.609	H(30)	H(71)[2)]	3.122
H(30)	H(72)	3.331	H(31)	C(61)[1)]	3.536
H(31)	C(79)[2)]	3.595	H(31)	H(3)[1)]	3.367
H(31)	H(8)[1)]	2.728	H(31)	H(65)[2)]	3.581
H(31)	H(66)[2)]	3.457	H(31)	H(67)[2)]	3.178
H(31)	H(71)[2)]	3.170	H(31)	H(75)[2)]	3.127
H(31)	H(76)[2)]	3.495	H(32)	C(71)[12)]	3.045
H(32)	C(80)[3)]	3.322	H(32)	H(51)[3)]	3.514
H(32)	H(59)[12)]	2.363	H(32)	H(60)[12)]	2.911
H(32)	H(61)[12)]	3.560	H(32)	H(63)[12)]	3.419
H(32)	H(65)[12)]	3.479	H(32)	H(68)[3)]	2.618
H(32)	H(70)[3)]	3.240	H(32)	H(74)[7)]	3.483
H(33)	O(18)[1)]	3.031	H(33)	C(78)[1)]	3.486
H(33)	C(79)[12)]	3.371	H(33)	H(18)[1)]	3.520
H(33)	H(19)[1)]	3.552	H(33)	H(30)[1)]	3.013
H(33)	H(59)[12)]	2.697	H(33)	H(65)[12)]	2.511
H(33)	H(66)[12)]	3.471	H(33)	H(68)[3)]	3.169
H(34)	O(18)[1)]	3.586	H(34)	H(59)[12)]	3.024
H(34)	H(63)[12)]	3.052	H(34)	H(74)[7)]	3.396
H(35)	C(69)[1)]	3.115	H(35)	H(3)[1)]	3.407
H(35)	H(7)[1)]	3.458	H(35)	H(17)[1)]	3.404
H(35)	H(18)[1)]	3.377	H(35)	H(19)[1)]	2.247
H(36)	H(15)	3.004	H(37)	O(16)[5)]	3.520
H(37)	C(25)[5)]	3.127	H(37)	C(33)[5)]	3.581
H(37)	C(39)[5)]	3.120	H(37)	C(55)[5)]	3.518
H(37)	C(57)[5)]	3.567	H(37)	H(46)[5)]	3.067
H(38)	H(30)[2)]	3.543	H(39)	C(71)[4)]	3.412
H(39)	H(42)[4)]	3.300	H(39)	H(43)[4)]	3.216

Table 6. Distances beyond the asymmetric unit out to 3.60 Å (continued)

atom	atom	distance	atom	atom	distance
H(39)	H(60)[4)]	3.072	H(39)	H(61)[4)]	2.895
H(40)	C(68)[4)]	3.517	H(40)	H(15)	2.823
H(40)	H(53)[4)]	3.504	H(40)	H(54)[4)]	3.505
H(40)	H(55)[4)]	2.998	H(41)	C(67)	3.282
H(41)	H(9)[1)]	3.493	H(41)	H(15)	2.871
H(41)	H(16)	2.845	H(41)	H(87)	3.528
H(41)	H(88)	3.153	H(42)	O(16)[5)]	2.529
H(42)	C(25)[5)]	3.335	H(42)	C(57)[5)]	3.276
H(42)	H(39)[5)]	3.300	H(42)	H(46)[5)]	3.242
H(43)	O(5)[5)]	2.848	H(43)	O(16)[5)]	3.569
H(43)	C(57)[5)]	3.045	H(43)	H(39)[5)]	3.216
H(44)	C(62)[8)]	3.184	H(44)	H(12)[8)]	2.773
H(44)	H(13)[8)]	2.756	H(44)	H(20)[8)]	2.732
H(44)	H(21)[5)]	3.214	H(44)	H(50)[4)]	3.421
H(45)	C(70)[5)]	3.571	H(45)	C(72)[5)]	3.258
H(45)	H(21)[5)]	2.424	H(45)	H(22)[5)]	3.318
H(45)	H(23)[5)]	3.396	H(45)	H(57)[5)]	2.831
H(45)	H(86)[4)]	3.565	H(46)	C(49)[4)]	3.384
H(46)	H(37)[4)]	3.067	H(46)	H(42)[4)]	3.242
H(46)	H(47)[4)]	3.437	H(46)	H(86)[4)]	3.589
H(47)	O(16)[5)]	2.687	H(47)	C(68)[5)]	2.960
H(47)	H(46)[5)]	3.437	H(47)	H(53)[5)]	3.398
H(47)	H(54)[5)]	2.435	H(48)	C(77)[1)]	3.292
H(48)	H(26)[1)]	2.549	H(48)	H(27)[1)]	3.343
H(48)	H(54)[5)]	2.992	H(48)	H(79)[11)]	3.109
H(49)	O(9)[1)]	3.206	H(49)	C(61)[1)]	3.316
H(49)	H(9)[1)]	2.666	H(49)	H(26)[1)]	3.058
H(49)	H(86)	3.320	H(49)	H(88)	3.473
H(50)	O(20)[5)]	3.597	H(50)	C(50)[5)]	3.461
H(50)	C(62)[3)]	3.324	H(50)	H(5)[3)]	3.208
H(50)	H(13)[3)]	2.393	H(50)	H(44)[5)]	3.421
H(50)	H(67)[5)]	3.586	H(50)	H(76)[5)]	3.569
H(51)	O(13)[5)]	3.330	H(51)	C(29)[3)]	3.212
H(51)	C(64)[3)]	3.452	H(51)	C(65)[3)]	3.419
H(51)	C(85)[5)]	3.175	H(51)	H(5)[3)]	2.875
H(51)	H(13)[3)]	3.564	H(51)	H(32)[3)]	3.514
H(51)	H(74)[5)]	2.856	H(51)	H(76)[5)]	2.627

Table 6. Distances beyond the asymmetric unit out to 3.60 Å (continued)

atom	atom	distance	atom	atom	distance
H(52)	O(13)$^{5)}$	2.748	H(52)	C(3)$^{5)}$	3.514
H(52)	C(33)$^{5)}$	3.519	H(52)	C(54)$^{5)}$	3.444
H(52)	H(76)$^{5)}$	3.240	H(53)	O(5)$^{5)}$	3.448
H(53)	O(13)$^{5)}$	2.507	H(53)	H(40)$^{5)}$	3.504
H(53)	H(47)$^{4)}$	3.398	H(53)	H(60)$^{4)}$	2.858
H(54)	O(13)$^{5)}$	2.848	H(54)	C(56)$^{4)}$	3.120
H(54)	H(15)$^{5)}$	3.479	H(54)	H(40)$^{5)}$	3.505
H(54)	H(47)$^{4)}$	2.435	H(54)	H(48)$^{4)}$	2.992
H(54)	H(57)$^{5)}$	3.496	H(54)	H(60)$^{4)}$	2.978
H(54)	H(79)$^{5)}$	3.339	H(55)	O(5)$^{5)}$	2.528
H(55)	O(13)$^{5)}$	3.214	H(55)	C(70)$^{5)}$	3.563
H(55)	H(40)$^{5)}$	2.998	H(55)	H(57)$^{5)}$	3.010
H(55)	H(58)$^{5)}$	3.478	H(56)	O(7)	3.553
H(56)	C(72)	3.542	H(56)	C(81)	3.494
H(56)	H(16)	3.492	H(56)	H(22)	2.654
H(56)	H(80)$^{3)}$	2.964	H(56)	H(83)	2.674
H(56)	H(84)$^{3)}$	3.567	H(56)	H(85)	3.589
H(57)	O(8)$^{4)}$	2.782	H(57)	C(46)$^{4)}$	3.279
H(57)	C(67)	3.340	H(57)	H(14)	2.940
H(57)	H(15)	3.370	H(57)	H(16)	3.169
H(57)	H(21)	3.548	H(57)	H(22)	3.107
H(57)	H(45)$^{4)}$	2.831	H(57)	H(54)$^{4)}$	3.496
H(57)	H(55)$^{4)}$	3.010	H(58)	O(8)$^{4)}$	2.838
H(58)	H(55)$^{4)}$	3.478	H(58)	H(80)$^{3)}$	3.361
H(59)	C(82)$^{10)}$	2.842	H(59)	H(32)$^{10)}$	2.363
H(59)	H(33)$^{10)}$	2.697	H(59)	H(34)$^{10)}$	3.024
H(60)	O(16)$^{5)}$	3.244	H(60)	C(68)$^{5)}$	3.165
H(60)	H(32)$^{10)}$	2.911	H(60)	H(39)$^{5)}$	3.072
H(60)	H(53)$^{5)}$	2.858	H(60)	H(54)$^{5)}$	2.978
H(60)	H(79)$^{11)}$	3.309	H(61)	O(5)$^{5)}$	3.573
H(61)	O(16)$^{5)}$	3.422	H(61)	C(57)$^{5)}$	3.357
H(61)	H(32)$^{10)}$	3.560	H(61)	H(39)$^{5)}$	2.895
H(61)	H(70)$^{5)}$	3.540	H(62)	O(9)$^{1)}$	3.035
H(62)	O(11)$^{1)}$	3.344	H(62)	H(26)$^{1)}$	3.326
H(62)	H(72)$^{2)}$	3.373	H(62)	H(75)$^{2)}$	3.453
H(62)	H(78)$^{2)}$	2.955	H(63)	C(82)$^{10)}$	3.543
H(63)	H(32)$^{10)}$	3.419	H(63)	H(34)$^{10)}$	3.052

Table 6. Distances beyond the asymmetric unit out to 3.60 Å (continued)

atom	atom	distance	atom	atom	distance
H(63)	H(72)[2)]	3.586	H(63)	H(78)[11)]	3.543
H(63)	H(78)[2)]	3.104	H(63)	H(79)[11)]	3.409
H(64)	C(84)[2)]	3.501	H(64)	H(29)[2)]	3.527
H(64)	H(30)[2)]	3.241	H(64)	H(72)[2)]	2.599
H(64)	H(78)[2)]	3.230	H(65)	C(78)[2)]	3.317
H(65)	C(82)[10)]	3.373	H(65)	H(18)[2)]	3.274
H(65)	H(30)[2)]	2.534	H(65)	H(31)[2)]	3.581
H(65)	H(32)[10)]	3.479	H(65)	H(33)[10)]	2.511
H(65)	H(69)[5)]	3.369	H(65)	H(70)[5)]	3.556
H(66)	O(1)[2)]	2.841	H(66)	C(62)[8)]	3.080
H(66)	C(69)[2)]	3.011	H(66)	C(78)[2)]	3.557
H(66)	H(11)[8)]	2.488	H(66)	H(12)[8)]	3.140
H(66)	H(13)[8)]	3.143	H(66)	H(18)[2)]	2.380
H(66)	H(19)[2)]	3.575	H(66)	H(30)[2)]	3.076
H(66)	H(31)[2)]	3.457	H(66)	H(33)[10)]	3.471
H(67)	C(78)[2)]	3.294	H(67)	H(11)[8)]	3.568
H(67)	H(30)[2)]	2.609	H(67)	H(31)[2)]	3.178
H(67)	H(50)[4)]	3.586	H(68)	O(19)[3)]	2.893
H(68)	C(65)[3)]	3.460	H(68)	C(82)[3)]	3.055
H(68)	H(4)[3)]	3.482	H(68)	H(5)[3)]	2.685
H(68)	H(32)[3)]	2.618	H(68)	H(33)[3)]	3.169
H(69)	O(20)[4)]	3.197	H(69)	C(75)[3)]	3.536
H(69)	H(65)[4)]	3.369	H(69)	H(80)[3)]	2.891
H(69)	H(82)[3)]	3.386	H(70)	O(20)[4)]	3.538
H(70)	H(32)[3)]	3.240	H(70)	H(61)[4)]	3.540
H(70)	H(65)[4)]	3.556	H(71)	C(78)[2)]	3.302
H(71)	C(84)[2)]	3.437	H(71)	H(29)	3.567
H(71)	H(29)[2)]	3.060	H(71)	H(30)[2)]	3.122
H(71)	H(31)[2)]	3.170	H(71)	H(71)[2)]	3.240
H(71)	H(72)[2)]	3.088	H(71)	H(73)[2)]	3.423
H(72)	C(74)[2)]	3.330	H(72)	C(78)	3.263
H(72)	H(29)	2.423	H(72)	H(30)	3.331
H(72)	H(62)[2)]	3.373	H(72)	H(63)[2)]	3.586
H(72)	H(64)[2)]	2.599	H(72)	H(71)[2)]	3.088
H(73)	O(15)	3.544	H(73)	C(78)	3.499
H(73)	H(15)	3.304	H(73)	H(29)	2.565
H(73)	H(71)[2)]	3.423	H(74)	O(11)[8)]	3.195

Table 6. Distances beyond the asymmetric unit out to 3.60 Å (continued)

atom	atom	distance	atom	atom	distance
H(74)	C(40)[8]	3.343	H(74)	C(58)[8]	3.017
H(74)	C(64)[8]	3.361	H(74)	H(32)[8]	3.483
H(74)	H(34)[8]	3.396	H(74)	H(51)[4]	2.856
H(75)	O(9)[8]	3.068	H(75)	C(40)[8]	3.048
H(75)	C(58)[8]	3.374	H(75)	H(8)[8]	3.198
H(75)	H(31)[2]	3.127	H(75)	H(62)[2]	3.453
H(76)	C(66)[4]	3.294	H(76)	H(31)[2]	3.495
H(76)	H(50)[4]	3.569	H(76)	H(51)[4]	2.627
H(76)	H(52)[4]	3.240	H(77)	O(15)	3.232
H(77)	C(67)	3.588	H(77)	C(77)[6]	3.545
H(77)	H(15)	3.142	H(77)	H(27)[6]	2.683
H(77)	H(29)	3.123	H(78)	C(74)[2]	3.278
H(78)	H(62)[2]	2.955	H(78)	H(63)[9]	3.543
H(78)	H(63)[2]	3.104	H(78)	H(64)[2]	3.230
H(79)	H(15)	3.515	H(79)	H(48)[9]	3.109
H(79)	H(54)[4]	3.339	H(79)	H(60)[9]	3.309
H(79)	H(63)[9]	3.409	H(80)	O(12)[3]	3.420
H(80)	C(70)[3]	3.586	H(80)	C(80)[3]	3.566
H(80)	H(12)[11]	3.360	H(80)	H(20)[3]	3.588
H(80)	H(22)[3]	3.219	H(80)	H(56)[3]	2.964
H(80)	H(58)[3]	3.361	H(80)	H(69)[3]	2.891
H(81)	C(72)[3]	3.488	H(81)	H(12)[11]	3.255
H(81)	H(20)[3]	2.915	H(81)	H(21)[11]	3.027
H(81)	H(22)[3]	3.255	H(82)	C(62)[11]	3.381
H(82)	H(11)[11]	3.447	H(82)	H(12)[11]	2.647
H(82)	H(18)[1]	3.352	H(82)	H(21)[11]	3.526
H(82)	H(24)[11]	3.045	H(82)	H(69)[3]	3.386
H(83)	O(12)	3.295	H(83)	O(14)	2.975
H(83)	C(37)	3.142	H(83)	C(41)	3.324
H(83)	C(70)	3.271	H(83)	H(56)	2.674
H(83)	H(84)[3]	3.216	H(83)	H(85)[3]	3.275
H(84)	O(7)[3]	3.268	H(84)	O(12)	3.585
H(84)	C(81)[3]	3.052	H(84)	H(22)[3]	3.587
H(84)	H(56)[3]	3.567	H(84)	H(83)[3]	3.216
H(84)	H(84)[3]	2.919	H(84)	H(85)[3]	2.549
H(85)	O(12)[3]	3.255	H(85)	C(81)[3]	3.114
H(85)	H(56)	3.589	H(85)	H(83)[3]	3.275

Table 6. Distances beyond the asymmetric unit out to 3.60 Å (continued)

atom	atom	distance	atom	atom	distance
H(85)	H(84)[3]	2.549	H(85)	H(85)[3]	3.047
H(86)	H(9)[1]	3.239	H(86)	H(24)[11]	3.493
H(86)	H(45)[5]	3.565	H(86)	H(46)[5]	3.589
H(86)	H(49)	3.320	H(87)	C(35)	3.099
H(87)	C(37)	3.107	H(87)	C(41)	3.402
H(87)	C(48)	3.337	H(87)	H(41)	3.528
H(88)	C(61)[1]	3.317	H(88)	H(9)[1]	2.481
H(88)	H(10)[1]	3.385	H(88)	H(41)	3.153
H(88)	H(49)	3.473			

Symmetry Operators:

(1) -X+1/2+1,Y+1/2-1,-Z+1/2
(2) -X+2,-Y,-Z+1
(3) -X+1,-Y,-Z+1
(4) -X+1/2+1,Y+1/2,-Z+1/2+1
(5) -X+1/2+1,Y+1/2-1,-Z+1/2+1
(6) -X+1/2+1,Y+1/2,-Z+1/2
(7) X+1/2-1,-Y+1/2,Z+1/2-1
(8) X+1/2,-Y+1/2,Z+1/2
(9) X,Y+1,Z
(10) X+1/2,-Y+1/2-1,Z+1/2
(11) X,Y-1,Z
(12) X+1/2-1,-Y+1/2-1,Z+1/2-1
(13) -X+1,-Y+1,-Z+1

Intramolecular and Intermolecular Hydrogen bonds

D	H	A	D...A	D-H	H...A	D-H...A
N(1)	H(1)	O(7)	2.835(4)	0.950	2.010	144.1
N(3)	H(36)	O(3)	2.796(3)	0.950	1.946	147.9

Note) 1. The symmetry operations are applied to the acceptors.
2. Estimated standard deviations (esd's) are shown in the parentheses.
They are not calculated when all atoms have an esd=0.0.
